# Disentangling the Structure–Activity Relationships of Naphthalene Diimides as Anticancer G-Quadruplex-Targeting Drugs

**DOI:** 10.1021/acs.jmedchem.1c00125

**Published:** 2021-03-22

**Authors:** Chiara Platella, Ettore Napolitano, Claudia Riccardi, Domenica Musumeci, Daniela Montesarchio

**Affiliations:** † Department of Chemical Sciences, University of Naples Federico II, via Cintia 21, I-80126 Naples, Italy; ‡ Institute of Biostructures and Bioimages, CNR, via Mezzocannone 16, I-80134 Naples, Italy

## Abstract

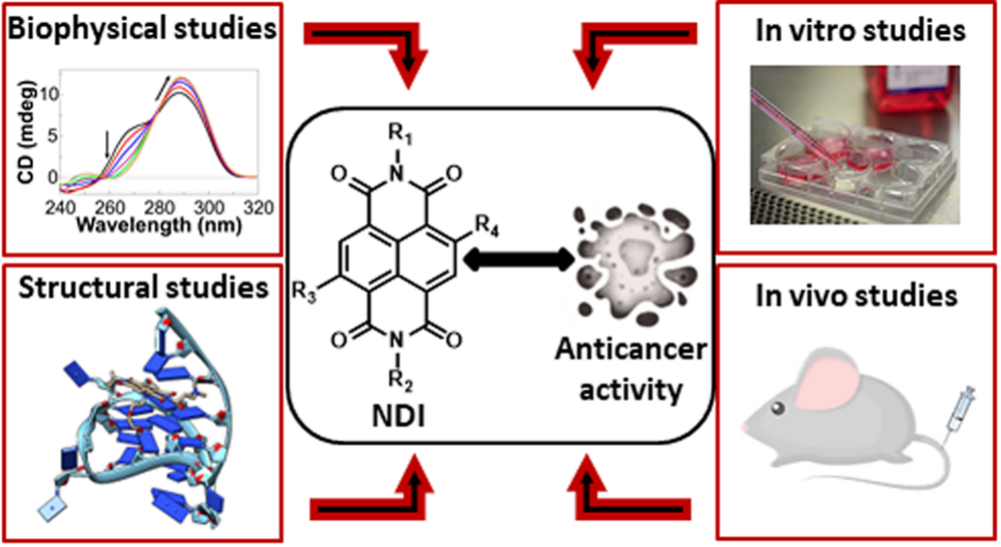

In the context of developing efficient anticancer therapies aimed at eradicating any sort of tumors, G-quadruplexes represent excellent targets. Small molecules able to interact with G-quadruplexes can interfere with cell pathways specific of tumors and common to all cancers. Naphthalene diimides (NDIs) are among the most promising, putative anticancer G-quadruplex-targeting drugs, due to their ability to simultaneously target multiple G-quadruplexes and their strong, selective in vitro and in vivo anticancer activity. Here, all the available biophysical, biological, and structural data concerning NDIs targeting G-quadruplexes were systematically analyzed. Structure–activity correlations were obtained by analyzing biophysical data of their interactions with G-quadruplex targets and control duplex structures, in parallel to biological data concerning the antiproliferative activity of NDIs on cancer and normal cells. In addition, NDI binding modes to G-quadruplexes were discussed in consideration of the structures and properties of NDIs by in-depth analysis of the available structural models of G-quadruplex/NDI complexes.

## Introduction

Precision medicine is the new frontier in cancer treatment.^1−3^ The possibility of developing ad hoc therapies targeted to each patient in relation to the specific type of cancer has attracted the interest of researchers worldwide. Promising results have been achieved in this field, and several studies are currently underway to find new targets and drugs thereof.^4,5^ Nevertheless, crucial issues are still unsolved, related to, e.g., low benefits/costs ratio and tumor heterogeneity.^6^ Indeed, once an effective drug is identified for a specific target, this could then prove to be inactive on cells of a different cancer type or even be overall toxic to the patient. In addition, cancer cells of the same tissues could exhibit different molecular features, and thus a single drug could be partially ineffective. To overcome the drawbacks related to tumor heterogeneity and increase the benefits/costs ratio of a therapeutic treatment, a very promising but still challenging strategy could be identifying key molecular targets common to all cancer cells and types.

In this context, telomeres, i.e., the 3′-ends of chromosomes, could play a central role. Indeed, uncontrolled telomere lengthening is the main mechanism responsible for cancer cell immortalization, the crucial process that makes cancer so hard to eradicate.^7^ Therefore, aiming at blocking chromosome elongation, targeting telomeres is an attractive strategy to interfere with a mechanism common to all cancer types and ultimately develop a universal cancer therapy. In addition, since telomere lengthening does not occur in normal cells, and telomeres in normal stem and germ cells are longer than in cancer cells, this approach intrinsically guarantees a specific impact against cancer cells, as well as very low toxicity to normal cells associated with low toxicity to stem/germ cells if the treatment is limited in time.^8^

In detail, telomeres are guanine-rich DNA regions able to fold into non-canonical secondary structures, named G-quadruplexes.^9^ G-quadruplex structures are formed by stacking of two or more G-quartets (hereafter referred as quartets), i.e., cyclic planar arrangements of four guanines. Each quartet is stabilized by eight Hoogsteen-type hydrogen bonds involving, for each guanine, the N1 and the exocyclic NH_2_ on C2 as H-bond donors and the O6 and N7 as H-bond acceptors.^10^ Guanine arrangement in a quartet determines the formation of a cavity in the center of the planar structure, delimited by guanine carbonyl oxygens, which represents a specific binding site for metal ions, typically K^+^ or Na^+^. Notably, G-quadruplexes exhibit a remarkable structural polymorphism with respect to duplex DNA, which depends on (i) the number of strands involved in the structure, (ii) the type of linking loops, (iii) the relative strands orientation, (iv) the *syn*/*anti* conformation of the guanine residues, and (v) the nature of the associated cations. In particular, based on the orientation of the four strands, G-quadruplexes are classified in the following topologies: (i) parallel (all parallel strands), (ii) antiparallel (all antiparallel), or (iii) hybrid (three parallel and one antiparallel).^9−11^

G-quadruplex-forming sequences are non-randomly distributed in the genome: in addition to telomeric regions, they are widely localized also at oncogene promoters.^12^

When folded into G-quadruplex structures, telomeres are neither recognized by telomerase (the enzyme responsible for telomere extension) nor involved in homologous recombination processes known as alternative telomere lengthening (ALT), and thus undesired chromosome lengthening cannot occur.^13^ At oncogene level, G-quadruplexes play a key role in transcription regulation: when promoters are folded into G-quadruplex structures, transcription is repressed.^14^ In this perspective, a specific drug inducing G-quadruplex folding at telomeres can block uncontrolled cancer cell growth and proliferation. Furthermore, since oncogenes are overexpressed only in cancer cells,^15^ simultaneously promoting G-quadruplex formation also at oncogene promoters level, using a single drug or a cocktail of drugs, could dramatically reinforce the anticancer activity by exploiting a multi-target approach.

The high potential of a general therapeutic approach based on the use of G-quadruplex ligands as anticancer drugs was strongly corroborated in the past decade. Indeed, the existence of G-quadruplex structures in the genetic material of human cells has been established.^16^ In addition, two G-quadruplex ligands progressed to clinical trials, i.e., Quarfloxin (Phase II for the treatment of neuroendocrine/carcinoid tumors, ClinicalTrials.gov Identifier: NCT00780663(17)) and CX-5461 (Phase I for the treatment of breast cancer with BRCA1/2 deficiency, ClinicalTrials.gov Identifier: NCT02719977(18)). However, in spite of the outstanding advances in this field, no G-quadruplex ligand has been approved as a drug yet, mainly due to bioavailability and/or toxicity issues.

In order to reduce off-target effects and thus overcome toxicity issues, an effective strategy could be to identify G-quadruplex-targeting ligands able to tightly bind genomic G-quadruplexes discriminating the most similar chemical entity present in cells, i.e., duplex DNA, which moreover is more abundant than G-quadruplex DNA in cell.

**Figure 1:**
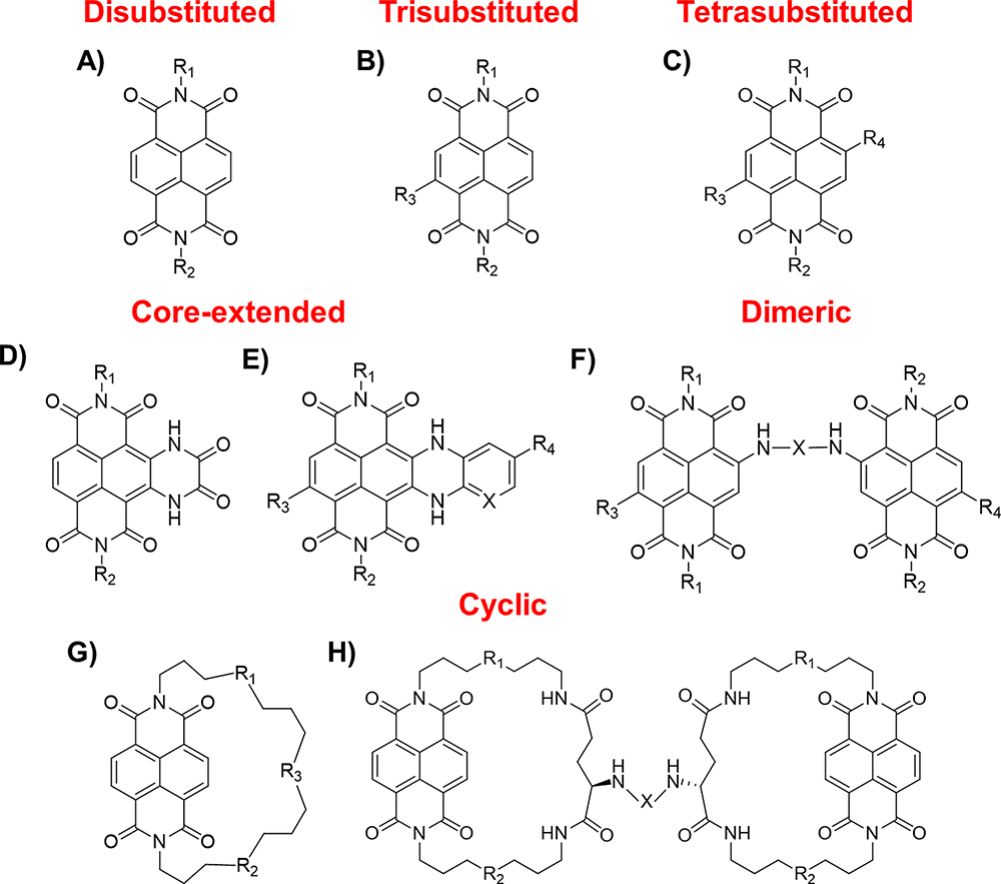
Chemical structures of (A) disubstituted, (B) trisubstituted, (C) tetrasubstituted, (D, E) core-extended, (F) dimeric, and (G, H) cyclic NDIs.

In this frame, among the plethora of investigated G-quadruplex-targeting ligands,^19−21^ we will focus on naphthalene diimides (NDIs), emerged as ones of the most promising compounds due to their good water solubility, permeability, pharmacokinetic, and toxicity profiles.^22,23^ Indeed, their well-proven ability to interact with quartets, as well as their chemical accessibility and the possibility to easily functionalize their aromatic cores with multiple, diverse pendant groups, allow finely modulating their affinity toward different secondary structure-forming oligonucleotides,^24−26^ thus providing a solution to the above-mentioned toxicity issues. Remarkably, it has been shown that the substitution pattern of the NDI core as well as the chemical nature of the substituents play crucial roles in G-quadruplex binding and G-quadruplex over duplex DNA selectivity.^27^ Moreover, the possibility to easily functionalize the NDI core scaffold with chemically diverse moieties represents a high potential solution even to improve their bioavailability. Noteworthy, the high attractiveness of the NDI core scaffold stimulated the synthesis of more than 200 different NDIs in the past two decades (Table S1), thus emerging as the most investigated class of G-quadruplex-targeting compounds with the highest number of structural analogues synthesized thus far. In detail, NDIs can be distinguished in the following six classes: disubstituted, trisubstituted, tetrasubstituted, core-extended, dimeric, and cyclic NDIs (Figure 1 and Table S1). In addition to intrinsic interest for the peculiar chemical properties of these compounds, NDIs proved to be effective potential drugs both in vitro and in vivo, with anticancer activities well correlating with their ability to target genomic G-quadruplexes.^22−27^

On this basis, we reasoned that the time is now ripe to systematically analyze the plethora of biophysical, biological, and structural data acquired throughout the past two decades, aiming at elucidating the structure–activity relationships of this class of compounds in the context of specifically targeting G-quadruplex structures. The interesting correlations here found can be useful to design novel, more active and selective G-quadruplex-targeting compounds as well as further clarify the mechanism of action of NDIs in cancer cells with respect to normal cells.

## Biophysical Studies on NDIs

NDI binding to G-quadruplex structures has been investigated by exploiting several biophysical techniques in a combined approach. Quantitative data of their interactions are summarized in Tables S2–S4. In detail, the ability of NDIs to stabilize the G-quadruplex or duplex target of choice was quantified by calculating the differences between the melting temperatures of each DNA/NDI complex and those of the related free DNA target (Δ*T*_m_), measured by fluorescence resonance energy transfer (FRET), circular dichroism (CD), and/or UV-melting experiments (Table S2). To get a deeper insight into the G-quadruplex over duplex selectivity based on thermal stability data, the Δ*T*_m_(G-quadruplex)/Δ*T*_m_ (duplex) ratios were here calculated starting from the Δ*T*_m_ values reported in the literature (Table S2). From these data, it emerged that the highest stabilizing effects were found for tetrasubstituted **106**, **152**, and **153** interacting with *c-kit2* (**106** and **152**) or *HSP90A* (**153**) G-quadruplex in 50 (**106** and **152**) or 60 (**153**) mM K^+^-containing buffer and at 1:2.5 (**106** and **152**) or 1:5 (**153**) DNA/NDI ratio analyzed by FRET-melting experiments. On the other hand, the most selective NDIs on the basis of the Δ*T*_m_(G-quadruplex)/Δ*T*_m_ (duplex) ratio were found to be tetrasubstituted **139**, **142**, and **150** interacting with *HSP90A* G-quadruplex in 60 mM K^+^-containing buffer at 1:5 (**139** and **142**) or 1:10 (**150**) DNA/NDI ratio analyzed by FRET-melting experiments.

Binding constant (*K*_b_) and stoichiometry values for the interaction between the different NDIs and the target G-quadruplex or duplex were obtained by fluorescence, surface plasmon resonance (SPR), isothermal titration calorimetry (ITC), and UV–vis spectroscopy (Tables S3 and S4). The G-quadruplex over duplex selectivity was evaluated also based on the binding constant values. Starting from the *K*_b_ values reported in the literature, the *K*_b_(G-quadruplex)/*K*_b_ (duplex) ratios were here calculated (Table S3). The highest binding constant values were found for disubstituted **36** and tetrasubstituted **159** and **162** interacting with telomeric (**36** and **159**) or *hTERT* (**159** and **162**) G-quadruplex in 50 (**159** and **162**) or 100 (**36**) mM K^+^-containing buffer analyzed by fluorescence (**159** and **162**) or SPR (**36**) techniques. On the other hand, the most selective NDIs on the basis of the *K*_b_(G-quadruplex)/*K*_b_ (duplex) ratio were found to be core-extended **179** and cyclic **201** and **203** interacting with *c-myc* (**179**) or telomeric (**201** and **203**) G-quadruplex in 100 mM K^+^-containing buffer, as evaluated by fluorescence (**179**) or UV–vis (**201** and **203**) experiments.

In the interaction with G-quadruplex structures, NDIs typically showed binding stoichiometries of 1:1 and 1:2 DNA/NDI (Table S4). This is consistent with the most expected binding mode for this class of compounds, i.e., stacking on one or both the outer quartets. Higher binding stoichiometries, i.e., 1:3 or 1:4 G-quadruplex/NDI, were also observed. This can be explained considering that NDIs can interact also with other regions of a G-quadruplex in addition to the outer quartets, such as grooves and/or loops, and that NDIs can easily self-assemble through stacking interactions, forming dimers which can in turn stack on the outer quartets. Interestingly, an even higher binding stoichiometry, i.e., 1:9 G-quadruplex/NDI, was found for trisubstituted **46** bound to a telomeric dimeric G-quadruplex model, offering multiple binding sites for the NDI molecules, i.e., the G-quadruplex–G-quadruplex interface, the two outer quartets, and the grooves/loops.^28^ On the other hand, binding stoichiometries of 1:1, 1:2, 1:3, and 1:5 were found in complexes formed by duplex structures with NDIs (Table S4). In this case, stoichiometries of 1:1 and 1:2 can be explained considering that one or two NDIs can intercalate into the duplex and/or target one or both of the grooves. Due to the lower number of specific binding sites on the investigated duplex structures with respect to the G-quadruplexes, the highest binding stoichiometries found for the duplex/NDI systems may be correlated with unspecific electrostatic interactions.

Further information on the binding of NDIs to G-quadruplex and duplex structures was obtained by exploiting the above-mentioned or additional experimental techniques.

CD analysis allowed insights into the conformational changes induced by the studied NDI on the target. Increase of the 290 nm band and decrease of the 240 nm band were observed for hybrid monomeric (with disubstituted **2**, **30**, **32**, and **39**; trisubstituted **43**, **46**, **55**, **80**, **83**, **84**, and **93**; tetrasubstituted **159**, **160**, and **162**; core-extended **175** and **179**; dimeric **185**, **186**, **188**, **189**, and **190**; and cyclic **202**, **203**, **209**, and **213**) and dimeric G-quadruplexes (with trisubstituted **46** and cyclic **204**, **214**, **215**, and **216**).^25,26,36−38,28−35^ As far as parallel G-quadruplexes are concerned, a reduction of both the 260 and 240 nm bands was observed (with trisubstituted **46** and **93**; tetrasubstituted **159**, **160**, and **162**; core-extended **165** and **175**; and cyclic **202** and **203**).^25,30,35,37−39^ Conformational changes upon NDI interaction were produced also on duplex structures: increases of both the maximum at 280 nm and the minimum at 250 nm were observed for trisubstituted **46**; dimeric **185**, **186**, **188**, **189**, and **190**; and cyclic **209**.^25,33,36^ Notably, cyclic **200**, **201**, and **202** were studied in their interaction with telomeric and *c-myc* G-quadruplexes under both dilute and molecular crowding conditions by CD.^40^ The studied telomeric G-quadruplex adopted hybrid or parallel topologies in dilute or molecular crowding conditions, respectively, while *c-myc* G-quadruplex folded in parallel topologies in both conditions. Upon addition of the investigated cyclic NDIs, a slight reduction of the CD signals was observed in all cases, with the only exception of the hybrid telomeric G-quadruplex, for which an increase of the band at 290 nm was found. Additionally, strong stabilizing effects were found by CD-melting experiments for all the investigated systems. However, the NDIs stabilizing effect was higher in dilute than in crowding conditions for both G-quadruplexes in the presence of the different NDIs. Interestingly, under crowding conditions without potassium ions, the three NDIs were able to induce the formation of hybrid G-quadruplexes starting from the unfolded telomeric sequence.^40^ Moreover, also trisubstituted **39** and **46** proved to induce hybrid G-quadruplex formation in the absence of cations.^25,29^ In turn, **46** induced antiparallel G-quadruplex formation in the absence of cations starting from the unfolded form of a telomeric DNA sequence, which in K^+^-containing buffers folds in two consecutive hybrid G-quadruplexes.^28,41^

In addition to providing the binding constant and stoichiometry values, ITC allows obtaining the thermodynamic parameters of a specific binding event.^42,43^ In the interaction NDI-G-quadruplexes, enthalpy proved to be the main driving force for the binding of most NDIs (i.e., disubstituted **2**, **30**, **32**, and **39**; and cyclic **200**, **201**, **202**, and **204**). On the other hand, some differences were found in the entropic contribution, mainly due to the different ability of the substituents on the NDI core to displace water molecules on binding the target. A negative entropic contribution was obtained for **30**, **32**, **39**, **200**, **201**, and **202**, suggesting that the resulting G-quadruplex/NDI complexes were less flexible than the free G-quadruplexes.^26,29,40,44^ In contrast, a positive Δ*S* contribution was found for disubstituted **2**, trisubstituted **46**, and cyclic **204**.^26,28,44^ Notably, the entropic factor proved to be the main driving force for the formation of cyclic **205**–**207**/G-quadruplex complexes.^38^

A variant of FRET, i.e., FRET competition experiments, can be very helpful to evaluate the G-quadruplex over duplex or the G-quadruplex over G-quadruplex selectivity.^45^ In these experiments, the affinity for the sequence of interest (DNA G-quadruplex) is evaluated in the presence of increasing concentrations of a competing sequence (duplex or G-quadruplex DNA). The G-quadruplex vs duplex selectivity of the ligands was assessed for disubstituted **1**, **2**, **9**, and **11**; trisubstituted **40**, **41**, **44**, **48**, **50**, **55**, **61**, **62**, **63**, **64**, **65**, **66**, **82**, **87**, **88**, **89**, **90**, and **91**; tetrasubstituted **102**, **103**, **106**, **107**, **109**, **110**, **116**, **117**, **118**, **119**, **123**, **124**, **137**, **145**, **146**, **148**, **152**, **153**, **154**, **160**, and **161**; core-extended **179**; and cyclic **209**.^22,31,33,34,36,37,46−48^ On the other hand, a preference for a telomeric G-quadruplex forming sequence over other DNA G-quadruplexes was well established for cyclic **203**.^30^ Moreover, for trisubstituted **52**, **60**–**66** and tetrasubstituted **116**–**119**, *c-myc* and *bcl-2* G-quadruplexes proved to efficiently compete with telomeric G-quadruplex for binding to the NDIs, whereas *c-kit1* and *c-kit2* G-quadruplexes were less effective competitors.^46,49^

An additional fluorescence-based method useful to determine ligands affinity for G-quadruplexes is the G-quadruplex fluorescent intercalator displacement (G4-FID) assay, in which the ligand binding triggers the displacement of thiazole orange (TO), a fluorescent probe targeting DNA secondary structures. Thus, the affinity of the competing ligand can be assessed by monitoring the changes in TO emission.^50^ When tested by G4-FID assay, core-extended **175** proved to displace TO from telomeric, *c-myc*, and *kRAS* G-quadruplexes, although to different extents.^37^ The displacement was complete upon addition of only two molar equivalents of **175** for the *c-myc* and *kRAS* G-quadruplexes, whereas ca. five equivalents were required for the telomeric G-quadruplex, evidencing a lower affinity for the latter target.^37^

Another assay to evaluate the affinity for G-quadruplex structures and the G-quadruplex vs duplex DNA selectivity is the G-quadruplex on Controlled Pore Glass (G4-CPG) assay.^25,51−56^ This affinity chromatography-based method consists in incubating putative G-quadruplex ligands solutions with CPG solid supports functionalized with different G-quadruplex- and duplex-forming oligonucleotides. The putative ligands are flown through ad hoc synthesized glass supports bearing, covalently attached, the G-quadruplex of interest or the duplex DNA model. The molecules with high affinity for the folded oligonucleotides are retained by the solid supports, while those with low affinity are eluted by a washing solution and quantified through spectrophotometric measurements. The specific interaction of a ligand with the G-quadruplex or duplex structure is further confirmed by inducing its unfolding on the support by a denaturing solution, causing the full release in solution of the bound ligand. According to the G4-CPG assay, trisubstituted **46** was identified as a very promising ligand for G-quadruplex-forming sequences, being endowed with significant G-quadruplex vs duplex DNA selectivity.^25,28^

Studies based on dynamic light scattering (DLS) analysis allowed evaluating the hydrodynamic size of G-quadruplex/NDI complexes in comparison with that of the free G-quadruplex.^41^ These experiments showed that **46** bound a hybrid G-quadruplex forming stable complexes featured by a hydrodynamic diameter significantly increased compared to that of the unbound DNA, indicating the formation of species comprising two or more G-quadruplex units whose interaction was mediated by **46**. On the other hand, when **46** interacted with a parallel G-quadruplex, no relevant change in the DNA hydrodynamic diameter was observed, denoting that the monomeric folding of the parallel model was preserved.^41^

Overall, NDIs showed strong affinity for G-quadruplex structures and displayed in most cases even a high G-quadruplex over duplex selectivity, thus validating their role as appealing G-quadruplex ligands. In addition, their ability to strongly interact with G-quadruplexes of different topologies, located in both telomeres and oncogene promoters, proved that these compounds can act as multi-targeting agents with enhanced anticancer activity. Notably, NDIs are not only able to stabilize G-quadruplex structures, but also to induce G-quadruplex formation, which can be an interesting property for in vivo applications. Stacking interactions with one or both outer quartets of monomeric G-quadruplexes, as well as with both quartets at G-quadruplex-G-quadruplex interface of dimeric G-quadruplexes, appear to be the preferential binding mode. This is well proved by the observed conformational changes of the target, typically obtained upon stacking, and by the fact that the driving force of the binding is mainly enthalpic. However, interactions with grooves and loops are also possible through the substituents on the NDI core, which can involve the displacement of water molecules surrounding the target.

## In Vitro Studies on NDIs

NDIs showed a remarkable anticancer activity in a large number of cancer cell lines. In some studies, these compounds were also tested on normal cells, giving an indication of their overall toxicity. A summary of the in vitro activity against cancer and normal cells is reported in Table S5. Notably, IC_50_ values of the investigated NDIs were significantly lower in cancer over normal cells, proving the highly selective activity on cancer cells of this class of G-quadruplex-targeting compounds. This selectivity on cancer cells was here quantitatively evaluated by calculating the IC_50_(normal cells)/IC_50_(cancer cells) ratios, starting from the IC_50_ values reported in the literature (Table S5). The most active compounds were dimeric **186**, **188**, and **190** on MDA-MB321 (**186**, **188**, and **190**), HT-29 (**186** and **188**), HCT116 (**186** and **188**), and U2OS (**186** and **188**) cell lines, while the most selective compounds proved to be trisubstituted **93** and tetrasubstituted **157** and **158** on MIA-Pa-Ca-2 (**93** and **157**) and PANC-1 (**158**) cell lines.

In addition to the evaluation of the antiproliferative activity against cancer cells, several in vitro assays were performed to investigate more in detail the anticancer properties of NDIs (Table S5).

Different tests were carried out with NDIs to determine possible effects on telomere and telomerase.

For instance, senescence and DNA damage response (DDR) triggered by tetrasubstituted **161** were proved to be consequences of telomere uncapping, while chromosomal instability observed upon treatment with tetrasubstituted **152** was the result of the ligand’s ability to induce telomeric aggregation.^57^

Trisubstituted **44** and **48** and tetrasubstituted **106**, **107**, **145**, **148**, and **152** inhibited telomerase activity, and particularly **48**, **106**, **148**, and **152** showed potent senescence-based short-term antiproliferative effects on different cancer cell lines.^22,58^ Notably, senescence effects were consistent with telomerase activity inhibition, and cytotoxicity on cancer cells of these NDIs was a consequence of telomere targeting and telomerase uncapping.^22,58^

As far as trisubstituted **45**, **46**, and **47** are concerned, their ability to produce DNA damage was evidenced in cancer cells, while no significant DDR was detected in normal cells.^25^ Remarkably, the DNA damage was localized at the telomeric level, as determined by an immunofluorescence-based assay measuring the co-localization spots between γH2AX (marker of DDR) and TRF1 (marker for interphase telomeres).^25^

Interestingly, comparative studies showed that cyclic **201** and **203** were stronger inhibitors of the telomerase activity than their disubstituted acyclic analogues,^30,32,44^ whereas the cyclic dimeric **214** proved to inhibit telomerase activity more efficiently than the cyclic monomeric **204**.^26^

On the other hand, for some NDIs showing anticancer activity (i.e., disubstituted **20** and tetrasubstituted **158** and **159**) and/or able to induce cellular senescence (i.e., tetrasubstituted **139**, **142**, **157**, and **159**), the anticancer activity could not be attributed to effects on telomere and/or telomerase.^59−62^ Indeed, these NDIs were not able to inhibit telomerase activity at the dosage required for inhibition of cellular proliferation. This means that telomere and telomerase are not the unique targets of NDIs in cell, but other mechanisms have to be considered to explain the anticancer activity of these compounds. For instance, cancer cells treated with **159** showed significant dose-dependent modulation of a distinct subset of genes, associated with strong induction of DNA damage responsive genes *CDKN1A*, *DDIT3*, *GADD45A/G*, and *PPM1D*, and repression of genes involved in telomere maintenance, including *hPOT1* and *PARP1*.^61,62^ As concerns the trisubstituted analogue of **159**, i.e., **93**, it produced down-regulation of genes (*PRDM16*, *CBFA2T3*, *TREX1*, *SHANK2*, *TP73*, and *ZNF469*) implicated in human pancreatic cancer, whose promoters are rich in putative G-quadruplex-forming sequences. In addition, up-regulation of genes (*ITGAM*, *ITGA1*, *ST14*, *FLG*, *AVIL*) coding for proteins involved in membrane and extracellular matrix structure and function, including the Suppressor of Tumorigenicity (*ST14*), was observed.^63^ Furthermore, whole-transcriptome RNA-seq analysis of the global gene expression proved that **93** down-regulated a large number of genes rich in putative G-quadruplex-forming sequences and associated with essential pathways of pancreatic ductal adenocarcinoma (PDAC) survival, metastasis, and drug resistance, together with the activation of G-quadruplex replication-dependent DNA damage.^64^ More notably, **93** retained its strong activity in pancreatic cancer cell lines resistant to gemcitabine, a commonly used chemotherapy drug.^65^ A similar ability of down-regulating several cancer gene pathways was observed for a tetrasubstituted derivative of **93**, i.e., **162**.^66^ However, **162** down-regulated a lower number of genes compared to **93**, thus showing a more selective activity than its parent NDI.^66^

Trisubstituted **51** and **52** and tetrasubstituted **113** were evaluated against a panel of genes involved in tumor progression, DNA repair, telomere maintenance, and cell-cycle regulation at both transcriptional and translational levels. In particular, they could inhibit the expression of *hTERT* and *bcl-2* oncogenes.^67^ In addition, **52** was able to interfere with the expression of MYC and KIT oncoproteins in human tumor cell lines of different histological origin and modulation of genes implicated in telomere function and mechanisms of cancer.^68^**52** also decreased the amount of TRF2 and hPOT1 telomeric proteins, induced senescence, reduced telomerase activity,^49^ and down-regulated *RET* oncogene at both mRNA and protein levels.^69^

Tetrasubstituted **145** inhibited telomerase activity, almost fully suppressed expression of the KIT mRNA and protein in a wild-type gastrointestinal tumors (GIST) cell line and down-regulated BCL-2 protein expression in imatinib-resistant GIST cell lines.^47,70^

Core-extended **165** was able to interfere with aberrant androgen receptor (AR) signaling in castration-resistant prostate cancer (CRPC), consistent with its ability to interact with G-quadruplexes within the *AR* gene promoter. Moreover, **165** induced remarkable impairment of AR mRNA and protein amounts and significant perturbations in the expression levels of *KLK3* and genes involved in the activation of *AR* program via feedback mechanisms.^71^ In addition, **165** was able to suppress KIT and BCL-2 protein expression and thus interfere with oncogenic signaling pathways involved in *BRAF* mutant melanoma cell survival, apoptosis, and resistance to drugs.^39^

Dimeric **186** and **188** were able to trigger DDR, but the damage was not limited only at telomeres. Indeed, they targeted also *kRAS* and *c-myc*, whose expression was reduced. However, the reduction of the expression of these oncogenes was not so significant to account for the high toxicity exerted by these NDIs, which is indeed probably caused by DDR.^36^

Finally, fast cell penetration and nucleus/nucleolar localization were demonstrated for several NDIs, even for tetracationic NDIs. The most accredited mechanisms of cell internalization are thought to be passive diffusion or facilitated transport.^22,29,31,57,58,72,73^

In conclusion, several in vitro studies fully demonstrated the high and general anticancer activity of NDIs, able to inhibit at low nanomolar concentration the cell proliferation of breast, ovarian, cervical, prostate, lung, colon, renal, pancreatic, gastric, and gastrointestinal cancers, as well as leukemia, melanoma, osteosarcoma, and glioblastoma. Notably, higher IC_50_ values were found for normal cells with respect to cancer cell lines, strongly corroborating the potential of NDIs in the context of real applications on humans. Overall, the following different in-cell mechanisms can be associated with anticancer activity of NDIs: (i) targeting of telomeres, triggering telomere uncapping, telomere end-to-end fusion, and telomerase activity inhibition and/or (ii) down-regulation of genes rich in putative G-quadruplex-forming sequences, especially oncogenes, involved in tumor onset and progression, DNA repair, telomere maintenance, and cell-cycle regulation. All these findings, along with the ability of NDIs to easily reach the DNA targets into the cells due to their excellent cell internalization, and nuclear localization, make these compounds appealing candidate drugs for novel, effective anticancer therapies.

## In Vivo Studies on NDIs

Tetrasubstituted **153** has been the first NDI investigated for its anticancer activity in vivo by Neidle et al. in 2011, using mouse xenograft models.^74^ Initially, the maximum tolerated dose (MTD) was determined, starting with a 10 μg/kg dose and stepwise increasing it. A safe therapeutic dose, if administered both intraperitoneally (i.p.) and intravenously (i.v.) after dissolution in PBS, was found to be 15 mg/kg given as a single injection. However, even a 3 mg/kg dose injected i.v. three times a week, which was indeed the therapeutic regimen of choice in this study, exhibited a significant antitumor activity. Growth reduction of ca. 50% and 30% relative to controls was observed for MIA-Pa-Ca-2 and HPAC pancreatic tumors, respectively, at the end of treatment (26 and 34 days, respectively). Afterward, analysis of the excised tumor tissues revealed 50% decrease of telomerase activity, associated with 30% reduction of the expression of two proteins involved in telomerase regulation, i.e., HSP90 and hTERT. On the other hand, no significant BCL-2 and kRAS down-regulation was induced by this NDI. Moreover, **153** proved to mainly localize in tumors and pancreas. Only traces of this compound were found in lung, heart, kidneys, liver, and spleen. Notably, neither off-target toxicities nor loss of animal body weight were observed.^74^

Four years later, the same group undertook in vivo studies with a different tetrasubstituted NDI, i.e., **159**.^23,62^ The NDI was dissolved in saline and administered i.v. to evaluate its effects against MIA-Pa-Ca-2 pancreatic cancer xenograft model. In this case, the MTD was 30 mg/kg, while the tested therapeutic doses were 10 and 15 mg/kg, administered twice weekly. Upon 40 days of treatment, ca. 60% and 80% decrease in tumor growth were observed for the two doses, respectively. Remarkably, tumors fully regressed in two mice, which were further observed for additional 239 days. Interestingly, the two animals survived with no tumor regrowth. In addition, down-regulation of both BCL-2 (40%) and kRAS (30%) proteins was observed, while no effect on telomerase activity was detected. **159** mainly localized in cell nuclei and tumors, not being significantly found in other organs. In addition, preliminary pharmacokinetic studies proved that half-life of **159** was 4 h, and no amount of it was detected after 24 h. Moreover, neither vital organ damage nor side effects were produced both during and after the treatment with **159**.^23,62^

Afterward, trisubstituted **52** was tested as anticancer agent in a xenograft model of medullary thyroid cancer (MTC).^69^ The therapeutic regimen was optimized on the basis of the above study on tetrasubstituted **153**.^74^**52** was dissolved in PBS and administered i.p. at 12 mg/kg every 2 days for 12 times. 50% and 37% tumor volume inhibitions were found after 55 and 58 days of treatment for mice xenotransplanted with MZ CRC-1 and TT cells, respectively. Moreover, down-regulation of RET, a protein associated with MTC proliferation, was detected in vivo. Differently from **153** and **159**, treatment with **52** resulted in a slight (<15%) body weight loss. However, no general toxicity was observed.^69^

Successively, trisubstituted **93** was evaluated for its antitumor activity in PDAC animal models.^64,75^ It was tested in comparison with tetrasubstituted **159** and gemcitabine. The MTD for **93** was determined to be 45–50 mg/kg, which is twice as high as the one of **159**. The study was carried out administering i.v. doses of 10 mg/kg for **93** and/or 15 mg/kg for **93**, **159**, and gemcitabine, all dissolved in saline, twice weekly for 28 days. Tumor growth inhibition was observed for all the compounds even after 62 days from the beginning of the treatment, with similar decreases in tumor volume for **93** administered at 10 mg/kg (52%) and **159** and gemcitabine at 15 mg/kg (57% and 62%, respectively). Notably, **93** produced higher reduction of the tumor volume (85%) than **159** and gemcitabine when injected at the same dose (15 mg/kg). In addition, after the end of the treatment with **93** (from day 28 to 62), no tumor regrowth was found, contrarily to what observed upon treatment with **159** and gemcitabine. No body weight loss or side effects were detected for **93**. Moreover, this NDI mainly accumulated in the tumor mass. Interestingly, **93** was tested also on KPC mouse model, a better model of pancreatic cancer, typically used in advanced steps of preclinical studies, which is resistant to chemotherapy. Analogously to gemcitabine,^76^ even if i.p. injection of **93** at 15 mg/kg showed no significant effect relative to the controls, a longer survival was observed in treated than untreated mice.^64^

More recently, trisubstituted **43** and **85** were tested on HT-29 colon cancer xenograft mice model.^63^ Both compounds were injected i.p. three times a week for 14 days. Doses of 19 and 39 mg/kg were used for **85**, while a reduced dose (11 mg/kg) was used for **43** due to toxicity issues. **43** showed no significant difference with respect to controls, while dose-dependent effects were observed for **85** with a 35% decrease of tumor volume at 39 mg/kg after 12 days of treatment. Weight loss and adverse effects were not observed during the treatment. Only after 27 days a slight reduction of weight was observed in mice treated with **85** at 39 mg/kg. Tissues were not damaged by the treatment, even if treated mice showed inflamed liver, whereas no inflammation was found in the controls. **43** and **85** mainly accumulated in the proximity of the tumor but not inside it.^63^

The most recent in vivo study on NDIs was performed by Neidle et al. on tetrasubstituted **162** in comparison with the previously investigated trisubstituted **93** and gemcitabine.^66^ A MIA-Pa-Ca-2 xenograft model was used, and mice were treated with **162** at 1 mg/kg dose (dissolved in PBS) once or twice a week, while **93** and gemcitabine were respectively administered at doses of 10 and 15 mg/kg (in PBS) or 15 mg/kg (in saline) twice a week. A significant antitumor effect was detected for **93** and gemcitabine at both doses after 28 days of treatment. However, from day 28 to 53 some tumor regrowth was observed. On the other hand, the highest tumor volume reduction was found for **162** with no tumor regrowth after day 28. Notably, several animals showed complete tumor regression when treated with **162**. Moreover, there was no evidence of cardiac or neurological effects. In addition, KPC mice were also treated with **162** once a week at 1 mg/kg dose. Noteworthy, four mice out of six survived for more than 20 days.^66^

Although all the in vivo tested NDIs showed significant anticancer activity, excluding **43**, the most attractive compounds appeared to be **93**, **153**, **159**, and **162** due to their strong activity accompanied by absence of toxicity and of body weight loss (Table S6). Among them, trisubstituted **93** and tetrasubstituted **162** showed higher effects on tumor growth than tetrasubstituted **153** and **159**. Overall, **162** emerged as the best putative anticancer drug among the tested compounds, showing the highest percentage of tumor volume inhibition and the lowest effective dose.

## Crystallography, Molecular Modeling, and NMR Studies on NDIs

X-ray crystallography and/or molecular modeling studies allowed a deep insight into the structural details of the complexes formed between NDIs and G-quadruplex structures (Table S7).

The first G-quadruplex/NDI crystal structure was solved by Parkinson et al. in 2008.^77^ In detail, the tetrasubstituted **109** was co-crystallized with the parallel telomeric G-quadruplex of the sequence d[TAGGG(TTAGGG)_3_] (23-mer). Notably, **109** was able to mediate the interaction between two G-quadruplexes, thus inducing the formation of a 2:6 G-quadruplex/NDI complex where the two G-quadruplexes were 5′-5′ stacked, with two NDI molecules sandwiched between the two G-quadruplexes, two additional NDIs stacked each on a 3′-end quartet, and the latter NDIs bound to two different G-quadruplex loops. All the four stacked NDIs, on both the 5′- and 3′-end quartets, were involved in interactions with all guanines forming the specific quartet, due to the central location of the NDIs with respect to each quartet. However, due to both the central location on the quartets and the short length of the substituents, the two *N,N*-dimethylamino and two hydroxyl groups of **109** were not involved in hydrogen bonds with the DNA phosphate backbone. In turn, these contacts were mediated by water molecules or, in other cases, these substituents even pointed away from the G-quadruplex grooves and backbone. Interestingly, despite the parallel folding of both G-quadruplexes was preserved, NDIs induced rearrangements of both loop and 5′-end flanking nucleobases, thus promoting the formation of additional binding surfaces for stacking interactions. Indeed, the two loop-bound NDIs were well-sandwiched between two different A-T base-pair associations formed, in one case, by bases of the same loop and, on the other hand, by a space-close 5′-end thymine of one G-quadruplex and an adenine in the loop of the other G-quadruplex involved in the complex. Notably, contrarily to quartet-bound NDIs, loop-bound NDIs were involved in some direct hydrogen bonds with the DNA.^77^

Successively, tetrasubstituted **153** and **160** were studied in their interactions with a parallel telomeric G-quadruplex of the sequence d[AGGG(TTAGGG)_3_] (22-mer).^78^ 2:2 G-quadruplex/NDI complexes were formed where both NDIs preferentially interacted with the 3′-end quartets and the two G-quadruplexes were bound via 5′-5′ stacking. Interestingly, stacking between the two G-quadruplexes was mediated by a coordinating potassium ion, and the parallel folding of both G-quadruplexes was conserved. As concerns **153**, this ligand was asymmetrically located on the quartet, with two of its four substituents deeply inserted into the G-quadruplex grooves, the third close to the groove, and the latter pointing away from the DNA. Interactions with the grooves involved direct or water-mediated hydrogen bonds, as well as electrostatic interactions between the positively charged *N-*methylpiperazine nitrogen atoms of **153** and DNA negatively charged phosphate groups. On the contrary, **160** was positioned in the center of the quartet, with all four substituents located each in one of the four G-quadruplex grooves, and particularly three of them deeply inserted in the grooves, stabilized by weak hydrogen bonds and/or electrostatic interactions. Overall, **153** produced stronger interactions with the G-quadruplexes than **160**, which, associated with the higher mobility of both the core and the substituents of **160**, resulted in higher affinity and more specific binding for **153** than **160**.^78^

More recently, the crystal structure of the complex of the tetrasubstituted **159** with the 22-mer parallel telomeric G-quadruplex was solved.^61^**159** was designed starting from **153** and then replacing two of the four *N-*methylpiperazino groups with two morpholino moieties. As for **153**, 2:2 G-quadruplex/NDI complexes were formed with two 5′-5′ stacked G-quadruplexes and **159** located at both the 3′-end quartets. Three of the four substituents of **159** were placed in the grooves, with one morpholino group deeply inserted, while the latter morpholine pointed away from the near groove. Hydrogen bonds and electrostatic interactions of the *N-*methylpiperazino group well-inserted in the groove were direct, while the interactions of the morpholino group pointing into the groove were all mediated by water molecules.^61^

A higher number of structures of G-quadruplex/NDI complexes have been analyzed by molecular modeling studies than by crystallographic investigations. Molecular modeling was used mainly to design novel functionalized NDIs, validate potential binding modes, and support the crystallographic data. In all the here described studies, 1:1 G-quadruplex/NDI models were analyzed, unless otherwise stated.

A detailed molecular dynamics (MD) simulation was performed with tetrasubstituted **109** starting from the 2:6 G-quadruplex/NDI crystallographic model previously solved with the 23-mer parallel telomeric G-quadruplex.^79^ In detail, the analysis was carried out building four different models based on the 2:6 DNA/NDI X-ray structure: (1) the native complex, (2) a model containing one G-quadruplex and four NDIs bound to two quartets and two loops, (3) a sub-model of model 2 where only the two quartet-bound NDIs were considered, and (4) a different sub-model of model 2 where only the two loop-bound NDIs were considered. Using these four models as starting points for MD simulations, it was proved that (i) binding to the loops is possible for NDIs also in aqueous solution and is not a mere consequence of crystal packing effects; (ii) binding to the loops enhances G-quadruplex flexibility, while binding simultaneously to both loops and quartets results in a more rigid G-quadruplex; (iii) binding to quartets provides more stable and less dynamic complexes than binding to the loops; (iv) in-plane motions are possible for NDIs stacked on the quartets, while both in-plane and up-and-down motions can be observed for NDIs in the grooves.^79^

For tetrasubstituted **153** and **160**, docking was performed with the same 22-mer telomeric sequence used in crystallographic studies, forming a parallel G-quadruplex. By placing the NDIs on the 3′-end quartet, similar binding modes were observed with respect to the crystallographic models.^48,80^ However, slight differences in the location/distance of **153** and **160** onto/from the 3′-end quartet and of their substituents in the grooves were found comparing the molecular modeling binding poses with crystallographic data.^48,80,81^ These discrepancies can be explained considering both the ligand mobility, giving rise to a certain degree of uncertainty of the real ligand position, and the crystal packing effects.^80,81^ Moreover, an additional modeling study on **153** and **160** proved that, when the terminal nitrogen atoms of *N-*methylpiperazino groups are protonated, the interaction with the G-quadruplex is stronger for both compounds than when the internal nitrogen atoms of *N-*methylpiperazine are protonated. Indeed, in the case of terminal nitrogen protonation, two direct electrostatic interactions and two water-mediated hydrogen bonds, involving the protonated nitrogen atoms, were formed with DNA, while, in the case of internal protonation, only indirect hydrogen bonds could be formed.^81^

As concerns tetrasubstituted **159**, MD studies were performed using as the target the parallel telomeric G-quadruplex of the sequence d[GGG(TTAGGG)_3_] (21-mer),^60^ lacking the 5′-end nucleotide with respect to the target used in the above-described crystallographic study. In addition, the positional isomer of **159**, i.e., tetrasubstituted **158**, was also investigated, obtained by interchanging the morpholino and *N-*methylpiperazino side chains. MD showed that **159** slightly changed during the 50 ns run with one morpholine positioning closer to the loops with respect to the starting crystal structure. On the other hand, **158** moved by 90° from its starting position, so that in the final pose its morpholino and *N-*methylpiperazino groups were positioned in the same grooves as the ones respectively preferred by morpholine and *N-*methylpiperazine of **159**. However, the substituents of **158** were differently positioned into each groove in comparison to the ones of **159**, due to the different lengths of the chains bearing morpholino and *N-*methylpiperazino moieties in **158** with respect to **159**.^60^

Moreover, **159** was also studied in its interaction with the parallel G-quadruplex of *bcl-2* promoter of sequence d(GGGCGCGGAGGAAGGGGGCGGG) (22-mer).^23^ In this case, MD simulation was needed due to the high mobility of the *bcl-2* loop close to the quartet where **159** was expected to bind. At the end of the MD run, **159** was sandwiched between the 5′-end quartet and G12 residue of the flexible loop, with its substituents interacting with the loop.^23^

In addition, docking studies on **159** in its interaction with the 22-mer parallel telomeric G-quadruplex were in good agreement with crystallographic data.^64^ Binding to 3′-end quartet was preferred, and the four substituents were close to the four grooves, with one morpholino and one *N-*methylpiperazino residue deeply inserted into the grooves. Considering that both in crystallographic and docking structures the contribution to the binding of the fourth substituent was marginal, a suitable trisubstituted NDI, i.e., **93**, was designed.^64^**93**, derivatized with two morpholino groups and one pyrrolidino group, was asymmetrically stacked on the 3′-end quartet, and its three substituents were close to three different grooves, with one of the two morpholino rings well-inserted into the groove. Notably, similar binding poses were found for both **159** and **93** when docked with a parallel G-quadruplex of *hTERT* promoter of the sequence d(AIGGGAGGGICTGGGAGGGC) (20-mer).^35^ In addition, **162**, a tetrasubstituted analogue of **93**, was also docked with both *hTERT* G-quadruplex and a hybrid-1 telomeric G-quadruplex model of the sequence d[TTGGG(TTAGGG)_3_A] (24-mer). In both cases, all the substituents of **162** pointed away from the grooves and, with the hybrid G-quadruplex, even the NDI overlap on the quartet was partly limited by the TTA lateral loop.^35^

Two NDIs bearing four substituents, all with pyrrolidine rings and differing only in the length of the side chains, i.e., tetrasubstituted **145** and **152**, were also studied by docking and molecular dynamics in their interaction with the 23- and 22-mer parallel telomeric G-quadruplexes, respectively.^22,47^ Even if **145** and **152** were docked exploring only the 3′- or 5′-end surfaces, respectively, similar binding poses were observed, where the NDIs stacked on the center of the specific quartet. However, due to their longer side chains, the substituents of **152** were closer to the grooves, even if not deeply inserted, than in the case of **145**.^22,47^

As the last described modeling study on tetrasubstituted NDIs, we report on the case of **116**, **117**, and **118**.^46^ The target of choice was in this case the 21-mer parallel telomeric G-quadruplex. All the compounds were stacked onto the 3′-end quartet. The substituents of **116**, **117**, and **118** interacted by electrostatic and/or hydrogen bonds with the DNA phosphate backbone. Moreover, while **116** and **117** were similarly located on the quartet and interacted with the same or close residues, **118** was approximately 90° rotated with respect to them, forming interactions with different residues.^46^

As far as dimeric NDIs are concerned, molecular modeling studies were undertaken for compounds **2**, **18**, **20**, **23**, **30**-**34**, **38**, and **39**.

**33** and **34** interacted with the 22-mer parallel telomeric G-quadruplex at its 3′-end quartet by stacking and electrostatic interactions between the alkylpyridinium rings and the negatively charged DNA phosphate groups.^80^

**18**, **20**, and **23** were studied in their interaction with telomeric G-quadruplexes of the sequences d[TAGGG(TTAGGG)_3_] (23-mer) and d[AGGG(TTAGGG)_3_T] (23-mer), adopting hybrid-1 and hybrid-2 topologies, respectively.^59^ Moreover, the interaction with the bimolecular B-duplex structure, obtained by hybridization of the sequence d(GGATGTGAGTGTGAGTGTGAGGG) (23-mer) with its complementary strand d(CCCTCACACTCACACTCACATCC), containing an intercalative binding site, was also investigated. In this study, 1:2 and 1:1 binding stoichiometries were considered for the complexes G-quadruplex/NDI and duplex/NDI, respectively. All the compounds interacted with both hybrid-1 and hybrid-2 at the grooves by forming hydrogen bonds, electrostatic interactions, and T-shaped π–π stacking. However, a higher number of electrostatic interactions were found with hybrid-1, which appeared to be the preferred target with respect to hybrid-2. Among the tested compounds, **18** proved to be the worst ligand. Indeed, differently from **20** and **23**, this compound had only one protonated nitrogen atom and, not being decorated by polyamine chains, formed a lower number of hydrogen bonds and electrostatic interactions. Notably, the observed binding to the G-quadruplex grooves can be explained considering that rigid G-quadruplex models were used in the docking procedure, featured by the presence of terminal nucleotides which, covering the quartets, impeded the binding of the ligands in those regions. Surprisingly, despite the presence of an intercalative binding site, groove binding of these NDIs was observed also with the duplex structure. However, a lower number of hydrogen bonds and electrostatic interactions were found in this case, proving a good G-quadruplex over duplex selectivity.^59^

**2**, **30**, **31**, **32**, **38**, and **39** were docked with the 22-mer parallel telomeric G-quadruplex.^29^ All of them stacked onto the 3′-end quartet, having one substituent deeply inserted into a groove, while the other one pointed away from the G-quadruplex. Interactions with the groove involved hydrogen bonds and/or electrostatic attractions between protonated nitrogen atoms of imidazole, *N-*methylpiperazino, or *N,N-*dimethylamino substituents and the DNA.

Additionally, molecular modeling approaches were exploited to investigate the binding mode of cyclic NDIs to G-quadruplexes as well as duplex structures. In detail, molecular dynamics investigations were carried out with **201**, **203**, **205**, and **212** using the 24-mer hybrid-1 telomeric G-quadruplex as the target.^30,38,44^**201**, **203**, and **212** were accommodated close to the 5′-end, and T1, T2, T18, T19, and A20 residues, covering the upper quartet in the free G-quadruplex model, were maintained flexible during the modeling process.^30,44^ On the other hand, also T12, T13, A14, and A24, covering the lower quartet, were left as flexible residues during the simulation with **205**.^38^ Therefore, 1:1 DNA/NDI complexes were obtained for **201**, **203**, and **212**, while, due to similar accessibility of both quartets, 1:2 complexes were formed with **205**. In all cases, cyclic NDIs bound to the target G-quadruplex by stacking of the naphthalene diimide core onto the quartets. Moreover, due to their shorter linker chains, **201**, **203**, and **205** were more hidden than **212** in the binding pocket formed by loop and flanking end residues close to the binding-involved quartet.^30,38,44^

As concerns cyclic NDIs **199**, **208**, **209**, **210**, and **211**, docking and molecular dynamics simulations were performed with the bimolecular parallel telomeric G-quadruplex of the sequence d(TAGGGTTAGGGT) (12-mer).^33^ The bimolecular B-duplex model of sequence d(CGATCG) (6-mer), containing an intercalative binding site, was also used as a control in this study. Remarkably, grooves proved to be the preferred binding sites for **199**, **208**, and **209** when interacting with both models, even though quartets (for the G-quadruplex) and intercalative site (for the duplex) were fully accessible. As regards **210** and **211**, these compounds interacted with the upper quartet of the G-quadruplex and with the grooves of the duplex. Electrostatic interactions were the driving force in the binding to the G-quadruplex for **208**, **209**, and **210**, while van der Waals interactions were prevailing in the binding to the G-quadruplex of **199** and **211** and to the duplex for all the five investigated cyclic NDIs. The strongest interactions were found for **199**, **208**, **209**, and **210**, and, among them, **199**, having the shortest linker and only two protonated aliphatic amino groups (vs four found for **208**, **209**, and **210**), was the worst ligand. Notably, the average Total Interaction Energy of **199**, **208**, **209**, and **210** showed a good G-quadruplex vs duplex selectivity, while **211** exhibited preference for the duplex.^33^

As far as the interaction NDIs/dimeric G-quadruplexes is concerned, trisubstituted **46** and dimeric **182** were in parallel studied by molecular docking in their binding to the hybrid dimeric telomeric G-quadruplex of sequence d[GGG(TTAGGG)_7_] (45-mer).^28^ Interestingly, both NDIs were able to target the G-quadruplex-G-quadruplex interface. However, **46** was better accommodated into the pocket between the two G-quadruplexes compared to **182**, whose second NDI unit was located outside the target. Overall, a worse fit of the dimeric vs monomeric ligand to the target was observed, thus indicating that trisubstituted **46** was a better ligand for the dimeric G-quadruplex than dimeric **182**.^28^

Despite the wide interest in NDIs as G-quadruplex ligands, only one in-depth NMR study on the binding to G-quadruplexes of a naphthalene diimide, i.e., trisubstituted **46**, has been reported thus far.^41^ In detail, **46** was studied in its interaction with parallel and hybrid G-quadruplex models. Interplay of different binding modes of **46** to G-quadruplexes was observed for both parallel and hybrid topologies, with end-stacking always operative as the predominant binding event. While **46** primarily targeted the 5′-end quartet of the hybrid G-quadruplex, the binding to the parallel G-quadruplex occurred simultaneously at the 5′- and 3′-end quartets. Notably, G-quadruplex loops containing two nucleobases proved to mediate the interaction between **46** and the G-quadruplex structures, and their role was notable in the context of interdependence of secondary binding events.^41^

Overall, all the different classes of NDIs investigated by crystallography, molecular modeling, and NMR, i.e., di-, tri-, and tetrasubstituted, dimeric, and cyclic NDIs, share similar binding modes to G-quadruplexes, regardless of their topology (parallel, hybrid-1, or hybrid-2), consistent with the ability of the naphthalene diimide core to strongly stack on the outer or interfacial quartets. Binding stoichiometry of 1:1 G-quadruplex/NDI is the most commonly observed, but also stoichiometries of 1:2 (for disubstituted **18**, **20**, **23**, and cyclic **205**) and 1:3 (for tetrasubstituted **109**) proved to be possible. Notably, a general preference for the 3′-end quartet was observed, although some crystallographic, molecular modeling, and NMR studies showed potential and stable interaction also with the 5′-end quartet. This is probably due to the higher accessibility of the 3′-end with respect to the 5′-end quartet in the analyzed G-quadruplex models, where flanking nucleobases were always present at the 5′-end and not always at the 3′-end and/or were more abundant at the 5′-end than at the 3′-end. Therefore, rearrangement of the 5′-end residues seems to be less favorable than at the 3′-end, when the ligand targeting the G-quadruplex structure is an NDI. As an alternative explanation, due to the absence or very limited presence of flanking residues, the 3′-end rearrangement could even not be necessary to allow NDI binding to this outer quartet, while in the case of the 5′-end the rearrangement could always be required. On the other hand, groove binding was observed in a few cases both by crystallography and molecular modeling, however, proving that interactions with the grooves are possible both in solid state and in solution. Interestingly, groove binding was found for the tetrasubstituted **109** and the cyclic **199**, **208**, and **209** when interacting with parallel G-quadruplexes having fully accessible quartets. This probably occurred because all these compounds are able to provide stacking interactions with loop nucleobases and/or a high number and/or strong electrostatic interactions and hydrogen bonds with the loops, which makes groove binding more energetically favorable than binding to quartets. Groove binding was operative also for disubstituted **18**, **20**, and **23** when interacting with hybrid-1 and hybrid-2 G-quadruplex topologies, even if in this case flanking end and loop residues impeded the binding to quartets. Therefore, in our opinion, this study cannot be considered conclusive about the real binding mode of these compounds. On the contrary, binding to the grooves was always observed when the target was a duplex, even when the duplex contained an intercalative binding site. Overall, a good G-quadruplex vs duplex selectivity was proved, which could be explained by a higher number of interactions and thus higher binding energies for G-quadruplex than duplex structures, as revealed by molecular modeling studies.

Due to the smaller surface of the NDI core with respect to the quartets, an NDI can simultaneously interact with the four guanines involved in the quartet but is not able to cover the whole quartet surface. Thus, two possible planar orientations onto the quartet were observed: symmetrical and asymmetrical. The first is preferred by tetrasubstituted NDIs, while the second by di- and trisubstituted NDIs. As concerns the disubstituted NDIs, typically, one substituent is closer to a groove than the second one, and in some cases even inserted into it, which is not able to interact with the opposite groove and sometimes even points away from the G-quadruplex. This is a consequence of the asymmetrical stacking of the NDI core on the quartet, which maximizes the interaction of one substituent with a groove, in turn fully impeding the binding of the second substituent to the opposite groove. On the contrary, tetrasubstituted NDIs are located in the center of the quartet, and all their four substituents could, in principle, interact with all four grooves. However, none of these substituents is very close to the grooves, and their interaction seems to be averaged, and even the third and more often the fourth substituent are not inserted into the grooves or point away from the G-quadruplex. Notably, trisubstituted NDIs seem to be better ligands than the tetrasubstituted ones. Indeed, after removing the fourth substituent, the NDI core tends to asymmetrically locate onto the quartet and the three substituents benefit from this binding pose, thus interacting with three different grooves, stronger than in the case of the substituents of tetrasubstituted NDIs. As concerns cyclic NDIs, the central or lateral location with respect to the quartet is less relevant. Indeed, due to the cyclic structures, their chains cannot point toward the grooves but are rather directed away from the grooves and can interact only with the loops of the G-quadruplex. Indeed, in some cases, loops are able to rearrange upon NDI interaction to maximize the contact surface with the cyclic NDIs and generate a peculiar binding pocket for the cyclic ligand. On the other hand, cyclic NDIs can also be located at the grooves.

Altogether the NDI core position with respect to the quartet, as well as the length of the NDI side chains and the functional groups on their substituents, play relevant roles in the binding mode to G-quadruplexes and stability of the resulting G-quadruplex/NDI complexes. In general, electrostatic interactions and direct or water-mediated hydrogen bonds are the most common interactions of the substituents with the grooves/loops, even though T-shaped π–π stacking as well as face-to-face stacking interactions were also observed.

Finally, binding of NDIs is dynamic on the nanosecond time scale: in-plane and rotational motions are possible for NDIs stacked on the quartet, while both in-plane and up-and-down motions can be observed for NDIs in the grooves. However, binding to quartets is more stable and less dynamic than binding to the loops.

## Analysis of Biophysical Properties and In Vitro/In Vivo Activity of NDIs

In this section, all the above-described biophysical properties of NDIs able to interact with G-quadruplex/duplex structures, along with their in vitro/in vivo activity on cancer/normal cells, have been analyzed and compared based on the class of each NDI (di-, tri-, and tetrasubstituted, core-extended, dimeric, and cyclic) and the different experimental conditions and techniques exploited.

**Figure 2:**
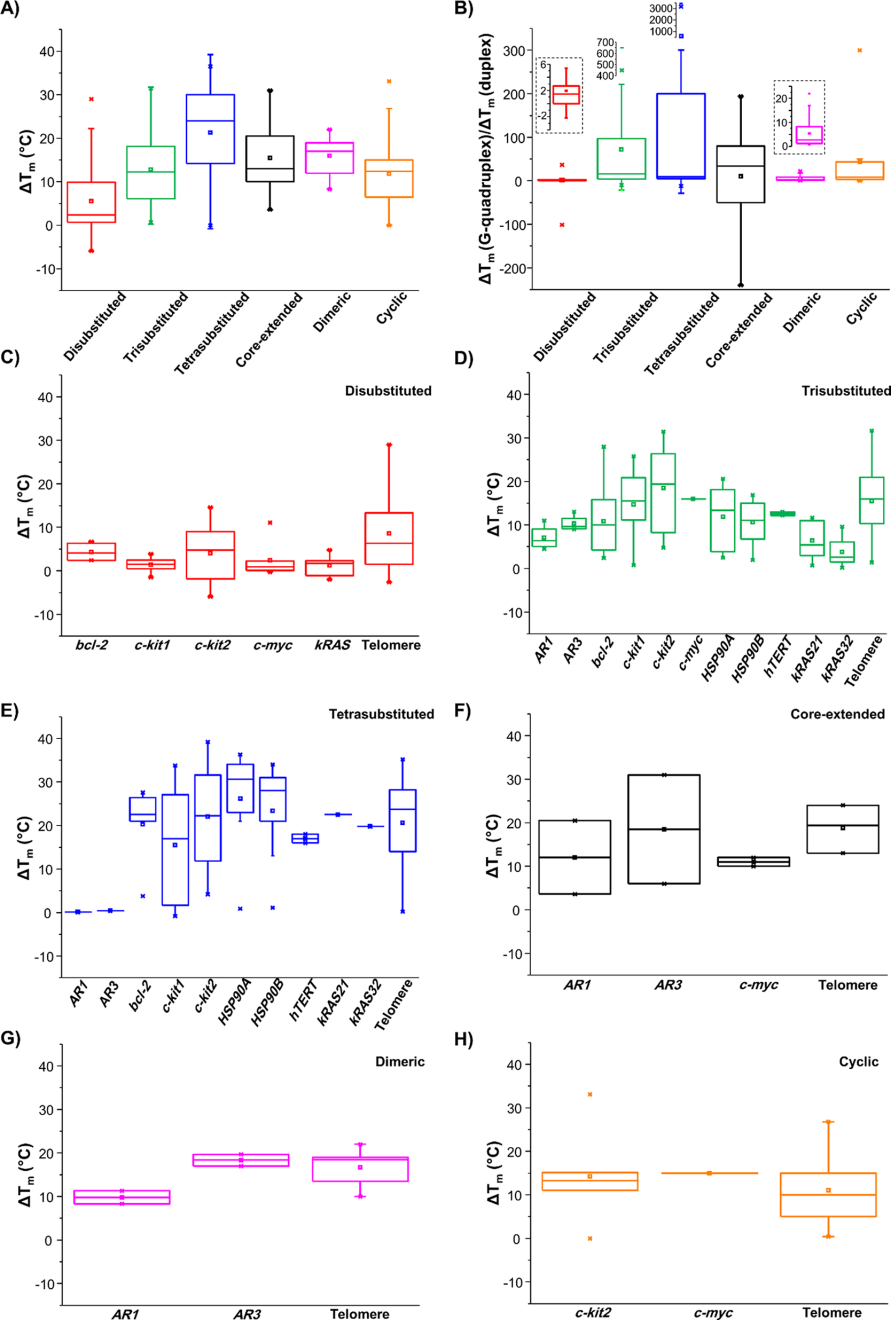
(A) Δ*T*_m_ values for the six classes of NDIs interacting with G-quadruplex structures. (B) Δ*T*_m_(G-quadruplex)/Δ*T*_m_(duplex) ratios for the six classes of NDIs. Insets: enlargements of the bars related to disubstituted and dimeric NDIs. (C–H) Δ*T*_m_ values for di-, tri-, and tetrasubstituted, core-extended, dimeric, and cyclic NDIs, respectively, interacting with G-quadruplex structures originating from the indicated genomic regions (gene promoters or telomeres).

Comparison of Δ*T*_m_ values for the six classes of NDIs interacting with G-quadruplexes provided the following insights. In detail, it emerged that the trend of NDIs’ stabilizing ability on G-quadruplexes is as follows: tetrasubstituted > dimeric > core-extended > trisubstituted > cyclic > disubstituted (Figure 2A). Moreover, destabilizing effects were found for some disubstituted NDIs. As far as the G-quadruplex vs duplex selectivity is concerned, as derived from Δ*T*_m_(G-quadruplex)/Δ*T*_m_(duplex) ratios, the following trend was found: tetrasubstituted > core-extended > trisubstituted > cyclic > dimeric > disubstituted (Figure 2B). On the basis of Δ*T*_m_ values (G-quadruplex) and Δ*T*_m_(G-quadruplex)/Δ*T*_m_(duplex) ratios, it seems that the most selective stabilizers of G-quadruplex structures are the tetrasubstituted, core-extended, and trisubstituted NDIs. However, both the above trends should be taken only as rough estimates of the real behavior of different NDIs, since these ligands were tested in their ability to stabilize different G-quadruplexes at different G-quadruplex/NDI ratios, in different buffers, and by exploiting different techniques. Indeed, as reported below, the *T*_m_ values are strictly dependent on all these experimental conditions.

However, regardless of the exploited techniques and experimental conditions, it clearly appears that, with the only exception of disubstituted NDIs/G-quadruplex systems along with trisubstituted NDIs/*AR1*, *kRAS21*, *kRAS32* G-quadruplexes, and tetrasubstituted NDIs/*AR1*, *AR3* G-quadruplexes systems, Δ*T*_m_ values ≥10 °C were observed in all cases. These findings denote high stabilizing ability of NDIs on all the investigated G-quadruplex-forming sequences, as well as no marked preference of NDIs for a specific G-quadruplex structural topology (Figure 2C–H).

**Figure 3:**
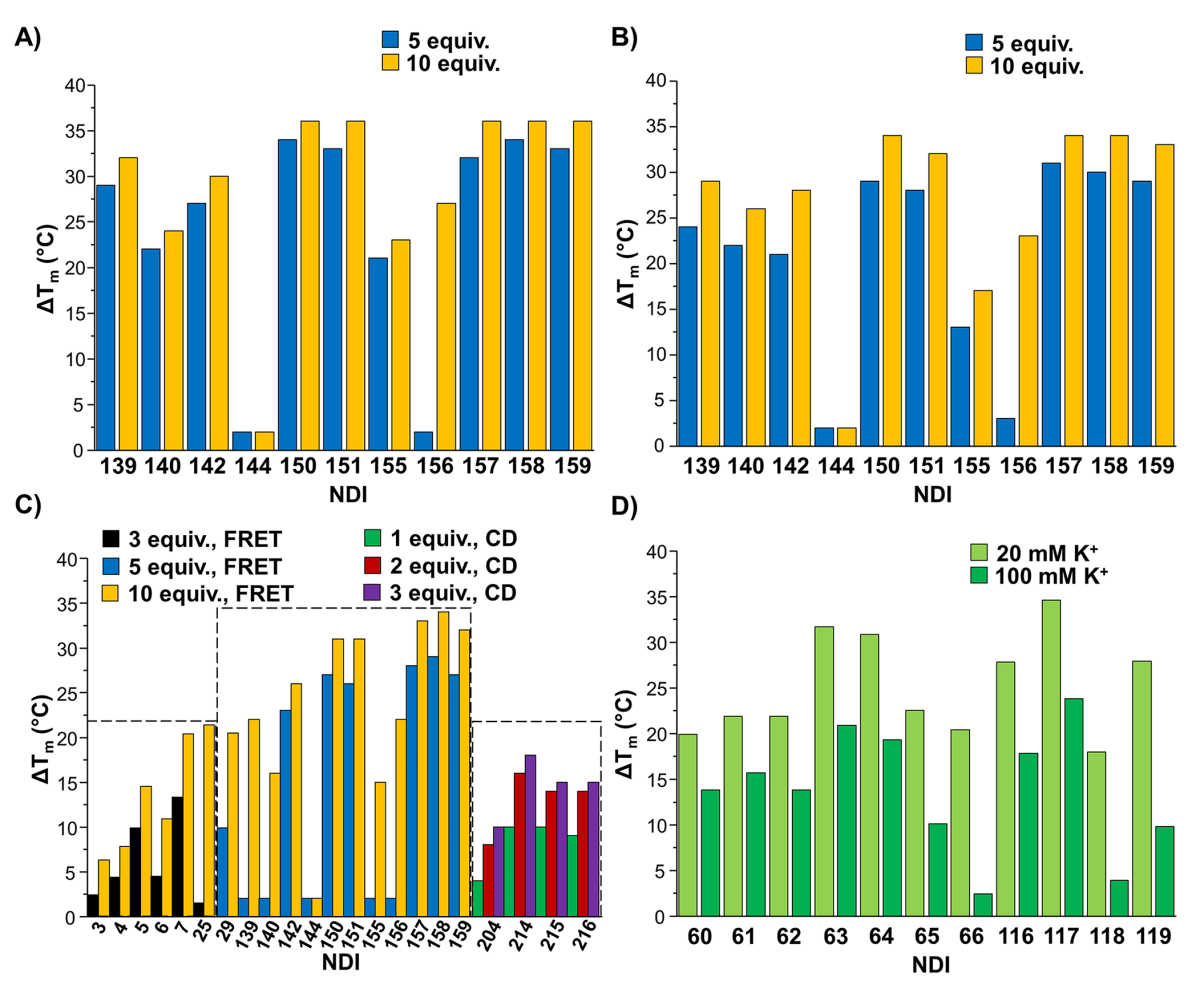
(A) Δ*T*_m_ values for the indicated NDIs interacting with *HSP90A* G-quadruplex at 1:5 or 1:10 G-quadruplex/NDI ratio evaluated by FRET-melting experiments. (B) Δ*T*_m_ values for the indicated NDIs interacting with *HSP90B* G-quadruplex at 1:5 or 1:10 G-quadruplex/NDI ratio evaluated by FRET-melting experiments. (C) Δ*T*_m_ values for the indicated NDIs interacting with telomeric G-quadruplexes at 1:3, 1:5, or 1:10 G-quadruplex/NDI ratio evaluated by FRET-melting experiments and at 1:1, 1:2, and 1:3 G-quadruplex/NDI ratio as evaluated by CD-melting experiments. (D) Δ*T*_m_ values for the indicated NDIs interacting with telomeric G-quadruplexes at 1:4 G-quadruplex/NDI ratio and in 20 or 100 mM K^+^-containing buffer as evaluated by FRET-melting experiments.

The dependence of Δ*T*_m_ on the experimental conditions was evaluated by careful analysis of the data available in the literature, as reported below. In detail, by comparing the differences in the Δ*T*_m_ values (ΔΔ*T*_m_) obtained by FRET-melting experiments for *HSP90A* G-quadruplex when incubated with 10 or 5 equiv of different NDIs, it emerged that the average ΔΔ*T*_m_ value was +3 °C (Figure 3A). A similar behavior was observed for *HSP90B* G-quadruplex with an average ΔΔ*T*_m_ value of +4 °C (Figure 3B). On the other hand, comparing the ΔΔ*T*_m_ values calculated from FRET-melting experiments for a telomeric G-quadruplex when incubated with 10 or 3 equiv of different NDIs, an average ΔΔ*T*_m_ value of +8 °C was obtained (Figure 3C). Conversely, an average ΔΔ*T*_m_ value of +9 °C was obtained comparing FRET-melting data for a different telomeric G-quadruplex model when incubated with 10 or 5 equiv of different NDIs (Figure 3C). In addition, calculating the ΔΔ*T*_m_ values from CD-melting experiments for a telomeric G-quadruplex incubated with 1, 2, or 3 equiv of different NDIs, average ΔΔ*T*_m_ values of +4 or +2 °C were determined, considering the differences between the 1:2 and 1:1 G-quadruplex/NDI ratio systems or the 1:3 and 1:2 G-quadruplex/NDI ratio systems, respectively (Figure 3C).

Altogether the above findings evidenced, as partly expected, that the G-quadruplex stabilization increases on increasing the NDI molar equivalents. However, the target stabilization does not linearly increase as a function of the NDI molar equivalents, but a hyperbolic behavior could better describe the ΔΔ*T*_m_ values vs NDI molar equivalents trend.

Noteworthy, the evaluation of Δ*T*_m_ values obtained from FRET-melting experiments for 1:4 G-quadruplex/NDI ratio systems in 20 or 100 mM K^+^-containing buffer showed that the stabilizing effects of the NDIs on the target are reduced by 10 °C on average in the buffer with the highest K^+^ concentration tested, suggesting that the more the target is stabilized by the buffer conditions, the less the ligand can induce stabilizing effects (Figure 3D).

Notably, using the same experimental conditions, i.e., same buffer, investigated G-quadruplex/NDI system, and G-quadruplex/NDI ratio, the Δ*T*_m_ values obtained by FRET-melting experiments appear to be higher than those obtained by CD-melting (see the case of **52** in Table S2, for which a difference of +3.4 °C between the Δ*T*_m_ values was obtained by the two techniques).

**Figure 4:**
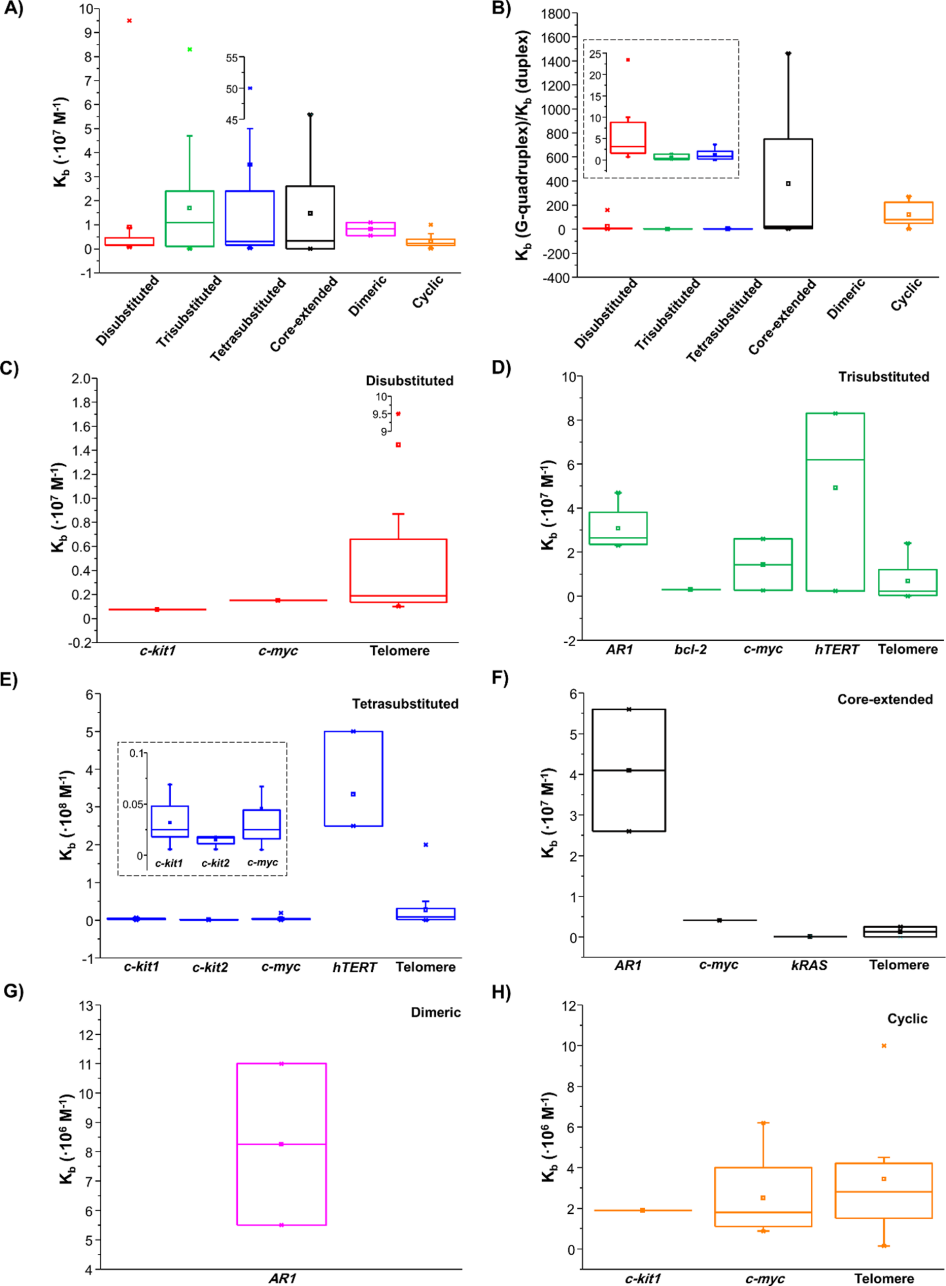
(A) *K*_b_ values for the six classes of NDIs interacting with G-quadruplex structures. (B) *K*_b_(G-quadruplex)/*K*_b_(duplex) ratios for the six classes of NDIs. Inset: enlargement of the bars related to di-, tri-, and tetrasubstituted NDIs. (C–H) *K*_b_ values for di-, tri-, and tetrasubstituted, core-extended, dimeric, and cyclic NDIs, respectively, interacting with G-quadruplex structures originating from the indicated genomic regions (gene promoters or telomeres). Inset in (E): enlargement of the bars related to *c-kit1*, *c-kit2*, and *c-myc* oncogene promoters.

By comparing the *K*_b_ values for the six different classes of NDIs interacting with G-quadruplex structures, it emerged that the trend of affinity of the NDIs for G-quadruplexes is as follows: tetrasubstituted > trisubstituted > core-extended > dimeric > disubstituted > cyclic (Figure 4A). As far as the G-quadruplex over duplex binding selectivity is concerned, evaluated from *K*_b_(G-quadruplex)/*K*_b_(duplex) ratios, the following trend was found: core-extended > cyclic > disubstituted = trisubstituted = tetrasubstituted, while no data are reported for dimeric NDIs (Figure 4B).

As in the case of thermal stability data, both the above trends should be taken only as a rough estimation of the real trends due to the fact that the available data were collected on NDIs tested in their ability of interacting with different G-quadruplexes at different buffer conditions and by exploiting different techniques, and binding constants are strictly dependent on these different experimental conditions.

Nevertheless, regardless of the experimental conditions and techniques used, it clearly appears that almost all the analyzed NDIs strongly bind all the investigated G-quadruplex structures, with *K*_b_ values ≥10^6^ M^–1^ (Figure 4C–H).

**Figure 5:**
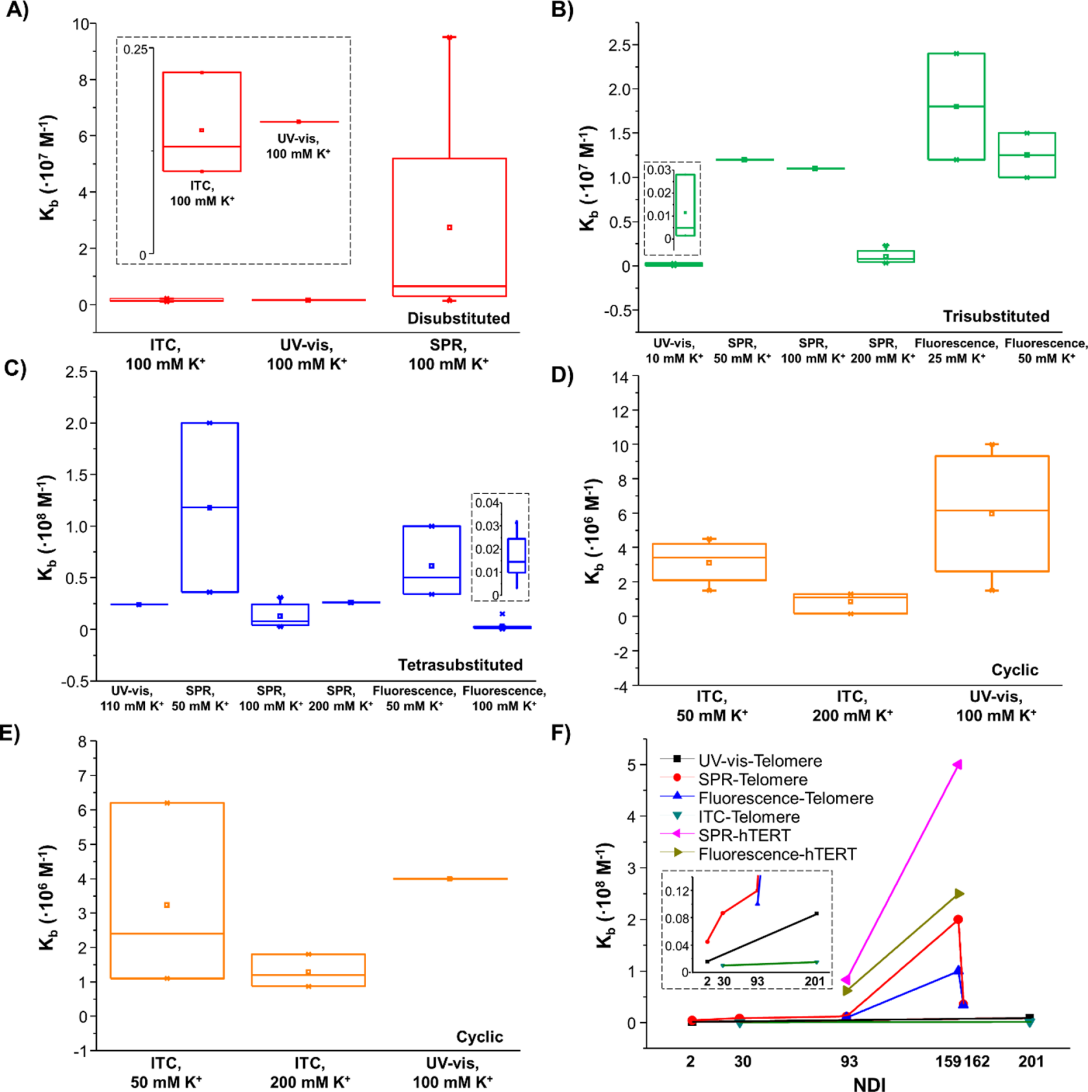
(A–D)*K*_b_ values for di-, tri-, and tetrasubstituted and cyclic NDIs, respectively, interacting with telomeric G-quadruplexes evaluated in buffers containing the indicated K^+^ concentration and by the indicated technique. Insets in (A), (B), and (C) show enlargements of the bars related to (A) disubstituted NDIs studied at 100 mM K^+^ concentration by ITC and UV–vis, (B) trisubstituted NDIs studied at 10 mM K^+^ concentration by UV–vis, and (C) tetrasubstituted NDIs studied at 100 mM K^+^ concentration by fluorescence. (E) *K*_b_ values for cyclic NDIs interacting with *c-myc* G-quadruplex evaluated in buffers containing the indicated K^+^ concentration and by the indicated technique. (F) *K*_b_ values evaluated for the indicated G-quadruplex/NDI couple in the same buffers and by the different indicated techniques. Inset: enlargement of the graph from 0 to 0.14 × 10^8^ M^–1^.

The dependence of *K*_b_ on the experimental conditions is as follows. In detail, *K*_b_ values for disubstituted NDIs interacting with telomeric G-quadruplexes were determined by ITC ((1.0–2.2) × 10^6^ M^–1^), UV–vis (1.6 × 10^6^ M^–1^), and SPR (1.4 × 10^6^–9.5 × 10^7^ M^–1^) in 100 mM K^+^-containing buffers (Figure 5A). *K*_b_ values for trisubstituted NDIs interacting with telomeric G-quadruplexes were determined by UV–vis in 10 mM K^+^-containing buffer (1.5 × 10^4^–2.8 × 10^5^ M^–1^), SPR in 50 mM K^+^-containing buffer (1.2 × 10^7^ M^–1^), 100 mM K^+^-containing buffer (1.1 × 10^7^ M^–1^), or 200 mM K^+^-containing buffer (3.2 × 10^5^–2.3 × 10^6^ M^–1^), and fluorescence in 25 mM K^+^-containing buffer ((1.2–2.4) × 10^7^ M^–1^) or 50 mM K^+^-containing buffer ((1.0–1.5) × 10^7^ M^–1^) (Figure 5B). *K*_b_ values for tetrasubstituted NDIs interacting with telomeric G-quadruplexes were determined by UV–vis in 110 mM K^+^-containing buffer (2.4 × 10^7^ M^–1^), SPR in 50 mM K^+^-containing buffer (3.6 × 10^7^–2.0 × 10^8^ M^–1^), 100 mM K^+^-containing buffer (2.7 × 10^6^–3.1 × 10^7^ M^–1^), or 200 mM K^+^-containing buffer (2.6 × 10^7^ M^–1^), and fluorescence in 50 mM K^+^-containing buffer (3.4 × 10^7^–1.0 × 10^8^ M^–1^) or 100 mM K^+^-containing buffer (3.2 × 10^5^–3.2 × 10^7^ M^–1^) (Figure 5C). *K*_b_ values for cyclic NDIs interacting with telomeric G-quadruplexes were determined by UV–vis in 100 mM K^+^-containing buffer (1.5 × 10^6^–1.0 × 10^7^ M^–1^) and ITC in 50 mM K^+^-containing buffer ((1.5–4.5) × 10^6^ M^–1^) or 200 mM K^+^-containing buffer (1.5 × 10^5^–1.3 × 10^6^ M^–1^) (Figure 5D). *K*_b_ values for cyclic NDIs interacting with *c-myc* G-quadruplex were determined by UV–vis in 100 mM K^+^-containing buffer (4.0 × 10^6^ M^–1^) and ITC in 50 mM K^+^-containing buffer ((1.1–6.2) × 10^6^ M^–1^) or 200 mM K^+^-containing buffer (8.7 × 10^5^–1.8 × 10^6^ M^–1^) (Figure 5E).

Moreover, comparing *K*_b_ values obtained for the same investigated G-quadruplex/NDI couple under the same conditions (Figure 5F), it appeared that (i) *K*_b_ values derived from SPR are ca. 2-fold higher than those obtained by fluorescence-based experiments (see the cases of **93** and **159** interacting with *hTERT* and telomeric G-quadruplexes, as well as **162** with a telomeric G-quadruplex); (ii) *K*_b_ values derived from SPR are ca. 3-fold higher than those obtained by UV–vis (see the case of **2** interacting with a telomeric G-quadruplex); (iii) *K*_b_ values derived from SPR are ca. 9-fold higher than those obtained by ITC (see the case of **30** interacting with a telomeric G-quadruplex); and (iv) *K*_b_ values derived from UV–vis are ca. 6-fold higher than those obtained by ITC (see the case of **201** interacting with a telomeric G-quadruplex).

**Figure 6:**
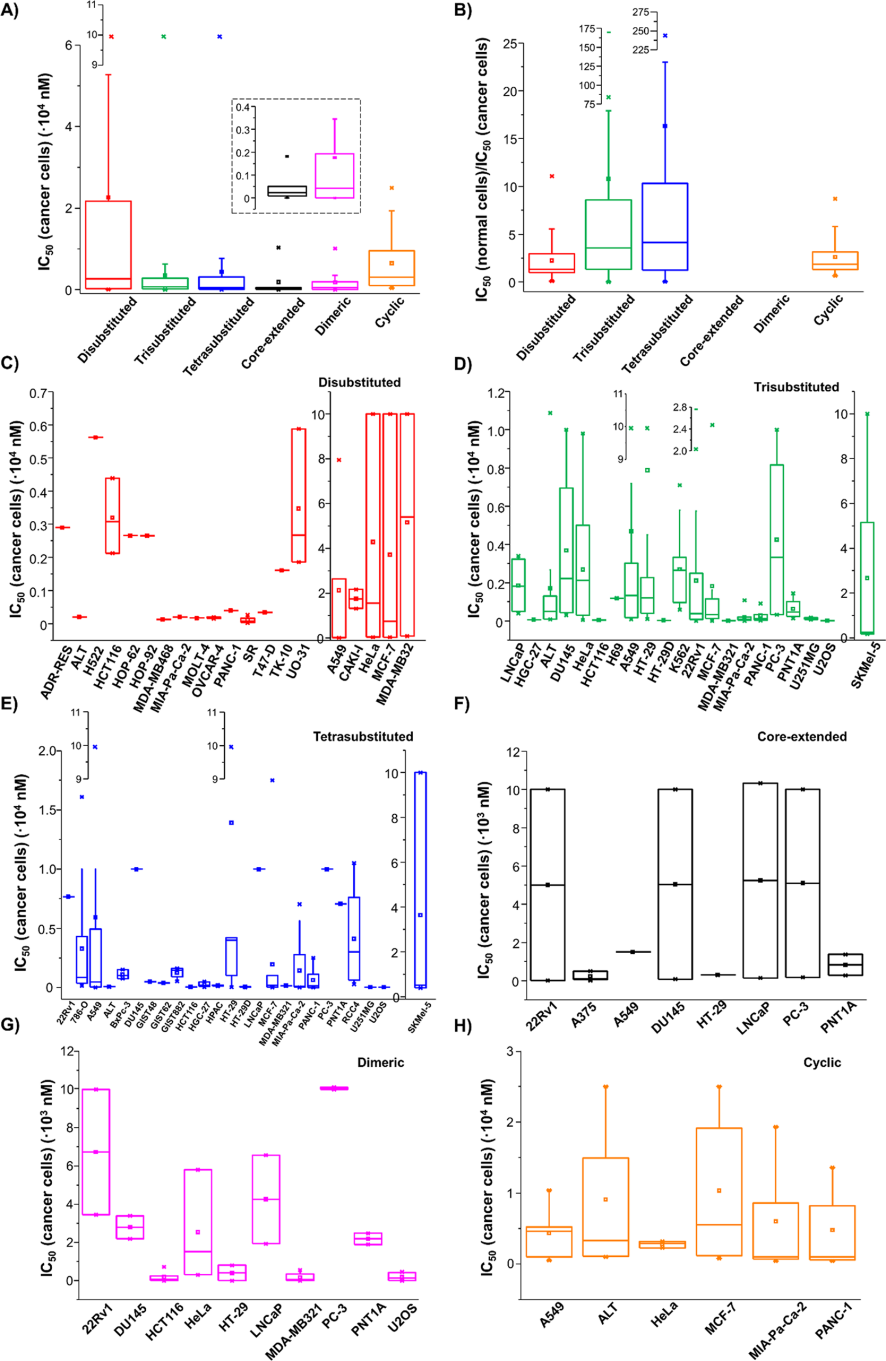
(A) IC_50_ values for the six classes of NDIs tested on cancer cells. Inset: enlargement of the bars related to core-extended and dimeric NDIs. (B) IC_50_(normal cells)/IC_50_(cancer cells) ratios for the six classes of NDIs. (C–H) IC_50_ values for di-, tri-, and tetrasubstituted, core-extended, dimeric, and cyclic NDIs, respectively, tested on the indicated cancer cell lines.

An analysis similar to the one carried out to obtain the Δ*T*_m_ and *K*_b_ values was performed for IC_50_ values obtained by in vitro assays on cancer cells (Figure 6A). By comparing the IC_50_ values for the six different classes of NDIs, regardless of the cancer cell lines used, it emerged that core-extended and dimeric NDIs have the highest antiproliferative activity in the series (IC_50_ values in the nM/sub-nM range), followed by tri- and tetrasubstituted NDIs (IC_50_ values in the nM range). Conversely, the lowest antiproliferative activity was found for disubstituted and cyclic NDIs, even if a good cytotoxicity was still detected (IC_50_ values in the μM/nM range). As concerns the cancer vs normal cells selectivity, evaluated from IC_50_(normal cells)/IC_50_(cancer cells) ratios, no data are reported for core-extended and dimeric NDIs, while the lowest selectivity was found for disubstituted and cyclic NDIs (Figure 6B). Thus, the latter two classes of NDIs appear to be the least promising classes of NDIs in terms of selective anticancer compounds. On the other hand, tri- and tetrasubstituted NDIs showed the highest cancer vs normal cells selectivity in the series (Figure 6B). Thus, considering both their good activity and selectivity toward cancer cells, tri- and tetrasubstituted NDIs emerged as the most appealing NDIs for cancer treatment in vivo.

To get further insights, for each class of NDIs the cancer cell lines on which anticancer activity was tested were taken in consideration (Figure 6C–H). In detail, the lowest antiproliferative activities (IC_50_ > 10 μM) were found for disubstituted NDIs on A549, CAKI-I, HeLa, MCF-7, and MDA-MB32; for trisubstituted NDIs on SKMel-5; for tetrasubstituted NDIs on HT-29 and SKMel-5; and for cyclic NDIs on ALT and MCF-7, while significant anticancer activities (IC_50_ values in the nM range) were detected for all six classes of NDIs on all the other investigated cancer cell lines (Figure 6C–H).

Unfortunately, no simple correlation between stabilizing ability of NDIs on the target and binding affinities for the target was found, and no overall relationship between these biophysical properties and in vitro/in vivo activity of NDIs was obtained, considering either the Δ*T*_m_(G-quadruplex), *K*_b_(G-quadruplex), and IC_50_(cancer cells) values or the Δ*T*_m_(G-quadruplex)/Δ*T*_m_(duplex), *K*_b_(G-quadruplex)/*K*_b_(duplex), and IC_50_(normal cells)/IC_50_(cancer cells) ratios.

**Figure 7:**
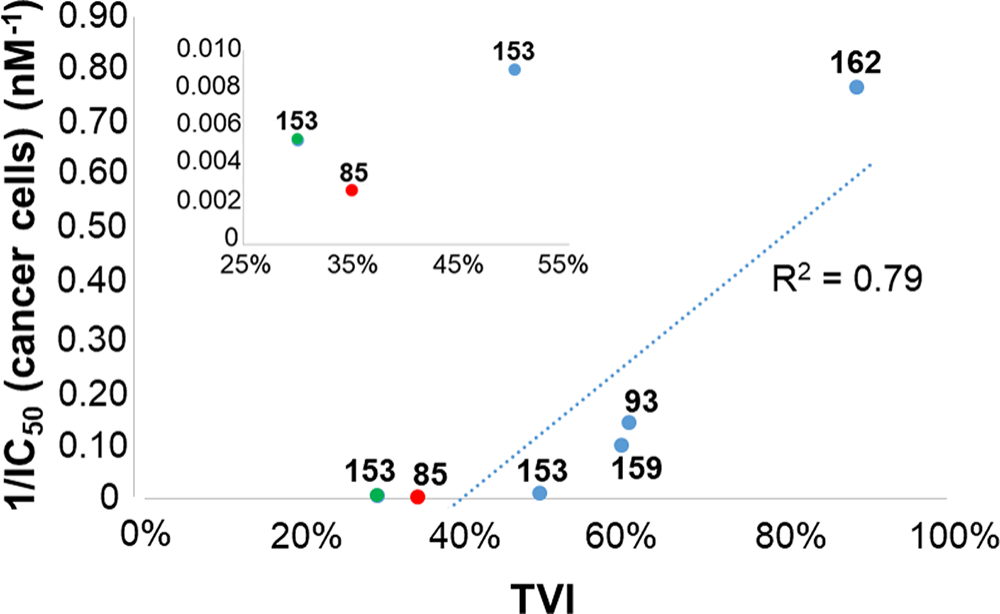
1/IC_50_(cancer cells) as a function of tumor volume inhibition (TVI) percentage for the indicated NDIs for the same tumor type. Blue and green dots refer to MIA-Pa-Ca-2 or HPAC pancreatic cancer, respectively, while red dots refer to HT-29 colon cancer. Linear regression line and the related *R*^2^ value are reported. Inset: enlargement of the graph from 25 to 55% and from 0 to 0.010 nM^–1^.

On the other hand, interestingly, a correlation between in vitro and in vivo activities of the tested NDIs could be extrapolated. Indeed, a good linear correlation was found between the reverse values of IC_50_ (cancer cells), i.e., 1/IC_50_(cancer cells), and the tumor volume inhibition (TVI) percentage by comparing each IC_50_ and TVI value for the same tumor type (Figure 7). Thus, on the basis of the IC_50_ values on cancer cells determined for an NDI in vitro, it can, in principle, be estimated the related TVI percentage in vivo that could be achieved with the optimal therapeutic regimen. Interestingly, a good correlation was found also between the in vitro anticancer selectivity, evaluated from IC_50_(normal cells)/IC_50_(cancer cells) ratios, and the adverse effects observed in vivo. Indeed, for **93**, **153**, and **159**, showing higher in vitro selectivity, no in vivo adverse effects were detected, whereas **43** and **85**, showing very low in vitro selectivity, induced a significant body weight loss in vivo. Therefore, on the basis of the available and comparable in vitro/in vivo data, it appears that IC_50_(normal cells)/IC_50_(cancer cells) ratios ≤3 or ≥23 result in significant or low-to-null adverse effects in vivo, respectively.

## Relationship between NDI Structures and Their Biophysical Properties, Anticancer Activity, and Binding Mode to G-Quadruplexes

In this section, correlations between NDI structures (Table S1) and their biophysical properties (Tables S2 and S3) and/or anticancer activity (Tables S5 and S6) are discussed for each of the six classes of known NDIs. A comparative analysis among these classes of G-quadruplex ligands is also presented. In addition, at the end of this section, NDI structures and related properties are correlated to NDI binding mode to G-quadruplexes (Table S7).

Taking in due consideration the strict dependence of biophysical properties and anticancer activity on the experimental conditions (as evidenced in the previous section), all the following insights were obtained by correlating NDI structures to their properties only if the latter ones were obtained under the same experimental conditions, i.e., same G-quadruplex/NDI ratio, buffer, technique and/or cell line.

### Disubstituted NDIs

**1** and **2** bear R1 = R2 = −(CH_2_)_2_N(CH_3_)_2_ or −(CH_2_)_3_N(CH_3_)_2_, respectively. **2** has higher stabilizing ability on G-quadruplexes than **1**, but **1** is more selective in stabilizing G-quadruplex over duplex structures than **2**. On the other hand, despite the activity on cancer cells is similar for both NDIs, **2** is more selective in killing cancer over normal cells than **1**.

Interestingly, in the case of **3**–**7** sharing the same R1 = −(CH_2_)_5_CONHOH and similar R2 substituents differing only in their length, which increases from **3** to **7**, the stabilizing effects on G-quadruplexes increase on increasing R2 length.

**8**–**11** and **13**–**16** differ only in the stereochemistry of the amino acid-based side chains. **8**–**11** have higher stabilizing abilities on G-quadruplexes than **13**–**16**, denoting that the stereochemistry of the side chains is relevant in defining the ligands best interacting with G-quadruplex structures. In particular, it can be inferred that only in the case of stereoisomers of L series, i.e., **8**–**11**, the amino groups in the side chains are directed toward the target, being involved in additional H-bonds compared to **13**–**16** and thus resulting in higher Δ*T*_m_(G-quadruplex) values.

**17** and **18**, sharing the same R1 and having R2 = −(CH_2_)_2_CH_3_ or −(CH_2_)_3_NH_2_, respectively, show far lower stabilizing properties on G-quadruplexes than **19**–**26**, having the same R1 as **17** and **18** but longer aminoalkyl substituents. This suggests that short alkyl or aminoalkyl side chains do not significantly contribute to the overall G-quadruplex/ligand complex stability.

**25** and **27** share the same R1 and differ in R2, which is −(CH_2_)_3_NH(CH_2_)_4_NH(CH_2_)_3_NH_2_ or −(CH_2_)_3_O(CH_2_)_4_O(CH_2_)_3_NH_2_, respectively. Interestingly, the substitution of nitrogen (**25**) with oxygen atoms (**27**) results in 10 °C decrease of the Δ*T*_m_ value (G-quadruplex), showing the relevance of protonable amines in those positions of R2 stabilizing the interaction with G-quadruplex structures. However, **27** is more selective toward G-quadruplex over duplex DNA, denoting that the nitrogen atoms in **25**—lacking in **27**—can be involved in stabilizing interactions (electrostatic or H-bonds) also with duplex structures. Therefore, the substitution of H-bond donor/acceptor atoms with H-bond acceptor atoms, not undergoing protonation at physiological pH, in specific position(s) of the NDI side chains could be beneficial to increase the G-quadruplex over duplex selectivity.

Moreover, comparing **26** with **25** and **22** with **19**, it emerges that the methylation of the terminal amino group results in higher thermal stabilization of G-quadruplex structures.

As far as **31** and **32** are concerned, the latter is more active and selective than **31** toward cancer cells and, notably, their activity and selectivity correlate linearly with their stabilizing properties on G-quadruplexes and ability to interact with G-quadruplexes better than with duplex DNA, as determined by melting experiments. This finding suggests that when an *N-*methylimidazolium moiety is connected to the NDI core through a three carbon atoms linker (**32**), the interaction to G-quadruplexes is stronger than in the case it is linked via a two carbon atoms linker (**31**), probably due to the better orientation in which the *N-*methylimidazolium can be accommodated in the G-quadruplex groove, thus forming additional interactions, when connected through a longer linker.

By comparing **35** with **36**, wherein **36** is more active on cancer cells and a stronger G-quadruplex ligand than **35**, it emerges that the introduction of a phenyl (**36**) in place of a methylene linker (**35**) between the NDI core and the attached side chains is beneficial to the interaction with G-quadruplexes and also results in higher activity against cancer cells.

Overall, it appears that disubstituted NDIs featuring longer side chains, having one or more nitrogen atoms with the terminal one methylated, are endowed with higher affinity and stabilizing ability to G-quadruplexes. However, a high number of nitrogen atoms on longer, and hence more flexible, side chains results in low G-quadruplex over duplex selectivity due to the higher number of protonable positions, producing more positively charged ligands, coupled with the ability of nitrogen atoms to act as H-bond donor/acceptor with both G-quadruplex and duplex DNA. In this regard, substitution of nitrogen with oxygen atoms in specific positions of the NDI side chain can modulate the affinity and selectivity of disubstituted NDIs toward G-quadruplex structures. On the other hand, when *N-*methylimidazolium is the terminal moiety of the R1/R2 groups of disubstituted NDIs, longer side chains are preferred to achieve both higher affinity/selectivity toward G-quadruplexes and higher selective anticancer activity.

### Trisubstituted NDIs

**41** and **48** bear R1 = R2 = −(CH_2_)_*n*_N(CH_2_CH_3_)_2_ and R3 = −NH(CH_2_)_*n*_N(CH_2_CH_3_)_2_, where *n* = 2 or 3 for **41** or **48**, respectively. **48** is more active on cancer cells and has higher stabilizing ability on G-quadruplexes than **41**, thus evidencing that the insertion of an additional methylene in R1, R2, and R3 is beneficial for the interaction with G-quadruplex structures as well as for the anticancer activity. Probably, the longer side chains in **48** make the terminal nitrogen atoms more prone to forming additional stabilizing interactions with the G-quadruplex target.

As far as **40**, **41**, **44**, and **48** are concerned, these ligands have R1 = R2 = −(CH_2_)_*n*_N(Y)_2_ and R3 = −NH(CH_2_)_*n*_N(Y)_2_, where Y = −CH_3_ and *n* = 2 or 3 for **40** and **44**, respectively, while Y = −CH_2_CH_3_ and *n* = 2 or 3 for **41** and **48**, respectively. **40** is more active on cancer cells than **41**, and **44** is more active than **48**, indicating that steric effects can be relevant on the terminal nitrogen atoms of NDI substituents in the context of developing trisubstituted NDIs as putative anticancer drugs.

Interestingly, a good correlation is found comparing the thermal stabilizing ability on G-quadruplexes, on one side, and the anticancer activity, on the other, of **51**, **52**, and **54**, which share the same R1 = R2 = −(CH_2_)_2_N(CH_3_)_2_ and differ in R3. Indeed, for both G-quadruplex stabilizing ability and anticancer activity, the same trend is observed, i.e., **52** > **51** > **54**, suggesting that the best substituent ortho to the −OH of the 4-hydroxyphenylethylamino group in R3 is the *N,N*-dimethylaminoethyl group. Interestingly, **53**, the methylated analogue of **52**, is even more active on cancer cells than **52**.

By comparing **46** with **68**–**71**, sharing similar R1 = R2 = −(CH_2_)_*n*_N(CH_3_)_2_ where *n* = 3 or 2, respectively, and differing in R3 = −NH(CH_2_)_2_O(CH_2_)_2_Y, where Y is a hydroxyl, pyrrolidino, piperidino, *N-*methylpiperazino, or morpholino group, respectively, it emerges that the anticancer activity trend is as follows: **46** > **68** > **69** > **71** > **70**, i.e., is strictly dependent on the terminal group in the side chain. Moreover, the optimal terminal group, conferring an activity higher by 2 orders of magnitude than the others, appears to be hydroxyl, followed by pyrrolidino, piperidino, morpholino, and *N-*methylpiperazino groups, listed from the best to the worst in the series.

On the other hand, when R1 = R2 = −(CH_2_)_2_N(CH_3_)_2_ and R3 = −NH(CH_2_)_2_[O(CH_2_)_2_]_2_Y (**72**–**75**) the anticancer activity depends on the Y terminal group with the following trend: piperidino > morpholino > pyrrolidino > *N-*methylpiperazino, while when R3 is one ethylene glycol unit longer, i.e., −NH(CH_2_)_2_[O(CH_2_)_2_]_3_Y (**76**–**79**), the anticancer activity varies with Y as follows: morpholino > piperidino > *N-*methylpiperazino > pyrrolidino. Interestingly, **47**, having the same R1 and R2 as **72**–**75**, i.e., R1 = R2 = −(CH_2_)_3_N(CH_3_)_2_ and similar R3, i.e., −NH(CH_2_)_2_[O(CH_2_)_2_]_2_Y with Y = −NH_2_, is more active than **72**–**75**.

Notably, **68**, **69**, and **71** are more active on cancer cells than **72**, **73**, and **75**, which in turn are more active than **76**, **77**, and **79**. These findings prove that (i) the trisubstituted NDIs with R1 = R2 = −(CH_2_)_2_N(CH_3_)_2_ and R3 = −NH(CH_2_)_2_O(CH_2_)_2_Y are the ones with the highest anticancer activity among the trisubstituted NDIs with ethylene glycol-based side chain in R3 and (ii) the anticancer activity reduces on increasing the length of ethylene glycol-based side chain. Only when the Y terminal group in R3 is *N-*methylpiperazine, the activity trend is not linearly correlated to the ethylene glycol chain length and indeed is as follows: **70** > **78** > **74**, showing the highest activity in the NDI with the shortest ethylene glycol chain. Moreover, the affinity trend toward G-quadruplexes for **71**, **75**, and **79** is **71** > **75** > **79**, confirming that (i) the shortest ethylene glycol-based side chain in R3 is the best choice for optimal targeting of G-quadruplex structures and (ii) the affinity is reduced on increasing the length of the ethylene glycol-based side chain.

**87**, **88**, and **89** respectively carry pyrrolidinoethyl, piperidinoethyl, and morpholinoethyl side chains at all R1, R2, and R3 positions. **87** is more active on cancer cells and has higher stabilizing ability on G-quadruplexes than **88**, which in turn is more active and stabilizing than **89**. Therefore, when the linker between the trisubstituted NDI core and the terminal group of the side chains is an ethyl moiety, pyrrolidine is preferred over piperidine and morpholine rings in terms of best G-quadruplex-targeting and enhanced anticancer activity.

**89** and **91** have morpholinoethyl or morpholinopropyl groups as side chains, respectively, in R1, R2, and R3. **91** is more active on cancer cells and has higher stabilizing ability on G-quadruplexes than **89**, denoting that, when the terminal group on R1, R2, and R3 of trisubstituted NDIs is a morpholino, a propyl linker results in more stabilizing interactions with the target, in turn producing higher anticancer activity, if compared with an ethyl linker.

Comparing **91** with **100**, sharing the same R1 and R2, i.e., morpholinopropyl substituents, and differing in R3, which is a morpholinopropylamino in **91** and an *N-*methylpiperazinopropylamino in **100**, it emerges that **100** is more active on cancer cells than **91**. On this basis, the *N-*methylpiperazinopropylamino group seems to be a better substituent in R3 than morpholinopropyl when morpholinopropyl substituents are also in R1 and R2.

**93**, **95**, and **97** carry a morpholinopropyl group at R1 and R2 positions, while pyrrolidinoethylamino, piperidinoethylamino, and morpholinoethylamino are their side chains in R3, respectively. When compared with **87**, **88**, and **89**—featuring pyrrolidinoethyl, piperidinoethyl, and morpholinoethyl side chains, respectively, in R1, R2, and R3—it emerges that **87**, **88**, and **89** are more active on cancer cells than **93**, **95**, and **97**, respectively, suggesting that, when R3 is the same group, R1 and R2 with shorter alkyl linkers connecting the heterocycle to the NDI core can result in a more favorable anticancer activity.

**95** is more active on cancer cells and has a higher stabilizing ability on G-quadruplexes than **97**, and in turn **96** is more active and stabilizing than **95**, proving that when R1 and R2 are morpholinopropyl substituents, a promising trisubstituted NDI is the one with *N-*methylpiperazinoethylamino side chain in R3.

Notably, **87**, **88**, and **90**—having pyrrolidinoethyl, piperidinoethyl, and pyrrolidinopropyl side chains, respectively, in R1 and R2, and pyrrolidinoethylamino, piperidinoethylamino, and pyrrolidinopropylamino side chains in R3—show similar anticancer activities and stabilizing properties on G-quadruplexes when compared respectively with **40**, **41**, and **44**—having *N*,*N*-dimethylaminoethyl, *N*,*N*-diethylaminoethyl, and *N*,*N*-dimethylaminopropyl side chains, respectively, in R1 and R2, and the corresponding alkylamino groups in R3. This is probably due to the similar spatial presentation of −N(CH_3_)_2_ and pyrrolidino terminal groups at R1, R2, and R3, as well as −N(CH_2_CH_3_)_2_ and piperidino residues, which could result in similar binding modes to the target DNA and, in turn, similar mechanisms of action in cancer cells.

Finally, **93** is more active on cancer cells and has a higher stabilizing ability on G-quadruplexes than **94**, suggesting that when R1 and R2 are both morpholinopropyl groups, tetrahydrofuran is a worse terminal group on R3 than pyrrolidino.

Overall, good combinations of substituents for trisubstituted NDIs to achieve both high target stabilizing ability and anticancer activity appear to be (i) R1 = R2 = −(CH_2_)_3_N(CH_3_)_2_ and R3 = −NH(CH_2_)_3_N(CH_3_)_2_, or (ii) R1 = R2 = −(CH_2_)_3_N(CH_3_)_2_ and R3 = −NH(CH_2_)_2_[O(CH_2_)_2_]_*n*_Y, where *n* = 1 and Y = −OH or *n* = 2 and Y = −NH_2_, or (iii) R1 and R2 are morpholinopropyl side chains and R3 is an *N*-methylpiperazinopropylamino or *N*-methylpiperazinoethylamino group.

### Tetrasubstituted NDIs

**106** and **109** share the same R1 and R2, which is −(CH_2_)_3_N(CH_3_)_2_, and differ in R3 and R4, which are −NH(CH_2_)_3_N(CH_3_)_2_ in the first compound and −NH(CH_2_)_3_OH in the second one. **106** is more active on cancer cells and has higher stabilizing ability on G-quadruplexes than **109**; however, **109** has a higher selectivity of action, better recognizing G-quadruplex than duplex structures and better interacting with cancer cells than with normal cells.

**106** and **107** have R1 = R2 = −(CH_2_)_3_N(Y)_2_ and R3 = R4 = −NH(CH_2_)_3_N(Y)_2_, where Y is a methyl or an ethyl group, respectively. **107** is more active on cancer cells and has a higher stabilizing ability on G-quadruplexes than **106**, suggesting the importance of an additional methylene group at the end of the side chains of tetrasubstituted NDIs to optimize their interaction with G-quadruplexes, beneficial also in terms of anticancer activity.

**102** and **106** bear R1 = R2 = −(CH_2_)_*n*_N(CH_3_)_2_ and R3 = R4 = −NH(CH_2_)_*n*_N(CH_3_)_2_, where *n* = 2 or 3, respectively. **106** has higher affinity and stabilizing ability toward G-quadruplexes than **102**, but **102** is more active on cancer cells than **106**, evidencing that in this case there is not a direct correlation, but actually an inverse correlation between G-quadruplex-targeting ability and anticancer effects. On the other hand, by comparing **103** with **107**, having R1 = R2 = −(CH_2_)_*n*_N(CH_2_CH_3_)_2_ and R3 = R4 = −NH(CH_2_)_*n*_N(CH_2_CH_3_)_2_, where *n* = 2 or 3, respectively, a perfect correlation between G-quadruplex stabilizing properties and anticancer activity is observed, with **107** both more stabilizing and active than **103**.

**116** and **117** are analogues sharing R1 = R2 = −(CH_2_)_2_N(CH_3_)_2_ and differing only in the substituent in the meta position with respect to the alkyne on the aromatic ring in R3 and R4, which is −CH_2_OH or −CH_2_N(CH_3_)_2_, respectively. **117** has a higher stabilizing ability on G-quadruplexes than **116**. As far as **118** and **119** are concerned, they have R1 = R2 = −(CH_2_)_2_N(CH_3_)_2_ and differ only in the relative position of hydroxyl and morpholino substituents on the aromatic ring in R3 and R4. **119** has a higher stabilizing effect on G-quadruplexes than **118**, suggesting that **119**, featured by that specific, relative position of substituents on the aromatic ring in R3 and R4, is a better G-quadruplex ligand.

**120**, **121**, and **122** share the same R1 = R2 = −(CH_2_)_2_N(CH_3_)_2_ and R4 = −Br and differ only in the substituent in the position ortho to the hydroxyl group on the aromatic ring in R3, which is −H, −(CH_2_)_2_N(CH_3_)_2_, or morpholinoethyl, respectively. **121** is more active on cancer cells than **120** and **122**. Notably, **121** has also higher stabilizing ability on G-quadruplexes than **120**, while no data are reported for **122**, thus showing for the first NDIs a good correlation between anticancer activity and ability to stabilize G-quadruplex structures.

**149** and **150** share the same R1 and R2, i.e., morpholinoethyl, and differ in the length of the linkers, ethyl or propyl, respectively, present in R3 and R4 connecting the *N-*methylpiperazine ring to the NDI core. Notably, it seems that an additional methylene group in R3 and R4 is beneficial for tetrasubstituted NDIs in terms of both the interaction with the G-quadruplex target and anticancer activity, and the G-quadruplex over duplex and cancer over normal cells selectivity.

**154** and **159** share the same R1 and R2, i.e., morpholinopropyl, and differ in the terminal groups on propyl linkers in R3 and R4, which are morpholino or *N*-methylpiperazino, respectively. **159** is more active and selective against cancer cells than **154**.

On the other hand, **150** and **159** bear the same R3 and R4, i.e., *N*-methylpiperazinopropylamino, and differ in R1 and R2, which are morpholinoethyl and morpholinopropyl, respectively. **159** is more active on cancer cells than **150**, even if their ability to stabilize G-quadruplex structures is similar.

**149** and **157** share the same R3 and R4, i.e., *N*-methylpiperazinoethylamino group, while they differ in R1 and R2, which are morpholinoethyl and morpholinopropyl, respectively. **157** has a higher stabilizing ability on G-quadruplexes than **149**, in line with its higher activity on cancer cells than **149**, suggesting that an additional methylene in R1 and R2 favors the interaction with G-quadruplexes and enhances the related anticancer activity of the ligand.

**151** and **158** are decorated with the same group at both R3 and R4, i.e., morpholinopropylamino, while they differ in R1 and R2, which are *N*-methylpiperazinoethyl and *N*-methylpiperazinopropyl, respectively. The IC_50_ values on cancer cells are lower for **158** than for **151**. Notably, **158** has higher stabilizing ability on G-quadruplexes than **151**, in line with its higher activity on cancer cells than **151**, proving also in this case that an additional methylene in R1 and R2 is beneficial for both the interaction of the NDI side chains with G-quadruplexes and the anticancer activity.

**144** and **156** are positional isomers, wherein R1 and R2 are tetrahydropyran-4-ylmethyl and R3 and R4 are *N*-methylpiperazinopropylamino groups in **144**, while R1 and R2 are *N*-methylpiperazinopropyl and R3 and R4 are tetrahydropyran-4-ylmethylamino groups in **156**. **156** is more active on cancer cells and has a higher stabilizing ability on G-quadruplexes than **144**. Additionally, **156** has higher G-quadruplex over duplex and cancer over normal cells selectivity than **144**.

**145**-**148** share the same linkers in R1–R4 (ethyl) and differ in the terminal groups on the four side chains, which are pyrrolidino, piperidino, *N*-methylpiperazino, and morpholino, respectively. The trend of both stabilizing ability on G-quadruplexes and anticancer activity is as follows: **145** > **146** > **147** > **148**, and hence in terms of terminal groups: pyrrolidino > piperidino > *N*-methylpiperazino > morpholino. On the other hand, the trend of G-quadruplex over duplex and of cancer over normal cells selectivity follows this order: **147** > **146** > **145** > **148**.

Finally, **152** and **154** share the same linkers in R1–R4 (propyl) and differ in the terminal groups on the four side chains, which are pyrrolidino and morpholino, respectively. **152** has higher stabilizing effects on G-quadruplexes, is more selective toward G-quadruplexes, and is more active on cancer cells than **154**.

Overall, for tetrasubstituted NDIs, it seems that higher G-quadruplex affinity and anticancer activity are achieved by combining four substituents R1–R4 including propyl linkers, rather than ethyl linkers, so to have R1 = R2 = −(CH_2_)_3_N(Y)_2_ and R3 = R4 = −NH(CH_2_)_3_N(Y)_2_. Moreover, contrarily to trisubstituted NDIs, ethyl is preferred to methyl at Y positions in enhancing G-quadruplex affinity and anticancer activity. Y can also be a heterocyclic group, and particularly the ones showing the highest stabilizing ability and anticancer activity are *N*-methylpiperazino, morpholino, and pyrrolidino. Tetrasubstituted NDIs with Y = *N*-methylpiperazino and/or pyrrolidino exhibit higher G-quadruplex and cancer cells selectivity than the ones having Y = morpholino. On the other hand, an additional good combination of substituents in tetrasubstituted NDIs to achieve good G-quadruplex over duplex and cancer over normal cells selectivity is R1 = R2 = −(CH_2_)_3_N(CH_3_)_2_ and R3 = R4 = −NH(CH_2_)_3_OH. Thus, in analogy to disubstituted NDIs, nitrogen-to-oxygen atoms replacement can improve the tetrasubstituted NDIs selectivity for G-quadruplexes and cancer cells.

### Core-Extended NDIs

**165**–**167** and **173** share the same R1 = R2 = −(CH_2_)_3_N(CH_3_)_2_, R3 = −H, X = −CH–, and differ only in R4, which is −H, −NO_2_, −COOH, and *N*-(morpholinoethyl)amido, respectively. Likewise, **168**–**171** and **174** share the same R1 = R2 = −(CH_2_)_3_N(CH_3_)_2_, R3 = −Br, and X = −CH–, and differ only in R4, which is −H, −NO_2_, −COOH, −Cl, and *N*-(morpholinoethyl)amido, respectively. Interestingly, their IC_50_ values on cancer cells show these trends: **167** > **173** > **166** > **165** and **170** > **174** > **171** > **169** > **168**, thus evidencing that in both series the highest anticancer activity is observed when R4 = −H and it progressively decreases when the hydrogen atom is replaced by (in the following order) a nitro, chlorine, *N*-(morpholinoethyl)amido, or carboxylic substituent. Notably, **168** is more active on cancer cells than **176**, both sharing the same R1–R4 and differing in X, which is −CH– or −N– respectively, evidencing that the substitution of −CH– with −N– in X position is not beneficial for increasing the anticancer activity. Comparing the activity on cancer cells of **165** with **177** and **168** with **178**, sharing the same substituents at R3, R4, and X positions, it emerges that methylation of the *N,N-*dimethylaminopropyl group in R1 and R2, providing quaternary ammonium salts (**177**, **178**), significantly reduces the anticancer activity. Finally, comparing the activities on cancer cells of **165**, **168**, **172**, and **175**, sharing the same R1 = R2 = −(CH_2_)_3_N(CH_3_)_2_, R4 = −H, and X = −CH– and differing in R3, which is −H, −Br, −Cl, and −NH(CH_2_)_3_N(CH_3_)_2_ respectively, the anticancer activity gradually decreases substituting hydrogen with (in the following order) a bromine, chlorine, or *N,N-*dimethylaminopropylamino substituent.

Overall, it seems that the optimal combination of substituents on the scaffold of core-extended NDIs to achieve the best anticancer activity is the simplest one, with R1 = R2 = −(CH_2_)_3_N(CH_3_)_2_, R3 = R4 = −H, and X = −CH–.

### Dimeric NDIs

**180**–**182** all carry R1 = −(CH_2_)_5_COOH, R2 = −(CH_2_)_3_N(CH_3_)_2_, and X = −(CH_2_)_2_[O(CH_2_)_2_]_2_– and differ in R3 and R4, which are −H/–H, −H/–Br, and −Br/–H, respectively. The highest anticancer activity in this series is observed when R3 and R4 are both −H, while the presence of a bromine atom significantly reduces the activity. In particular, the lowest activity in the series is observed when −Br is on the NDI core bearing the *N,N-*dimethylaminopropyl substituents.

On the other hand, **185**, **187**, and **189**, having the same R1 = R2 = −(CH_2_)_3_N(CH_3_)_2_, R3 = −H, and X = −(CH_2_)_4_– and differing in R4, which is −H, −Br, or −NH(CH_2_)_3_N(CH_3_)_2_ respectively, show similar ability in stabilizing G-quadruplex structures along with similar G-quadruplex over duplex selectivity. The highest activity is observed for **187** with R4 = −Br.

**186**, **188**, and **190** share the same R1 = R2 = −(CH_2_)_3_N(CH_3_)_2_, R3 = −H, and X = −(CH_2_)_7_– and differ in R4, which is −H, −Br, or −NH(CH_2_)_3_N(CH_3_)_2_, respectively. **186** and **188** have similar stabilizing ability on G-quadruplexes, higher than that of **190**, consistent with their similar anticancer activity, higher than that of **190**.

Notably, methylation of the terminal tertiary amino group in both R1 and R2 of dimeric NDIs results in lower stabilization of G-quadruplex structures when X = −(CH_2_)_7_– (cf. **186** with **192** and **188** with **194**), while no relevant differences are observed when X = −(CH_2_)_4_–(cf. **185** with **191** and **187** with **193**). On the other hand, methylation of the terminal amino group in R1, R2, and R4 produces a lower stabilization of G-quadruplex structures when X is both −(CH_2_)_7_– and −(CH_2_)_4_– (cf. **189** with **195** and **190** with **196**).

Interestingly, comparing dimeric NDIs having the same R1–R4 set of substituents on each monomeric NDI component, a remarkable enhancement of anticancer activity is observed by replacing the −(CH_2_)_4_– linker with the −(CH_2_)_7_– linker (cf. **185**, **187**, **189**, **191**, **193**, and **195** with **186**, **188**, **190**, **192**, **194**, and **196** respectively). However, an opposite trend is observed for the same compounds in terms of stabilizing ability on G-quadruplexes, which indeed decreases on increasing the linker length.

Overall, despite the high anticancer activity observed for dimeric NDIs among the six classes of NDIs, no general correlation among their structures, ability to stabilize G-quadruplex structures, and anticancer activity can be extrapolated.

### Cyclic NDIs

By comparing **200**–**203** in their affinity for telomeric and *c-myc* G-quadruplexes, the highest affinities are observed for **200** and **201** to telomeric and *c-myc* G-quadruplexes, respectively, thus evidencing that alkyldiamido and cycloalkyldiamido linkers in R3 are preferred in the interaction with hybrid and parallel G-quadruplexes, respectively.

As far as **205**–**207** are concerned, IC_50_ values trend on cancer cells is as follows: **206** > **207** > **205**, while the cancer vs normal cells selectivity trend is **205** > **206** > **207**, denoting that the most active and selective cyclic NDI carrying a ferrocene moiety is **205** with the bulky group in R3 lacking the additional alkyl spacers present in **206** and **207**. On the other hand, the highest ability to stabilize G-quadruplexes along with the highest affinity for these structures is observed for **207**, followed by **206** and **205**, thus suggesting that G-quadruplex structures could not be the only targets in cells for these NDIs and more complex mechanisms of action, involving or not G-quadruplexes, could occur.

**212** and **213** share the same R1 and R2, i.e., the piperazino group, and differ in R3, containing a cycloalkyl or phenyl moiety, respectively. Higher affinity and stabilizing properties on G-quadruplexes are found for **213**. However, no ability to discriminate G-quadruplexes over duplexes is observed for **213**, contrarily to **212**.

For cyclic dimeric NDIs **214**–**216**, differing only in the length of the linker connecting the two cyclic NDIs and particularly bearing propyl, pentyl, and heptyl linkers bridging the two carboxamide moieties respectively, the following trends are observed for the *K*_b_ (G-quadruplex) and *T*_m_ values (G-quadruplex): **215** > **214** > **216** and **214** > **215** > **216**. It can be concluded that the long heptyl linker disfavors the interaction with G-quadruplexes in terms of both binding constant and thermal stabilization.

**199** and **208**–**211** share the same R3 but differ in R1 and R2, which are −NH–, −NH(CH_2_)_2_NH–, −NH(CH_2_)_3_NH–, −NH(CH_2_)_4_NH–, and −O(CH_2_)_4_O–, respectively. Interestingly, a linear correlation between linker length and stabilizing properties on G-quadruplexes is observed for **199** and **208**–**210**, with increasing Δ*T*_m_ values on increasing the linker length. This can be explained considering that longer and more flexible linkers can easily direct toward G-quadruplex loops and flanking ends of the DNA target and be involved in a different pattern of interactions, ultimately resulting in higher stabilization of the target. On the other hand, a drastic reduction of Δ*T*_m_ value is observed for **211** compared to **210**, proving that, when the length of the linker in R1 and R2 is the same, the replacement of nitrogen with oxygen atoms is detrimental for the interaction with G-quadruplexes, probably due to the reduction of H-bond donor atoms within the cyclic NDI. As far as the G-quadruplex over duplex selectivity is concerned, evaluated from thermal stability data, the trend is as follows: **208** > **199** > **209** > **210** > **211**. This trend is the evidence that, despite the more effective G-quadruplex stabilizing properties of **209** and **210** compared to **208** and **199**, higher flexibility of linkers in R1 and R2 can result in lower G-quadruplex over duplex selectivity. Notably, an opposite trend is observed for **199** and **208**-**210** in terms of activity against cancer cells, with increasing IC_50_ values on increasing the linker length. On the other hand, the highest cancer vs normal cell selectivity values are observed for **208**, in line with the Δ*T*_m_ selectivity trend.

**203** has higher G-quadruplex affinity and selectivity than **213**, analyzing both their binding constant and Δ*T*_m_ values. Notably, a similar behavior is observed comparing **201** with **212**, thus proving that the substitution of a piperazino with a methylamino group in R1 and R2 is beneficial for the binding to G-quadruplex structures.

Overall, cyclic dimeric NDIs show higher affinity and stabilizing ability toward G-quadruplexes than cyclic monomeric NDIs. Unfortunately, no data related to the activity on cancer cells of cyclic dimeric NDIs is reported in the literature, and therefore no comparison with cyclic NDIs can be carried out from a biological point of view.

### Comparative Analysis among the Six NDI Classes

Tetrasubstituted **102** bears R4 = −NH(CH_2_)_2_N(CH_3_)_2_ and the same R1 = R2 = −(CH_2_)_2_N(CH_3_)_2_ and R3 = −NH(CH_2_)_2_N(CH_3_)_2_ as trisubstituted **40**. **102** has higher stabilizing ability on G-quadruplexes and anticancer activity than **40**, while **40** is more selective toward G-quadruplex structures and cancer cells compared to **102**, thus proving that adding an *N,N-*dimethylaminoethylamino on the core of a trisubstituted NDI is beneficial in terms of interaction with G-quadruplexes and overall antiproliferative activity, but this also results in a loss of selectivity of binding to G-quadruplexes and of ability to discriminate cancer over normal cells. On the other hand, adding an *N,N-*dimethylaminoethylamino in R3 to a disubstituted NDI (cf. **40** with **1**) enhances both the affinity to G-quadruplexes with related anticancer activity and the G-quadruplex over duplex selectivity with related cancer over normal cells selectivity.

Disubstituted **2**, trisubstituted **44**, and tetrasubstituted **106** bear the same R1 = R2 = −(CH_2_)_3_N(CH_3_)_2_, with **44** and **106** having one (in R3) or two (in both R3 and R4) additional *N,N-*dimethylaminopropylamino groups, respectively. **106** has higher affinity toward G-quadruplexes and higher stabilizing ability on G-quadruplexes than (in the following order) **44** and **2**. However, **44** is the most active NDI analogue on cancer cells in this series, and due to its lower, compared to **106**, but still high stabilizing properties on G-quadruplex targets, it emerges as a more promising NDI than **2** and **106**.

Trisubstituted **48** and tetrasubstituted **107** both share the same R1 = R2 = −(CH_2_)_3_N(CH_2_CH_3_)_2_ and R3 = −NH(CH_2_)_3_N(CH_2_CH_3_)_2_. In turn, **107** has an additional substituent on the NDI core, i.e., R4 = −NH(CH_2_)_3_N(CH_2_CH_3_)_2_. **107** has higher affinity and selectivity to G-quadruplexes, higher stabilizing ability on G-quadruplexes vs duplexes, and higher cancer vs normal cells activity than **48**.

Trisubstituted **86** and **87** have a pyrrolidinomethyl or pyrrolidinoethyl group in R1 and R2, and a pyrrolidinomethylamino or pyrrolidinoethylamino substituent in R3 position, respectively, while **141** and **145** share the same R1, R2, and R3 as **86** and **87** and bear pyrrolidinomethylamino or pyrrolidinoethylamino as R4 substituents, respectively. **141** and **145** have higher selective stabilizing ability on G-quadruplexes over duplexes, along with higher selective activity on cancer over normal cells than **86** and **87**, respectively.

Finally, the second NDI unit of dimeric **180** and **182** can be compared with trisubstituted **46** and **47**. Indeed, R4 = −H in both **180** and **182** and the R2 substituents of **180** and **182** are the same as R1 and R2 of **46** and **47**, i.e., −(CH_2_)_3_N(CH_3_)_2_, while X of **180** and **182** as well as R3 of **46** and **47** are in all cases ethylene glycol-based chains. Notably, the activity on cancer cells of **46** and **47** is far higher than the anticancer activity of **180** and **182**, evidencing that the first NDI unit of **180** and **182**, carrying two carboxylic acid moieties and thus reducing the overall charges of the NDI analogues, seems to reduce the antiproliferative effects on cancer cells.

Overall, it emerges that adding a substituent on the core of disubstituted NDIs, thus obtaining trisubstituted NDIs, results in higher and more selective ability to interact with G-quadruplexes over duplexes and, in parallel, higher and more selective activity on cancer over normal cells. On the other hand, adding a substituent on the core of trisubstituted NDIs, thus obtaining tetrasubstituted NDIs, always results in higher stabilizing ability on G-quadruplexes, while the effects on G-quadruplex over duplex selectivity and on the anticancer activity are strictly dependent on the whole pattern of substituents present on the core of both trisubstituted and tetrasubstituted NDIs. Lastly, monomeric NDIs appear to be better anticancer agents than dimeric NDIs.

### Correlation among NDI Structures, Properties, and Binding Mode to G-Quadruplexes

**18**, **20**, and **23** share the same R1 and differ in the length of the aminoalkyl/polyamino substituents in R2. **18** shows lower stabilizing ability on G-quadruplexes than **20** and **23**. This behavior can be explained considering that it has only one protonable nitrogen atom and no polyamine chain—which in contrast is the case of **20** and **23**—thus resulting in a lower number of hydrogen bonds and electrostatic interactions with the G-quadruplex target (Table S7).

**199** and **208**–**211** bear the same R3 and differ in the length of the linkers in R1 and R2. **199**, **208**, and **209** interact with the grooves of a hybrid telomeric G-quadruplex, with the number of hydrogen bonds/electrostatic interactions increasing going from **199** to **208** to **209** (Table S7), thus justifying the observed trend of stabilization of G-quadruplexes, i.e., **209** > **208** > **199**. In addition to the hydrogen bonds/electrostatic interactions, **210** forms also stacking interactions (Table S7) due to its different binding mode to the G-quadruplex with respect to **199**, **208** and **209**, i.e., stacking on the outer quartet, thus explaining why **210** has higher stabilizing ability on G-quadruplexes than **199**, **208**, and **209**. On the other hand, also **211** is able to target the outer quartet of the hybrid G-quadruplex model, but, in addition to the stacking interactions, only one hydrogen bond is formed with the target. This proves that the lower ability of stabilizing G-quadruplexes for **211** compared to **199** and **208**–**210** is due to the lower number of hydrogen bonds/electrostatic interactions that the oxygen-rich linker (**211**) in R1 and R2 can form with respect to nitrogen-rich linkers (**199** and **208**–**210**). On the other hand, **199** and **208**–**210** show a lower number of hydrogen bonds/electrostatic interactions with the duplex than to the G-quadruplex model compared to **211** (Table S7). These binding features well match the lower binding energies with the duplex obtained in silico, resulting in a good G-quadruplex over duplex selectivity for **199** and **208**–**210** and higher affinity for the duplex than the G-quadruplex observed for **211**.

**201** and **212** share the same R3 and differ in R1 and R2, which are the methylamino and piperazino groups, respectively. **201** has higher affinity toward G-quadruplexes along with higher stabilizing ability of the G-quadruplex targets than **212**. This behavior fully correlates with the binding poses of **201** and **212** in a G-quadruplex model (Table S7), wherein the best interactions with loop and flanking end residues is observed for **201**, which is therefore better accommodated than **212** in the binding pocket, ultimately resulting in a more stable complex.

Overall, a good correlation is found among the NDI structures, along with related biophysical properties defining the interaction with G-quadruplexes and the NDI binding modes as well as the types of interactions involved in the binding evaluated by structural studies. In addition, also the G-quadruplex over duplex discriminating ability can be well explained by considering the G-quadruplex vs duplex selectivity evaluated by molecular modeling studies.

## Summary and Perspectives

Simultaneously and selectively targeting telomeric and oncogenic G-quadruplexes by small organic molecules can produce a synergistic antiproliferative action against cancer cells, without side effects on normal cells. As a main requirement to evolve candidate drugs featured by low-to-null toxicity, a G-quadruplex ligand has to specifically recognize genomic G-quadruplexes, discriminating duplex DNA.

In this regard, NDIs emerged as attractive compounds because of their ability to selectively bind multiple G-quadruplex structures, resulting in strong and targeted anticancer activity both in vitro and in vivo. The chemical accessibility and high potential of this class of compounds as anticancer agents stimulated the synthesis of more than 200 different NDIs in the past two decades, leading them to be the most in-depth investigated class of G-quadruplex-targeting compounds with the highest number of structural analogues thus far produced.

Therefore, we undertook a systematic analysis on NDIs aimed at elucidating the structure–activity relationships of these putative anticancer G-quadruplex-targeting drugs. Here, a detailed analysis of the plethora of biophysical, in vitro, in vivo, and structural data acquired for NDIs throughout the past two decades has been reported for the first time. Data evaluation for single NDIs led to general conclusions extrapolated for whole classes of NDIs, always taking in due consideration and comparing the available data for both G-quadruplex targets and control duplexes, as well as both cancer and normal cells.

NDIs show strong affinity for G-quadruplex structures and in most cases even a high G-quadruplex over duplex selectivity. In addition, their ability to strongly interact with G-quadruplexes of different topologies, located in both telomeres and oncogene promoters, proves that these compounds can act as multi-targeting agents with enhanced anticancer activity. Notably, NDIs are not only able to stabilize G-quadruplex structures, but also to induce G-quadruplex formation. Stacking interactions with one or both outer quartets of monomeric G-quadruplexes, as well as with both the quartets at G-quadruplex-G-quadruplex interface of dimeric G-quadruplexes, appear to be the preferential binding mode, regardless of the target G-quadruplex topology. However, interactions with grooves and loops are also possible through the substituents and/or the core of the NDIs. Notably, a general preference for the most accessible quartet of the G-quadruplex is observed in the first binding event, while the role of the G-quadruplex loops is notable in the context of interdependence of secondary binding events. On the other hand, binding to the grooves is always observed when the target is a duplex structure, even when the duplex contains an intercalative binding site. The good G-quadruplex over duplex selectivity proved to be mainly due to the higher number of interactions and in turn higher binding energies found for G-quadruplex than duplex structures.

Due to the small surface of the NDI core with respect to the quartets, the NDI core can simultaneously interact with the four guanines involved in the quartet, but it is not able to cover the whole surface of a quartet. Thus, two possible planar orientations onto the quartet are observed: symmetrical and asymmetrical. An asymmetrical position of the NDI core on the quartet allows maximizing the interactions of substituents with the grooves, while in a symmetrical position none of the substituents is very close to the grooves and their interactions seem to be averaged. Altogether the NDI core position with respect to the quartet and both the length of the NDI side chains and the functional groups on their substituents play relevant roles in determining the binding mode to G-quadruplexes and stability of the resulting G-quadruplex/NDI complexes. In general, electrostatic interactions and direct or water-mediated hydrogen bonds are the most common interactions of the substituents with the grooves/loops, even though T-shaped π–π stacking as well as face-to-face stacking interactions were also observed.

High and general anticancer activity of NDIs is proved by their ability to inhibit at low nanomolar concentrations the proliferation of breast, ovarian, cervical, prostate, lung, colon, renal, pancreatic, gastric, and gastrointestinal cancer cells, as well as leukemia, melanoma, osteosarcoma, and glioblastoma. Notably, higher IC_50_ values were found for normal cell with respect to cancer cell lines, strongly corroborating the potential of NDIs in the context of a real application on humans. Different in-cell mechanisms can be associated with the observed anticancer activity of NDIs: (i) targeting of telomeres, triggering telomere uncapping, telomere end-to-end fusion, and telomerase activity inhibition, and/or (ii) down-regulation of genes rich in putative G-quadruplex-forming sequences, especially oncogenes, involved in tumor onset and progression, DNA repair, telomere maintenance, and cell-cycle regulation. Moreover, NDIs easily reach the DNA targets in the cells, showing excellent cell internalization and nuclear localization.

From in vivo studies, it can be deduced that all the tested NDIs do not require complex formulations to result into effective drugs. Indeed, due to their excellent water solubility, they are well suited to be administered in aqueous solutions intravenously, as largely preferable in chemotherapy of tumors. Notably, these compounds easily penetrate and accumulate in tumor mass, in some cases leading to complete tumor regression. Moreover, they feature good pharmacokinetic and toxicity profiles.

Correlations between NDI structures and their biophysical properties and/or anticancer activity have been here discussed for each NDI for which data were available in the literature. The best pattern of substituents for G-quadruplex over duplex and/or cancer over normal cells selective targeting has been inferred for the different classes of NDIs by analyzing each NDI on the basis of the existing data. In addition, NDI structures and related properties have been correlated to NDI binding mode to G-quadruplexes vs duplexes. A good correlation is found between the NDI structures and the related biophysical properties defining the interaction with G-quadruplexes and the NDI binding modes to target, as well as the types of interactions involved in the binding. In addition, also the G-quadruplex over duplex discriminating ability can be well explained by considering the different binding modes to G-quadruplexes vs duplexes derived by structural studies.

The correlations here found can be useful to design novel and more selective G-quadruplex-targeting NDIs, or, more in general, new classes of selective G-quadruplex-targeting compounds in the context of the development of highly effective anticancer drugs with low-to-null toxicity.

Finally, due to the high potential of NDIs as anticancer agents, with this work we intend to offer an incentive to advance the most active and selective compounds—among all the described ones or new, next-generation analogues—from preliminary in vivo studies to more in-depth preclinical as well as clinical studies.

## Supporting Information Available

Table S1, chemical structures of NDIs; Tables S2–S4, biophysical data (Δ*T*_m_, *K*_b_, binding stoichiometry) for NDIs interacting with G-quadruplexes and duplexes; Table S5, in vitro data for NDIs on cancer and normal cells; Table S6, in vivo antitumor data for NDIs; and Table S7, crystallographic and molecular modeling structures of NDIs interacting with G-quadruplexes and duplexes (PDF)

## Abbreviations Used

ALT: alternative telomere lengthening
AR: androgen receptor
CPG: controlled pore glass
CRPC: castration-resistant prostate cancer
DDR: DNA damage response
DLS: dynamic light scattering
G4-CPG: G-quadruplex on controlled pore glass
G4-FID: G-quadruplex fluorescent intercalator displacement
GIST: gastrointestinal tumors
ITC: isothermal titration calorimetry
*K*_b_: binding constant
MTC: medullary thyroid cancer
NDI: naphthalene diimide
PDAC: pancreatic ductal adenocarcinoma
SPR: surface plasmon resonance
*T*_m_: melting temperature
TO: thiazole orange
TVI: tumor volume inhibition
