# Supplementary material for: Disentangling the Structure–Activity Relationships of Naphthalene Diimides as Anticancer G-Quadruplex-Targeting Drugs
Source: J Med Chem. 2021 Mar 22;64(7):3578–603. doi: 10.1021/acs.jmedchem.1c00125 (PMC8041303; doi:10.1021/acs.jmedchem.1c00125)
Supplement: Supplementary file 1 — jm1c00125_si_001.pdf [file jm1c00125_si_001.pdf]

Supplementary Information

to the manuscript:

Disentangling the structure-activity relationships of naphthalene diimides as anticancer G-quadruplex targeting drugs

Chiara Platella[1], Ettore Napolitano[1], Claudia Riccardi[1], Domenica Musumeci[1,2], Daniela Montesarchio[1,*]

[1]Department of Chemical Sciences, University of Naples Federico II, via Cintia 21, I-80126 Naples, Italy

[2]Institute of Biostructures and Bioimages, CNR, via Mezzocannone 16, I-80134 Naples, Italy

*To whom correspondence should be addressed.

Email: daniela.montesarchio@unina.it

Present Address: Daniela Montesarchio, Department of Chemical Sciences, University of Naples Federico II, via Cintia 21, I-80126 Naples, Italy

Tel: +39 081 674126; Fax: +39 081 674313

Table S1. Chemical structures of the here discussed disubstituted, trisubstituted, tetrasubstituted, core-extended, dimeric and cyclic NDIs.

Compound	R1	R2	Refs.
**Disubstituted NDIs**			
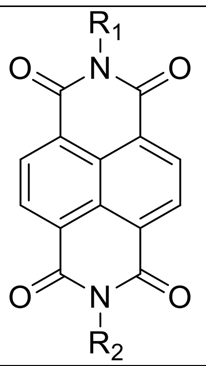			
**1**	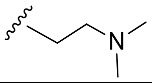	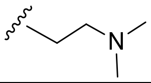	1,2
**2**	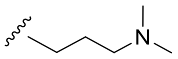	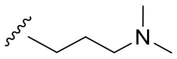	1–6
**3**	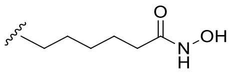	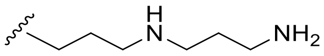	7
**4**	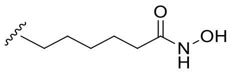	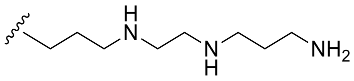	7
**5**	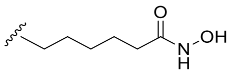	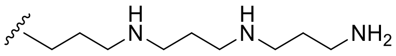	7
**6**	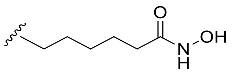	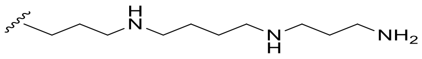	7
**7**	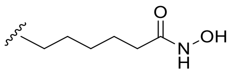	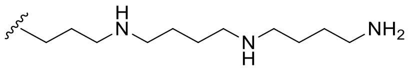	7
**8**	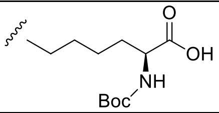	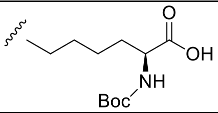	8,9
**9**	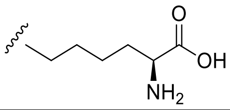	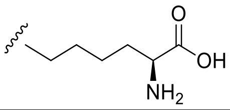	8,9
**10**	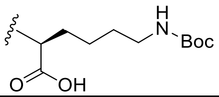	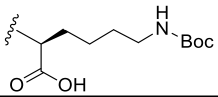	8,9
**11**	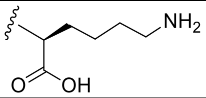	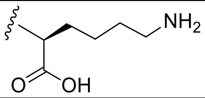	8,9
**12**	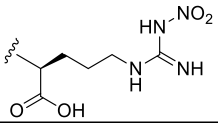	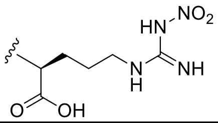	8
**13**	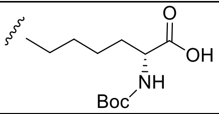	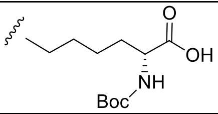	9
**14**	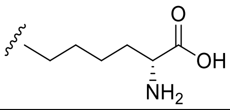	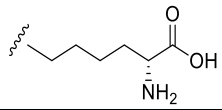	9
**15**	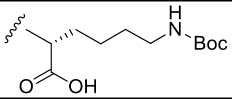	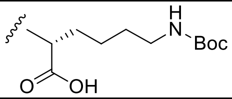	9
**16**	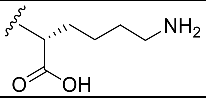	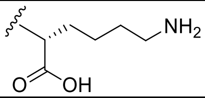	9
**17**	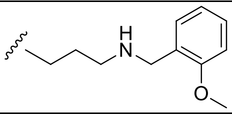	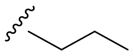	10
**18**	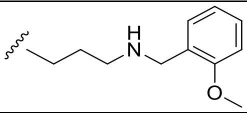	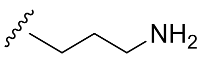	10

**19**	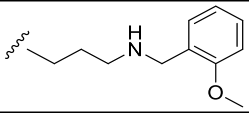	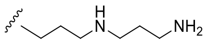	10
**20**	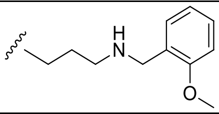	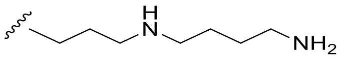	10
**21**	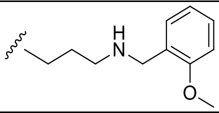	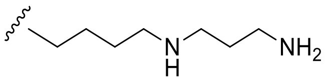	10
**22**	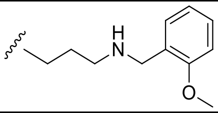	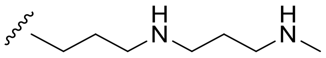	10
**23**	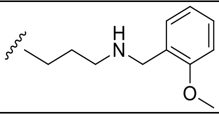	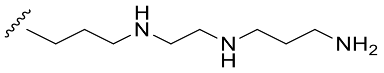	10
**24**	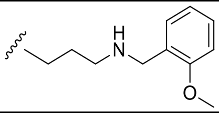	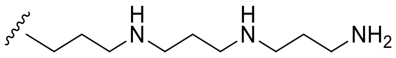	10
**25**	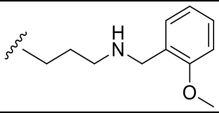	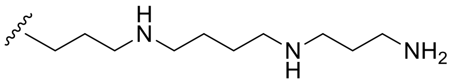	7,10
**26**	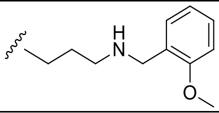	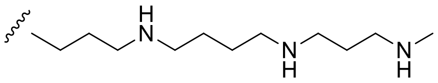	10
**27**	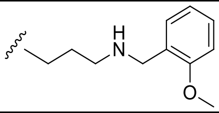	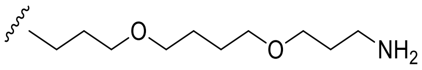	10
**28**	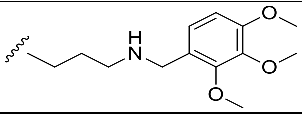	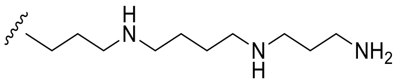	10
**29**	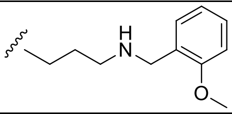	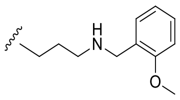	10,11
**30**	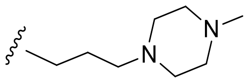	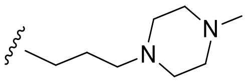	6,12
**31**	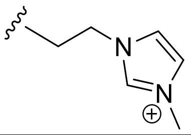	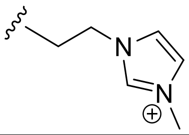	6
**32**	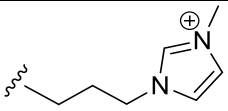	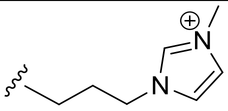	6
**33**	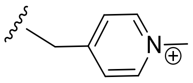	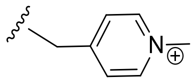	13
**34**	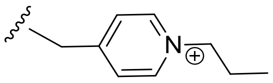	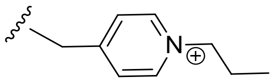	13
**35**	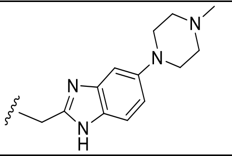	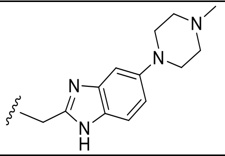	12
**36**	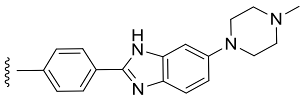	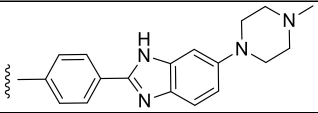	12
**37**	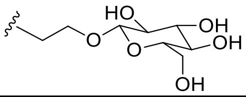	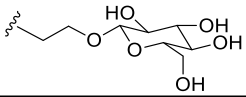	6
**38**	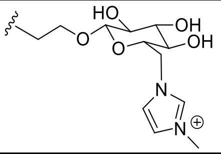	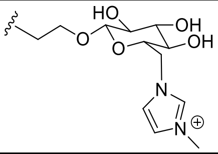	6

**39**	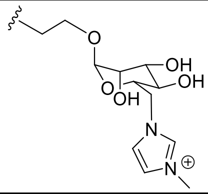	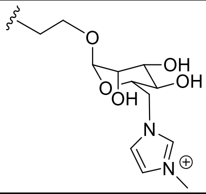	6

Trisubstituted NDIs

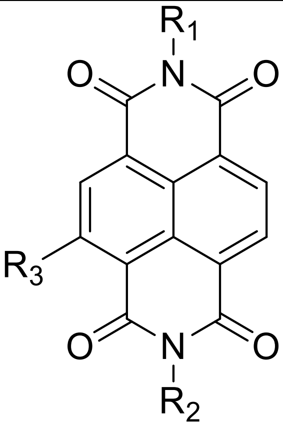

**Compound**	**R1**	**R2**	**R3**	**Refs.**
**40**	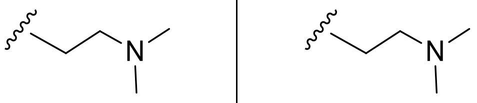		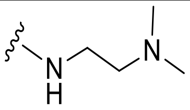	1,2
**41**	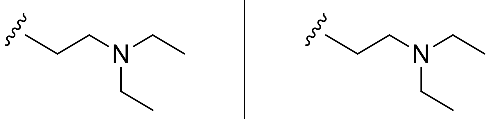		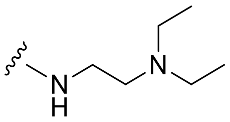	1,2
**42**	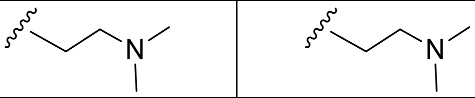		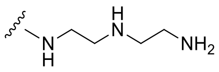	14
**43**	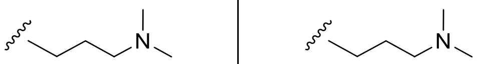		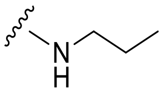	15–17
**44**	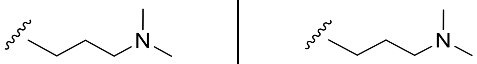		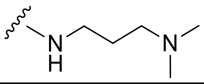	1,2,18,19
**45**	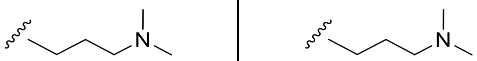		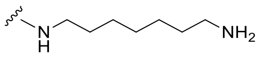	17
**46**	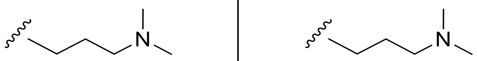		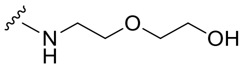	17,20,21
**47**	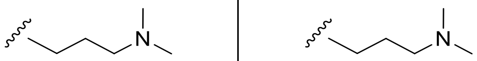		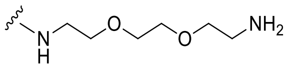	17
**48**	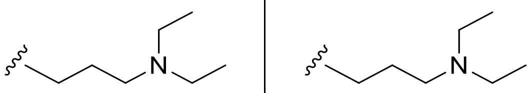		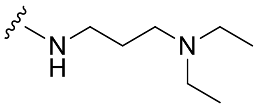	1,2
**49**	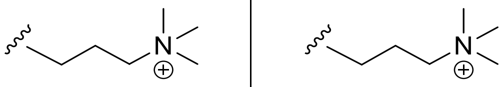		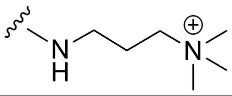	19
**50**	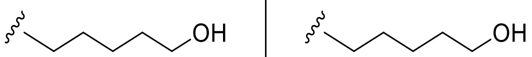		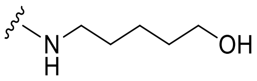	1,2
**51**	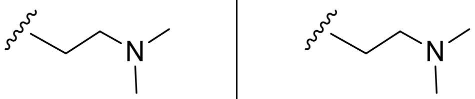		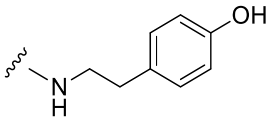	22,23
**52**	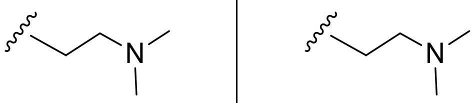		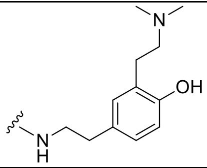	18,22–25
**53**	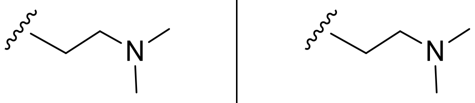		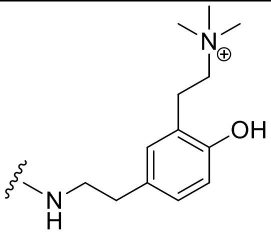	23

**54**	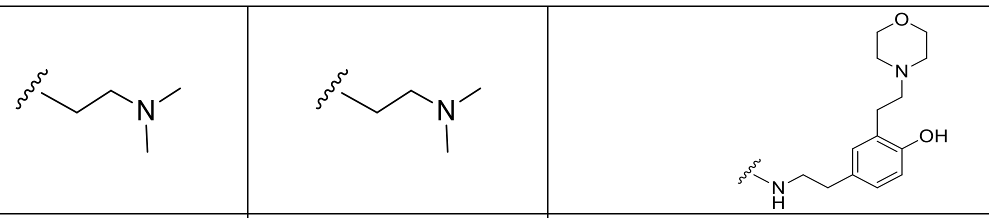			23
**55**	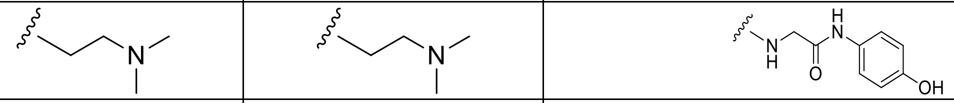			26
**56**	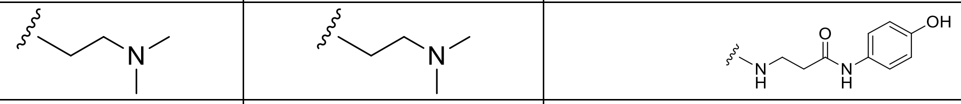			26
**57**	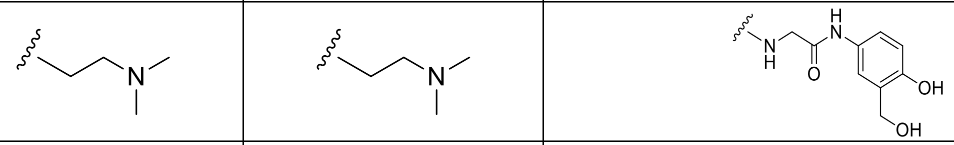			18,26
**58**	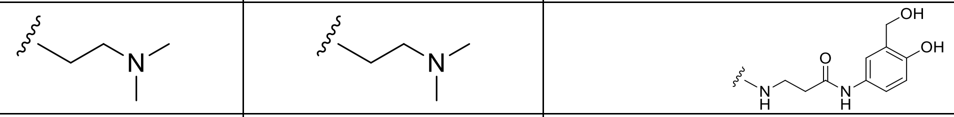			26
**59**	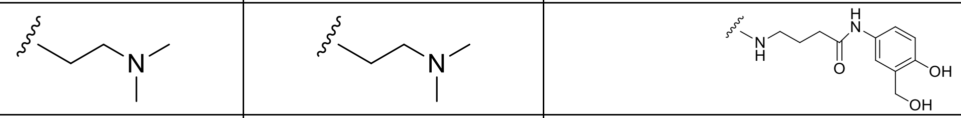			26
**60**	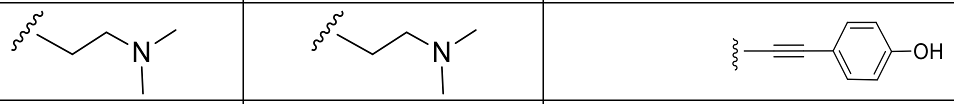			27
**61**	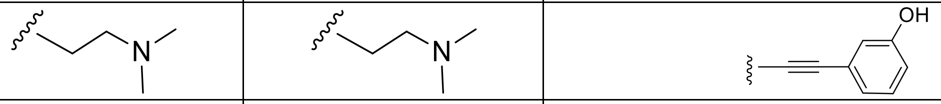			27
**62**	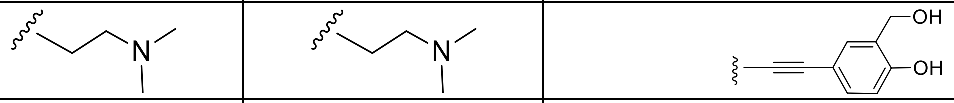			27
**63**	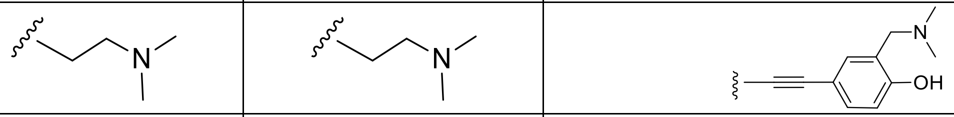			27
**64**	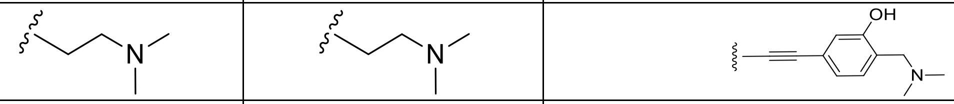			18,27
**65**	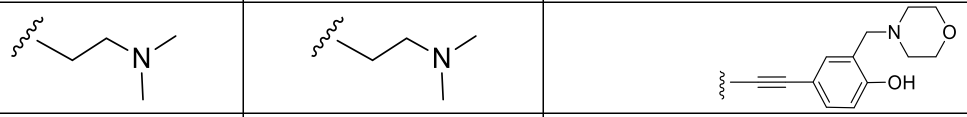			27
**66**	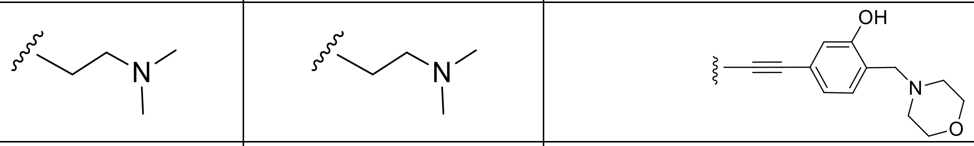			27
**67**	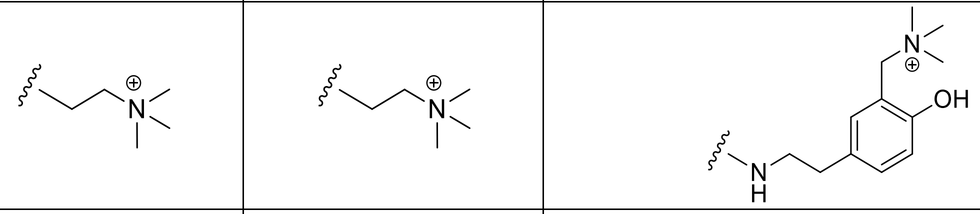			23
**68**	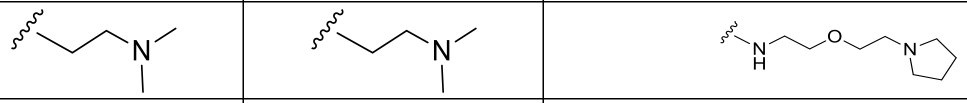			28
**69**	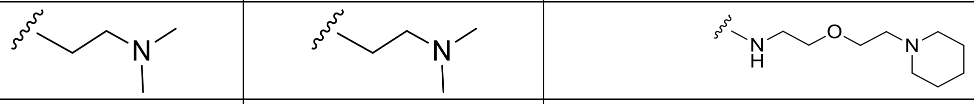			28
**70**	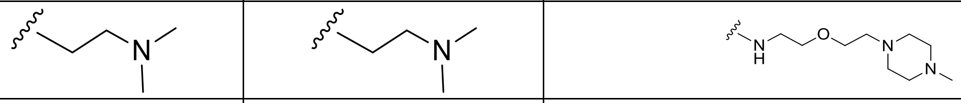			28
**71**	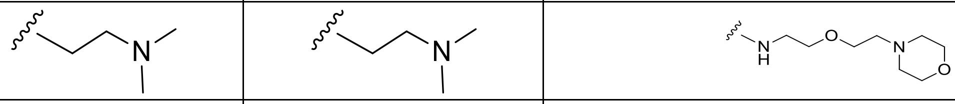			28
**72**	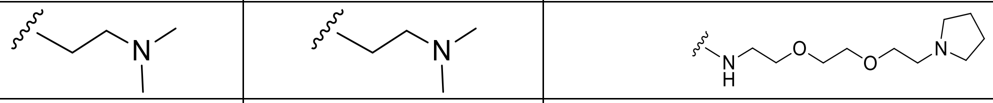			28
**73**	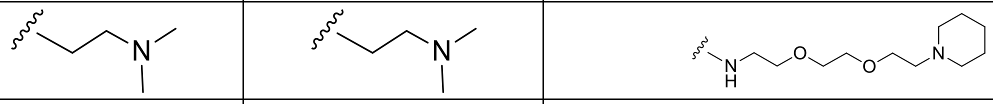			28
**74**	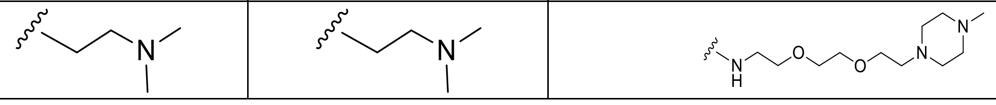			28

**75**	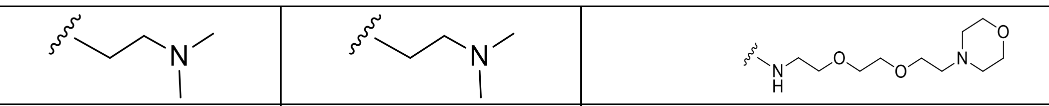			28
**76**	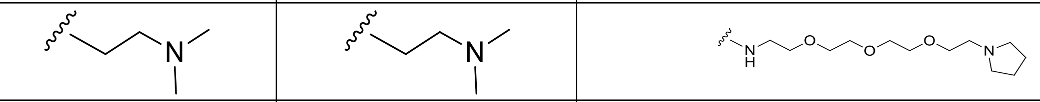			28
**77**	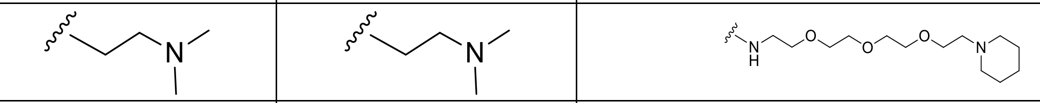			28
**78**	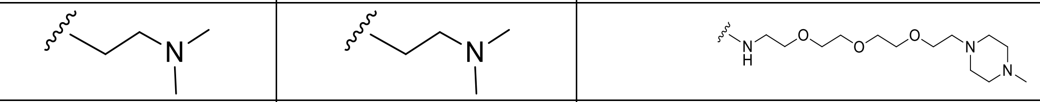			28
**79**	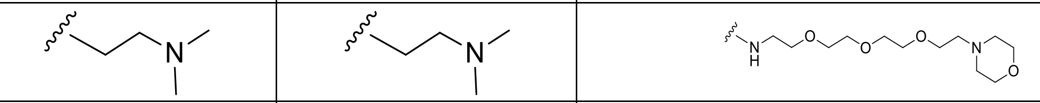			28
**80**	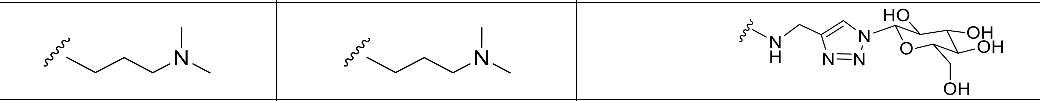			15,16
**81**	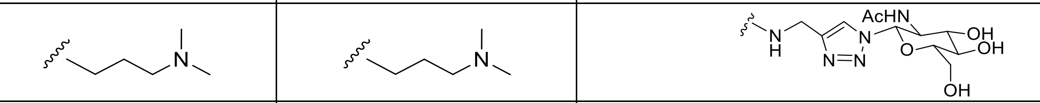			15,16
**82**	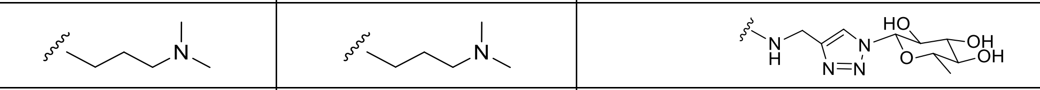			15,16
**83**	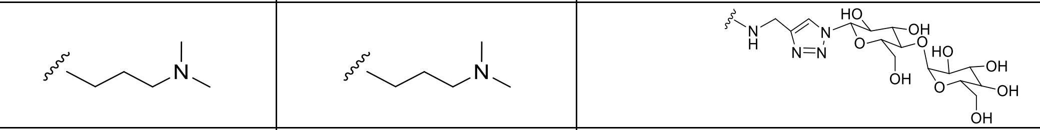			15,16
**84**	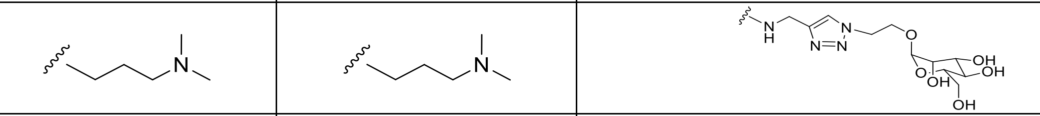			15,16
**85**	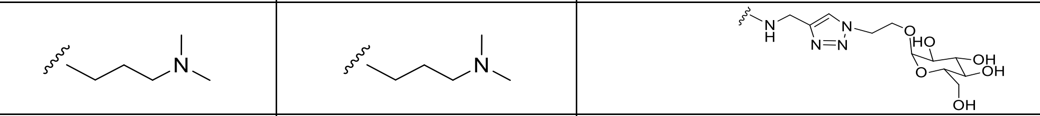			15,16
**86**	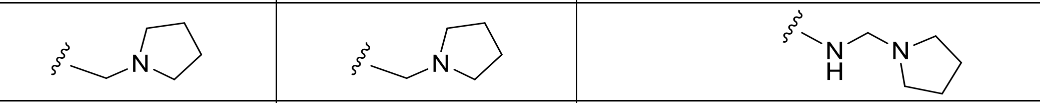			2
**87**	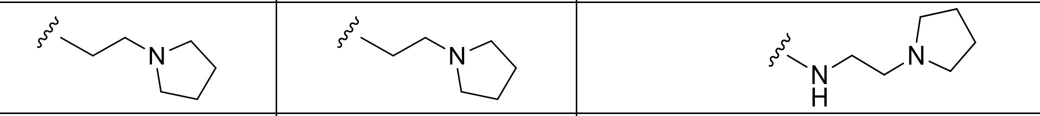			1
**88**	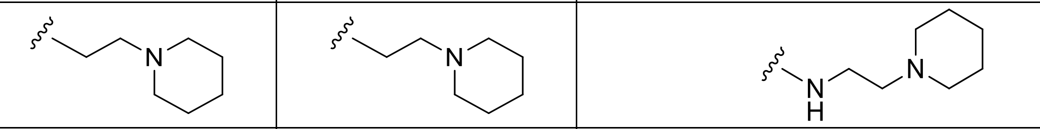			1,2
**89**	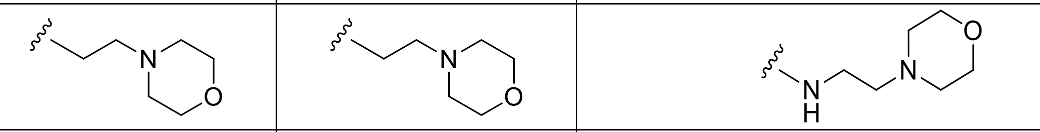			1,2
**90**	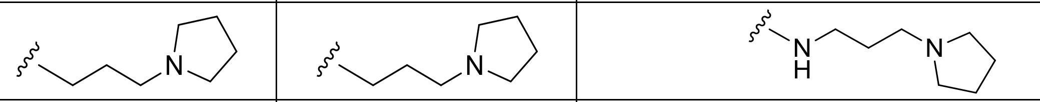			1,2
**91**	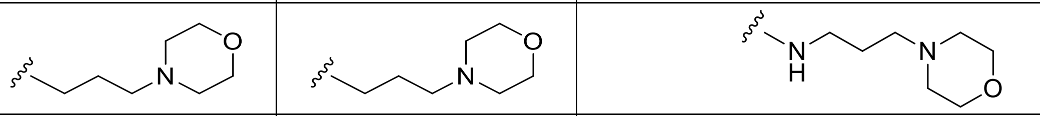			1,2
**92**	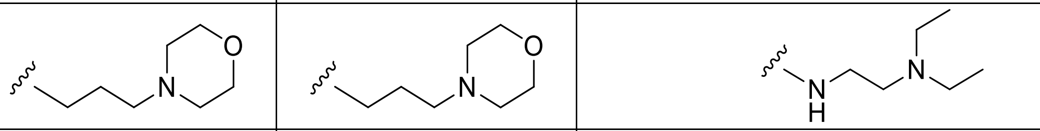			29
**93**	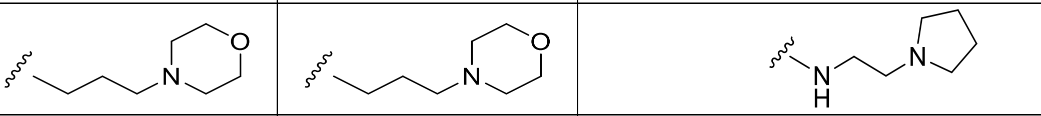			29–32
**94**	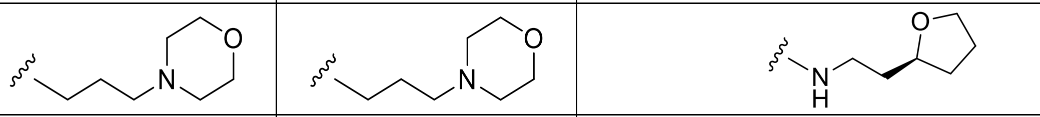			29
**95**	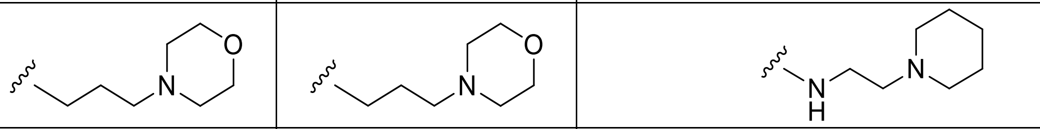			29
**96**	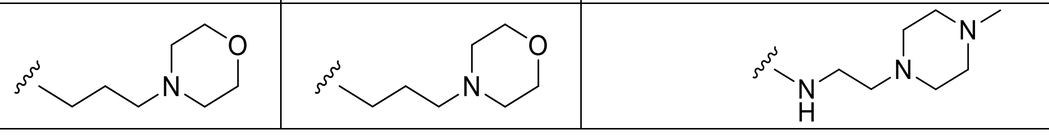			29

**97**	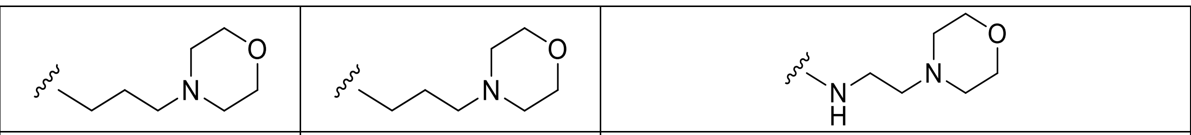			29
**98**	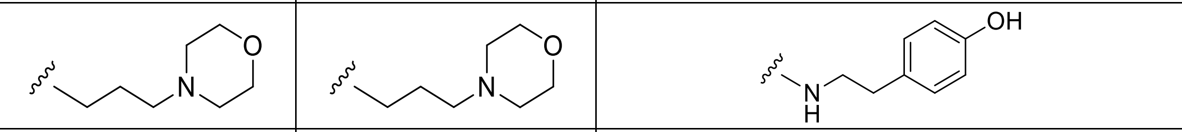			29
**99**	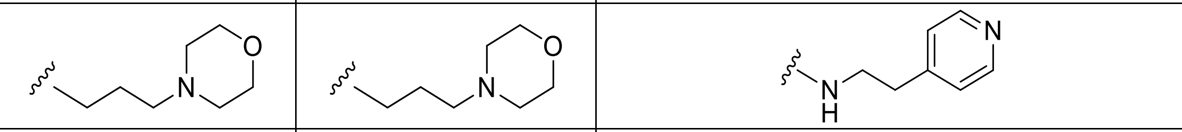			29
**100**	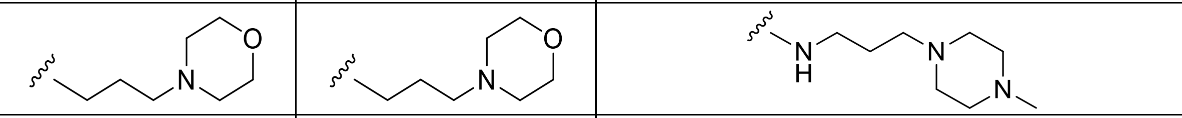			29
**101**	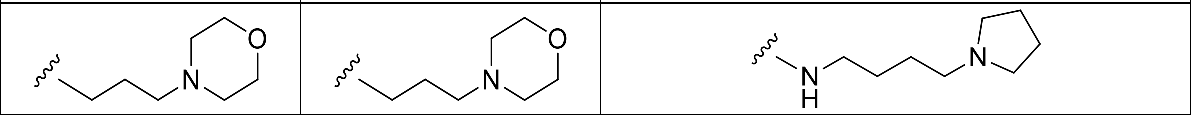			29

Tetrasubstituted NDIs

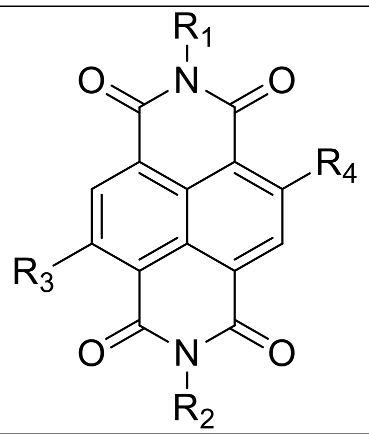

Compound	R1	R2	R3	R4	Refs.
**102**	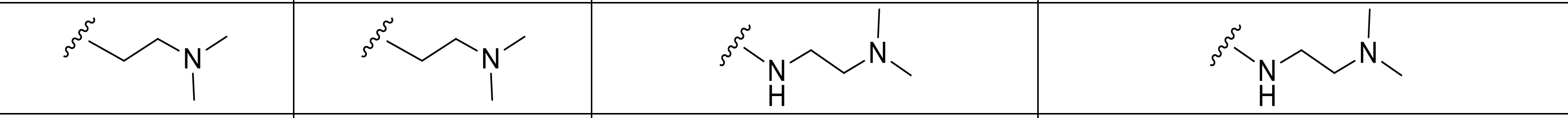				1,2
**103**	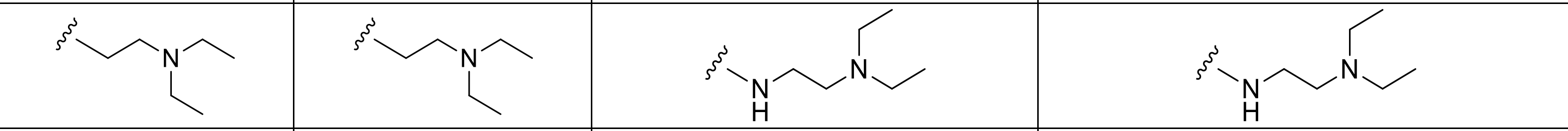				1,2
**104**	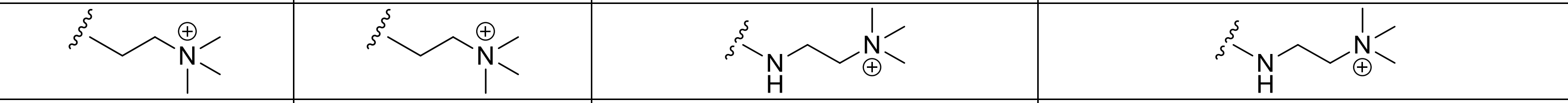				33
**105**	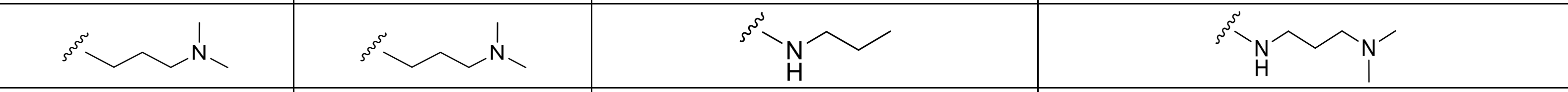				17
**106**	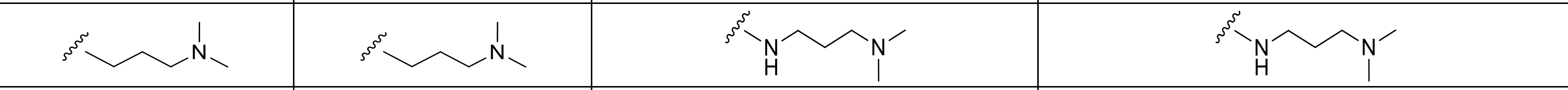				1,2,19
**107**	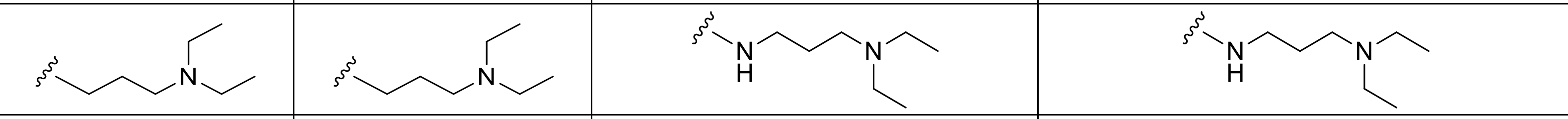				1,2
**108**	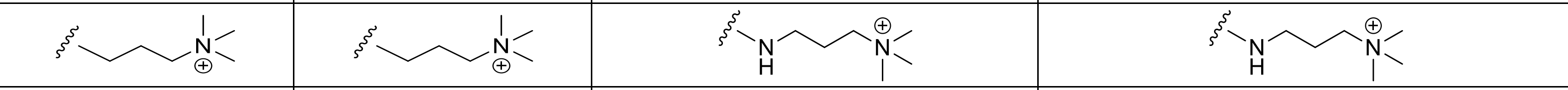				19,33
**109**	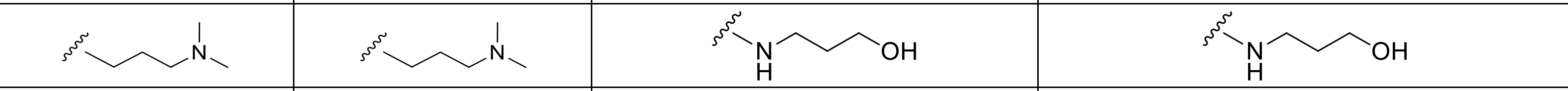				1,2,34,35
**110**	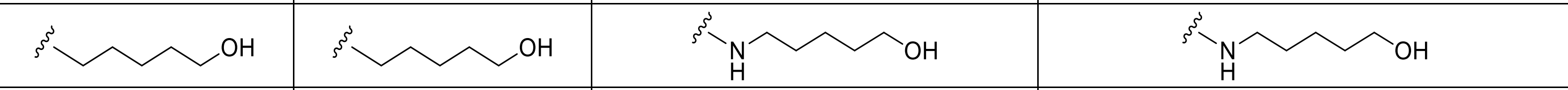				1,2
**111**	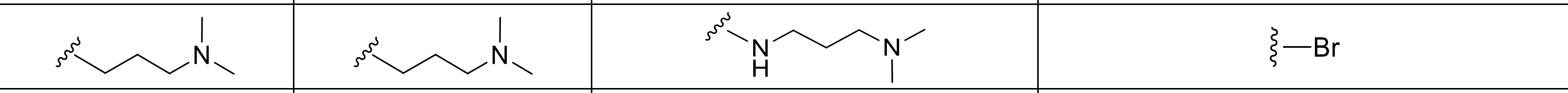				19
**112**	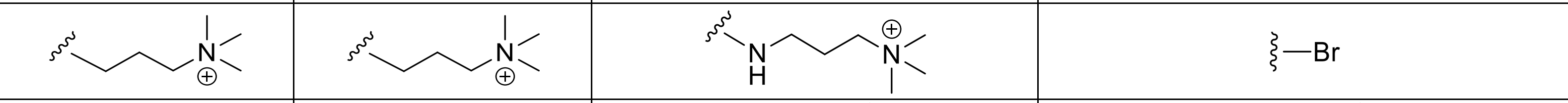				19
**113**	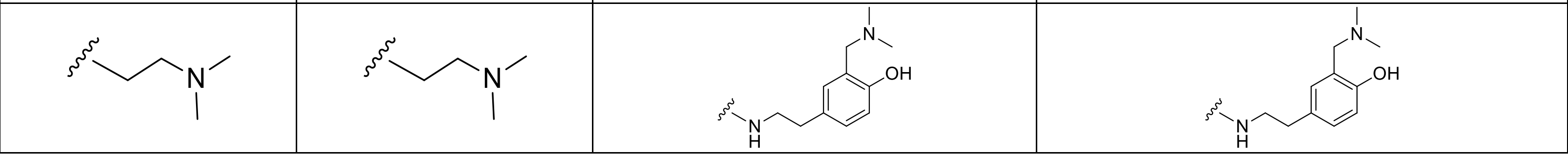				22,23

**114**	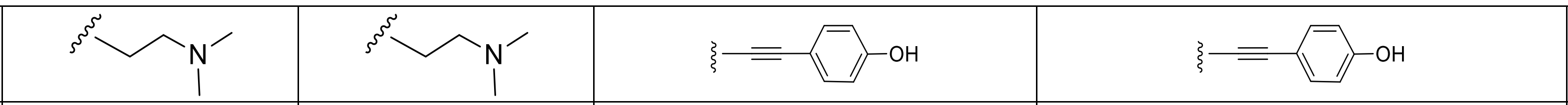	27
**115**	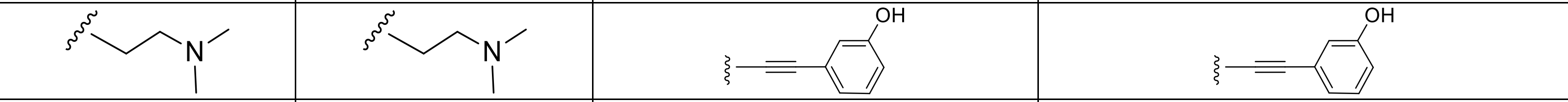	27
**116**	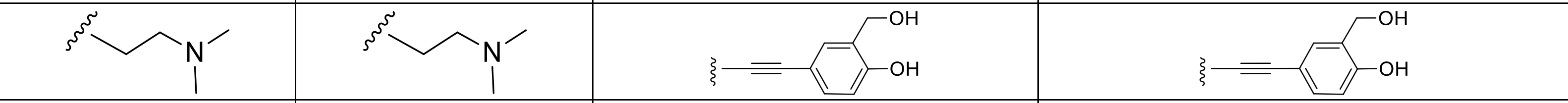	27
**117**	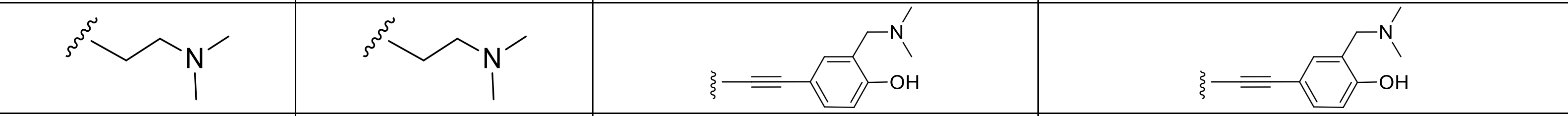	27
**118**	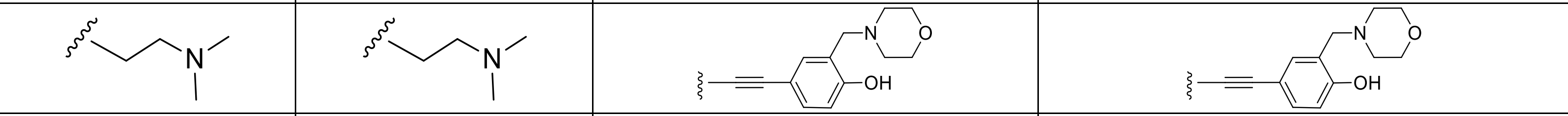	27
**119**	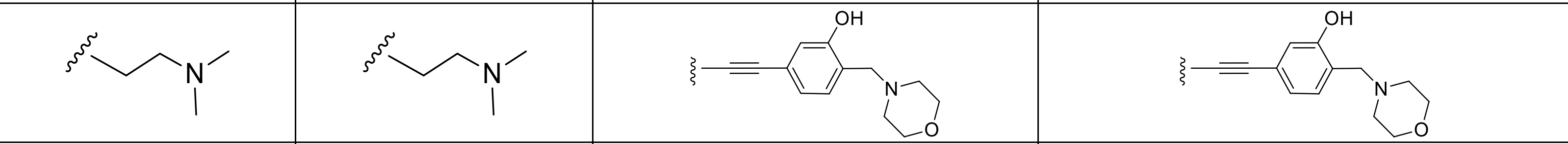	27
**120**	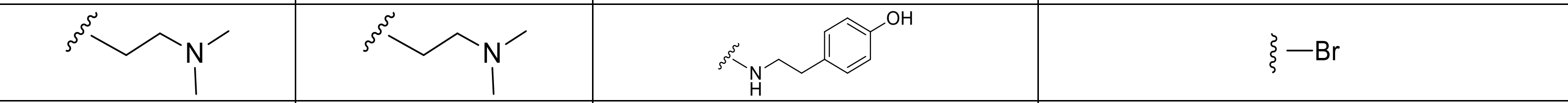	23
**121**	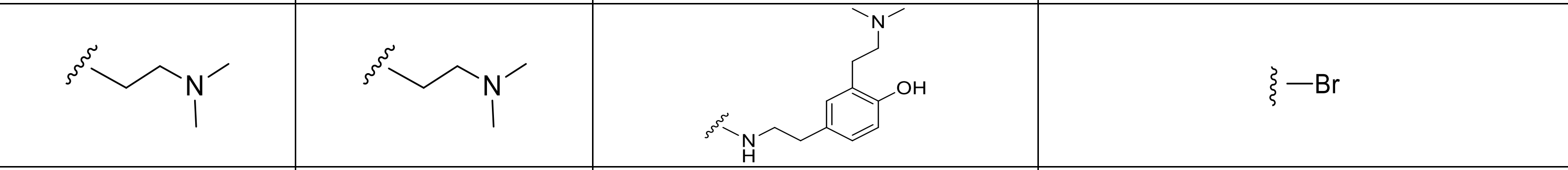	23
**122**	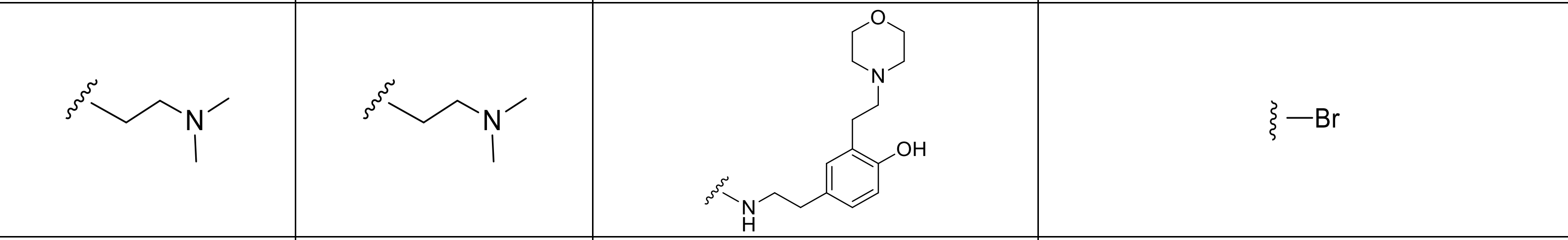	23
**123**	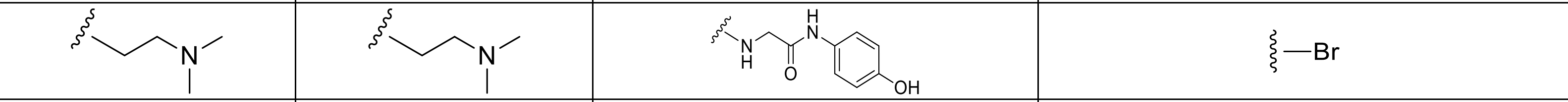	26
**124**	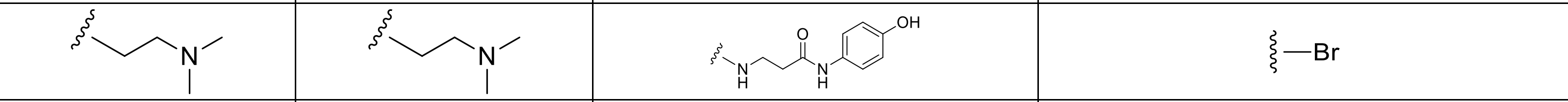	26
**125**	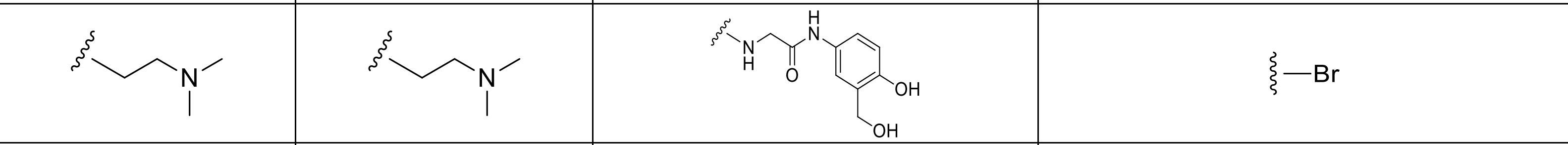	26
**126**	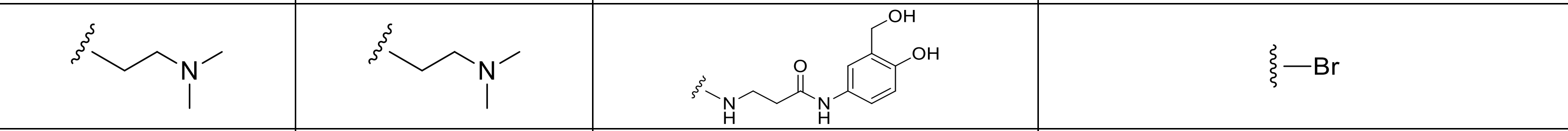	26
**127**	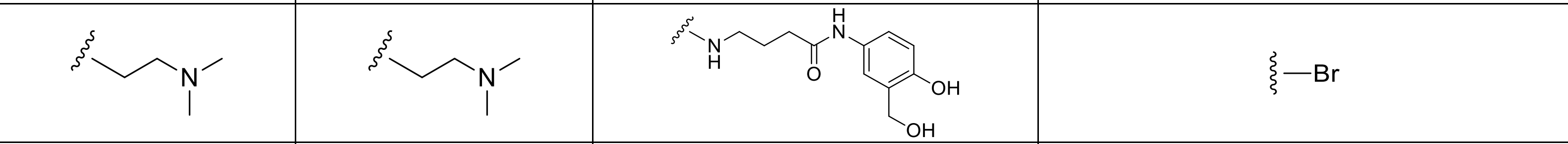	26
**128**	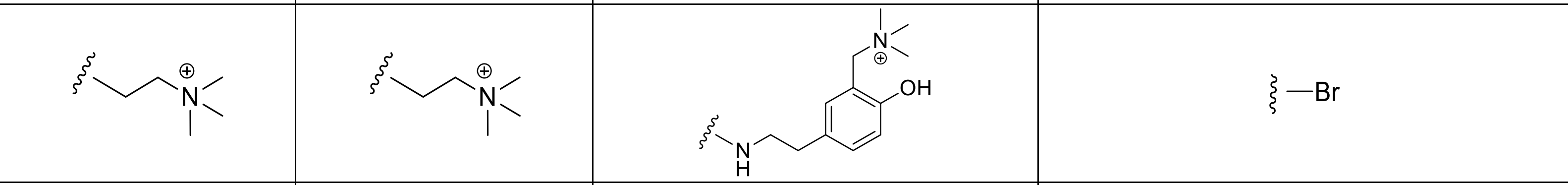	23
**129**	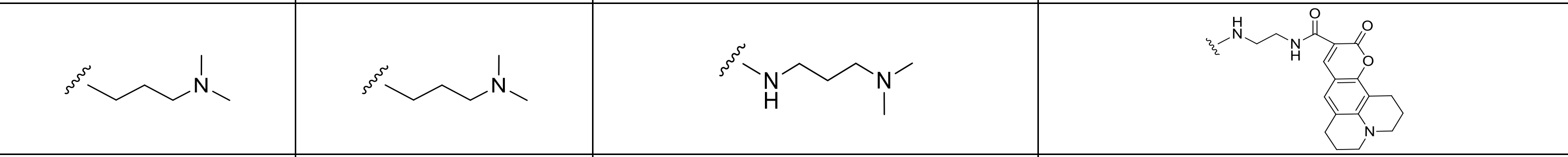	36
**130**	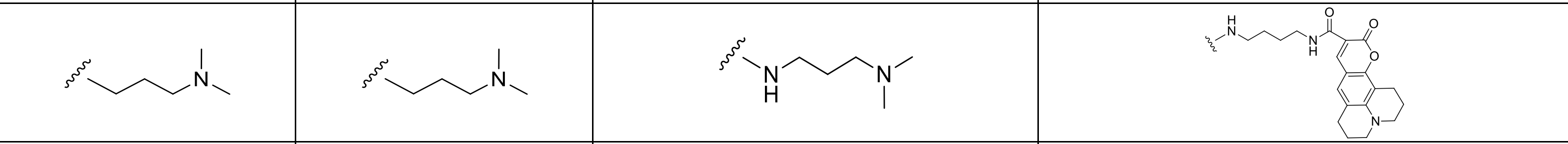	36
**131**	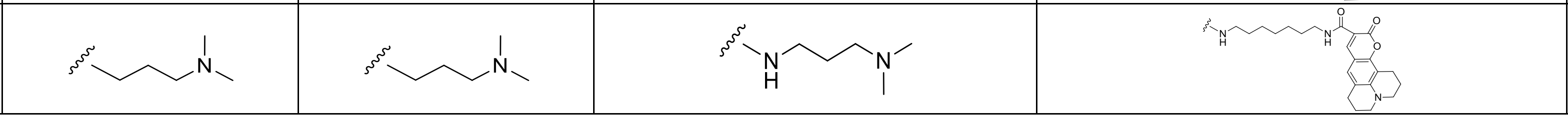	36

**132**	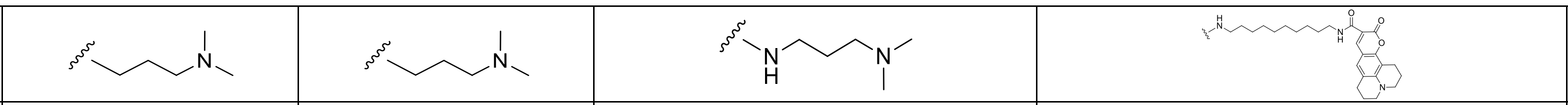	36
**133**	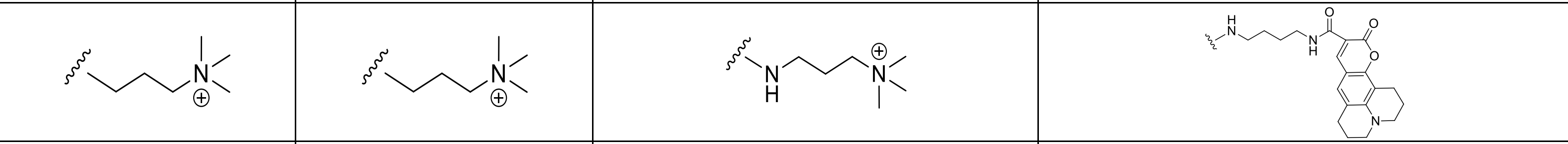	36
**134**	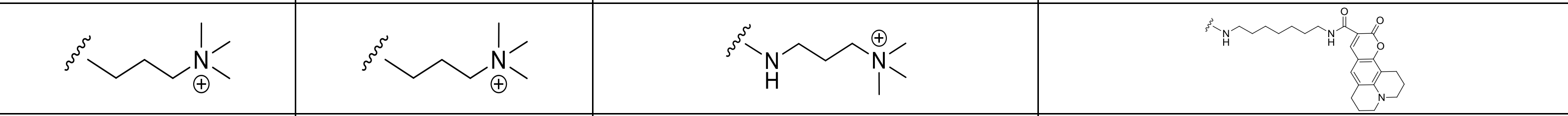	36
**135**	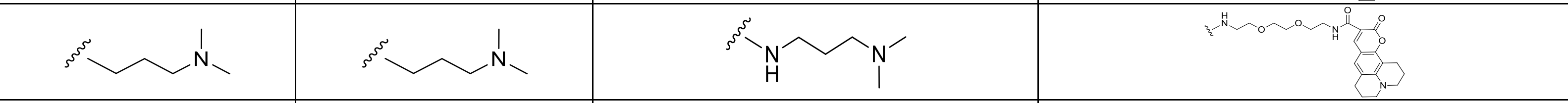	36
**136**	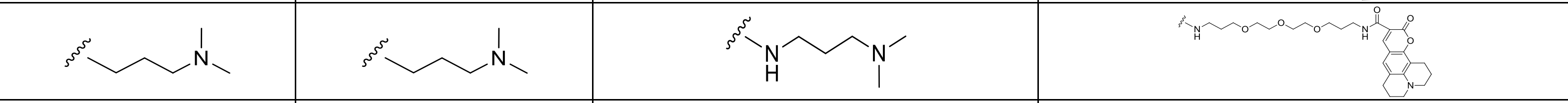	36
**137**	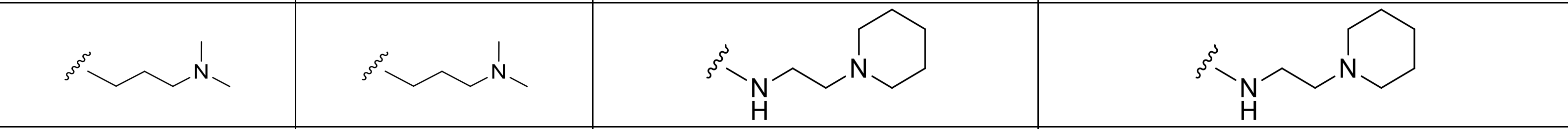	1,2
**138**	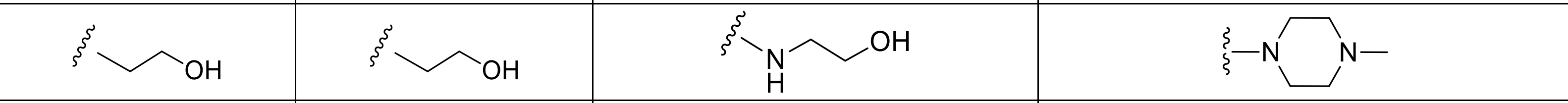	18
**139**	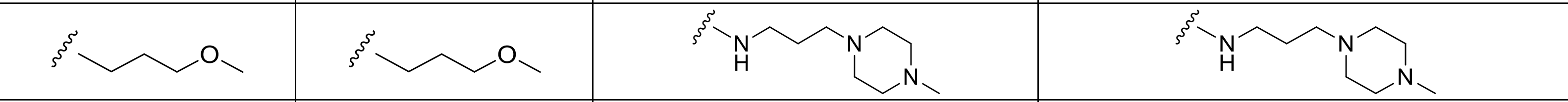	37–39
**140**	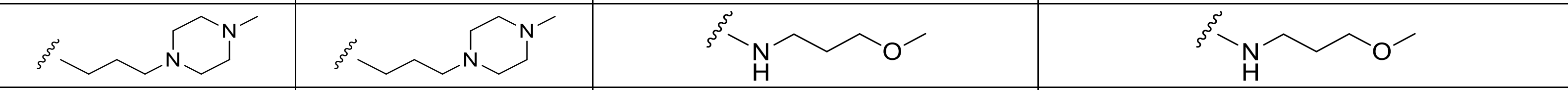	37,39
**141**	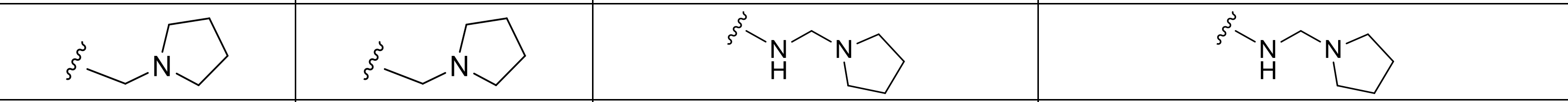	2
**142**	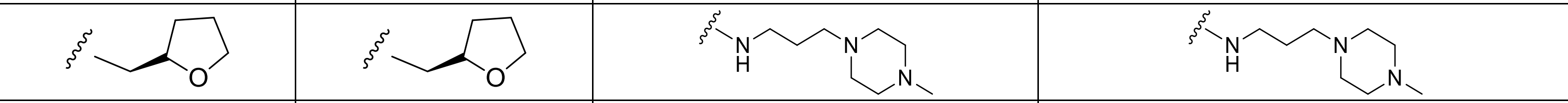	37–39
**143**	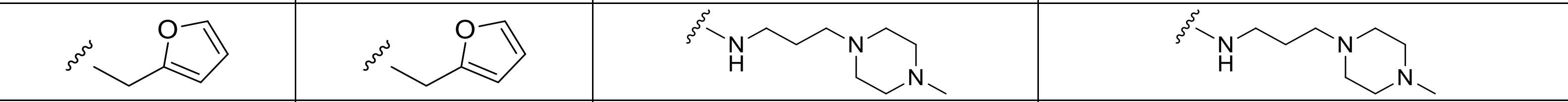	38,39
**144**	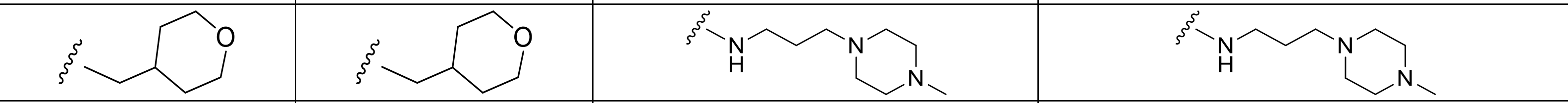	37–39
**145**	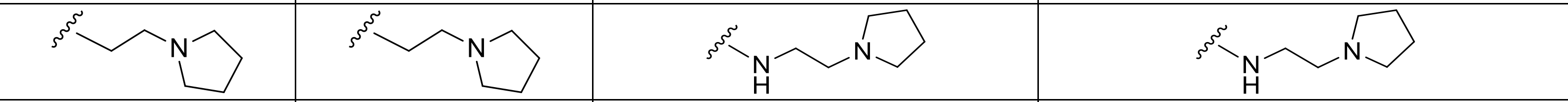	1,40,41
**146**	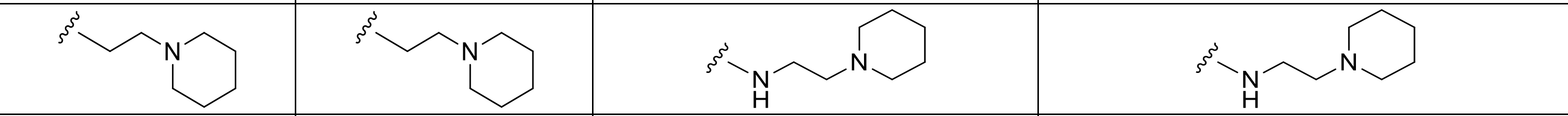	1,2
**147**	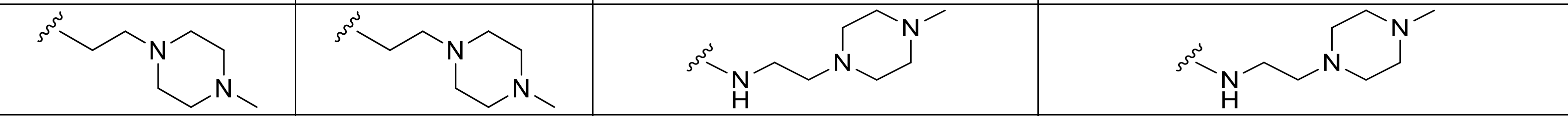	42
**148**	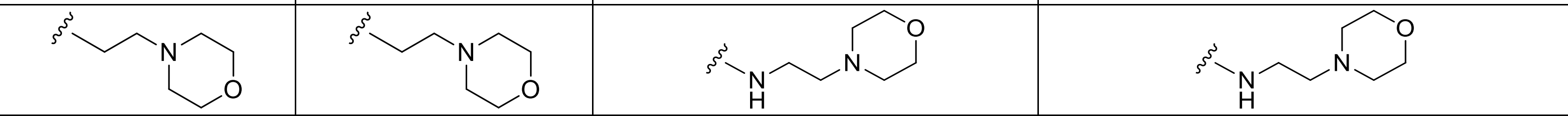	1,2
**149**	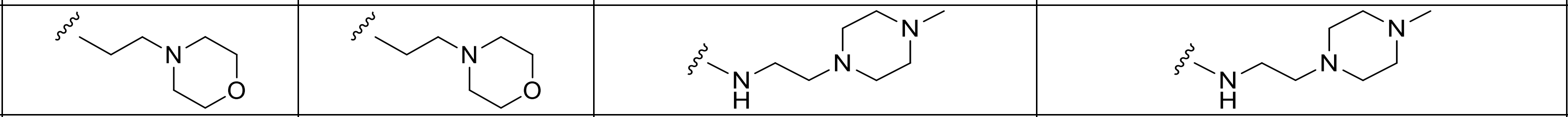	38,39
**150**	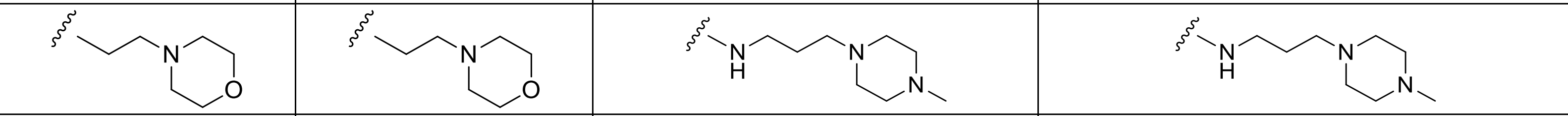	37–39
**151**	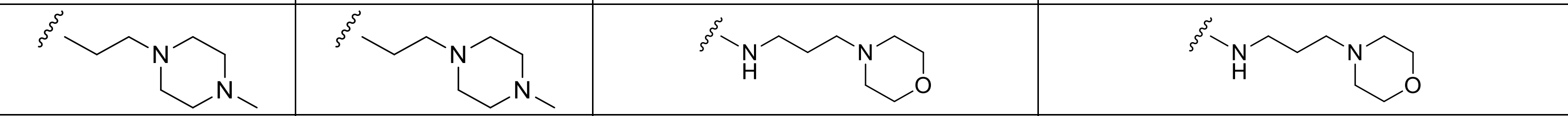	37,39
**152**	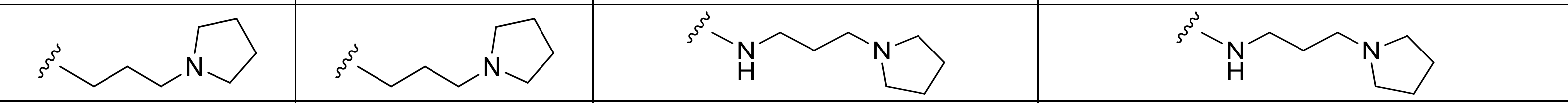	1,2
**153**	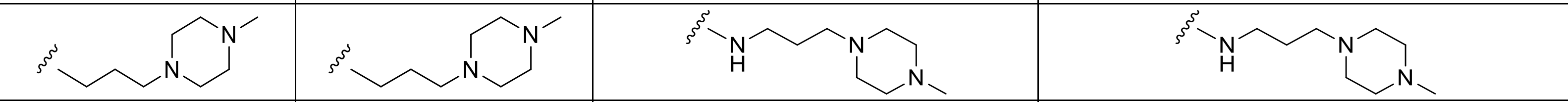	13,38,39,42–45
**154**	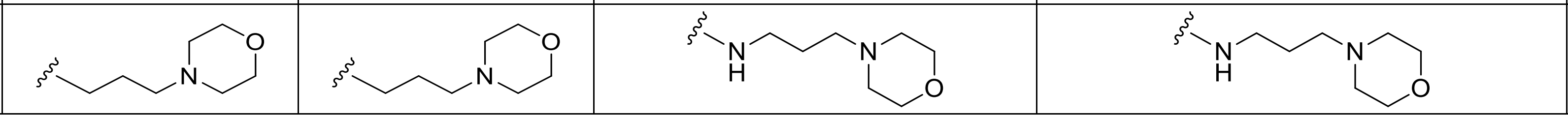	1,2

**155**	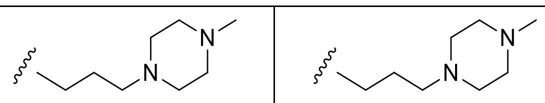	(see R1 column)	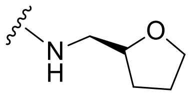	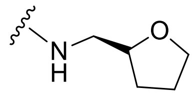	37,39
**156**	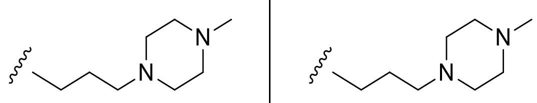	(see R1 column)	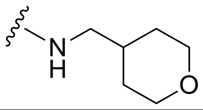	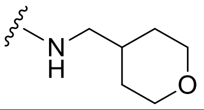	37,39
**157**	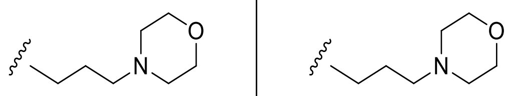	(see R1 column)	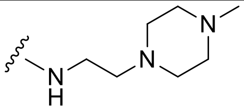	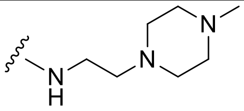	37–39
**158**	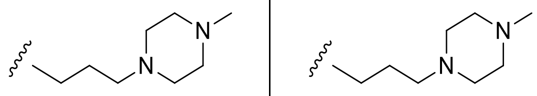	(see R1 column)	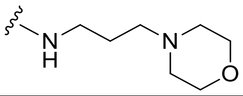	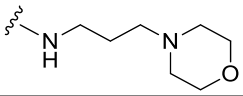	37,39
**159**	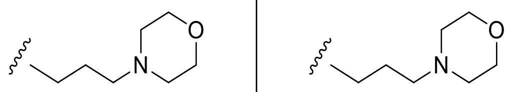	(see R1 column)	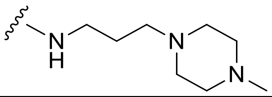	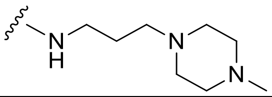	29–31,37–39,46
**160**	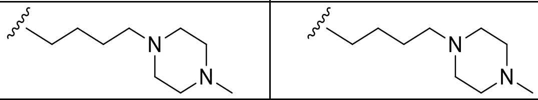	(see R1 column)	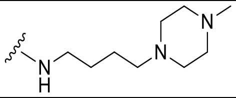	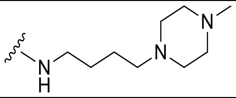	42,43
**161**	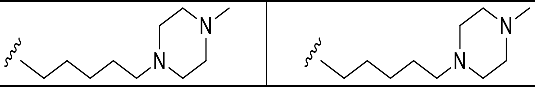	(see R1 column)	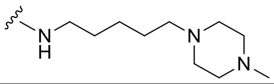	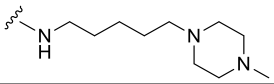	42,43
**162**	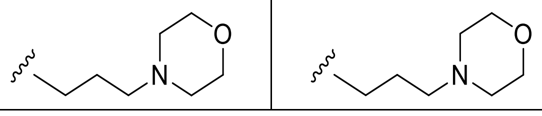	(see R1 column)	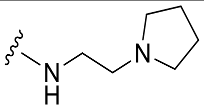	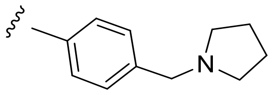	31,47

Core-extended NDIs

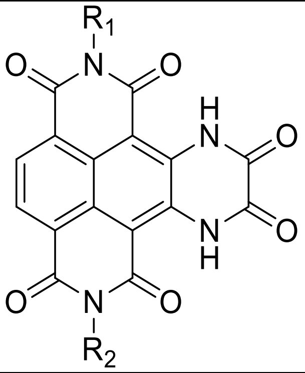

Compound	R1	R2	Refs.
**163**	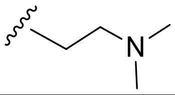	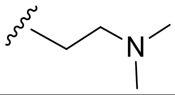	48
**164**	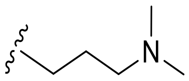	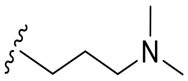	18

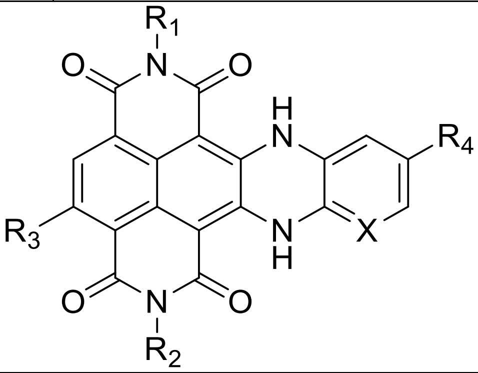

Compound	R1	R2	R3	R4	X	Refs.
**165**	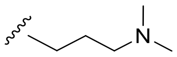	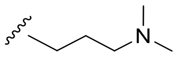	—H	—H	CH	18,49
**166**	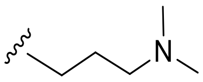	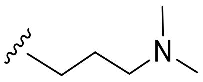	—H	—NO_2	CH	49
**167**	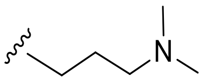	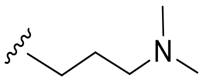	—H	—COOH	CH	49
**168**	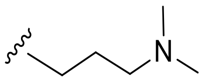	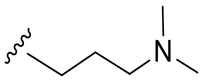	—Br	—H	CH	49
**169**	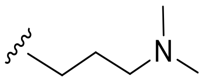	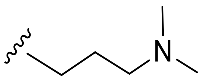	—Br	—NO_2	CH	49

**170**	N	N	—Br	—COOH	CH	49
**171**	N	N	—Br	—Cl	CH	49
**172**	N	N	—Cl	—H	CH	49
**173**	N	N	—H	O, HN, N, O	CH	49
**174**	N	N	—Br	O, HN, N, O	CH	49
**175**	N	N	HN, N	—H	CH	49,50
**176**	N	N	—Br	—H	N	49
**177**	⊕N	⊕N	—H	—H	CH	49
**178**	⊕N	⊕N	—Br	—H	CH	49
**179**	O, OH	O, OH	HN, O, OH	—H	CH	50

Dimeric NDIs

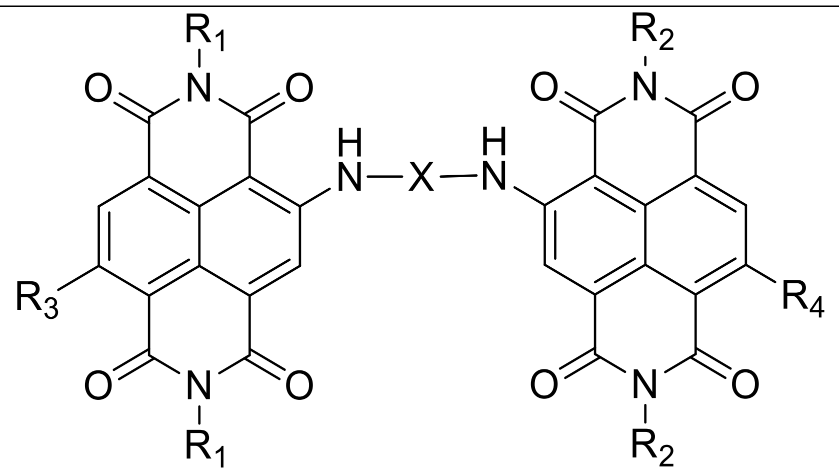

Compound	R1	R2	R3	R4	X	Refs.
**180**	OH, O	N	—H	—H	O, O	17
**181**	OH, O	N	—H	—Br	O, O	17
**182**	OH, O	N	—Br	—H	O, O	17
**183**	OH, O	N	—Br	—Br	O, O	17
**184**	OH, O	N	—H	HN, N	O, O	17
**185**	N	N	—H	—H		19
**186**	N	N	—H	—H		18,19
**187**	N	N	—H	—Br		19
**188**	N	N	—H	—Br		19
**189**	N	N	—H	HN, N		19

**190**	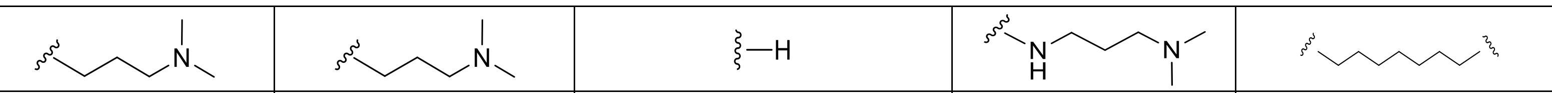					19
**191**	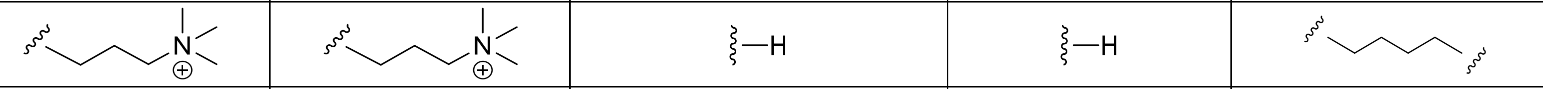					19
**192**	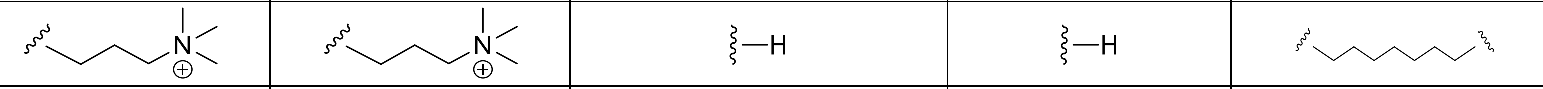					19
**193**	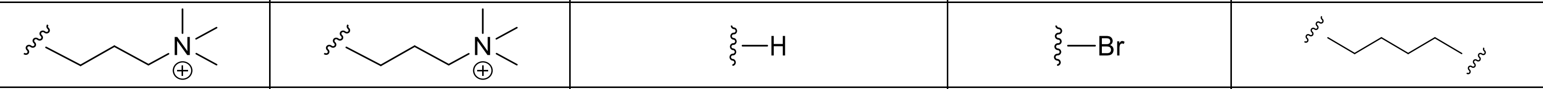					19
**194**	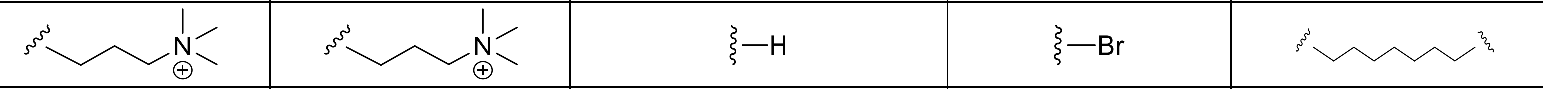					19
**195**	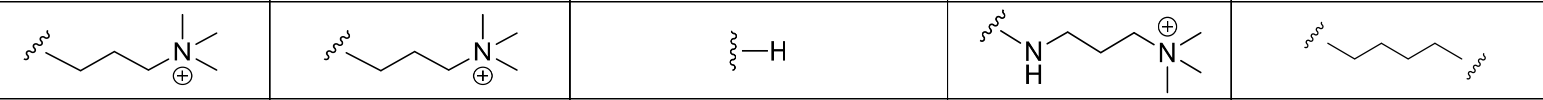					19
**196**	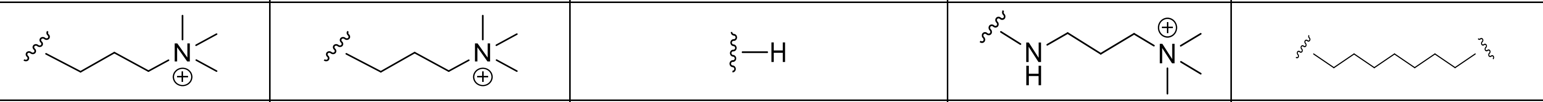					19
**197**	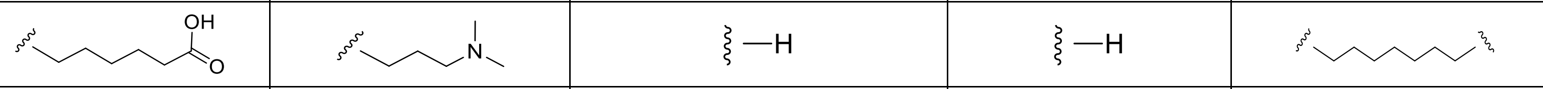					17
**198**	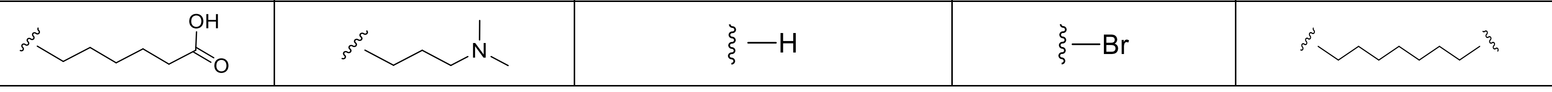					17

Cyclic NDIs

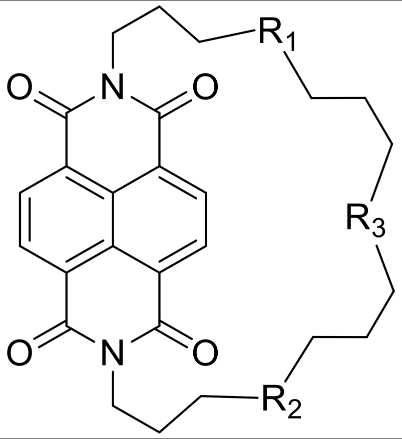

Compound	R1	R2	R3	Refs.
**199**	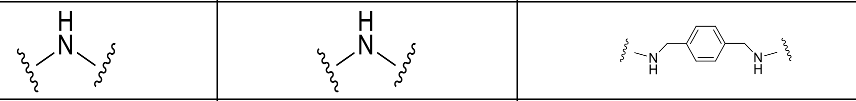			11
**200**	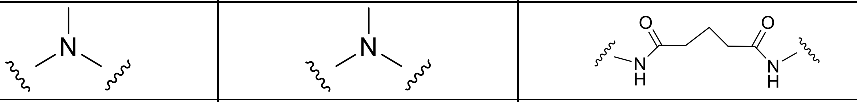			51
**201**	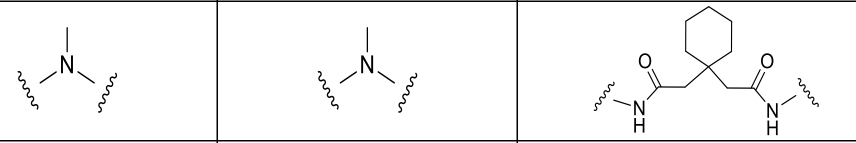			4,51
**202**	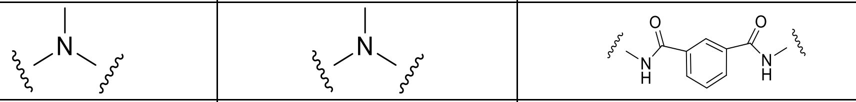			51
**203**	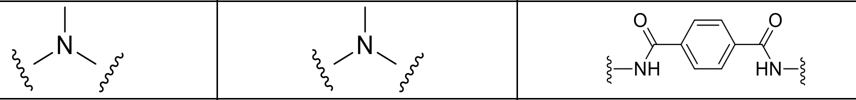			5
**204**	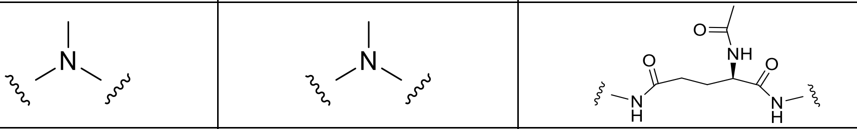			52
**205**	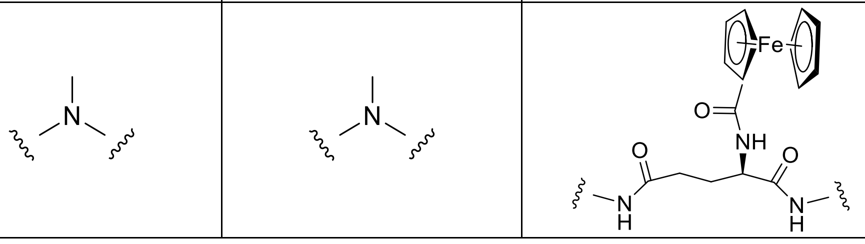			53

**206**	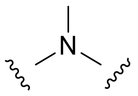	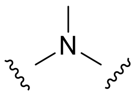	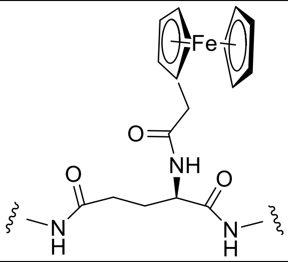	53
**207**	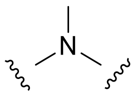	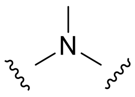	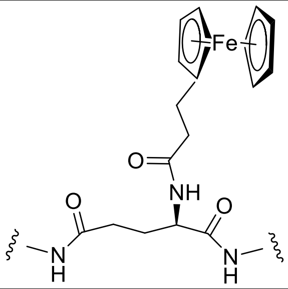	53
**208**	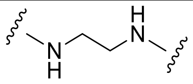	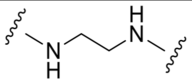	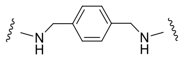	11
**209**	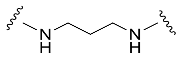	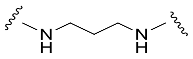	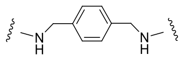	11
**210**	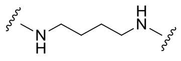	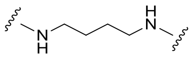	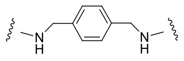	11
**211**	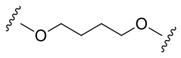	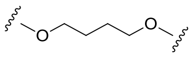	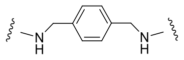	11
**212**	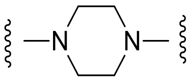	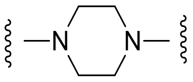	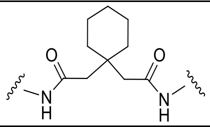	4
**213**	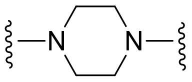	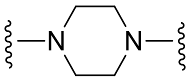	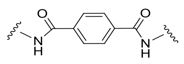	3,5

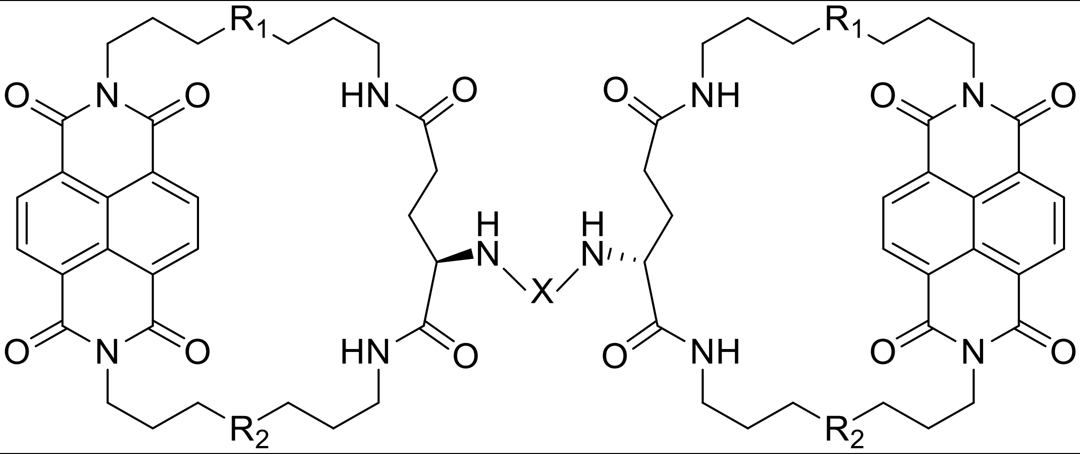

**Compound**	**R1**	**R2**	**X**	**Refs.**
**214**	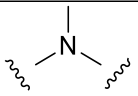	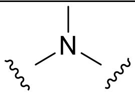	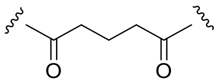	52
**215**	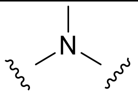	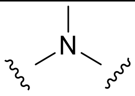	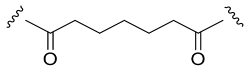	52
**216**	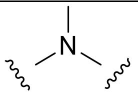	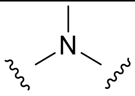	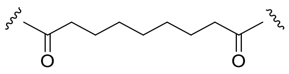	52

Table S2. Summary of the differences between the melting temperatures of each DNA/NDI complex and those of the related free DNA target (ΔT_m) obtained by biophysical techniques for the indicated DNA/NDI systems. n.d. = not defined; FRET = fluorescence resonance energy transfer; CD = circular dichroism. In order to maintain information about the G-quadruplex over duplex selectivity evaluated by thermal stability data, ΔT_m values equal to 0.0 were considered equal to 0.01.

Compound	G-quadruplex			Duplex			Experimental conditions ([DNA], [NDI], buffer composition)	ΔT_m (G-quadruplex)/ ΔT_m (duplex)	Refs.
	ΔT_m (°C)	DNA target	Technique	ΔT_m (°C)	DNA target	Technique			
**1**	3.2	Telomere	FRET	1.0	T-loop	FRET	0.20 µM, 0.50 µM, 50 mM potassium cacodylate, pH 7.4	3.2	1,2
**1**	1.2	*c-kit1*	FRET	1.0	T-loop	FRET	0.20 µM, 0.50 µM, 50 mM potassium cacodylate, pH 7.4	1.2	2
**1**	7.2	*c-kit2*	FRET	1.0	T-loop	FRET	0.20 µM, 0.50 µM, 50 mM potassium cacodylate, pH 7.4	7.2	2
**2**	5.2	Telomere	FRET	2.7	dsDNA	FRET	0.20 µM, 0.50 µM, 50 mM potassium cacodylate, pH 7.4	1.9	1,2
**2**	6.0	Telomere	CD	5.0	dsDNA	UV	Telomere: 1.5 µM, 3.0 µM, 100 mM KCl, 50 mM Tris-HCl, pH 7.4<br>dsDNA: 3.2 µM, 6.4 µM, 100 mM KCl, 50 mM Tris-HCl, pH 7.4	1.2	3
**2**	2.5	*c-kit1*	FRET	2.7	T-loop	FRET	0.20 µM, 0.5 µM, 50 mM potassium cacodylate, pH 7.4	0.93	2
**2**	7.8	*c-kit2*	FRET	2.7	T-loop	FRET	0.20 µM, 0.5 µM, 50 mM potassium cacodylate, pH 7.4	2.9	2
**3**	2.4	Telomere	FRET	1.0	dsDNA	FRET	0.25 µM, 0.75 µM, 50 mM KCl, 40 mM Li_3PO_4, pH 7.5	2.4	7
**3**	6.3	Telomere	FRET	3.3	dsDNA	FRET	0.25 µM, 2.5 µM, 50 mM KCl, 40 mM Li_3PO_4, pH 7.5	1.9	7
**4**	4.4	Telomere	FRET	2.0	dsDNA	FRET	0.25 µM, 0.75 µM, 50 mM KCl, 40 mM Li_3PO_4, pH 7.5	2.2	7
**4**	7.8	Telomere	FRET	3.4	dsDNA	FRET	0.25 µM, 2.5 µM, 50 mM KCl, 40 mM Li_3PO_4, pH 7.5	2.3	7
**5**	9.9	Telomere	FRET	5.0	dsDNA	FRET	0.25 µM, 0.75 µM, 50 mM KCl, 40 mM Li_3PO_4, pH 7.5	2.0	7
**5**	14.5	Telomere	FRET	8.5	dsDNA	FRET	0.25 µM, 2.5 µM, 50 mM KCl, 40 mM Li_3PO_4, pH 7.5	1.7	7
**6**	4.5	Telomere	FRET	3.5	dsDNA	FRET	0.25 µM, 0.75 µM, 50 mM KCl, 40 mM Li_3PO_4, pH 7.5	1.3	7
**6**	10.9	Telomere	FRET	8.1	dsDNA	FRET	0.25 µM, 2.5 µM, 50 mM KCl, 40 mM Li_3PO_4, pH 7.5	1.3	7
**7**	13.3	Telomere	FRET	7.5	dsDNA	FRET	0.25 µM, 0.75 µM, 50 mM KCl, 40 mM Li_3PO_4, pH 7.5	1.8	7
**7**	20.4	Telomere	FRET	14.0	dsDNA	FRET	0.25 µM, 2.5 µM, 50 mM KCl, 40 mM Li_3PO_4, pH 7.5	1.5	7
**8**	10.2	*c-kit2*	CD	1.9	dsDNA	CD	10 µM, 100 µM, 100 mM KF, 10 mM potassium phosphate, pH 7.4	5.4	8
**8**	1.7	*c-kit1*	CD	1.9	dsDNA	CD	10 µM, 100 µM, 100 mM KF, 10 mM potassium phosphate, pH 7.4	0.89	8
**8**	9.2	*c-myc*	CD	1.9	dsDNA	CD	10 µM, 100 µM, 100 mM KF, 10 mM potassium phosphate, pH 7.4	4.8	8
**8**	2.3	*kRAS*	CD	1.9	dsDNA	CD	10 µM, 100 µM, 100 mM KF, 10 mM potassium phosphate, pH 7.4	1.2	8
**8**	-0.7	Telomere	CD	1.9	dsDNA	CD	10 µM, 100 µM, 100 mM KF, 10 mM potassium phosphate, pH 7.4	-0.4	8
**9**	10.5	*c-kit2*	CD	0.7	dsDNA	CD	10 µM, 100 µM, 100 mM KF, 10 mM potassium phosphate, pH 7.4	15	8
**9**	3.0	*c-kit1*	CD	0.7	dsDNA	CD	10 µM, 100 µM, 100 mM KF, 10 mM potassium phosphate, pH 7.4	4.3	8
**9**	11.1	*c-myc*	CD	0.7	dsDNA	CD	10 µM, 100 µM, 100 mM KF,	16	8

							10 mM potassium phosphate, pH 7.4		
**9**	0.4	*kRAS*	CD	0.7	dsDNA	CD	10 µM, 100 µM, 100 mM KF,<br>10 mM potassium phosphate, pH 7.4	0.6	8
**9**	-1.1	Telomere	CD	0.7	dsDNA	CD	10 µM, 100 µM, 100 mM KF,<br>10 mM potassium phosphate, pH 7.4	-1.6	8
**10**	14.6	*c-kit2*	CD	-2.5	dsDNA	CD	10 µM, 100 µM, 100 mM KF,<br>10 mM potassium phosphate, pH 7.4	-5.8	8
**10**	-1.5	*c-kit1*	CD	-2.5	dsDNA	CD	10 µM, 100 µM, 100 mM KF,<br>10 mM potassium phosphate, pH 7.4	0.60	8
**10**	0.1	*c-myc*	CD	-2.5	dsDNA	CD	10 µM, 100 µM, 100 mM KF,<br>10 mM potassium phosphate, pH 7.4	0.040	8
**10**	1.7	*kRAS*	CD	-2.5	dsDNA	CD	10 µM, 100 µM, 100 mM KF,<br>10 mM potassium phosphate, pH 7.4	-0.68	8
**10**	-1.1	Telomere	CD	-2.5	dsDNA	CD	10 µM, 100 µM, 100 mM KF,<br>10 mM potassium phosphate, pH 7.4	0.44	8
**11**	-3.1	*c-kit2*	CD	1.8	dsDNA	CD	10 µM, 100 µM, 100 mM KF,<br>10 mM potassium phosphate, pH 7.4	-1.7	8
**11**	-0.2	*c-kit1*	CD	1.8	dsDNA	CD	10 µM, 100 µM, 100 mM KF,<br>10 mM potassium phosphate, pH 7.4	-0.11	8
**11**	2.2	*c-myc*	CD	1.8	dsDNA	CD	10 µM, 100 µM, 100 mM KF,<br>10 mM potassium phosphate, pH 7.4	1.2	8
**11**	4.1	*kRAS*	CD	1.8	dsDNA	CD	10 µM, 100 µM, 100 mM KF,<br>10 mM potassium phosphate, pH 7.4	2.3	8
**11**	2.0	Telomere	CD	1.8	dsDNA	CD	10 µM, 100 µM, 100 mM KF,<br>10 mM potassium phosphate, pH 7.4	1.1	8
**12**	-5.9	*c-kit2*	CD	-2.7	dsDNA	CD	10 µM, 100 µM, 100 mM KF,<br>10 mM potassium phosphate, pH 7.4	2.2	8
**12**	0.5	*c-kit1*	CD	-2.7	dsDNA	CD	10 µM, 100 µM, 100 mM KF,<br>10 mM potassium phosphate, pH 7.4	-0.18	8
**12**	0.2	*c-myc*	CD	-2.7	dsDNA	CD	10 µM, 100 µM, 100 mM KF,<br>10 mM potassium phosphate, pH 7.4	-0.074	8
**12**	1.8	*kRAS*	CD	-2.7	dsDNA	CD	10 µM, 100 µM, 100 mM KF,<br>10 mM potassium phosphate, pH 7.4	-0.67	8
**12**	0.3	Telomere	CD	-2.7	dsDNA	CD	10 µM, 100 µM, 100 mM KF,<br>10 mM potassium phosphate, pH 7.4	-0.11	8
**13**	1.4	*c-kit2*	CD	0.5	dsDNA	CD	10 µM, 100 µM, 100 mM KF,<br>10 mM potassium phosphate, pH 7.4	2.8	9
**13**	1.7	*c-kit1*	CD	0.5	dsDNA	CD	10 µM, 100 µM, 100 mM KF,<br>10 mM potassium phosphate, pH 7.4	3.4	9
**13**	-1.1	*kRAS*	CD	0.5	dsDNA	CD	10 µM, 100 µM, 100 mM KF,<br>10 mM potassium phosphate, pH 7.4	-2.2	9
**13**	5.9	*bcl-2*	CD	0.5	dsDNA	CD	10 µM, 100 µM, 100 mM KF,<br>10 mM potassium phosphate, pH 7.4	12	9
**13**	1.3	Telomere	CD	0.5	dsDNA	CD	10 µM, 100 µM, 100 mM KF,<br>10 mM potassium phosphate, pH 7.4	2.6	9
**14**	2.3	*c-kit2*	CD	-4.9	dsDNA	CD	10 µM, 100 µM, 100 mM KF,<br>10 mM potassium phosphate, pH 7.4	-0.47	9
**14**	3.9	*c-kit1*	CD	-4.9	dsDNA	CD	10 µM, 100 µM, 100 mM KF,<br>10 mM potassium phosphate, pH 7.4	-0.80	9
**14**	4.8	*kRAS*	CD	-4.9	dsDNA	CD	10 µM, 100 µM, 100 mM KF,<br>10 mM potassium phosphate, pH 7.4	-1.0	9
**14**	6.7	*bcl-2*	CD	-4.9	dsDNA	CD	10 µM, 100 µM, 100 mM KF,<br>10 mM potassium phosphate, pH 7.4	-1.4	9
**14**	-0.6	Telomere	CD	-4.9	dsDNA	CD	10 µM, 100 µM, 100 mM KF,<br>10 mM potassium phosphate, pH 7.4	0.12	9
**15**	-1.6	*c-kit2*	CD	-1.8	dsDNA	CD	10 µM, 100 µM, 100 mM KF,<br>10 mM potassium phosphate, pH 7.4	0.89	9
**15**	1.5	*c-kit1*	CD	-1.8	dsDNA	CD	10 µM, 100 µM, 100 mM KF,<br>10 mM potassium phosphate, pH 7.4	-0.83	9
**15**	-2.0	*kRAS*	CD	-1.8	dsDNA	CD	10 µM, 100 µM, 100 mM KF,<br>10 mM potassium phosphate, pH 7.4	1.1	9
**15**	2.4	*bcl-2*	CD	-1.8	dsDNA	CD	10 µM, 100 µM, 100 mM KF,<br>10 mM potassium phosphate, pH 7.4	-1.3	9
**15**	-2.6	Telomere	CD	-1.8	dsDNA	CD	10 µM, 100 µM, 100 mM KF,	1.4	9

							10 mM potassium phosphate, pH 7.4		
**16**	-2.1	*c-kit2*	CD	-1.9	dsDNA	CD	10 µM, 100 µM, 100 mM KF,<br>10 mM potassium phosphate, pH 7.4	1.1	9
**16**	1.0	*c-kit1*	CD	-1.9	dsDNA	CD	10 µM, 100 µM, 100 mM KF,<br>10 mM potassium phosphate, pH 7.4	-0.53	9
**16**	-1.2	*kRAS*	CD	-1.9	dsDNA	CD	10 µM, 100 µM, 100 mM KF,<br>10 mM potassium phosphate, pH 7.4	0.63	9
**16**	2.4	*bcl-2*	CD	-1.9	dsDNA	CD	10 µM, 100 µM, 100 mM KF,<br>10 mM potassium phosphate, pH 7.4	-1.3	9
**16**	0.9	Telomere	CD	-1.9	dsDNA	CD	10 µM, 100 µM, 100 mM KF,<br>10 mM potassium phosphate, pH 7.4	-0.47	9
**17**	2.4	Telomere	FRET	0.9	dsDNA	FRET	0.25 µM, 2.5 µM, 50 mM KCl,<br>10 mM LiOH, pH 7.0	2.7	10
**18**	1.4	Telomere	FRET	6.7	dsDNA	FRET	0.25 µM, 2.5 µM, 50 mM KCl,<br>10 mM LiOH, pH 7.0	0.21	10
**19**	13.3	Telomere	FRET	7.0	dsDNA	FRET	0.25 µM, 2.5 µM, 50 mM KCl,<br>10 mM LiOH, pH 7.0	1.9	10
**20**	29.0	Telomere	FRET	12.2	dsDNA	FRET	0.25 µM, 2.5 µM, 50 mM KCl,<br>10 mM LiOH, pH 7.0	2.4	10
**21**	22.2	Telomere	FRET	9.1	dsDNA	FRET	0.25 µM, 2.5 µM, 50 mM KCl,<br>10 mM LiOH, pH 7.0	2.4	10
**22**	18.0	Telomere	FRET	9.5	dsDNA	FRET	0.25 µM, 2.5 µM, 50 mM KCl,<br>10 mM LiOH, pH 7.0	1.9	10
**23**	22.0	Telomere	FRET	10.4	dsDNA	FRET	0.25 µM, 2.5 µM, 50 mM KCl,<br>10 mM LiOH, pH 7.0	2.1	10
**24**	16.3	Telomere	FRET	12.2	dsDNA	FRET	0.25 µM, 2.5 µM, 50 mM KCl,<br>10 mM LiOH, pH 7.0	1.3	10
**25**	1.5	Telomere	FRET	0.6	dsDNA	FRET	0.25 µM, 0.75 µM, 50 mM KCl,<br>40 mM Li_3PO_4, pH 7.5	2.5	7
**25**	21.4	Telomere	FRET	6.7	dsDNA	FRET	0.25 µM, 2.5 µM, 50 mM KCl,<br>40 mM Li_3PO_4, pH 7.5	3.2	7,10
**26**	26.1	Telomere	FRET	17.1	dsDNA	FRET	0.25 µM, 2.5 µM, 50 mM KCl,<br>10 mM LiOH, pH 7.0	1.5	10
**27**	12.9	Telomere	FRET	1.5	dsDNA	FRET	0.25 µM, 2.5 µM, 50 mM KCl,<br>10 mM LiOH, pH 7.0	8.6	10
**28**	13.1	Telomere	FRET	12.6	dsDNA	FRET	0.25 µM, 2.5 µM, 50 mM KCl,<br>10 mM LiOH, pH 7.0	1.0	10
**29**	9.9	Telomere	FRET	2.2	T-loop	FRET	0.20 µM, 1.0 µM, 60 mM potassium<br>cacodylate, pH 7.4	4.5	11
**29**	7.4	*c-kit2*	FRET	2.2	T-loop	FRET	0.20 µM, 1.0 µM, 60 mM potassium<br>cacodylate, pH 7.4	3.4	11
**29**	20.5	Telomere	FRET	8.4	dsDNA	FRET	0.25 µM, 2.5 µM, 50 mM KCl,<br>10 mM LiOH, pH 7.0	2.4	10
**30**	9.8	Telomere	FRET	0.3	F10T	FRET	0.20 µM, 1.0 µM, 10 mM KCl,<br>90 mM LiCl, 10 mM lithium<br>cacodylate, pH 7.4	33	6
**30**	0.5	*c-myc*	FRET	0.3	F10T	FRET	*c-myc*: 0.20 µM, 1.0 µM, 1 mM KCl,<br>99 mM LiCl, 10 mM lithium<br>cacodylate, pH 7.4<br>F10T: 0.20 µM, 1.0 µM, 10 mM KCl,<br>90 mM LiCl, 10 mM lithium<br>cacodylate, pH 7.4	1.7	6
**31**	1.3	Telomere	FRET	0.4	F10T	FRET	0.20 µM, 1.0 µM, 10 mM KCl,<br>90 mM LiCl, 10 mM lithium<br>cacodylate, pH 7.4	3.3	6
**31**	1.0	*c-myc*	FRET	0.4	F10T	FRET	*c-myc*: 0.20 µM, 1.0 µM, 1 mM KCl,<br>99 mM LiCl, 10 mM lithium<br>cacodylate, pH 7.4<br>F10T: 0.20 µM, 1.0 µM, 10 mM KCl,<br>90 mM LiCl, 10 mM lithium<br>cacodylate, pH 7.4	2.5	6
**32**	3.7	Telomere	FRET	0.1	F10T	FRET	0.20 µM, 1.0 µM, 10 mM KCl,<br>90 mM LiCl, 10 mM lithium<br>cacodylate, pH 7.4	37	6

**32**	1.8	*c-myc*	FRET	0.1	F10T	FRET	*c-myc*: 0.20 µM, 1.0 µM, 1 mM KCl, 99 mM LiCl, 10 mM lithium cacodylate, pH 7.4<br>F10T: 0.20 µM, 1.0 µM, 10 mM KCl, 90 mM LiCl, 10 mM lithium cacodylate, pH 7.4	18	[6]
**33**	n.d.	n.d.	n.d.	n.d.	n.d.	n.d.	n.d.	n.d.	**-**
**34**	n.d.	n.d.	n.d.	n.d.	n.d.	n.d.	n.d.	n.d.	**-**
**35**	7.0	Telomere	FRET	n.d.	n.d.	n.d.	0.25 µM, 2.5 µM, 25 mM KCl, 10 mM sodium cacodylate, 0.5 mM EDTA, pH 7.0	n.d.	[12]
**36**	14.0	Telomere	FRET	n.d.	n.d.	n.d.	0.25 µM, 2.5 µM, 25 mM KCl, 10 mM sodium cacodylate, 0.5 mM EDTA, pH 7.0	n.d.	[12]
**37**	0.4	Telomere	FRET	0.5	F10T	FRET	0.20 µM, 1.0 µM, 10 mM KCl, 90 mM LiCl, 10 mM lithium cacodylate, pH 7.4	0.8	[6]
**37**	-0.3	*c-myc*	FRET	0.5	F10T	FRET	*c-myc*: 0.20 µM, 1.0 µM, 1 mM KCl, 99 mM LiCl, 10 mM lithium cacodylate, pH 7.4<br>F10T: 0.20 µM, 1.0 µM, 10 mM KCl, 90 mM LiCl, 10 mM lithium cacodylate, pH 7.4	-0.6	[6]
**38**	2.9	Telomere	FRET	0.1	F10T	FRET	0.20 µM, 1.0 µM, 10 mM KCl, 90 mM LiCl, 10 mM lithium cacodylate, pH 7.4	29	[6]
**38**	0.9	*c-myc*	FRET	0.1	F10T	FRET	*c-myc*: 0.20 µM, 1.0 µM, 1 mM KCl, 99 mM LiCl, 10 mM lithium cacodylate, pH 7.4<br>F10T: 0.20 µM, 1.0 µM, 10 mM KCl, 90 mM LiCl, 10 mM lithium cacodylate, pH 7.4	9	[6]
**39**	10.1	Telomere	FRET	-0.1	F10T	FRET	0.20 µM, 1.0 µM, 10 mM KCl, 90 mM LiCl, 10 mM lithium cacodylate, pH 7.4	-101	[6]
**39**	-0.3	*c-myc*	FRET	-0.1	F10T	FRET	*c-myc*: 0.20 µM, 1.0 µM, 1 mM KCl, 99 mM LiCl, 10 mM lithium cacodylate, pH 7.4<br>F10T: 0.20 µM, 1.0 µM, 10 mM KCl, 90 mM LiCl, 10 mM lithium cacodylate, pH 7.4	3	[6]
**40**	17.2	Telomere	FRET	4.2	T-loop	FRET	0.20 µM, 0.50 µM, 50 mM potassium cacodylate, pH 7.4	4.1	[1,2]
**40**	12.0	*c-kit1*	FRET	4.2	T-loop	FRET	0.20 µM, 0.50 µM, 50 mM potassium cacodylate, pH 7.4	2.9	[2]
**40**	18.8	*c-kit2*	FRET	4.2	T-loop	FRET	0.20 µM, 0.50 µM, 50 mM potassium cacodylate, pH 7.4	4.5	[2]
**41**	21.0	Telomere	FRET	5.0	T-loop	FRET	0.20 µM, 0.50 µM, 50 mM potassium cacodylate, pH 7.4	4.2	[1,2]
**41**	16.2	*c-kit1*	FRET	5.0	T-loop	FRET	0.20 µM, 0.50 µM, 50 mM potassium cacodylate, pH 7.4	3.2	[2]
**41**	24.2	*c-kit2*	FRET	5.0	T-loop	FRET	0.20 µM, 0.50 µM, 50 mM potassium cacodylate, pH 7.4	4.8	[2]
**42**	15.0	Telomere	FRET	2.0	dsDNA	FRET	0.25 µM, 1.0 µM, 100 mM KCl, 10 mM lithium cacodylate, pH 7.4	7.5	[14]
**42**	10.3	*c-kit1*	FRET	2.0	dsDNA	FRET	0.25 µM, 1.0 µM, 100 mM KCl, 10 mM lithium cacodylate, pH 7.4	5.2	[14]
**42**	7.3	*c-kit2*	FRET	2.0	dsDNA	FRET	0.25 µM, 1.0 µM, 100 mM KCl, 10 mM lithium cacodylate, pH 7.4	3.7	[14]
**43**	18.0	Telomere	FRET	4.0	dsDNA	FRET	0.20 µM, 2.0 µM 10 mM KCl, 90 mM LiCl, 10 mM lithium cacodylate, pH 7.2	4.5	[15]
**44**	26.5	Telomere	FRET	12.5	dsDNA	FRET	0.20 µM, 0.50 µM, 50 mM potassium cacodylate, pH 7.4	2.1	[1]

**44**	11.0	*AR1*	FRET	5.1	dsDNA	FRET	0.25 µM, 1.0 µM, 100 mM KCl, 10 mM lithium cacodylate, pH 7.4	2.2	[18]
**44**	13.0	*AR3*	FRET	5.1	dsDNA	FRET	0.25 µM, 1.0 µM, 100 mM KCl, 10 mM lithium cacodylate, pH 7.4	2.5	[18]
**44**	23.5	*c-kit1*	FRET	12.5	T-loop	FRET	0.20 µM, 0.50 µM, 50 mM potassium cacodylate, pH 7.4	1.9	[2]
**44**	29.0	*c-kit2*	FRET	12.5	T-loop	FRET	0.20 µM, 0.50 µM, 50 mM potassium cacodylate, pH 7.4	2.3	[2]
**45**	n.d.	n.d.	n.d.	n.d.	n.d.	n.d.	n.d.	n.d.	-
**46**	15.0	Telomere	CD	n.d.	n.d.	n.d.	2.0 µM, 12 µM, 20 mM KCl, 5 mM potassium phosphate, 10% DMSO, pH 7.0	n.d.	[17]
**46**	12.0	Telomere	CD	n.d.	n.d.	n.d.	2.0 µM, 12 µM, 20 mM KCl, 5 mM potassium phosphate, pH 7.0	n.d.	[20]
**46**	13.0	Telomere	CD	n.d.	n.d.	n.d.	2.0 µM, 12 µM, 20 mM KCl, 5 mM potassium phosphate, pH 7.0	n.d.	[21]
**47**	n.d.	n.d.	n.d.	n.d.	n.d.	n.d.	n.d.	n.d.	-
**48**	26.2	Telomere	FRET	11.5	T-loop	FRET	0.20 µM, 0.50 µM, 50 mM potassium cacodylate, pH 7.4	2.3	[1,2]
**48**	24.5	*c-kit1*	FRET	11.5	T-loop	FRET	0.20 µM, 0.50 µM, 50 mM potassium cacodylate, pH 7.4	2.1	[2]
**48**	28.5	*c-kit2*	FRET	11.5	T-loop	FRET	0.20 µM, 0.50 µM, 50 mM potassium cacodylate, pH 7.4	2.5	[2]
**49**	n.d.	n.d.	n.d.	n.d.	n.d.	n.d.	n.d.	n.d.	-
**50**	10.5	Telomere	FRET	-0.5	T-loop	FRET	0.20 µM, 0.50 µM, 50 mM potassium cacodylate, pH 7.4	-21	[1,2]
**50**	0.8	*c-kit1*	FRET	-0.5	T-loop	FRET	0.20 µM, 0.50 µM, 50 mM potassium cacodylate, pH 7.4	-1.6	[2]
**50**	4.8	*c-kit2*	FRET	-0.5	T-loop	FRET	0.20 µM, 0.50 µM, 50 mM potassium cacodylate, pH 7.4	-9.6	[2]
**51**	18.2	Telomere	FRET	n.d.	n.d.	n.d.	0.20 µM, 0.80 µM, 50 mM KCl, 10 mM lithium cacodylate, pH 7.4	n.d.	[23]
**52**	22.2	Telomere	FRET	<0.1	dsDNA	FRET	Telomere: 0.20 µM, 0.80 µM, 50 mM KCl, 10 mM lithium cacodylate, pH 7.4; dsDNA: 0.25 µM, 1.0 µM, 100 mM KCl, 10 mM lithium cacodylate, pH 7.4	>222	[18,23]
**52**	18.8	Telomere	CD	<0.1	dsDNA	FRET	Telomere: 4.0 µM, 16 µM, 50 mM KCl, 10 mM lithium cacodylate, pH 7.4 dsDNA: 0.25 µM, 1.0 µM, 100 mM KCl, 10 mM lithium cacodylate, pH 7.4	>188	[18,24]
**52**	>16.0	*c-myc*	CD	<0.1	dsDNA	FRET	*c-myc*: 4.0 µM, 16 µM, 50 mM KCl, 10 mM lithium cacodylate, pH 7.4 dsDNA: 0.25 µM, 1.0 µM, 100 mM KCl, 10 mM lithium cacodylate, pH 7.4	>160	[18,24]
**52**	>28.0	*bcl-2*	CD	<0.1	dsDNA	FRET	*bcl-2*: 4.0 µM, 16 µM, 50 mM KCl, 10 mM lithium cacodylate, pH 7.4 dsDNA: 0.25 µM, 1.0 µM, 100 mM KCl, 10 mM lithium cacodylate, pH 7.4	280	[18,24]
**52**	15.2	*c-kit1*	CD	<0.1	dsDNA	FRET	*c-kit1*: 4.0 µM, 16 µM, 50 mM KCl, 10 mM lithium cacodylate, pH 7.4 dsDNA: 0.25 µM, 1.0 µM, 100 mM KCl, 10 mM lithium cacodylate, pH 7.4	>152	[18,24]
**52**	18.8	*c-kit2*	CD	<0.1	dsDNA	FRET	*c-kit2*: 4.0 µM, 16 µM,	>188	[18,24]

							50 mM KCl, 10 mM lithium cacodylate, pH 7.4<br>dsDNA: 0.25 μM, 1.0 μM, 100 mM KCl, 10 mM lithium cacodylate, pH 7.4		
**52**	12.2	*hTERT*	CD	<0.1	dsDNA	FRET	*hTERT*: 4.0 μM, 16 μM, 50 mM KCl, 10 mM lithium cacodylate, pH 7.4<br>dsDNA: 0.25 μM, 1.0 μM, 100 mM KCl, 10 mM lithium cacodylate, pH 7.4	>122	18,24
**52**	7.2	*AR1*	FRET	<0.1	dsDNA	FRET	0.25 μM, 1.0 μM, 100 mM KCl, 10 mM lithium cacodylate, pH 7.4	>72	18
**52**	10.0	*AR3*	FRET	<0.1	dsDNA	FRET	0.25 μM, 1.0 μM, 100 mM KCl, 10 mM lithium cacodylate, pH 7.4	>100	18
**53**	n.d.	n.d.	n.d.	n.d.	n.d.	n.d.	n.d.	n.d.	-
**54**	4.5	Telomere	FRET	n.d.	n.d.	n.d.	0.20 μM, 0.80 μM, 50 mM KCl, 10 mM lithium cacodylate, pH 7.4	n.d.	23
**55**	20.9	Telomere	FRET	n.d.	n.d.	n.d.	0.25 μM, 1.0 μM, 50 mM KCl, 10 mM lithium cacodylate, pH 7.4	n.d.	26
**56**	14.0	Telomere	FRET	n.d.	n.d.	n.d.	0.25 μM, 1.0 μM, 50 mM KCl, 10 mM lithium cacodylate, pH 7.4	n.d.	26
**57**	4.5	*AR1*	FRET	<0.1	dsDNA	FRET	0.25 μM, 1.0 μM, 100 mM KCl, 10 mM lithium cacodylate, pH 7.4	>45	18
**57**	9.3	*AR3*	FRET	<0.1	dsDNA	FRET	0.25 μM, 1.0 μM, 100 mM KCl, 10 mM lithium cacodylate, pH 7.4	>93	18
**58**	n.d.	n.d.	n.d.	n.d.	n.d.	n.d.	n.d.	n.d.	-
**59**	n.d.	n.d.	n.d.	n.d.	n.d.	n.d.	n.d.	n.d.	-
**60**	19.9	Telomere	FRET	n.d.	n.d.	n.d.	0.25 μM, 1.0 μM, 20 mM KCl, 10 mM lithium cacodylate, pH 7.4	n.d.	27
**60**	13.8	Telomere	FRET	n.d.	n.d.	n.d.	0.25 μM, 1.0 μM, 100 mM KCl, 10 mM lithium cacodylate, pH 7.4	n.d.	27
**61**	21.9	Telomere	FRET	n.d.	n.d.	n.d.	0.25 μM, 1.0 μM, 20 mM KCl, 10 mM lithium cacodylate, pH 7.4	n.d.	27
**61**	15.7	Telomere	FRET	n.d.	n.d.	n.d.	0.25 μM, 1.0 μM, 100 mM KCl, 10 mM lithium cacodylate, pH 7.4	n.d.	27
**62**	21.9	Telomere	FRET	n.d.	n.d.	n.d.	0.25 μM, 1.0 μM, 20 mM KCl, 10 mM lithium cacodylate, pH 7.4	n.d.	27
**62**	13.8	Telomere	FRET	n.d.	n.d.	n.d.	0.25 μM, 1.0 μM, 100 mM KCl, 10 mM lithium cacodylate, pH 7.4	n.d.	27
**63**	31.7	Telomere	FRET	n.d.	n.d.	n.d.	0.25 μM, 1.0 μM, 20 mM KCl, 10 mM lithium cacodylate, pH 7.4	n.d.	27
**63**	20.9	Telomere	FRET	n.d.	n.d.	n.d.	0.25 μM, 1.0 μM, 100 mM KCl, 10 mM lithium cacodylate, pH 7.4	n.d.	27
**64**	5.6	*AR1*	FRET	<0.1	dsDNA	FRET	0.25 μM, 1.0 μM, 100 mM KCl, 10 mM lithium cacodylate, pH 7.4	>56	18
**64**	9.0	*AR3*	FRET	<0.1	dsDNA	FRET	0.25 μM, 1.0 μM, 100 mM KCl, 10 mM lithium cacodylate, pH 7.4	>90	18
**64**	30.8	Telomere	FRET	<0.1	dsDNA	FRET	Telomere: 0.25 μM, 1.0 μM, 20 mM KCl, 10 mM lithium cacodylate, pH 7.4<br>dsDNA: 0.25 μM, 1.0 μM, 100 mM KCl, 10 mM lithium cacodylate, pH 7.4	>308	18,27
**64**	19.3	Telomere	FRET	<0.1	dsDNA	FRET	0.25 μM, 1.0 μM, 100 mM KCl, 10 mM lithium cacodylate, pH 7.4	>193	18,27
**65**	22.5	Telomere	FRET	n.d.	n.d.	n.d.	0.25 μM, 1.0 μM, 20 mM KCl, 10 mM lithium cacodylate, pH 7.4	n.d.	27
**65**	10.1	Telomere	FRET	n.d.	n.d.	n.d.	0.25 μM, 1.0 μM, 100 mM KCl, 10 mM lithium cacodylate, pH 7.4	n.d.	27
**66**	20.4	Telomere	FRET	n.d.	n.d.	n.d.	0.25 μM, 1.0 μM, 20 mM KCl, 10 mM lithium cacodylate, pH 7.4	n.d.	27

**66**	2.4	Telomere	FRET	n.d.	n.d.	n.d.	0.25 µM, 1.0 µM, 100 mM KCl, 10 mM lithium cacodylate, pH 7.4	n.d.	[27]
**67**	n.d.	n.d.	n.d.	n.d.	n.d.	n.d.	n.d.	n.d.	-
**68**	n.d.	n.d.	n.d.	n.d.	n.d.	n.d.	n.d.	n.d.	-
**69**	n.d.	n.d.	n.d.	n.d.	n.d.	n.d.	n.d.	n.d.	-
**70**	n.d.	n.d.	n.d.	n.d.	n.d.	n.d.	n.d.	n.d.	-
**71**	n.d.	n.d.	n.d.	n.d.	n.d.	n.d.	n.d.	n.d.	-
**72**	n.d.	n.d.	n.d.	n.d.	n.d.	n.d.	n.d.	n.d.	-
**73**	n.d.	n.d.	n.d.	n.d.	n.d.	n.d.	n.d.	n.d.	-
**74**	n.d.	n.d.	n.d.	n.d.	n.d.	n.d.	n.d.	n.d.	-
**75**	n.d.	n.d.	n.d.	n.d.	n.d.	n.d.	n.d.	n.d.	-
**76**	n.d.	n.d.	n.d.	n.d.	n.d.	n.d.	n.d.	n.d.	-
**77**	n.d.	n.d.	n.d.	n.d.	n.d.	n.d.	n.d.	n.d.	-
**78**	n.d.	n.d.	n.d.	n.d.	n.d.	n.d.	n.d.	n.d.	-
**79**	n.d.	n.d.	n.d.	n.d.	n.d.	n.d.	n.d.	n.d.	-
**80**	17.0	Telomere	FRET	4.0	dsDNA	FRET	0.20 µM, 2.0 µM, 10 mM KCl, 90 mM LiCl, 10 mM lithium cacodylate, pH 7.2	4.3	[15]
**81**	14.0	Telomere	FRET	3.0	dsDNA	FRET	0.20 µM, 2.0 µM, 10 mM KCl, 90 mM LiCl, 10 mM lithium cacodylate, pH 7.2	4.7	[15]
**82**	16.0	Telomere	FRET	3.0	dsDNA	FRET	0.20 µM, 2.0 µM, 10 mM KCl, 90 mM LiCl, 10 mM lithium cacodylate, pH 7.2	5.3	[15]
**83**	17.0	Telomere	FRET	4.0	dsDNA	FRET	0.20 µM, 2.0 µM, 10 mM KCl, 90 mM LiCl, 10 mM lithium cacodylate, pH 7.2	4.3	[15]
**84**	16.0	Telomere	FRET	5.0	dsDNA	FRET	0.20 µM, 2.0 µM, 10 mM KCl, 90 mM LiCl, 10 mM lithium cacodylate, pH 7.2	3.2	[15]
**85**	8.0	Telomere	FRET	2.0	dsDNA	FRET	0.20 µM, 2.0 µM, 10 mM KCl, 90 mM LiCl, 10 mM lithium cacodylate, pH 7.2	4.0	[15]
**86**	21.2	Telomere	FRET	6.5	T-loop	FRET	0.20 µM, 0.50 µM, 50 mM potassium cacodylate, pH 7.4	3.3	[2]
**86**	18.2	*c-kit1*	FRET	6.5	T-loop	FRET	0.20 µM, 0.50 µM, 50 mM potassium cacodylate, pH 7.4	2.8	[2]
**86**	24.2	*c-kit2*	FRET	6.5	T-loop	FRET	0.20 µM, 0.50 µM, 50 mM potassium cacodylate, pH 7.4	3.7	[2]
**87**	21.2	Telomere	FRET	6.5	dsDNA	FRET	0.20 µM, 0.50 µM, 50 mM potassium cacodylate, pH 7.4	3.3	[1]
**88**	17.5	Telomere	FRET	4.7	T-loop	FRET	0.20 µM, 0.50 µM, 50 mM potassium cacodylate, pH 7.4	3.7	[1,2]
**88**	15.8	*c-kit1*	FRET	4.7	T-loop	FRET	0.20 µM, 0.50 µM, 50 mM potassium cacodylate, pH 7.4	3.4	[2]
**88**	20.0	*c-kit2*	FRET	4.7	T-loop	FRET	0.20 µM, 0.50 µM, 50 mM potassium cacodylate, pH 7.4	4.3	[2]
**89**	6.2	Telomere	FRET	3.0	T-loop	FRET	0.20 µM, 0.50 µM, 50 mM potassium cacodylate, pH 7.4	2.1	[1,2]
**89**	2.0	*c-kit1*	FRET	3.0	T-loop	FRET	0.20 µM, 0.50 µM, 50 mM potassium cacodylate, pH 7.4	0.67	[2]
**89**	5.5	*c-kit2*	FRET	3.0	T-loop	FRET	0.20 µM, 0.50 µM, 50 mM potassium cacodylate, pH 7.4	1.8	[2]
**90**	28.2	Telomere	FRET	10.7	dsDNA	FRET	0.20 µM, 0.50 µM, 50 mM potassium cacodylate, pH 7.4	2.6	[1,2]
**90**	25.8	*c-kit1*	FRET	10.7	T-loop	FRET	0.20 µM, 0.50 µM, 50 mM potassium cacodylate, pH 7.4	2.4	[2]
**90**	31.5	*c-kit2*	FRET	10.7	T-loop	FRET	0.20 µM, 0.50 µM, 50 mM potassium cacodylate, pH 7.4	2.9	[2]
**91**	10.2	Telomere	FRET	3.2	T-loop	FRET	0.20 µM, 0.50 µM, 50 mM potassium cacodylate, pH 7.4	3.2	[1,2]
**91**	12.0	*c-kit1*	FRET	3.2	T-loop	FRET	0.20 µM, 0.50 µM, 50 mM potassium cacodylate, pH 7.4	3.8	[2]

**91**	9.2	*c-kit2*	FRET	3.2	T-loop	FRET	0.20 μM, 0.50 μM, 50 mM potassium cacodylate, pH 7.4	2.9	[2]
**92**	<2.0	Telomere	FRET	0.01	T-loop	FRET	0.20 μM, 1.0 μM, 60 mM potassium cacodylate, pH 7.4	<200	[29]
**92**	2.7	*HSP90A*	FRET	0.01	T-loop	FRET	0.20 μM, 1.0 μM, 60 mM potassium cacodylate, pH 7.4	270	[29]
**92**	4.5	*HSP90B*	FRET	0.01	T-loop	FRET	0.20 μM, 1.0 μM, 60 mM potassium cacodylate, pH 7.4	450	[29]
**92**	0.7	*kRAS21*	FRET	0.01	T-loop	FRET	0.20 μM, 1.0 μM, 60 mM potassium cacodylate, pH 7.4	70	[29]
**92**	0.2	*kRAS32*	FRET	0.01	T-loop	FRET	0.20 μM, 1.0 μM, 60 mM potassium cacodylate, pH 7.4	20	[29]
**92**	2.4	*bcl-2*	FRET	0.01	T-loop	FRET	0.20 μM, 1.0 μM, 60 mM potassium cacodylate, pH 7.4	240	[29]
**93**	13.0	*hTERT*	UV	2.0	duplex DNA	UV	5.0 μM, 5.0 μM, 50 mM KCl, 10 mM Tris-HCl, 1 mM EDTA, pH 7.4	6.5	[31]
**93**	11.8	Telomere	FRET	0.6	T-loop	FRET	0.20 μM, 1.0 μM, 60 mM potassium cacodylate, pH 7.4	20	[30]
**93**	5.0	Telomere	UV	2.0	duplex DNA	UV	5.0 μM, 5.0 μM, 50 mM KCl, 10 mM Tris-HCl, 1 mM EDTA, pH 7.4	2.5	[31]
**93**	15.7	*HSP90A*	FRET	0.6	T-loop	FRET	0.20 μM, 1.0 μM, 60 mM potassium cacodylate, pH 7.4	26	[30]
**93**	12.7	*HSP90B*	FRET	0.6	T-loop	FRET	0.20 μM, 1.0 μM, 60 mM potassium cacodylate, pH 7.4	21	[30]
**93**	11.0	*kRAS21*	FRET	0.6	T-loop	FRET	0.20 μM, 1.0 μM, 60 mM potassium cacodylate, pH 7.4	18	[30]
**93**	9.6	*kRAS32*	FRET	0.6	T-loop	FRET	0.20 μM, 1.0 μM, 60 mM potassium cacodylate, pH 7.4	16	[30]
**93**	13.3	*bcl-2*	FRET	0.6	T-loop	FRET	0.20 μM, 1.0 μM, 60 mM potassium cacodylate, pH 7.4	22	[30]
**94**	1.4	Telomere	FRET	0.01	T-loop	FRET	0.20 μM, 1.0 μM, 60 mM potassium cacodylate, pH 7.4	140	[29]
**94**	3.9	*HSP90A*	FRET	0.01	T-loop	FRET	0.20 μM, 1.0 μM, 60 mM potassium cacodylate, pH 7.4	390	[29]
**94**	6.8	*HSP90B*	FRET	0.01	T-loop	FRET	0.20 μM, 1.0 μM, 60 mM potassium cacodylate, pH 7.4	680	[29]
**94**	3.4	*kRAS21*	FRET	0.01	T-loop	FRET	0.20 μM, 1.0 μM, 60 mM potassium cacodylate, pH 7.4	340	[29]
**94**	1.2	*kRAS32*	FRET	0.01	T-loop	FRET	0.20 μM, 1.0 μM, 60 mM potassium cacodylate, pH 7.4	120	[29]
**94**	4.2	*bcl-2*	FRET	0.01	T-loop	FRET	0.20 μM, 1.0 μM, 60 mM potassium cacodylate, pH 7.4	420	[29]
**95**	9.8	Telomere	FRET	0.6	T-loop	FRET	0.20 μM, 1.0 μM, 60 mM potassium cacodylate, pH 7.4	16	[29]
**95**	9.1	*HSP90A*	FRET	0.6	T-loop	FRET	0.20 μM, 1.0 μM, 60 mM potassium cacodylate, pH 7.4	15	[29]
**95**	8.7	*HSP90B*	FRET	0.6	T-loop	FRET	0.20 μM, 1.0 μM, 60 mM potassium cacodylate, pH 7.4	14	[29]
**95**	4.8	*kRAS21*	FRET	0.6	T-loop	FRET	0.20 μM, 1.0 μM, 60 mM potassium cacodylate, pH 7.4	8.0	[29]
**95**	2.2	*kRAS32*	FRET	0.6	T-loop	FRET	0.20 μM, 1.0 μM, 60 mM potassium cacodylate, pH 7.4	3.7	[29]
**95**	5.9	*bcl-2*	FRET	0.6	T-loop	FRET	0.20 μM, 1.0 μM, 60 mM potassium cacodylate, pH 7.4	9.8	[29]
**96**	12.3	Telomere	FRET	0.4	T-loop	FRET	0.20 μM, 1.0 μM, 60 mM potassium cacodylate, pH 7.4	31	[29]
**96**	18.1	*HSP90A*	FRET	0.4	T-loop	FRET	0.20 μM, 1.0 μM, 60 mM potassium cacodylate, pH 7.4	45	[29]
**96**	15.0	*HSP90B*	FRET	0.4	T-loop	FRET	0.20 μM, 1.0 μM, 60 mM potassium cacodylate, pH 7.4	38	[29]
**96**	11.0	*kRAS21*	FRET	0.4	T-loop	FRET	0.20 μM, 1.0 μM, 60 mM potassium cacodylate, pH 7.4	28	[29]
**96**	6.1	*kRAS32*	FRET	0.4	T-loop	FRET	0.20 μM, 1.0 μM, 60 mM potassium cacodylate, pH 7.4	15	[29]

**96**	15.1	*bcl-2*	FRET	0.4	T-loop	FRET	0.20 µM, 1.0 µM, 60 mM potassium cacodylate, pH 7.4	38	[29]
**97**	6.0	Telomere	FRET	1.5	T-loop	FRET	0.20 µM, 1.0 µM, 60 mM potassium cacodylate, pH 7.4	4.0	[29]
**97**	11.0	*HSP90A*	FRET	1.5	T-loop	FRET	0.20 µM, 1.0 µM, 60 mM potassium cacodylate, pH 7.4	7.3	[29]
**97**	9.4	*HSP90B*	FRET	1.5	T-loop	FRET	0.20 µM, 1.0 µM, 60 mM potassium cacodylate, pH 7.4	6.3	[29]
**97**	2.4	*kRAS21*	FRET	1.5	T-loop	FRET	0.20 µM, 1.0 µM, 60 mM potassium cacodylate, pH 7.4	1.6	[29]
**97**	1.5	*kRAS32*	FRET	1.5	T-loop	FRET	0.20 µM, 1.0 µM, 60 mM potassium cacodylate, pH 7.4	1.0	[29]
**97**	5.8	*bcl-2*	FRET	1.5	T-loop	FRET	0.20 µM, 1.0 µM, 60 mM potassium cacodylate, pH 7.4	3.9	[29]
**98**	9.0	Telomere	FRET	0.1	T-loop	FRET	0.20 µM, 1.0 µM, 60 mM potassium cacodylate, pH 7.4	90	[29]
**98**	15.7	*HSP90A*	FRET	0.1	T-loop	FRET	0.20 µM, 1.0 µM, 60 mM potassium cacodylate, pH 7.4	157	[29]
**98**	13.8	*HSP90B*	FRET	0.1	T-loop	FRET	0.20 µM, 1.0 µM, 60 mM potassium cacodylate, pH 7.4	138	[29]
**98**	6.1	*kRAS21*	FRET	0.1	T-loop	FRET	0.20 µM, 1.0 µM, 60 mM potassium cacodylate, pH 7.4	61	[29]
**98**	3.0	*kRAS32*	FRET	0.1	T-loop	FRET	0.20 µM, 1.0 µM, 60 mM potassium cacodylate, pH 7.4	30	[29]
**98**	10.0	*bcl-2*	FRET	0.1	T-loop	FRET	0.20 µM, 1.0 µM, 60 mM potassium cacodylate, pH 7.4	100	[29]
**99**	<2.0	Telomere	FRET	0.01	T-loop	FRET	0.20 µM, 1.0 µM, 60 mM potassium cacodylate, pH 7.4	<200	[29]
**99**	2.5	*HSP90A*	FRET	0.01	T-loop	FRET	0.20 µM, 1.0 µM, 60 mM potassium cacodylate, pH 7.4	250	[29]
**99**	<2.0	*HSP90B*	FRET	0.01	T-loop	FRET	0.20 µM, 1.0 µM, 60 mM potassium cacodylate, pH 7.4	<200	[29]
**99**	3.0	*kRAS21*	FRET	0.01	T-loop	FRET	0.20 µM, 1.0 µM, 60 mM potassium cacodylate, pH 7.4	300	[29]
**99**	1.5	*kRAS32*	FRET	0.01	T-loop	FRET	0.20 µM, 1.0 µM, 60 mM potassium cacodylate, pH 7.4	150	[29]
**99**	2.8	*bcl-2*	FRET	0.01	T-loop	FRET	0.20 µM, 1.0 µM, 60 mM potassium cacodylate, pH 7.4	280	[29]
**100**	14.3	Telomere	FRET	0.8	T-loop	FRET	0.20 µM, 1.0 µM, 60 mM potassium cacodylate, pH 7.4	18	[29]
**100**	19.8	*HSP90A*	FRET	0.8	T-loop	FRET	0.20 µM, 1.0 µM, 60 mM potassium cacodylate, pH 7.4	25	[29]
**100**	16.5	*HSP90B*	FRET	0.8	T-loop	FRET	0.20 µM, 1.0 µM, 60 mM potassium cacodylate, pH 7.4	21	[29]
**100**	9.8	*kRAS21*	FRET	0.8	T-loop	FRET	0.20 µM, 1.0 µM, 60 mM potassium cacodylate, pH 7.4	12	[29]
**100**	6.1	*kRAS32*	FRET	0.8	T-loop	FRET	0.20 µM, 1.0 µM, 60 mM potassium cacodylate, pH 7.4	7.6	[29]
**100**	15.8	*bcl-2*	FRET	0.8	T-loop	FRET	0.20 µM, 1.0 µM, 60 mM potassium cacodylate, pH 7.4	20	[29]
**101**	15.9	Telomere	FRET	0.2	T-loop	FRET	0.20 µM, 1.0 µM, 60 mM potassium cacodylate, pH 7.4	80	[29]
**101**	20.6	*HSP90A*	FRET	0.2	T-loop	FRET	0.20 µM, 1.0 µM, 60 mM potassium cacodylate, pH 7.4	103	[29]
**101**	16.9	*HSP90B*	FRET	0.2	T-loop	FRET	0.20 µM, 1.0 µM, 60 mM potassium cacodylate, pH 7.4	84	[29]
**101**	11.7	*kRAS21*	FRET	0.2	T-loop	FRET	0.20 µM, 1.0 µM, 60 mM potassium cacodylate, pH 7.4	58	[29]
**101**	6.6	*kRAS32*	FRET	0.2	T-loop	FRET	0.20 µM, 1.0 µM, 60 mM potassium cacodylate, pH 7.4	33	[29]
**101**	16.1	*bcl-2*	FRET	0.2	T-loop	FRET	0.20 µM, 1.0 µM, 60 mM potassium cacodylate, pH 7.4	80	[29]
**102**	28.0	Telomere	FRET	7.5	T-loop	FRET	0.20 µM, 0.50 µM, 50 mM potassium cacodylate, pH 7.4	3.7	[1,2]

**102**	25.2	*c-kit1*	FRET	7.5	T-loop	FRET	0.20 µM, 0.50 µM, 50 mM potassium cacodylate, pH 7.4	3.4	[2]
**102**	29.8	*c-kit2*	FRET	7.5	T-loop	FRET	0.20 µM, 0.50 µM, 50 mM potassium cacodylate, pH 7.4	4.0	[2]
**103**	30.0	Telomere	FRET	4.0	T-loop	FRET	0.20 µM, 0.50 µM, 50 mM potassium cacodylate, pH 7.4	7.5	[1,2]
**103**	27.2	*c-kit1*	FRET	4.0	T-loop	FRET	0.20 µM, 0.50 µM, 50 mM potassium cacodylate, pH 7.4	6.8	[2]
**103**	31.2	*c-kit2*	FRET	4.0	T-loop	FRET	0.20 µM, 0.50 µM, 50 mM potassium cacodylate, pH 7.4	7.8	[2]
**104**	n.d.	n.d.	n.d.	n.d.	n.d.	n.d.	n.d.	n.d.	**-**
**105**	n.d.	n.d.	n.d.	n.d.	n.d.	n.d.	n.d.	n.d.	**-**
**106**	33.2	Telomere	FRET	3.8	T-loop	FRET	0.20 µM, 0.50 µM, 50 mM potassium cacodylate, pH 7.4	8.7	[1,54]
**106**	29.8	*c-kit1*	FRET	3.8	T-loop	FRET	0.20 µM, 0.50 µM, 50 mM potassium cacodylate, pH 7.4	7.8	[2,54]
**106**	36.5	*c-kit2*	FRET	3.8	T-loop	FRET	0.20 µM, 0.50 µM, 50 mM potassium cacodylate, pH 7.4	9.6	[2,54]
**106**	22.5	*bcl-2*	FRET	3.8	T-loop	FRET	0.20 µM, 0.50 µM, 50 mM potassium cacodylate, pH 7.4	5.9	[54]
**107**	35.2	Telomere	FRET	4.2	T-loop	FRET	0.20 µM, 0.50 µM, 50 mM potassium cacodylate, pH 7.4	8.4	[1,2]
**107**	33.8	*c-kit1*	FRET	4.2	T-loop	FRET	0.20 µM, 0.50 µM, 50 mM potassium cacodylate, pH 7.4	8.0	[2]
**107**	36.2	*c-kit2*	FRET	4.2	T-loop	FRET	0.20 µM, 0.50 µM, 50 mM potassium cacodylate, pH 7.4	8.6	[2]
**108**	n.d.	n.d.	n.d.	n.d.	n.d.	n.d.	n.d.	n.d.	**-**
**109**	23.7	Telomere	FRET	3.5	T-loop	FRET	0.20 µM, 0.50 µM, 50 mM potassium cacodylate, pH 7.4	6.8	[1,2]
**109**	18.0	*c-kit1*	FRET	3.5	T-loop	FRET	0.20 µM, 0.50 µM, 50 mM potassium cacodylate, pH 7.4	5.1	[2]
**109**	24.0	*c-kit2*	FRET	3.5	T-loop	FRET	0.20 µM, 0.50 µM, 50 mM potassium cacodylate, pH 7.4	6.9	[2]
**110**	14.2	Telomere	FRET	-0.5	T-loop	FRET	0.20 µM, 0.50 µM, 50 mM potassium cacodylate, pH 7.4	-28	[1,2]
**110**	-0.8	*c-kit1*	FRET	-0.5	T-loop	FRET	0.20 µM, 0.50 µM, 50 mM potassium cacodylate, pH 7.4	1.5	[2]
**110**	6.0	*c-kit2*	FRET	-0.5	T-loop	FRET	0.20 µM, 0.50 µM, 50 mM potassium cacodylate, pH 7.4	-12	[2]
**111**	n.d.	n.d.	n.d.	n.d.	n.d.	n.d.	n.d.	n.d.	**-**
**112**	n.d.	n.d.	n.d.	n.d.	n.d.	n.d.	n.d.	n.d.	**-**
**113**	3.2	Telomere	FRET	n.d.	n.d.	n.d.	0.20 µM, 0.80 µM, 50 mM KCl, 10 mM lithium cacodylate, pH 7.4	n.d.	[23]
**114**	n.d.	n.d.	n.d.	n.d.	n.d.	n.d.	n.d.	n.d.	**-**
**115**	n.d.	n.d.	n.d.	n.d.	n.d.	n.d.	n.d.	n.d.	**-**
**116**	27.8	Telomere	FRET	n.d.	n.d.	n.d.	0.25 µM, 1.0 µM, 20 mM KCl, 10 mM lithium cacodylate, pH 7.4	n.d.	[27]
**116**	17.8	Telomere	FRET	n.d.	n.d.	n.d.	0.25 µM, 1.0 µM, 100 mM KCl, 10 mM lithium cacodylate, pH 7.4	n.d.	[27]
**117**	34.6	Telomere	FRET	n.d.	n.d.	n.d.	0.25 µM, 1.0 µM, 20 mM KCl, 10 mM lithium cacodylate, pH 7.4	n.d.	[27]
**117**	23.8	Telomere	FRET	n.d.	n.d.	n.d.	0.25 µM, 1.0 µM, 100 mM KCl, 10 mM lithium cacodylate, pH 7.4	n.d.	[27]
**118**	18.0	Telomere	FRET	n.d.	n.d.	n.d.	0.25 µM, 1.0 µM, 20 mM KCl, 10 mM lithium cacodylate, pH 7.4	n.d.	[27]
**118**	3.9	Telomere	FRET	n.d.	n.d.	n.d.	0.25 µM, 1.0 µM, 100 mM KCl, 10 mM lithium cacodylate, pH 7.4	n.d.	[27]
**119**	27.9	Telomere	FRET	n.d.	n.d.	n.d.	0.25 µM, 1.0 µM, 20 mM KCl, 10 mM lithium cacodylate, pH 7.4	n.d.	[27]
**119**	9.8	Telomere	FRET	n.d.	n.d.	n.d.	0.25 µM, 1.0 µM, 100 mM KCl, 10 mM lithium cacodylate, pH 7.4	n.d.	[27]
**120**	15.6	Telomere	FRET	n.d.	n.d.	n.d.	0.2 µM, 0.80 µM,	n.d.	[23]

							50 mM KCl, 10 mM lithium cacodylate, pH 7.4		
**121**	20.3	Telomere	FRET	n.d.	n.d.	n.d.	0.20 µM, 0.80 µM, 50 mM KCl, 10 mM lithium cacodylate, pH 7.4	n.d.	[23]
**122**	n.d.	n.d.	n.d.	n.d.	n.d.	n.d.	n.d.	n.d.	**-**
**123**	15.9	Telomere	FRET	n.d.	n.d.	n.d.	0.25 µM, 1.0 µM, 50 mM KCl, 10 mM lithium cacodylate, pH 7.4	n.d.	[26]
**124**	11.9	Telomere	FRET	n.d.	n.d.	n.d.	0.25 µM, 1.0 µM, 50 mM KCl, 10 mM lithium cacodylate, pH 7.4	n.d.	[26]
**125**	n.d.	n.d.	n.d.	n.d.	n.d.	n.d.	n.d.	n.d.	**-**
**126**	n.d.	n.d.	n.d.	n.d.	n.d.	n.d.	n.d.	n.d.	**-**
**127**	n.d.	n.d.	n.d.	n.d.	n.d.	n.d.	n.d.	n.d.	**-**
**128**	n.d.	n.d.	n.d.	n.d.	n.d.	n.d.	n.d.	n.d.	**-**
**129**	n.d.	n.d.	n.d.	n.d.	n.d.	n.d.	n.d.	n.d.	**-**
**130**	n.d.	n.d.	n.d.	n.d.	n.d.	n.d.	n.d.	n.d.	**-**
**131**	n.d.	n.d.	n.d.	n.d.	n.d.	n.d.	n.d.	n.d.	**-**
**132**	n.d.	n.d.	n.d.	n.d.	n.d.	n.d.	n.d.	n.d.	**-**
**133**	n.d.	n.d.	n.d.	n.d.	n.d.	n.d.	n.d.	n.d.	**-**
**134**	n.d.	n.d.	n.d.	n.d.	n.d.	n.d.	n.d.	n.d.	**-**
**135**	n.d.	n.d.	n.d.	n.d.	n.d.	n.d.	n.d.	n.d.	**-**
**136**	n.d.	n.d.	n.d.	n.d.	n.d.	n.d.	n.d.	n.d.	**-**
**137**	27.7	Telomere	FRET	0.2	T-loop	FRET	0.20 µM, 0.50 µM, 50 mM potassium cacodylate, pH 7.4	138	[1,2]
**137**	0.01	*c-kit1*	FRET	0.2	T-loop	FRET	0.20 µM, 0.50 µM, 50 mM potassium cacodylate, pH 7.4	0.05	[2]
**137**	4.2	*c-kit2*	FRET	0.2	T-loop	FRET	0.20 µM, 0.50 µM, 50 mM potassium cacodylate, pH 7.4	21	[2]
**138**	0.1	*AR1*	FRET	<0.1	dsDNA	FRET	0.25 µM, 1.0 µM, 100 mM KCl, 10 mM lithium cacodylate, pH 7.4	>1.0	[18]
**138**	0.4	*AR3*	FRET	<0.1	dsDNA	FRET	0.25 µM, 1.0 µM, 100 mM KCl, 10 mM lithium cacodylate, pH 7.4	>4.0	[18]
**139**	<2.0	Telomere	FRET	0.01	T-loop	FRET	0.20 µM, 1.0 µM, 60 mM potassium cacodylate, pH 7.4	<200	[37,39]
**139**	22.0	Telomere	FRET	0.01	T-loop	FRET	0.20 µM, 2.0 µM, 60 mM potassium cacodylate, pH 7.4	2200	[37,39]
**139**	29.0	*HSP90A*	FRET	0.01	T-loop	FRET	0.20 µM, 1.0 µM, 60 mM potassium cacodylate, pH 7.4	2900	[37,39]
**139**	32.0	*HSP90A*	FRET	0.01	T-loop	FRET	0.20 µM, 2.0 µM, 60 mM potassium cacodylate, pH 7.4	3200	[37,39]
**139**	24.0	*HSP90B*	FRET	0.01	T-loop	FRET	0.20 µM, 1.0 µM, 60 mM potassium cacodylate, pH 7.4	2400	[37,39]
**139**	29.0	*HSP90B*	FRET	0.01	T-loop	FRET	0.20 µM, 2.0 µM, 60 mM potassium cacodylate, pH 7.4	2900	[37,39]
**140**	<2.0	Telomere	FRET	0.01	T-loop	FRET	0.20 µM, 1.0 µM, 60 mM potassium cacodylate, pH 7.4	<200	[37,39]
**140**	16.0	Telomere	FRET	0.01	T-loop	FRET	0.20 µM, 2.0 µM, 60 mM potassium cacodylate, pH 7.4	1600	[37,39]
**140**	22.0	*HSP90A*	FRET	0.01	T-loop	FRET	0.20 µM, 1.0 µM, 60 mM potassium cacodylate, pH 7.4	2200	[37,39]
**140**	24.0	*HSP90A*	FRET	0.01	T-loop	FRET	0.20 µM, 2.0 µM, 60 mM potassium cacodylate, pH 7.4	2400	[37,39]
**140**	22.0	*HSP90B*	FRET	0.01	T-loop	FRET	0.20 µM, 1.0 µM, 60 mM potassium cacodylate, pH 7.4	2200	[37,39]
**140**	26.0	*HSP90B*	FRET	0.01	T-loop	FRET	0.20 µM, 2.0 µM, 60 mM potassium cacodylate, pH 7.4	2600	[37,39]
**141**	29.7	Telomere	FRET	3.5	T-loop	FRET	0.20 µM, 0.50 µM, 50 mM potassium cacodylate, pH 7.4	8.5	[2]
**141**	27.0	*c-kit1*	FRET	3.5	T-loop	FRET	0.20 µM, 0.50 µM, 50 mM potassium cacodylate, pH 7.4	7.7	[2]
**141**	32.0	*c-kit2*	FRET	3.5	T-loop	FRET	0.20 µM, 0.50 µM, 50 mM potassium cacodylate, pH 7.4	9.1	[2]

**142**	23.0	Telomere	FRET	0.01	T-loop	FRET	0.20 µM, 1.0 µM, 60 mM potassium cacodylate, pH 7.4	2300	[37,39]
**142**	26.0	Telomere	FRET	0.01	T-loop	FRET	0.20 µM, 2.0 µM, 60 mM potassium cacodylate, pH 7.4	2600	[37,39]
**142**	27.0	*HSP90A*	FRET	0.01	T-loop	FRET	0.20 µM, 1.0 µM, 60 mM potassium cacodylate, pH 7.4	2700	[37,39]
**142**	30.0	*HSP90A*	FRET	0.01	T-loop	FRET	0.20 µM, 2.0 µM, 60 mM potassium cacodylate, pH 7.4	3000	[37,39]
**142**	21.0	*HSP90B*	FRET	0.01	T-loop	FRET	0.20 µM, 1.0 µM, 60 mM potassium cacodylate, pH 7.4	2100	[37,39]
**142**	28.0	*HSP90B*	FRET	0.01	T-loop	FRET	0.20 µM, 2.0 µM, 60 mM potassium cacodylate, pH 7.4	2800	[37,39]
**143**	0.2	Telomere	FRET	0.3	T-loop	FRET	0.20 µM, 1.0 µM, 60 mM potassium cacodylate, pH 7.4	0.7	[38]
**143**	0.9	*HSP90A*	FRET	0.3	T-loop	FRET	0.20 µM, 1.0 µM, 60 mM potassium cacodylate, pH 7.4	3.0	[38]
**143**	1.1	*HSP90B*	FRET	0.3	T-loop	FRET	0.20 µM, 1.0 µM, 60 mM potassium cacodylate, pH 7.4	3.7	[38]
**144**	<2.0	Telomere	FRET	0.01	T-loop	FRET	0.20 µM, 1.0 µM, 60 mM potassium cacodylate, pH 7.4	<200	[37,39]
**144**	<2.0	Telomere	FRET	0.01	T-loop	FRET	0.20 µM, 2.0 µM, 60 mM potassium cacodylate, pH 7.4	<200	[37,39]
**144**	<2.0	*HSP90A*	FRET	0.01	T-loop	FRET	0.20 µM, 1.0 µM, 60 mM potassium cacodylate, pH 7.4	<200	[37,39]
**144**	<2.0	*HSP90A*	FRET	0.01	T-loop	FRET	0.20 µM, 2.0 µM, 60 mM potassium cacodylate, pH 7.4	<200	[37,39]
**144**	<2.0	*HSP90B*	FRET	0.01	T-loop	FRET	0.20 µM, 1.0 µM, 60 mM potassium cacodylate, pH 7.4	<200	[37,39]
**144**	<2.0	*HSP90B*	FRET	0.01	T-loop	FRET	0.20 µM, 2.0 µM, 60 mM potassium cacodylate, pH 7.4	<200	[37,39]
**145**	29.7	Telomere	FRET	3.5	duplex	FRET	0.20 µM, 0.50 µM, 50 mM potassium cacodylate, pH 7.4	8.5	[1]
**145**	11.2	*c-kit1*	FRET	3.5	duplex	FRET	0.20 µM, 0.50 µM, 50 mM potassium cacodylate, pH 7.4	3.2	[1,41]
**145**	29.0	*c-kit2*	FRET	3.5	duplex	FRET	0.20 µM, 0.50 µM, 50 mM potassium cacodylate, pH 7.4	8.3	[1,41]
**145**	27.6	*bcl-2*	FRET	3.5	duplex	FRET	0.20 µM, 0.50 µM, 50 mM potassium cacodylate, pH 7.4	7.9	[1,41]
**146**	27.5	Telomere	FRET	2.2	T-loop	FRET	0.20 µM, 0.50 µM, 50 mM potassium cacodylate, pH 7.4	12	[1,2]
**146**	20.2	*c-kit1*	FRET	2.2	T-loop	FRET	0.20 µM, 0.50 µM, 50 mM potassium cacodylate, pH 7.4	9.2	[2]
**146**	20.5	*c-kit2*	FRET	2.2	T-loop	FRET	0.20 µM, 0.50 µM, 50 mM potassium cacodylate, pH 7.4	9.3	[2]
**147**	19.0	Telomere	FRET	0.8	T-loop	FRET	0.20 µM, 0.50 µM, 50 mM potassium cacodylate, pH 7.4	24	[42]
**148**	13.7	Telomere	FRET	3.0	T-loop	FRET	0.20 µM, 0.50 µM, 50 mM potassium cacodylate, pH 7.4	4.6	[1,2]
**148**	1.0	*c-kit1*	FRET	3.0	T-loop	FRET	0.20 µM, 0.50 µM, 50 mM potassium cacodylate, pH 7.4	0.33	[2]
**148**	8.5	*c-kit2*	FRET	3.0	T-loop	FRET	0.20 µM, 0.50 µM, 50 mM potassium cacodylate, pH 7.4	2.8	[2]
**149**	24.7	Telomere	FRET	1.7	T-loop	FRET	0.20 µM, 1.0 µM, 60 mM potassium cacodylate, pH 7.4	14	[38]
**149**	30.6	*HSP90A*	FRET	1.7	T-loop	FRET	0.20 µM, 1.0 µM, 60 mM potassium cacodylate, pH 7.4	18	[38]
**149**	27.6	*HSP90B*	FRET	1.7	T-loop	FRET	0.20 µM, 1.0 µM, 60 mM potassium cacodylate, pH 7.4	16	[38]
**150**	27.0	Telomere	FRET	0.01	T-loop	FRET	0.20 µM, 1.0 µM, 60 mM potassium cacodylate, pH 7.4	2700	[37]
**150**	31.0	Telomere	FRET	1.0	T-loop	FRET	0.20 µM, 2.0 µM, 60 mM potassium cacodylate, pH 7.4	31	[37]
**150**	34.0	*HSP90A*	FRET	0.01	T-loop	FRET	0.20 µM, 1.0 µM, 60 mM potassium cacodylate, pH 7.4	3400	[37]

**150**	36.0	*HSP90A*	FRET	1.0	T-loop	FRET	0.20 μM, 2.0 μM, 60 mM potassium cacodylate, pH 7.4	36	37
**150**	29.0	*HSP90B*	FRET	0.01	T-loop	FRET	0.20 μM, 1.0 μM, 60 mM potassium cacodylate, pH 7.4	2900	37
**150**	34.0	*HSP90B*	FRET	1.0	T-loop	FRET	0.20 μM, 2.0 μM, 60 mM potassium cacodylate, pH 7.4	34	37
**151**	26.0	Telomere	FRET	2.0	T-loop	FRET	0.20 μM, 1.0 μM, 60 mM potassium cacodylate, pH 7.4	13	37,39
**151**	31.0	Telomere	FRET	8.0	T-loop	FRET	0.20 μM, 2.0 μM, 60 mM potassium cacodylate, pH 7.4	3.9	37,39
**151**	33.0	*HSP90A*	FRET	2.0	T-loop	FRET	0.20 μM, 1.0 μM, 60 mM potassium cacodylate, pH 7.4	16	37,39
**151**	36.0	*HSP90A*	FRET	8.0	T-loop	FRET	0.20 μM, 2.0 μM, 60 mM potassium cacodylate, pH 7.4	4.5	37,39
**151**	28.0	*HSP90B*	FRET	2.0	T-loop	FRET	0.20 μM, 1.0 μM, 60 mM potassium cacodylate, pH 7.4	14	37,39
**151**	32.0	*HSP90B*	FRET	8.0	T-loop	FRET	0.20 μM, 2.0 μM, 60 mM potassium cacodylate, pH 7.4	4.0	37,39
**152**	34.5	Telomere	FRET	2.0	dsDNA	FRET	0.20 μM, 0.50 μM, 50 mM potassium cacodylate, pH 7.4	17	1,2
**152**	31.5	*c-kit1*	FRET	2.0	dsDNA	FRET	0.20 μM, 0.50 μM, 50 mM potassium cacodylate, pH 7.4	16	2
**152**	39.2	*c-kit2*	FRET	2.0	dsDNA	FRET	0.20 μM, 0.50 μM, 50 mM potassium cacodylate, pH 7.4	20	2
**152**	21.0	*bcl-2*	FRET	2.0	dsDNA	FRET	0.20 μM, 0.50 μM, 50 mM potassium cacodylate, pH 7.4	10	2,54
**153**	28.3	Telomere	FRET	1.3	T-loop	FRET	0.20 μM, 1.0 μM, 60 mM potassium cacodylate, pH 7.4	22	38,39,43
**153**	1.8	*c-kit1*	FRET	1.3	T-loop	FRET	0.20 μM, 1.0 μM, 60 mM potassium cacodylate, pH 7.4	1.4	38,39,43
**153**	15.2	*c-kit2*	FRET	1.3	T-loop	FRET	0.20 μM, 1.0 μM, 60 mM potassium cacodylate, pH 7.4	12	38,39,43
**153**	36.3	*HSP90A*	FRET	1.3	T-loop	FRET	0.20 μM, 1.0 μM, 60 mM potassium cacodylate, pH 7.4	28	38,39,43
**153**	32.0	*HSP90B*	FRET	1.3	T-loop	FRET	0.20 μM, 1.0 μM, 60 mM potassium cacodylate, pH 7.4	24	38,39,43
**154**	20.5	Telomere	FRET	3.7	T-loop	FRET	0.20 μM, 0.50 μM, 50 mM potassium cacodylate, pH 7.4	5.5	1,2
**154**	16.0	*c-kit1*	FRET	3.7	T-loop	FRET	0.20 μM, 0.50 μM, 50 mM potassium cacodylate, pH 7.4	4.3	2
**154**	15.8	*c-kit2*	FRET	3.7	T-loop	FRET	0.20 μM, 0.50 μM, 50 mM potassium cacodylate, pH 7.4	4.3	2
**155**	<2.0	Telomere	FRET	0.01	T-loop	FRET	0.2 μM, 1.0 μM, 60 mM potassium cacodylate, pH 7.4	<200	37,39
**155**	15.0	Telomere	FRET	0.01	T-loop	FRET	0.20 μM, 2.0 μM, 60 mM potassium cacodylate, pH 7.4	1500	37,39
**155**	21.0	*HSP90A*	FRET	0.01	T-loop	FRET	0.20 μM, 1.0 μM, 60 mM potassium cacodylate, pH 7.4	2100	37,39
**155**	23.0	*HSP90A*	FRET	0.01	T-loop	FRET	0.20 μM, 2.0 μM, 60 mM potassium cacodylate, pH 7.4	2300	37,39
**155**	13.0	*HSP90B*	FRET	0.01	T-loop	FRET	0.20 μM, 1.0 μM, 60 mM potassium cacodylate, pH 7.4	1300	37,39
**155**	17.0	*HSP90B*	FRET	0.01	T-loop	FRET	0.20 μM, 2.0 μM, 60 mM potassium cacodylate, pH 7.4	1700	37,39
**156**	<2.0	Telomere	FRET	0.01	T-loop	FRET	0.20 μM, 1.0 μM, 60 mM potassium cacodylate, pH 7.4	<200	37,39
**156**	22.0	Telomere	FRET	0.01	T-loop	FRET	0.20 μM, 2.0 μM, 60 mM potassium cacodylate, pH 7.4	2200	37,39
**156**	2.0	*HSP90A*	FRET	0.01	T-loop	FRET	0.20 μM, 1.0 μM, 60 mM potassium cacodylate, pH 7.4	200	37,39
**156**	27.0	*HSP90A*	FRET	0.01	T-loop	FRET	0.20 μM, 2.0 μM, 60 mM potassium cacodylate, pH 7.4	2700	37,39
**156**	3.0	*HSP90B*	FRET	0.01	T-loop	FRET	0.20 μM, 1.0 μM, 60 mM potassium cacodylate, pH 7.4	300	37,39

**156**	23.0	*HSP90B*	FRET	0.01	T-loop	FRET	0.20 μM, 2.0 μM, 60 mM potassium cacodylate, pH 7.4	2300	[37,39]
**157**	28.0	Telomere	FRET	8.0	T-loop	FRET	0.20 μM, 1.0 μM, 60 mM potassium cacodylate, pH 7.4	3.5	[37,39]
**157**	33.0	Telomere	FRET	12.0	T-loop	FRET	0.20 μM, 2.0 μM, 60 mM potassium cacodylate, pH 7.4	2.8	[37,39]
**157**	32.0	*HSP90A*	FRET	8.0	T-loop	FRET	0.20 μM, 1.0 μM, 60 mM potassium cacodylate, pH 7.4	4.0	[37,39]
**157**	36.0	*HSP90A*	FRET	12.0	T-loop	FRET	0.20 μM, 2.0 μM, 60 mM potassium cacodylate, pH 7.4	3.0	[37,39]
**157**	31.0	*HSP90B*	FRET	8.0	T-loop	FRET	0.20 μM, 1.0 μM, 60 mM potassium cacodylate, pH 7.4	3.9	[37,39]
**157**	34.0	*HSP90B*	FRET	12.0	T-loop	FRET	0.20 μM, 2.0 μM, 60 mM potassium cacodylate, pH 7.4	2.8	[37,39]
**158**	29.0	Telomere	FRET	5.0	T-loop	FRET	0.20 μM, 1.0 μM, 60 mM potassium cacodylate, pH 7.4	5.8	[37,39]
**158**	34.0	Telomere	FRET	9.0	T-loop	FRET	0.20 μM, 2.0 μM, 60 mM potassium cacodylate, pH 7.4	3.8	[37,39]
**158**	34.0	*HSP90A*	FRET	5.0	T-loop	FRET	0.20 μM, 1.0 μM, 60 mM potassium cacodylate, pH 7.4	6.8	[37,39]
**158**	36.0	*HSP90A*	FRET	9.0	T-loop	FRET	0.20 μM, 2.0 μM, 60 mM potassium cacodylate, pH 7.4	4.0	[37,39]
**158**	30.0	*HSP90B*	FRET	5.0	T-loop	FRET	0.20 μM, 1.0 μM, 60 mM potassium cacodylate, pH 7.4	6.0	[37,39]
**158**	34.0	*HSP90B*	FRET	9.0	T-loop	FRET	0.20 μM, 2.0 μM, 60 mM potassium cacodylate, pH 7.4	3.8	[37,39]
**159**	27.0	Telomere	FRET	4.9	T-loop	FRET	0.20 μM, 1.0 μM, 60 mM potassium cacodylate, pH 7.4	5.5	[37–39]
**159**	32.0	Telomere	FRET	9.0	T-loop	FRET	0.20 μM, 2.0 μM, 60 mM potassium cacodylate, pH 7.4	3.6	[37–39]
**159**	33.0	*HSP90A*	FRET	4.9	T-loop	FRET	0.20 μM, 1.0 μM, 60 mM potassium cacodylate, pH 7.4	6.7	[37–39]
**159**	36.0	*HSP90A*	FRET	9.0	T-loop	FRET	0.20 μM, 2.0 μM, 60 mM potassium cacodylate, pH 7.4	4.0	[37–39]
**159**	29.0	*HSP90B*	FRET	4.9	T-loop	FRET	0.20 μM, 1.0 μM, 60 mM potassium cacodylate, pH 7.4	5.9	[37–39]
**159**	33.0	*HSP90B*	FRET	9.0	T-loop	FRET	0.20 μM, 2.0 μM, 60 mM potassium cacodylate, pH 7.4	3.7	[37–39]
**159**	>16.0	*hTERT*	UV	<2.0	duplex DNA	UV	5.0 μM, 5.0 μM, 50 mM KCl, 10 mM Tris-HCl, 1 mM EDTA, pH 7.4	>8.0	[31]
**159**	11.0	Telomere	UV	<2.0	duplex DNA	UV	5.0 μM, 5.0 μM, 50 mM KCl, 10 mM Tris-HCl, 1 mM EDTA, pH 7.4	>5.5	[31]
**159**	22.5	*kRAS21*	FRET	4.9	T-loop	FRET	0.20 μM, 1.0 μM, 60 mM potassium cacodylate, pH 7.4	4.6	[29]
**159**	19.8	*kRAS32*	FRET	4.9	T-loop	FRET	0.20 μM, 1.0 μM, 60 mM potassium cacodylate, pH 7.4	4.0	[29]
**159**	26.4	*bcl-2*	FRET	4.9	T-loop	FRET	0.20 μM, 1.0 μM, 60 mM potassium cacodylate, pH 7.4	5.4	[29]
**160**	24.7	Telomere	FRET	0.1	T-loop	FRET	0.20 μM, 1.0 μM, 60 mM potassium cacodylate, pH 7.4	247	[43]
**160**	4.9	*c-kit1*	FRET	0.1	T-loop	FRET	0.20 μM, 1.0 μM, 60 mM potassium cacodylate, pH 7.4	49	[43]
**160**	16.7	*c-kit2*	FRET	0.1	T-loop	FRET	0.20 μM, 1.0 μM, 60 mM potassium cacodylate, pH 7.4	167	[43]
**161**	23.8	Telomere	FRET	0.2	T-loop	FRET	0.20 μM, 1.0 μM, 60 mM potassium cacodylate, pH 7.4	119	[43]
**161**	1.5	*c-kit1*	FRET	0.2	T-loop	FRET	0.20 μM, 1.0 μM, 60 mM potassium cacodylate, pH 7.4	7.5	[43]
**161**	7.7	*c-kit2*	FRET	0.2	T-loop	FRET	0.20 μM, 1.0 μM, 60 mM potassium cacodylate, pH 7.4	38	[43]
**161**	3.8	*bcl-2*	FRET	0.2	T-loop	FRET	0.20 μM, 1.0 μM, 60 mM potassium cacodylate, pH 7.4	19	[43]
**162**	10.0	Telomere	UV	2.0	duplex DNA	UV	5.0 μM, 5.0 μM, 50 mM KCl, 10 mM Tris-HCl, 1 mM EDTA, pH 7.4	5.0	[31]

**162**	18.0	*hTERT*	UV	2.0	duplex DNA	UV	5.0 µM, 5.0 µM, 50 mM KCl, 10 mM Tris-HCl, 1 mM EDTA, pH 7.4	9.0	[31]
**163**	13.0	Telomere	FRET	n.d.	n.d.	n.d.	0.20 µM, 1.0 µM, 50 mM KCl, 10 mM lithium cacodylate, pH 7.4	n.d.	[48]
**164**	3.6	*AR1*	FRET	<0.1	dsDNA	FRET	0.25 µM, 1.0 µM, 100 mM KCl, 10 mM lithium cacodylate, pH 7.4	>36	[18]
**164**	6.0	*AR3*	FRET	<0.1	dsDNA	FRET	0.25 µM, 1.0 µM, 100 mM KCl, 10 mM lithium cacodylate, pH 7.4	>60	[18]
**165**	20.5	*AR1*	FRET	1.0	dsDNA	FRET	0.25 µM, 1.0 µM, 100 mM KCl, 10 mM lithium cacodylate, pH 7.4	20	[18]
**165**	31.0	*AR3*	FRET	1.0	dsDNA	FRET	0.25 µM, 1.0 µM, 100 mM KCl, 10 mM lithium cacodylate, pH 7.4	31	[18]
**166**	n.d.	n.d.	n.d.	n.d.	n.d.	n.d.	n.d.	n.d.	**-**
**167**	n.d.	n.d.	n.d.	n.d.	n.d.	n.d.	n.d.	n.d.	**-**
**168**	n.d.	n.d.	n.d.	n.d.	n.d.	n.d.	n.d.	n.d.	**-**
**169**	n.d.	n.d.	n.d.	n.d.	n.d.	n.d.	n.d.	n.d.	**-**
**170**	n.d.	n.d.	n.d.	n.d.	n.d.	n.d.	n.d.	n.d.	**-**
**171**	n.d.	n.d.	n.d.	n.d.	n.d.	n.d.	n.d.	n.d.	**-**
**172**	n.d.	n.d.	n.d.	n.d.	n.d.	n.d.	n.d.	n.d.	**-**
**173**	n.d.	n.d.	n.d.	n.d.	n.d.	n.d.	n.d.	n.d.	**-**
**174**	n.d.	n.d.	n.d.	n.d.	n.d.	n.d.	n.d.	n.d.	**-**
**175**	19.4	Telomere	FRET	0.1	FdxT	FRET	0.25 µM, 2.5 µM, 10 mM KCl, 90 mM LiCl, 10 mM lithium cacodylate, pH 7.2	194	[50]
**175**	10.0	*c-myc*	FRET	0.1	FdxT	FRET	*c-myc*: 0.25 µM, 2.5 µM, 1 mM KCl, 99 mM LiCl, 10 mM lithium cacodylate, pH 7.2 FdxT: 0.25 µM, 2.5 µM, 10 mM KCl, 90 mM LiCl, 10 mM lithium cacodylate, pH 7.2	100	[50]
**176**	n.d.	n.d.	n.d.	n.d.	n.d.	n.d.	n.d.	n.d.	**-**
**177**	n.d.	n.d.	n.d.	n.d.	n.d.	n.d.	n.d.	n.d.	**-**
**178**	n.d.	n.d.	n.d.	n.d.	n.d.	n.d.	n.d.	n.d.	**-**
**179**	24.0	Telomere	FRET	-0.1	FdxT	FRET	0.25 µM, 2.5 µM, 10 mM KCl, 90 mM LiCl, 10 mM lithium cacodylate, pH 7.2	-240	[50]
**179**	12.0	*c-myc*	FRET	-0.1	FdxT	FRET	*c-myc*: 0.25 µM, 2.5 µM, 1 mM KCl, 99 mM LiCl,10 mM lithium cacodylate, pH 7.2 FdxT: 0.25 µM, 2.5 µM, 10 mM KCl, 90 mM LiCl, 10 mM lithium cacodylate, pH 7.2	-120	[50]
**180**	n.d.	n.d.	n.d.	n.d.	n.d.	n.d.	n.d.	n.d.	**-**
**181**	n.d.	n.d.	n.d.	n.d.	n.d.	n.d.	n.d.	n.d.	**-**
**182**	0.01	Telomere	CD	n.d.	n.d.	n.d.	2.0 µM, 12 µM, 20 mM KCl, 5 mM potassium phosphate, pH 7.0	n.d.	[20]
**183**	n.d.	n.d.	n.d.	n.d.	n.d.	n.d.	n.d.	n.d.	**-**
**184**	n.d.	n.d.	n.d.	n.d.	n.d.	n.d.	n.d.	n.d.	**-**
**185**	19.0	Telomere	FRET	7.0	ds26	FRET	0.25 µM, 1.0 µM, 100 mM KCl, 10 mM lithium cacodylate, pH 7.4	2.7	[19]
**186**	8.3	*AR1*	FRET	1.0	dsDNA	FRET	0.25 µM, 1.0 µM, 100 mM KCl, 10 mM lithium cacodylate, pH 7.4	8.3	[18]
**186**	8.3	*AR1*	FRET	8.0	ds26	FRET	0.25 µM, 1.0 µM, 100 mM KCl, 10 mM lithium cacodylate, pH 7.4	1.0	[18,19]
**186**	17.0	*AR3*	FRET	1.0	dsDNA	FRET	0.25 µM, 1.0 µM, 100 mM KCl, 10 mM lithium cacodylate, pH 7.4	17	[18]
**186**	17.0	*AR3*	FRET	8.0	ds26	FRET	0.25 µM, 1.0 µM, 100 mM KCl, 10 mM lithium cacodylate, pH 7.4	2.1	[18,19]
**186**	22.0	Telomere	FRET	1.0	dsDNA	FRET	0.25 µM, 1.0 µM, 100 mM KCl, 10 mM lithium cacodylate, pH 7.4	22	[18,19]
**186**	22.0	Telomere	FRET	8.0	ds26	FRET	0.25 µM, 1.0 µM, 100 mM KCl, 10 mM lithium cacodylate, pH 7.4	2.8	[19]
**187**	18.0	Telomere	FRET	6.0	ds26	FRET	0.25 µM, 1.0 µM, 100 mM KCl, 10 mM lithium cacodylate, pH 7.4	3.0	[19]

**188**	19.0	Telomere	FRET	7.0	ds26	FRET	0.25 µM, 1.0 µM, 100 mM KCl, 10 mM lithium cacodylate, pH 7.4	2.7	19
**189**	21.0	Telomere	FRET	8.0	ds26	FRET	0.25 µM, 1.0 µM, 100 mM KCl, 10 mM lithium cacodylate, pH 7.4	2.6	19
**190**	14.0	Telomere	FRET	1.0	ds26	FRET	0.25 µM, 1.0 µM, 100 mM KCl, 10 mM lithium cacodylate, pH 7.4	14	19
**191**	19.0	Telomere	FRET	12.0	ds26	FRET	0.25 µM, 1.0 µM, 100 mM KCl, 10 mM lithium cacodylate, pH 7.4	1.6	19
**192**	14.0	Telomere	FRET	16.0	ds26	FRET	0.25 µM, 1.0 µM, 100 mM KCl, 10 mM lithium cacodylate, pH 7.4	0.88	19
**193**	19.0	Telomere	FRET	11.0	ds26	FRET	0.25 µM, 1.0 µM, 100 mM KCl, 10 mM lithium cacodylate, pH 7.4	1.7	19
**194**	12.0	Telomere	FRET	13.0	ds26	FRET	0.25 µM, 1.0 µM, 100 mM KCl, 10 mM lithium cacodylate, pH 7.4	0.92	19
**195**	13.0	Telomere	FRET	10.0	ds26	FRET	0.25 µM, 1.0 µM, 100 mM KCl, 10 mM lithium cacodylate, pH 7.4	1.3	19
**196**	10.0	Telomere	FRET	13.0	ds26	FRET	0.25 µM, 1.0 µM, 100 mM KCl, 10 mM lithium cacodylate, pH 7.4	0.77	19
**197**	n.d.	n.d.	n.d.	n.d.	n.d.	n.d.	n.d.	n.d.	**-**
**198**	n.d.	n.d.	n.d.	n.d.	n.d.	n.d.	n.d.	n.d.	**-**
**199**	12.6	Telomere	FRET	1.4	T-loop	FRET	0.20 µM, 1.0 µM, 60 mM potassium cacodylate, pH 7.4	9.0	11
**199**	12.2	*c-kit2*	FRET	1.4	T-loop	FRET	0.20 µM, 1.0 µM, 60 mM potassium cacodylate, pH 7.4	8.7	11
**200**	n.d.	n.d.	n.d.	n.d.	n.d.	n.d.	n.d.	n.d.	**-**
**201**	3.0	Telomere	CD	0.01	dsOligo	UV	1.5 µM, 1.5 µM, 100 mM KCl, 50 mM Tris-HCl, pH 7.4	300	4
**202**	14.0	Telomere	CD	n.d.	n.d.	n.d.	1.5 µM, 4.5 µM, 100 mM KCl, 50 mM Tris-HCl, pH 7.4	n.d.	51
**203**	15.0	Telomere	CD	0.3	dsDNA	UV	1.5 µM, 3.0 µM, 100 mM KCl, 50 mM Tris-HCl, pH 7.4	50	5
**203**	11.0	*c-kit2*	CD	0.3	dsDNA	UV	*c-kit2*: 1.5 µM, 3.0 µM, 20 mM KCl, 50 mM Tris-HCl, pH 7.4; dsDNA: 1.5 µM, 3.0 µM, 100 mM KCl, 50 mM Tris-HCl, pH 7.4	37	5
**203**	15.0	*c-myc*	CD	0.3	dsDNA	UV	*c-myc*: 1.5 µM, 3.0 µM, 5 mM KCl, 50 mM Tris-HCl, pH 7.4; dsDNA: 1.5 µM, 3.0 µM, 100 mM KCl, 50 mM Tris-HCl, pH 7.4	50	5
**204**	4.0	Telomere	CD	n.d.	n.d.	n.d.	1.5 µM, 1.5 µM, 30 mM KCl, 50 mM Tris-HCl, pH 7.4	n.d.	52
**204**	8.0	Telomere	CD	n.d.	n.d.	n.d.	1.5 µM, 3.0 µM, 30 mM KCl, 50 mM Tris-HCl, pH 7.4	n.d.	52
**204**	10.0	Telomere	CD	n.d.	n.d.	n.d.	1.5 µM, 4.5 µM, 30 mM KCl, 50 mM Tris-HCl, pH 7.4	n.d.	52
**205**	2.0	Telomere	CD	n.d.	n.d.	n.d.	1.5 µM, 4.5 µM, 100 mM KCl, 100 mM AcOK-AcOH, pH 5.5	n.d.	53
**206**	3.3	Telomere	CD	n.d.	n.d.	n.d.	1.5 µM, 4.5 µM, 100 mM KCl, 100 mM AcOK-AcOH, pH 5.5	n.d.	53
**207**	5.0	Telomere	CD	n.d.	n.d.	n.d.	1.5 µM, 4.5 µM, 100 mM KCl, 100 mM AcOK-AcOH, pH 5.5	n.d.	53
**208**	18.9	Telomere	FRET	1.2	T-loop	FRET	0.20 µM, 1.0 µM, 60 mM potassium cacodylate, pH 7.4	16	11
**208**	14.3	*c-kit2*	FRET	1.2	T-loop	FRET	0.20 µM, 1.0 µM, 60 mM potassium cacodylate, pH 7.4	12	11
**209**	22.1	Telomere	FRET	3.3	T-loop	FRET	0.20 µM, 1.0 µM, 60 mM potassium cacodylate, pH 7.4	6.7	11
**209**	15.1	*c-kit2*	FRET	3.3	T-loop	FRET	0.20 µM, 1.0 µM, 60 mM potassium cacodylate, pH 7.4	4.6	11
**210**	26.8	Telomere	FRET	8.6	T-loop	FRET	0.20 µM, 1.0 µM, 60 mM potassium cacodylate, pH 7.4	3.1	11
**210**	33.1	*c-kit2*	FRET	8.6	T-loop	FRET	0.20 µM, 1.0 µM, 60 mM potassium cacodylate, pH 7.4	3.8	11

**211**	0.4	Telomere	FRET	0.2	T-loop	FRET	0.20 µM, 1.0 µM, 60 mM potassium cacodylate, pH 7.4	2.0	[11]
**211**	0.01	*c-kit2*	FRET	0.2	T-loop	FRET	0.20 µM, 1.0 µM, 60 mM potassium cacodylate, pH 7.4	0.05	[11]
**212**	2.0	Telomere	CD	0.01	dsOligo	UV	1.5 µM, 1.5 µM, 100 mM KCl, 50 mM Tris-HCl, pH 7.4	200	[4]
**213**	9.0	Telomere	CD	10.0	dsDNA	UV	Telomere: 1.5 µM, 3.0 µM, 100 mM KCl, 50 mM Tris–HCl, pH 7.4<br>dsDNA: 3.2 µM, 6.4 µM, 100 mM KCl, 50 mM Tris–HCl, pH 7.4	0.90	[3]
**214**	10.0	Telomere	CD	n.d.	n.d.	n.d.	1.5 µM, 1.5 µM, 30 mM KCl, 50 mM Tris-HCl, pH 7.4	n.d.	[52]
**214**	16.0	Telomere	CD	n.d.	n.d.	n.d.	1.5 µM, 3.0 µM, 30 mM KCl, 50 mM Tris-HCl, pH 7.4	n.d.	[52]
**214**	18.0	Telomere	CD	n.d.	n.d.	n.d.	1.5 µM, 4.5 µM, 30 mM KCl, 50 mM Tris-HCl, pH 7.4	n.d.	[52]
**215**	10.0	Telomere	CD	n.d.	n.d.	n.d.	1.5 µM, 1.5 µM, 30 mM KCl, 50 mM Tris-HCl, pH 7.4	n.d.	[52]
**215**	14.0	Telomere	CD	n.d.	n.d.	n.d.	1.5 µM, 3.0 µM, 30 mM KCl, 50 mM Tris-HCl, pH 7.4	n.d.	[52]
**215**	15.0	Telomere	CD	n.d.	n.d.	n.d.	1.5 µM, 4.5 µM, 30 mM KCl, 50 mM Tris-HCl, pH 7.4	n.d.	[52]
**216**	9.0	Telomere	CD	n.d.	n.d.	n.d.	1.5 µM, 1.5 µM, 30 mM KCl, 50 mM Tris-HCl, pH 7.4	n.d.	[52]
**216**	14.0	Telomere	CD	n.d.	n.d.	n.d.	1.5 µM, 3.0 µM, 30 mM KCl, 50 mM Tris-HCl, pH 7.4	n.d.	[52]
**216**	15.0	Telomere	CD	n.d.	n.d.	n.d.	1.5 µM, 4.5 µM, 30 mM KCl, 50 mM Tris-HCl, pH 7.4	n.d.	[52]

Table S3. Summary of the binding constant (K_b) values obtained by biophysical techniques for the indicated DNA/NDI systems. n.d. = not defined; SPR = surface plasmon resonance; ITC = isothermal titration calorimetry.

Compound	G-quadruplex			Duplex			Experimental conditions (buffer composition)	K_b (G-quadruplex)/ K_b (duplex)	Refs.
	K_b (M^{-1})	DNA target	Technique	K_b (M^{-1})	DNA target	Technique			
**1**	n.d.	n.d.	n.d.	n.d.	n.d.	n.d.	n.d.	n.d.	-
**2**	$4.5\cdot10^{6}$	Telomere	SPR	$1.0\cdot10^{6}$	dsDNA	UV-vis	SPR: 100 mM KCl, 10 mM HEPES, 3 mM EDTA, 0.005% P20, pH 7.4; UV-vis: 100 mM KCl, 50 mM Tris-HCl, pH 7.4	4.5	1,3
**2**	$4.5\cdot10^{7}$	Telomere	SPR	$6.0\cdot10^{5}$	dsOligo	UV-vis	SPR: 100 mM KCl, 10 mM HEPES, 3 mM EDTA, 0.005% P20, pH 7.4; UV-vis: 100 mM KCl, 50 mM Tris-HCl, pH 7.4	7.5	1,4
**2**	$1.6\cdot10^{6}$	Telomere	UV-vis	$1.0\cdot10^{6}$	dsDNA	UV-vis	100 mM KCl, 50 mM Tris-HCl, pH 7.4	1.6	3,5
**2**	$1.6\cdot10^{6}$	Telomere	UV-vis	$6.0\cdot10^{5}$	dsOligo	UV-vis	100 mM KCl, 50 mM Tris-HCl, pH 7.4	2.7	4,5
**2**	$7.4\cdot10^{5}$	*c-kit1*	UV-vis	$1.0\cdot10^{6}$	dsDNA	UV-vis	100 mM KCl, 50 mM Tris-HCl, pH 7.4	0.74	3,5
**2**	$7.4\cdot10^{5}$	*c-kit1*	UV-vis	$6.0\cdot10^{5}$	dsOligo	UV-vis	100 mM KCl, 50 mM Tris-HCl, pH 7.4	1.2	4,5
**2**	$1.5\cdot10^{6}$	*c-myc*	UV-vis	$1.0\cdot10^{6}$	dsDNA	UV-vis	100 mM KCl, 50 mM Tris-HCl, pH 7.4	1.5	3,5
**2**	$1.5\cdot10^{6}$	*c-myc*	UV-vis	$6.0\cdot10^{5}$	dsOligo	UV-vis	100 mM KCl, 50 mM Tris-HCl, pH 7.4	2.5	4,5
**3**	n.d.	n.d.	n.d.	n.d.	n.d.	n.d.	n.d.	n.d.	-
**4**	n.d.	n.d.	n.d.	n.d.	n.d.	n.d.	n.d.	n.d.	-
**5**	n.d.	n.d.	n.d.	n.d.	n.d.	n.d.	n.d.	n.d.	-
**6**	n.d.	n.d.	n.d.	n.d.	n.d.	n.d.	n.d.	n.d.	-
**7**	n.d.	n.d.	n.d.	n.d.	n.d.	n.d.	n.d.	n.d.	-
**8**	n.d.	n.d.	n.d.	n.d.	n.d.	n.d.	n.d.	n.d.	-
**9**	n.d.	n.d.	n.d.	n.d.	n.d.	n.d.	n.d.	n.d.	-
**10**	n.d.	n.d.	n.d.	n.d.	n.d.	n.d.	n.d.	n.d.	-
**11**	n.d.	n.d.	n.d.	n.d.	n.d.	n.d.	n.d.	n.d.	-
**12**	n.d.	n.d.	n.d.	n.d.	n.d.	n.d.	n.d.	n.d.	-
**13**	n.d.	n.d.	n.d.	n.d.	n.d.	n.d.	n.d.	n.d.	-
**14**	n.d.	n.d.	n.d.	n.d.	n.d.	n.d.	n.d.	n.d.	-
**15**	n.d.	n.d.	n.d.	n.d.	n.d.	n.d.	n.d.	n.d.	-
**16**	n.d.	n.d.	n.d.	n.d.	n.d.	n.d.	n.d.	n.d.	-
**17**	n.d.	n.d.	n.d.	n.d.	n.d.	n.d.	n.d.	n.d.	-
**18**	n.d.	n.d.	n.d.	n.d.	n.d.	n.d.	n.d.	n.d.	-
**19**	n.d.	n.d.	n.d.	n.d.	n.d.	n.d.	n.d.	n.d.	-
**20**	n.d.	n.d.	n.d.	n.d.	n.d.	n.d.	n.d.	n.d.	-
**21**	n.d.	n.d.	n.d.	n.d.	n.d.	n.d.	n.d.	n.d.	-
**22**	n.d.	n.d.	n.d.	n.d.	n.d.	n.d.	n.d.	n.d.	-
**23**	n.d.	n.d.	n.d.	n.d.	n.d.	n.d.	n.d.	n.d.	-
**24**	n.d.	n.d.	n.d.	n.d.	n.d.	n.d.	n.d.	n.d.	-
**25**	n.d.	n.d.	n.d.	n.d.	n.d.	n.d.	n.d.	n.d.	-
**26**	n.d.	n.d.	n.d.	n.d.	n.d.	n.d.	n.d.	n.d.	-
**27**	n.d.	n.d.	n.d.	n.d.	n.d.	n.d.	n.d.	n.d.	-
**28**	n.d.	n.d.	n.d.	n.d.	n.d.	n.d.	n.d.	n.d.	-
**29**	n.d.	n.d.	n.d.	n.d.	n.d.	n.d.	n.d.	n.d.	-
**30**	$8.7\cdot10^{6}$	Telomere	SPR	$1.0\cdot10^{5}$	26-mer	SPR	100 mM KCl, 10 mM HEPES, 0.005% IGEPAL, pH 7.4	87	12
**30**	$1.0\cdot10^{6}$	Telomere	ITC	$1.0\cdot10^{5}$	26-mer	SPR	ITC: 100 mM potassium phosphate, pH 7.4; SPR: 100 mM KCl,	10	6,12

							10 mM HEPES, 0.005% IGEPAL, pH 7.4		
**31**	n.d.	n.d.	n.d.	n.d.	n.d.	n.d.	n.d.	n.d.	-
**32**	$2.2\cdot10^{6}$	Telomere	ITC	n.d.	n.d.	n.d.	100 mM potassium phosphate, pH 7.4	n.d.	[6]
**33**	n.d.	n.d.	n.d.	n.d.	n.d.	n.d.	n.d.	n.d.	-
**34**	n.d.	n.d.	n.d.	n.d.	n.d.	n.d.	n.d.	n.d.	-
**35**	$1.4\cdot10^{6}$	Telomere	SPR	$4.0\cdot10^{5}$	26-mer	SPR	100 mM KCl, 10 mM HEPES, 0.005% IGEPAL, pH 7.4	3.5	[12]
**36**	$9.5\cdot10^{7}$	Telomere	SPR	$6.0\cdot10^{5}$	26-mer	SPR	100 mM KCl, 10 mM HEPES, 0.005% IGEPAL, pH 7.4	158	[12]
**37**	n.d.	n.d.	n.d.	n.d.	n.d.	n.d.	n.d.	n.d.	-
**38**	n.d.	n.d.	n.d.	n.d.	n.d.	n.d.	n.d.	n.d.	-
**39**	$1.3\cdot10^{6}$	Telomere	ITC	n.d.	n.d.	n.d.	100 mM potassium phosphate, pH 7.4	n.d.	[6]
**40**	n.d.	n.d.	n.d.	n.d.	n.d.	n.d.	n.d.	n.d.	-
**41**	n.d.	n.d.	n.d.	n.d.	n.d.	n.d.	n.d.	n.d.	-
**42**	n.d.	n.d.	n.d.	n.d.	n.d.	n.d.	n.d.	n.d.	-
**43**	n.d.	n.d.	n.d.	n.d.	n.d.	n.d.	n.d.	n.d.	-
**44**	$2.4\cdot10^{7}$	*AR1*	SPR	n.d.	n.d.	n.d.	150 mM KCl, 10 mM HEPES, 3 mM EDTA, pH 7.4	n.d.	[18]
**45**	n.d.	n.d.	n.d.	n.d.	n.d.	n.d.	n.d.	n.d.	-
**46**	$2.4\cdot10^{7}$	Telomere	Fluorescence	$1.9\cdot10^{7}$	ds27	Fluorescence	20 mM KCl, 5 mM potassium phosphate, 10% DMSO, pH 7.0	1.3	[17]
**46**	$1.2\cdot10^{7}$	Telomere	Fluorescence	$1.9\cdot10^{7}$	ds27	Fluorescence	20 mM KCl, 5 mM potassium phosphate, pH 7.0	0.63	[20]
**46**	$7.0\cdot10^{6}$	Telomere	Fluorescence	$1.9\cdot10^{7}$	ds27	Fluorescence	20 mM KCl, 5 mM potassium phosphate, pH 7.0	0.37	[21]
**46**	$2.6\cdot10^{7}$	*c-myc*	Fluorescence	$1.9\cdot10^{7}$	ds27	Fluorescence	20 mM KCl, 5 mM potassium phosphate, 10% DMSO, pH 7.0	1.4	[17]
**47**	n.d.	n.d.	n.d.	n.d.	n.d.	n.d.	n.d.	n.d.	-
**48**	$1.1\cdot10^{7}$	Telomere	SPR	n.d.	n.d.	n.d.	100 mM KCl, 10 mM HEPES, 3 mM EDTA, 0.005% P20, pH 7.4	n.d.	[1]
**49**	n.d.	n.d.	n.d.	n.d.	n.d.	n.d.	n.d.	n.d.	-
**50**	n.d.	n.d.	n.d.	n.d.	n.d.	n.d.	n.d.	n.d.	-
**51**	n.d.	n.d.	n.d.	n.d.	n.d.	n.d.	n.d.	n.d.	-
**52**	$3.2\cdot10^{5}$	Telomere	SPR	n.d.	n.d.	n.d.	200 mM KCl, 10 mM HEPES, 0.005% Tween-20, pH 7.4	n.d.	[24]
**52**	$2.4\cdot10^{6}$	*hTERT*	SPR	n.d.	n.d.	n.d.	200 mM KCl, 10 mM HEPES, 0.005% Tween-20, pH 7.4	n.d.	[24]
**52**	$2.6\cdot10^{6}$	*c-myc*	SPR	n.d.	n.d.	n.d.	200 mM KCl, 10 mM HEPES, 0.005% Tween-20, pH 7.4	n.d.	[24]
**52**	$3.0\cdot10^{6}$	*bcl-2*	SPR	n.d.	n.d.	n.d.	200 mM KCl, 10 mM HEPES, 0.005% Tween-20, pH 7.4	n.d.	[24]
**52**	$2.9\cdot10^{7}$	*AR1*	SPR	n.d.	n.d.	n.d.	150 mM KCl, 10 mM HEPES, 3 mM EDTA, pH 7.4	n.d.	[18]
**53**	n.d.	n.d.	n.d.	n.d.	n.d.	n.d.	n.d.	n.d.	-
**54**	n.d.	n.d.	n.d.	n.d.	n.d.	n.d.	n.d.	n.d.	-
**55**	n.d.	n.d.	n.d.	n.d.	n.d.	n.d.	n.d.	n.d.	-
**56**	n.d.	n.d.	n.d.	n.d.	n.d.	n.d.	n.d.	n.d.	-
**57**	$2.3\cdot10^{7}$	*AR1*	SPR	n.d.	n.d.	n.d.	150 mM KCl, 10 mM HEPES, 3 mM EDTA, pH 7.4	n.d.	[18]
**58**	n.d.	n.d.	n.d.	n.d.	n.d.	n.d.	n.d.	n.d.	-
**59**	n.d.	n.d.	n.d.	n.d.	n.d.	n.d.	n.d.	n.d.	-
**60**	$4.8\cdot10^{5}$	Telomere	SPR	n.d.	n.d.	n.d.	200 mM KCl, 10 mM HEPES, 0.005% Tween-20, pH 7.4	n.d.	[27]
**61**	n.d.	n.d.	n.d.	n.d.	n.d.	n.d.	n.d.	n.d.	-
**62**	n.d.	n.d.	n.d.	n.d.	n.d.	n.d.	n.d.	n.d.	-
**63**	$1.1\cdot10^{6}$	Telomere	SPR	n.d.	n.d.	n.d.	200 mM KCl, 10 mM HEPES, 0.005% Tween-20, pH 7.4	n.d.	[27]
**64**	$4.7\cdot10^{7}$	*AR1*	SPR	n.d.	n.d.	n.d.	150 mM KCl, 10 mM HEPES, 3 mM EDTA, pH 7.4	n.d.	[18]

**64**	$2.3 \cdot 10^6$	Telomere	SPR	n.d.	n.d.	n.d.	200 mM KCl, 10 mM HEPES, 0.005% Tween-20, pH 7.4	n.d.	[27]
**65**	n.d.	n.d.	n.d.	n.d.	n.d.	n.d.	n.d.	n.d.	-
**66**	n.d.	n.d.	n.d.	n.d.	n.d.	n.d.	n.d.	n.d.	-
**67**	n.d.	n.d.	n.d.	n.d.	n.d.	n.d.	n.d.	n.d.	-
**68**	n.d.	n.d.	n.d.	n.d.	n.d.	n.d.	n.d.	n.d.	-
**69**	n.d.	n.d.	n.d.	n.d.	n.d.	n.d.	n.d.	n.d.	-
**70**	n.d.	n.d.	n.d.	n.d.	n.d.	n.d.	n.d.	n.d.	-
**71**	$2.8 \cdot 10^5$	Telomere	UV-vis	$2.1 \cdot 10^6$	ct-DNA	UV-vis	10 mM potassium phosphate, 50 mM NaCl, pH 7.4	0.13	[28]
**72**	n.d.	n.d.	n.d.	n.d.	n.d.	n.d.	n.d.	n.d.	-
**73**	n.d.	n.d.	n.d.	n.d.	n.d.	n.d.	n.d.	n.d.	-
**74**	n.d.	n.d.	n.d.	n.d.	n.d.	n.d.	n.d.	n.d.	-
**75**	$4.8 \cdot 10^4$	Telomere	UV-vis	$4.5 \cdot 10^5$	ct-DNA	UV-vis	10 mM potassium phosphate, 50 mM NaCl, pH 7.4	0.11	[28]
**76**	n.d.	n.d.	n.d.	n.d.	n.d.	n.d.	n.d.	n.d.	-
**77**	n.d.	n.d.	n.d.	n.d.	n.d.	n.d.	n.d.	n.d.	-
**78**	n.d.	n.d.	n.d.	n.d.	n.d.	n.d.	n.d.	n.d.	-
**79**	$1.5 \cdot 10^4$	Telomere	UV-vis	$2.3 \cdot 10^5$	ct-DNA	UV-vis	10 mM potassium phosphate, 50 mM NaCl, pH 7.4	0.065	[28]
**80**	n.d.	n.d.	n.d.	n.d.	n.d.	n.d.	n.d.	n.d.	-
**81**	n.d.	n.d.	n.d.	n.d.	n.d.	n.d.	n.d.	n.d.	-
**82**	n.d.	n.d.	n.d.	n.d.	n.d.	n.d.	n.d.	n.d.	-
**83**	n.d.	n.d.	n.d.	n.d.	n.d.	n.d.	n.d.	n.d.	-
**84**	n.d.	n.d.	n.d.	n.d.	n.d.	n.d.	n.d.	n.d.	-
**85**	n.d.	n.d.	n.d.	n.d.	n.d.	n.d.	n.d.	n.d.	-
**86**	n.d.	n.d.	n.d.	n.d.	n.d.	n.d.	n.d.	n.d.	-
**87**	n.d.	n.d.	n.d.	n.d.	n.d.	n.d.	n.d.	n.d.	-
**88**	n.d.	n.d.	n.d.	n.d.	n.d.	n.d.	n.d.	n.d.	-
**89**	n.d.	n.d.	n.d.	n.d.	n.d.	n.d.	n.d.	n.d.	-
**90**	n.d.	n.d.	n.d.	n.d.	n.d.	n.d.	n.d.	n.d.	-
**91**	n.d.	n.d.	n.d.	n.d.	n.d.	n.d.	n.d.	n.d.	-
**92**	n.d.	n.d.	n.d.	n.d.	n.d.	n.d.	n.d.	n.d.	-
**93**	$1.5 \cdot 10^7$	Telomere	Fluorescence	n.d.	n.d.	n.d.	50 mM KCl, 10 mM Tris-HCl, 1 mM EDTA, pH 7.3	n.d.	[30]
**93**	$1.0 \cdot 10^7$	Telomere	Fluorescence	n.d.	n.d.	n.d.	50 mM KCl, 10 mM Tris-HCl, pH 7.4	n.d.	[31]
**93**	$1.2 \cdot 10^7$	Telomere	SPR	n.d.	n.d.	n.d.	50 mM KCl, 10 mM Tris-HCl, 1 mM EDTA, 0.05% P20, pH 7.4	n.d.	[31]
**93**	$6.2 \cdot 10^7$	*hTERT*	Fluorescence	n.d.	n.d.	n.d.	50 mM KCl, 10 mM Tris-HCl, pH 7.4	n.d.	[31]
**93**	$8.3 \cdot 10^7$	*hTERT*	SPR	n.d.	n.d.	n.d.	50 mM KCl, 10 mM Tris-HCl, 1 mM EDTA, 0.05% P20, pH 7.4	n.d.	[31]
**94**	n.d.	n.d.	n.d.	n.d.	n.d.	n.d.	n.d.	n.d.	-
**95**	n.d.	n.d.	n.d.	n.d.	n.d.	n.d.	n.d.	n.d.	-
**96**	n.d.	n.d.	n.d.	n.d.	n.d.	n.d.	n.d.	n.d.	-
**97**	n.d.	n.d.	n.d.	n.d.	n.d.	n.d.	n.d.	n.d.	-
**98**	n.d.	n.d.	n.d.	n.d.	n.d.	n.d.	n.d.	n.d.	-
**99**	n.d.	n.d.	n.d.	n.d.	n.d.	n.d.	n.d.	n.d.	-
**100**	n.d.	n.d.	n.d.	n.d.	n.d.	n.d.	n.d.	n.d.	-
**101**	n.d.	n.d.	n.d.	n.d.	n.d.	n.d.	n.d.	n.d.	-
**102**	$4.0 \cdot 10^6$	Telomere	SPR	n.d.	n.d.	n.d.	100 mM KCl, 10 mM HEPES, 3 mM EDTA, 0.005% P20, pH 7.4	n.d.	[1]
**103**	n.d.	n.d.	n.d.	n.d.	n.d.	n.d.	n.d.	n.d.	-
**104**	n.d.	n.d.	n.d.	n.d.	n.d.	n.d.	n.d.	n.d.	-
**105**	n.d.	n.d.	n.d.	n.d.	n.d.	n.d.	n.d.	n.d.	-
**106**	$2.4 \cdot 10^7$	Telomere	SPR	n.d.	n.d.	n.d.	100 mM KCl, 10 mM HEPES, 3 mM EDTA, 0.005% P20, pH 7.4	n.d.	[1]
**107**	$3.1 \cdot 10^7$	Telomere	SPR	n.d.	n.d.	n.d.	100 mM KCl, 10 mM HEPES, 3 mM EDTA, 0.005% P20, pH 7.4	n.d.	[1]
**108**	$2.4 \cdot 10^7$	Telomere	UV-vis	$5.7 \cdot 10^7$	ds26	UV-vis	100 mM KCl, 10 mM potassium phosphate, pH 7.0	0.42	[33]

**108**	$1.6\cdot10^{6}$	*c-myc*	UV-vis	$5.7\cdot10^{7}$	ds26	UV-vis	100 mM KCl, 10 mM potassium phosphate, pH 7.0	0.028	33
**109**	n.d.	n.d.	n.d.	n.d.	n.d.	n.d.	n.d.	n.d.	-
**110**	n.d.	n.d.	n.d.	n.d.	n.d.	n.d.	n.d.	n.d.	-
**111**	n.d.	n.d.	n.d.	n.d.	n.d.	n.d.	n.d.	n.d.	-
**112**	n.d.	n.d.	n.d.	n.d.	n.d.	n.d.	n.d.	n.d.	-
**113**	n.d.	n.d.	n.d.	n.d.	n.d.	n.d.	n.d.	n.d.	-
**114**	n.d.	n.d.	n.d.	n.d.	n.d.	n.d.	n.d.	n.d.	-
**115**	n.d.	n.d.	n.d.	n.d.	n.d.	n.d.	n.d.	n.d.	-
**116**	n.d.	n.d.	n.d.	n.d.	n.d.	n.d.	n.d.	n.d.	-
**117**	$2.6\cdot10^{7}$	Telomere	SPR	n.d.	n.d.	n.d.	200 mM KCl, 10 mM HEPES, 0.005% Tween-20, pH 7.4	n.d.	27
**118**	n.d.	n.d.	n.d.	n.d.	n.d.	n.d.	n.d.	n.d.	-
**119**	n.d.	n.d.	n.d.	n.d.	n.d.	n.d.	n.d.	n.d.	-
**120**	n.d.	n.d.	n.d.	n.d.	n.d.	n.d.	n.d.	n.d.	-
**121**	n.d.	n.d.	n.d.	n.d.	n.d.	n.d.	n.d.	n.d.	-
**122**	n.d.	n.d.	n.d.	n.d.	n.d.	n.d.	n.d.	n.d.	-
**123**	n.d.	n.d.	n.d.	n.d.	n.d.	n.d.	n.d.	n.d.	-
**124**	n.d.	n.d.	n.d.	n.d.	n.d.	n.d.	n.d.	n.d.	-
**125**	n.d.	n.d.	n.d.	n.d.	n.d.	n.d.	n.d.	n.d.	-
**126**	n.d.	n.d.	n.d.	n.d.	n.d.	n.d.	n.d.	n.d.	-
**127**	n.d.	n.d.	n.d.	n.d.	n.d.	n.d.	n.d.	n.d.	-
**128**	n.d.	n.d.	n.d.	n.d.	n.d.	n.d.	n.d.	n.d.	-
**129**	$1.7\cdot10^{6}$	Telomere	Fluorescence	$8.4\cdot10^{5}$	ds26	Fluorescence	100 mM KCl, 10 mM Tris-HCl, pH 7.4	2.0	36
**129**	$3.0\cdot10^{6}$	*c-kit1*	Fluorescence	$8.4\cdot10^{5}$	ds26	Fluorescence	100 mM KCl, 10 mM Tris-HCl, pH 7.4	3.6	36
**129**	$1.7\cdot10^{6}$	*c-kit2*	Fluorescence	$8.4\cdot10^{5}$	ds26	Fluorescence	100 mM KCl, 10 mM Tris-HCl, pH 7.4	2.0	36
**129**	$2.5\cdot10^{6}$	*c-myc*	Fluorescence	$8.4\cdot10^{5}$	ds26	Fluorescence	100 mM KCl, 10 mM Tris-HCl, pH 7.4	3.0	36
**130**	$3.2\cdot10^{6}$	Telomere	Fluorescence	n.d.	ds26	Fluorescence	100 mM KCl, 10 mM Tris-HCl, pH 7.4	n.d.	36
**130**	$4.8\cdot10^{6}$	*c-kit1*	Fluorescence	n.d.	ds26	Fluorescence	100 mM KCl, 10 mM Tris-HCl, pH 7.4	n.d.	36
**130**	$1.5\cdot10^{6}$	*c-kit2*	Fluorescence	n.d.	ds26	Fluorescence	100 mM KCl, 10 mM Tris-HCl, pH 7.4	n.d.	36
**130**	$1.9\cdot10^{7}$	*c-myc*	Fluorescence	n.d.	ds26	Fluorescence	100 mM KCl, 10 mM Tris-HCl, pH 7.4	n.d.	36
**131**	$1.4\cdot10^{6}$	Telomere	Fluorescence	n.d.	ds26	Fluorescence	100 mM KCl, 10 mM Tris-HCl, pH 7.4	n.d.	36
**131**	$6.9\cdot10^{6}$	*c-kit1*	Fluorescence	n.d.	ds26	Fluorescence	100 mM KCl, 10 mM Tris-HCl, pH 7.4	n.d.	36
**131**	$1.8\cdot10^{6}$	*c-kit2*	Fluorescence	n.d.	ds26	Fluorescence	100 mM KCl, 10 mM Tris-HCl, pH 7.4	n.d.	36
**131**	$2.1\cdot10^{6}$	*c-myc*	Fluorescence	n.d.	ds26	Fluorescence	100 mM KCl, 10 mM Tris-HCl, pH 7.4	n.d.	36
**132**	$1.5\cdot10^{7}$	Telomere	Fluorescence	n.d.	ds26	Fluorescence	100 mM KCl, 10 mM Tris-HCl, pH 7.4	n.d.	36
**132**	$5.8\cdot10^{5}$	*c-kit1*	Fluorescence	n.d.	ds26	Fluorescence	100 mM KCl, 10 mM Tris-HCl, pH 7.4	n.d.	36
**132**	$1.8\cdot10^{6}$	*c-kit2*	Fluorescence	n.d.	ds26	Fluorescence	100 mM KCl, 10 mM Tris-HCl, pH 7.4	n.d.	36
**132**	$5.5\cdot10^{5}$	*c-myc*	Fluorescence	n.d.	ds26	Fluorescence	100 mM KCl, 10 mM Tris-HCl, pH 7.4	n.d.	36
**133**	$1.5\cdot10^{6}$	Telomere	Fluorescence	n.d.	ds26	Fluorescence	100 mM KCl, 10 mM Tris-HCl, pH 7.4	n.d.	36
**133**	$1.8\cdot10^{6}$	*c-kit1*	Fluorescence	n.d.	ds26	Fluorescence	100 mM KCl, 10 mM Tris-HCl, pH 7.4	n.d.	36
**133**	$1.7\cdot10^{6}$	*c-kit2*	Fluorescence	n.d.	ds26	Fluorescence	100 mM KCl, 10 mM Tris-HCl, pH 7.4	n.d.	36
**133**	$4.4\cdot10^{6}$	*c-myc*	Fluorescence	n.d.	ds26	Fluorescence	100 mM KCl, 10 mM Tris-HCl, pH 7.4	n.d.	36

**134**	$1.4\cdot10^{6}$	Telomere	Fluorescence	$9.4\cdot10^{6}$	ds26	Fluorescence	100 mM KCl, 10 mM Tris-HCl, pH 7.4	0.15	36
**134**	$2.0\cdot10^{6}$	*c-kit1*	Fluorescence	$9.4\cdot10^{6}$	ds26	Fluorescence	100 mM KCl, 10 mM Tris-HCl, pH 7.4	0.21	36
**134**	$1.8\cdot10^{6}$	*c-kit2*	Fluorescence	$9.4\cdot10^{6}$	ds26	Fluorescence	100 mM KCl, 10 mM Tris-HCl, pH 7.4	0.19	36
**134**	$6.7\cdot10^{6}$	*c-myc*	Fluorescence	$9.4\cdot10^{6}$	ds26	Fluorescence	100 mM KCl, 10 mM Tris-HCl, pH 7.4	0.71	36
**135**	$5.8\cdot10^{5}$	Telomere	Fluorescence	$4.6\cdot10^{5}$	ds26	Fluorescence	100 mM KCl, 10 mM Tris-HCl, pH 7.4	1.3	36
**135**	n.d.	*c-kit1*	Fluorescence	$4.6\cdot10^{5}$	ds26	Fluorescence	100 mM KCl, 10 mM Tris-HCl, pH 7.4	n.d.	36
**135**	$7.5\cdot10^{5}$	*c-kit2*	Fluorescence	$4.6\cdot10^{5}$	ds26	Fluorescence	100 mM KCl, 10 mM Tris-HCl, pH 7.4	1.6	36
**135**	$9.2\cdot10^{5}$	*c-myc*	Fluorescence	$4.6\cdot10^{5}$	ds26	Fluorescence	100 mM KCl, 10 mM Tris-HCl, pH 7.4	2.0	36
**136**	$3.2\cdot10^{5}$	Telomere	Fluorescence	$4.3\cdot10^{6}$	ds26	Fluorescence	100 mM KCl, 10 mM Tris-HCl, pH 7.4	0.074	36
**136**	n.d.	*c-kit1*	Fluorescence	$4.3\cdot10^{6}$	ds26	Fluorescence	100 mM KCl, 10 mM Tris-HCl, pH 7.4	n.d.	36
**136**	$5.8\cdot10^{5}$	*c-kit2*	Fluorescence	$4.3\cdot10^{6}$	ds26	Fluorescence	100 mM KCl, 10 mM Tris-HCl, pH 7.4	0.13	36
**136**	$3.6\cdot10^{6}$	*c-myc*	Fluorescence	$4.3\cdot10^{6}$	ds26	Fluorescence	100 mM KCl, 10 mM Tris-HCl, pH 7.4	0.83	36
**137**	$7.1\cdot10^{6}$	Telomere	SPR	n.d.	n.d.	n.d.	100 mM KCl, 10 mM HEPES, 3 mM EDTA, 0.005% P20, pH 7.4	n.d.	1
**138**	n.d.	n.d.	n.d.	n.d.	n.d.	n.d.	n.d.	n.d.	-
**139**	n.d.	n.d.	n.d.	n.d.	n.d.	n.d.	n.d.	n.d.	-
**140**	n.d.	n.d.	n.d.	n.d.	n.d.	n.d.	n.d.	n.d.	-
**141**	n.d.	n.d.	n.d.	n.d.	n.d.	n.d.	n.d.	n.d.	-
**142**	n.d.	n.d.	n.d.	n.d.	n.d.	n.d.	n.d.	n.d.	-
**143**	n.d.	n.d.	n.d.	n.d.	n.d.	n.d.	n.d.	n.d.	-
**144**	n.d.	n.d.	n.d.	n.d.	n.d.	n.d.	n.d.	n.d.	-
**145**	n.d.	n.d.	n.d.	n.d.	n.d.	n.d.	n.d.	n.d.	-
**146**	n.d.	n.d.	n.d.	n.d.	n.d.	n.d.	n.d.	n.d.	-
**147**	n.d.	n.d.	n.d.	n.d.	n.d.	n.d.	n.d.	n.d.	-
**148**	n.d.	n.d.	n.d.	n.d.	n.d.	n.d.	n.d.	n.d.	-
**149**	n.d.	n.d.	n.d.	n.d.	n.d.	n.d.	n.d.	n.d.	-
**150**	n.d.	n.d.	n.d.	n.d.	n.d.	n.d.	n.d.	n.d.	-
**151**	n.d.	n.d.	n.d.	n.d.	n.d.	n.d.	n.d.	n.d.	-
**152**	$8.6\cdot10^{6}$	Telomere	SPR	n.d.	n.d.	n.d.	100 mM KCl, 10 mM HEPES, 3 mM EDTA, 0.005% P20, pH 7.4	n.d.	1
**153**	$2.7\cdot10^{6}$	Telomere	SPR	$8.0\cdot10^{5}$	26-mer	SPR	100 mM KCl, 10 mM HEPES, 0.005% IGEPAL, pH 7.4	3.4	12
**154**	n.d.	n.d.	n.d.	n.d.	n.d.	n.d.	n.d.	n.d.	-
**155**	n.d.	n.d.	n.d.	n.d.	n.d.	n.d.	n.d.	n.d.	-
**156**	n.d.	n.d.	n.d.	n.d.	n.d.	n.d.	n.d.	n.d.	-
**157**	n.d.	n.d.	n.d.	n.d.	n.d.	n.d.	n.d.	n.d.	-
**158**	n.d.	n.d.	n.d.	n.d.	n.d.	n.d.	n.d.	n.d.	-
**159**	$5.0\cdot10^{7}$	Telomere	Fluorescence	n.d.	n.d.	n.d.	50 mM KCl, 10 mM Tris-HCl, 1 mM EDTA, pH 7.3	n.d.	30
**159**	$1.0\cdot10^{8}$	Telomere	Fluorescence	n.d.	n.d.	n.d.	50 mM KCl, 10 mM Tris-HCl, 1 mM EDTA, pH 7.3	n.d.	31
**159**	$2.0\cdot10^{8}$	Telomere	SPR	n.d.	n.d.	n.d.	50 mM KCl, 10 mM Tris-HCl, 1 mM EDTA, 0.05% P20, pH 7.4	n.d.	31
**159**	$2.5\cdot10^{8}$	*hTERT*	Fluorescence	n.d.	n.d.	n.d.	50 mM KCl, 10 mM Tris-HCl, 1 mM EDTA, pH 7.3	n.d.	31
**159**	$5.0\cdot10^{8}$	*hTERT*	SPR	n.d.	n.d.	n.d.	50 mM KCl, 10 mM Tris-HCl, 1 mM EDTA, 0.05% P20, pH 7.4	n.d.	31
**160**	n.d.	n.d.	n.d.	n.d.	n.d.	n.d.	n.d.	n.d.	-
**161**	n.d.	n.d.	n.d.	n.d.	n.d.	n.d.	n.d.	n.d.	-
**162**	$3.6\cdot10^{7}$	Telomere	SPR	n.d.	n.d.	n.d.	50 mM KCl, 10 mM Tris-HCl, 1 mM EDTA, 0.05% P20, pH 7.4	n.d.	31

**162**	$3.4\cdot10^{8}$	Telomere	Fluorescence	n.d.	n.d.	n.d.	50 mM KCl, 10 mM Tris-HCl, pH 7.4	n.d.	31
**162**	$2.5\cdot10^{8}$	*hTERT*	SPR	n.d.	n.d.	n.d.	50 mM KCl, 10 mM Tris-HCl, 1 mM EDTA, 0.05% P20, pH 7.4	n.d.	31
**162**	$2.5\cdot10^{8}$	*hTERT*	Fluorescence	n.d.	n.d.	n.d.	50 mM KCl, 10 mM Tris-HCl, pH 7.4	n.d.	31
**163**	n.d.	n.d.	n.d.	n.d.	n.d.	n.d.	n.d.	n.d.	-
**164**	$2.6\cdot10^{7}$	*AR1*	SPR	n.d.	n.d.	n.d.	150 mM KCl 10 mM HEPES, 3 mM EDTA, pH 7.4	n.d.	18
**165**	$5.6\cdot10^{7}$	*AR1*	SPR	n.d.	n.d.	n.d.	150 mM KCl 10 mM HEPES, 3 mM EDTA, pH 7.4	n.d.	18
**166**	n.d.	n.d.	n.d.	n.d.	n.d.	n.d.	n.d.	n.d.	-
**167**	n.d.	n.d.	n.d.	n.d.	n.d.	n.d.	n.d.	n.d.	-
**168**	n.d.	n.d.	n.d.	n.d.	n.d.	n.d.	n.d.	n.d.	-
**169**	n.d.	n.d.	n.d.	n.d.	n.d.	n.d.	n.d.	n.d.	-
**170**	n.d.	n.d.	n.d.	n.d.	n.d.	n.d.	n.d.	n.d.	-
**171**	n.d.	n.d.	n.d.	n.d.	n.d.	n.d.	n.d.	n.d.	-
**172**	n.d.	n.d.	n.d.	n.d.	n.d.	n.d.	n.d.	n.d.	-
**173**	n.d.	n.d.	n.d.	n.d.	n.d.	n.d.	n.d.	n.d.	-
**174**	n.d.	n.d.	n.d.	n.d.	n.d.	n.d.	n.d.	n.d.	-
**175**	$2.5\cdot10^{6}$	Telomere	Fluorescence	$3.2\cdot10^{6}$	ds26	Fluorescence	100 mM KCl, 10 mM Tris-HCl, pH 7.2	0.78	50
**176**	n.d.	n.d.	n.d.	n.d.	n.d.	n.d.	n.d.	n.d.	-
**177**	n.d.	n.d.	n.d.	n.d.	n.d.	n.d.	n.d.	n.d.	-
**178**	n.d.	n.d.	n.d.	n.d.	n.d.	n.d.	n.d.	n.d.	-
**179**	$2.2\cdot10^{4}$	Telomere	Fluorescence	$2.8\cdot10^{3}$	ds26	Fluorescence	100 mM KCl, 10 mM Tris-HCl, pH 7.2	7.9	50
**179**	$4.1\cdot10^{6}$	*c-myc*	Fluorescence	$2.8\cdot10^{3}$	ds26	Fluorescence	100 mM KCl, 10 mM Tris-HCl, pH 7.2	1464	50
**179**	$1.0\cdot10^{5}$	*kRAS*	Fluorescence	$2.8\cdot10^{3}$	ds26	Fluorescence	100 mM KCl, 10 mM Tris-HCl, pH 7.2	36	50
**180**	n.d.	n.d.	n.d.	n.d.	n.d.	n.d.	n.d.	n.d.	-
**181**	n.d.	n.d.	n.d.	n.d.	n.d.	n.d.	n.d.	n.d.	-
**182**	n.d.	n.d.	n.d.	n.d.	n.d.	n.d.	n.d.	n.d.	-
**183**	n.d.	n.d.	n.d.	n.d.	n.d.	n.d.	n.d.	n.d.	-
**184**	n.d.	n.d.	n.d.	n.d.	n.d.	n.d.	n.d.	n.d.	-
**185**	n.d.	n.d.	n.d.	n.d.	n.d.	n.d.	n.d.	n.d.	-
**186**	$5.5\cdot10^{6}$	*AR1*	SPR	n.d.	n.d.	n.d.	150 mM KCl, 10 mM HEPES, 3 mM EDTA, pH 7.4	n.d.	18
**187**	n.d.	n.d.	n.d.	n.d.	n.d.	n.d.	n.d.	n.d.	-
**188**	n.d.	n.d.	n.d.	n.d.	n.d.	n.d.	n.d.	n.d.	-
**189**	n.d.	n.d.	n.d.	n.d.	n.d.	n.d.	n.d.	n.d.	-
**190**	n.d.	n.d.	n.d.	n.d.	n.d.	n.d.	n.d.	n.d.	-
**191**	n.d.	n.d.	n.d.	n.d.	n.d.	n.d.	n.d.	n.d.	-
**192**	n.d.	n.d.	n.d.	n.d.	n.d.	n.d.	n.d.	n.d.	-
**193**	n.d.	n.d.	n.d.	n.d.	n.d.	n.d.	n.d.	n.d.	-
**194**	n.d.	n.d.	n.d.	n.d.	n.d.	n.d.	n.d.	n.d.	-
**195**	n.d.	n.d.	n.d.	n.d.	n.d.	n.d.	n.d.	n.d.	-
**196**	n.d.	n.d.	n.d.	n.d.	n.d.	n.d.	n.d.	n.d.	-
**197**	n.d.	n.d.	n.d.	n.d.	n.d.	n.d.	n.d.	n.d.	-
**198**	n.d.	n.d.	n.d.	n.d.	n.d.	n.d.	n.d.	n.d.	-
**199**	n.d.	n.d.	n.d.	n.d.	n.d.	n.d.	n.d.	n.d.	-
**200**	$3.4\cdot10^{6}$	Telomere	ITC	n.d.	n.d.	n.d.	50 mM potassium phosphate, pH 7.0	n.d.	51
**200**	$1.1\cdot10^{6}$	*c-myc*	ITC	n.d.	n.d.	n.d.	50 mM potassium phosphate, pH 7.0	n.d.	51
**201**	$8.6\cdot10^{6}$	Telomere	UV-vis	$3.3\cdot10^{4}$	dsOligo	UV-vis	100 mM KCl, 50 mM Tris-HCl, pH 7.4	261	4
**201**	$1.5\cdot10^{6}$	Telomere	ITC	$3.3\cdot10^{4}$	dsOligo	UV-vis	ITC: 50 mM potassium phosphate, pH 7.0 UV-vis: 100 mM KCl, 50 mM Tris-HCl, pH 7.4	46	4,51

**201**	$6.2\cdot10^{6}$	*c-myc*	ITC	$3.3\cdot10^{4}$	dsOligo	UV-vis	ITC: 50 mM potassium phosphate, pH 7.0<br>UV-vis: 100 mM KCl, 50 mM Tris-HCl, pH 7.4	188	[4,51]
**202**	$2.2\cdot10^{6}$	Telomere	ITC	n.d.	n.d.	n.d.	50 mM potassium phosphate, pH 7.0	n.d.	[51]
**202**	$2.4\cdot10^{6}$	*c-myc*	ITC	n.d.	n.d.	n.d.	50 mM potassium phosphate, pH 7.0	n.d.	[51]
**203**	$1.0\cdot10^{7}$	Telomere	UV-vis	$3.7\cdot10^{4}$	dsDNA	UV-vis	100 mM KCl, 50 mM Tris-HCl, pH 7.4	270	[5]
**203**	$1.9\cdot10^{6}$	*c-kit1*	UV-vis	$3.7\cdot10^{4}$	dsDNA	UV-vis	100 mM KCl, 50 mM Tris-HCl, pH 7.4	51	[5]
**203**	$4.0\cdot10^{6}$	*c-myc*	UV-vis	$3.7\cdot10^{4}$	dsDNA	UV-vis	100 mM KCl, 50 mM Tris-HCl, pH 7.4	108	[5]
**204**	$2.1\cdot10^{6}$	Telomere	ITC	n.d.	n.d.	n.d.	50 mM potassium phosphate, pH 7.0	n.d.	[52]
**205**	$1.5\cdot10^{5}$	Telomere	ITC	n.d.	n.d.	n.d.	100 mM KCl, 100 mM AcOK-AcOH, pH 5.5	n.d.	[53]
**205**	$8.7\cdot10^{5}$	*c-myc*	ITC	n.d.	n.d.	n.d.	100 mM KCl, 100 mM AcOK-AcOH, pH 5.5	n.d.	[53]
**206**	$1.3\cdot10^{6}$	Telomere	ITC	n.d.	n.d.	n.d.	100 mM KCl, 100 mM AcOK-AcOH, pH 5.5	n.d.	[53]
**206**	$1.2\cdot10^{6}$	*c-myc*	ITC	n.d.	n.d.	n.d.	100 mM KCl, 100 mM AcOK-AcOH, pH 5.5	n.d.	[53]
**207**	$1.1\cdot10^{6}$	Telomere	ITC	n.d.	n.d.	n.d.	100 mM KCl, 100 mM AcOK-AcOH, pH 5.5	n.d.	[53]
**207**	$1.8\cdot10^{6}$	*c-myc*	ITC	n.d.	n.d.	n.d.	100 mM KCl, 100 mM AcOK-AcOH, pH 5.5	n.d.	[53]
**208**	n.d.	n.d.	n.d.	n.d.	n.d.	n.d.	n.d.	n.d.	-
**209**	n.d.	n.d.	n.d.	n.d.	n.d.	n.d.	n.d.	n.d.	-
**210**	n.d.	n.d.	n.d.	n.d.	n.d.	n.d.	n.d.	n.d.	-
**211**	n.d.	n.d.	n.d.	n.d.	n.d.	n.d.	n.d.	n.d.	-
**212**	$1.5\cdot10^{6}$	Telomere	UV-vis	$3.0\cdot10^{4}$	dsOligo	UV-vis	100 mM KCl, 50 mM Tris-HCl, pH 7.4	50	[4]
**213**	$3.7\cdot10^{6}$	Telomere	UV-vis	$2.6\cdot10^{6}$	dsDNA	UV-vis	100 mM KCl, 50 mM Tris-HCl, pH 7.4	1.4	[3]
**214**	$4.2\cdot10^{6}$	Telomere	ITC	n.d.	n.d.	n.d.	50 mM potassium phosphate, pH 7.0	n.d.	[52]
**215**	$4.5\cdot10^{6}$	Telomere	ITC	n.d.	n.d.	n.d.	50 mM potassium phosphate, pH 7.0	n.d.	[52]
**216**	$3.9\cdot10^{6}$	Telomere	ITC	n.d.	n.d.	n.d.	50 mM potassium phosphate, pH 7.0	n.d.	[52]

Table S4. Summary of the binding stoichiometries obtained by biophysical techniques for the indicated DNA/NDI systems.

Compound	DNA target	Binding stoichiometry (DNA:NDI)	Refs.
**2**	**Telomeric, *c-myc* and *c-kit* G-quadruplexes**	**1:2**	[3]
	**Duplex**	**1:3**	[3]
**18**	**Telomeric G-quadruplex**	**1:2**	[10]
	**Duplex**	**1:1**	[10]
**20**	**Telomeric G-quadruplex**	**1:2**	[10]
	**Duplex**	**1:1**	[10]
**23**	**Telomeric G-quadruplex**	**1:2**	[10]
	**Duplex**	**1:1**	[10]
**30**	**Telomeric G-quadruplex**	**1:2**	[6]
**32**	**Telomeric G-quadruplex**	**1:2**	[6]
**39**	**Telomeric G-quadruplex**	**1:3**	[6]
**46**	**Telomeric G-quadruplex,<br>telomeric G-quadruplex dimer,<br>*c-myc* G-quadruplex**	**1:3 or 1:4,<br>1:9,<br>1:3**	[17,20,21]
	**Duplex**	**1:5**	[17]
**129**	**Telomeric, *c-myc* and *c-kit* G-quadruplexes**	**1:2**	[36]
	**Duplex**	**1:1**	[36]
**130**	**Telomeric, *c-myc* and *c-kit* G-quadruplexes**	**1:2**	[36]
	**Duplex**	**1:1**	[36]
**131**	**Telomeric, *c-myc* and *c-kit* G-quadruplexes**	**1:2**	[36]
	**Duplex**	**1:1**	[36]

**132**	**Telomeric, *c-myc* and *c-kit* G-quadruplexes**	**1:2**	[36]
	**Duplex**	**1:1**	[36]
**133**	**Telomeric, *c-myc* and *c-kit* G-quadruplexes**	**1:2**	[36]
	**Duplex**	**1:1**	[36]
**134**	**Telomeric, *c-myc* and *c-kit* G-quadruplexes**	**1:2**	[36]
	**Duplex**	**1:1**	[36]
**135**	**Telomeric, *c-myc* and *c-kit* G-quadruplexes**	**1:2**	[36]
	**Duplex**	**1:1**	[36]
**136**	**Telomeric, *c-myc* and *c-kit* G-quadruplexes**	**1:2**	[36]
	**Duplex**	**1:1**	[36]
**163**	**Telomeric G-quadruplex**	**1:3**	[48]
**175**	**Telomeric G-quadruplex**	**1:2**	[50]
	**Duplex**	**1:2**	[50]
**179**	**Telomeric, *c-myc* and *c-kit* G-quadruplexes**	**1:1**	[50]
	**Duplex**	**1:1**	[50]
**200**	**Telomeric G-quadruplex,**<br>***c-myc* G-quadruplex**	**1:2,**<br>**1:1**	[51]
**201**	**Telomeric and *c-myc* G-quadruplexes**	**1:1**	[4]
**202**	**Telomeric and *c-myc* G-quadruplexes**	**1:2**	[51]
**203**	**Telomeric and *c-myc* G-quadruplexes,**<br>***c-kit* G-quadruplex**	**1:2,**<br>**1:1**	[5]
**204**	**Telomeric G-quadruplex**	**1:2**	[52]
**205**	**Telomeric and *c-myc* G-quadruplexes**	**1:2**	[53]

**206**	**Telomeric and *c-myc* G-quadruplexes**	**1:2**	[53]
**207**	**Telomeric and *c-myc* G-quadruplexes**	**1:2**	[53]
**213**	**Telomeric G-quadruplex**	**1:2**	[3]
	**Duplex**	**1:3**	[3]
**214**	**Telomeric G-quadruplex**	**1:1**	[52]
**215**	**Telomeric G-quadruplex**	**1:1**	[52]
**216**	**Telomeric G-quadruplex**	**1:1**	[52]

Table S5. Overview of the in vitro studies on NDIs. Cancer cell lines and related cancer type: MCF-7, MDA-MB32, MDA-MB321, MDA-MB468, T47-D = breast cancer; HCT116, HT-29, HT-29D = colon cancer; ADR-RES, OVCAR-4 = ovarian cancer; A549, H69, H522, HOP-92, HOP-62 = lung cancer; CAKI-1, RCC4, TK-10, UO-31, 786-O = renal cancer; BxPc-3, HPAC, MIA-Pa-Ca-2, PANC-1, GemMIA-R3, Capan-1 = pancreatic cancer; DU145, LNCaP, PC-3, PNT1A, 22Rv1: prostate cancer; HGC-27 = gastric cancer; GIST48, GIST62, GIST882 = gastrointestinal cancer; K562, MOLT-4, SR = leukaemia; A375, SKMel-5 = melanoma; U2OS = osteosarcoma; U251MG = glioblastoma; HeLa = cervical cancer; ALT = alternative lengthening of telomeres. Normal cell lines and related tissue: MRC-5, WI38 = lung; AGO1522, ASF-4-4L2, HFF = skin; HEK293T= kidney. n.d. = not defined.

Compound	Cancer cells		Normal cells		IC_{50} (normal cells)/ IC_{50} (cancer cells)	Additional in vitro properties	Refs.
	IC_{50} (nM)	Cell line	IC_{50} (nM)	Cell line			
**1**	119	MCF-7	171	WI38	1.44	n.d.	1,2
**1**	99	A549	171	WI38	1.73	n.d.	1,2
**2**	135	MCF-7	720	WI38	5.33	Inhibition of telomerase activity	1,2,6
**2**	114	A549	720	WI38	6.32		1,2,6
**2**	380	HeLa	720	WI38	1.89		6
**2**	8100	MDA-MB32	720	WI38	0.0900		6
**3**	n.d.	n.d.	n.d.	n.d.	n.d.	n.d.	-
**4**	n.d.	n.d.	n.d.	n.d.	n.d.	n.d.	-
**5**	2130	HCT116	n.d.	n.d.	n.d.	n.d.	7
**6**	n.d.	n.d.	n.d.	n.d.	n.d.	n.d.	-
**7**	3080	HCT116	n.d.	n.d.	n.d.	n.d.	7
**8**	n.d.	n.d.	n.d.	n.d.	n.d.	n.d.	-
**9**	n.d.	n.d.	n.d.	n.d.	n.d.	n.d.	-
**10**	n.d.	n.d.	n.d.	n.d.	n.d.	n.d.	-
**11**	n.d.	n.d.	n.d.	n.d.	n.d.	n.d.	-
**12**	n.d.	n.d.	n.d.	n.d.	n.d.	n.d.	-
**13**	n.d.	n.d.	n.d.	n.d.	n.d.	n.d.	-
**14**	n.d.	n.d.	n.d.	n.d.	n.d.	n.d.	-
**15**	n.d.	n.d.	n.d.	n.d.	n.d.	n.d.	-
**16**	n.d.	n.d.	n.d.	n.d.	n.d.	n.d.	-
**17**	152	OVCAR-4	n.d.	n.d.	n.d.	n.d.	10
**17**	2654	HOP-92	n.d.	n.d.	n.d.	n.d.	10
**18**	167	SR	n.d.	n.d.	n.d.	n.d.	10
**18**	2900	ADR-RES	n.d.	n.d.	n.d.	n.d.	10
**19**	28	SR	n.d.	n.d.	n.d.	n.d.	10
**19**	1610	TK-10	n.d.	n.d.	n.d.	n.d.	10
**20**	76	SR	n.d.	n.d.	n.d.	n.d.	10
**20**	1860	UO-31	n.d.	n.d.	n.d.	n.d.	10
**21**	38	SR	n.d.	n.d.	n.d.	n.d.	10
**21**	13150	CAKI-I	n.d.	n.d.	n.d.	n.d.	10
**22**	270	SR	n.d.	n.d.	n.d.	n.d.	10
**22**	5880	UO-31	n.d.	n.d.	n.d.	n.d.	10
**23**	173	MOLT-4	n.d.	n.d.	n.d.	n.d.	10
**23**	21700	CAKI-I	n.d.	n.d.	n.d.	n.d.	10
**24**	n.d.	n.d.	n.d.	n.d.	n.d.	n.d.	-
**25**	4390	HCT116	n.d.	n.d.	n.d.	n.d.	7
**25**	133	MDA-MB-468	n.d.	n.d.	n.d.	n.d.	10
**25**	2675	UO-31	n.d.	n.d.	n.d.	n.d.	10
**26**	342	T47-D	n.d.	n.d.	n.d.	n.d.	10
**26**	5620	H522	n.d.	n.d.	n.d.	n.d.	10
**27**	197	OVCAR-4	n.d.	n.d.	n.d.	n.d.	10
**27**	2660	HOP-62	n.d.	n.d.	n.d.	n.d.	10
**28**	n.d.	n.d.	n.d.	n.d.	n.d.	n.d.	-
**29**	200	A549	400	WI38	2.00	n.d.	11
**29**	300	MCF-7	400	WI38	1.33	n.d.	11
**29**	200	MIA-Pa-Ca-2	400	WI38	2.00	n.d.	11
**29**	400	PANC-1	400	WI38	1.00	n.d.	11

**29**	200	ALT	400	WI38	2.00	n.d.	[11]
**30**	410	HeLa	2280	WI38	5.56	n.d.	[6]
**30**	3090	MCF-7	2280	WI38	0.740	n.d.	[6]
**30**	810	MDA-MB32	2280	WI38	2.81	n.d.	[6]
**31**	6230	HeLa	21460	WI38	3.44	n.d.	[6]
**31**	52760	MCF-7	21460	WI38	0.410	n.d.	[6]
**32**	3630	HeLa	40220	WI38	11.1	n.d.	[6]
**32**	11770	MCF-7	40220	WI38	3.42	n.d.	[6]
**33**	n.d.	n.d.	n.d.	n.d.	n.d.	n.d.	**-**
**34**	n.d.	n.d.	n.d.	n.d.	n.d.	n.d.	**-**
**35**	79500	A549	>100000	HEK293T	>1.26	n.d.	[12]
**35**	92700	HeLa	>100000	HEK293T	>1.08	n.d.	[12]
**36**	26400	A549	77900	HEK293T	2.95	n.d.	[12]
**36**	24700	HeLa	77900	HEK293T	3.15	n.d.	[12]
**37**	>100000	HeLa	>100000	WI38	~1.00	n.d.	[6]
**37**	>100000	MCF-7	>100000	WI38	~1.00	n.d.	[6]
**37**	>100000	MDA-MB32	>100000	WI38	~1.00	n.d.	[6]
**38**	>100000	HeLa	>100000	WI38	~1.00	n.d.	[6]
**38**	>100000	MCF-7	>100000	WI38	~1.00	n.d.	[6]
**38**	>100000	MDA-MB32	>100000	WI38	~1.00	n.d.	[6]
**39**	>100000	HeLa	>100000	WI38	~1.00	n.d.	[6]
**39**	>100000	MCF-7	>100000	WI38	~1.00	n.d.	[6]
**39**	>100000	MDA-MB32	>100000	WI38	~1.00	n.d.	[6]
**40**	18	MCF-7	138	WI38	7.67	n.d.	[1,2]
**40**	25	A549	138	WI38	5.52	n.d.	[1,2]
**41**	164	MCF-7	467	WI38	2.85	n.d.	[1,2]
**41**	144	A549	467	WI38	3.24	n.d.	[1,2]
**41**	35	HT-29D	467	WI38	13.3	n.d.	[1,2]
**41**	68	HGC-27	467	WI38	6.87	n.d.	[1,2]
**42**	n.d.	n.d.	n.d.	n.d.	n.d.	n.d.	**-**
**43**	120	HT-29	360	MRC-5	3.00	n.d.	[15,16]
**43**	130	MCF-7	360	MRC-5	2.77	n.d.	[15,16]
**43**	290	HeLa	360	MRC-5	1.24	n.d.	[15,16]
**44**	5	MCF-7	43	WI38	8.60	Inhibition of telomerase activity	[1,2]
**44**	13	A549	43	WI38	3.31		[1,2]
**44**	590	PNT1A	43	WI38	0.0700		[1,2,18]
**44**	3880	DU145	43	WI38	0.0100		[1,2,18]
**44**	6320	PC-3	43	WI38	0.0100		[1,2,18]
**44**	3400	LNCaP	43	WI38	0.0100		[1,2,18]
**44**	2430	22Rv1	43	WI38	0.0200		[1,2,18]
**44**	50	HCT116	43	WI38	0.860		[1,2,19]
**44**	100	HT-29	43	WI38	0.430		[1,2,19]
**44**	20	U2OS	43	WI38	2.15		[1,2,19]
**44**	<10	MDA-MB321	43	WI38	>4.30		[1,2,19]
**45**	64	HeLa	n.d.	n.d.	n.d.	DNA damage response	[17]
**46**	79	HeLa	n.d.	n.d.	n.d.	DNA damage response	[17]
**47**	52	HeLa	n.d.	n.d.	n.d.	DNA damage response	[17]
**48**	18	MCF-7	84	WI38	4.67	Inhibition of telomerase activity, senescence	[1,2]
**48**	48	A549	84	WI38	1.75		[1,2]
**49**	n.d.	n.d.	n.d.	n.d.	n.d.	n.d.	**-**
**50**	9200	MCF-7	>10000	WI38	>1.09	n.d.	[1,2]
**50**	>10000	A549	>10000	WI38	~1.00	n.d.	[1,2]
**51**	1250	HT-29	1020	AG01522	0.820	Inhibition of *hTERT* and *bcl-2* oncogenes expression	[22,23]
**51**	1100	A549	1020	AG01522	0.930		[22,23]
**51**	1900	SKMel-5	1020	AG01522	0.540		[22,23]
**51**	180	U251MG	1020	AG01522	5.67		[22]
**52**	1000	HT-29	430	AG01522	0.430		[22,23]
**52**	1200	A549	430	AG01522	0.360		[22,23]

**52**	1750	SKMel-5	430	AG01522	0.250	Inhibition of *hTERT* and *bcl-2* oncogenes expression, inhibition of MYC, KIT, RET, TRF2 and hPOT1 proteins expression, inhibition of telomerase activity, senescence	[22,24]
**52**	1180	H69	430	AG01522	0.360		[22,24]
**52**	75	U251MG	430	AG01522	5.73		[22]
**52**	350	PNT1A	430	AG01522	1.23		[18,22]
**52**	300	DU145	430	AG01522	1.43		[18,22]
**52**	320	PC-3	430	AG01522	1.34		[18,22]
**52**	390	LNCaP	430	AG01522	1.10		[18,22]
**52**	100	22Rv1	430	AG01522	4.30		[18,22]
**53**	400	HT-29	n.d.	n.d.	n.d.	n.d.	[23]
**53**	900	A549	n.d.	n.d.	n.d.	n.d.	[23]
**54**	1200	HT-29	n.d.	n.d.	n.d.	n.d.	[23]
**54**	3000	A549	n.d.	n.d.	n.d.	n.d.	[23]
**54**	2800	SKMel-5	n.d.	n.d.	n.d.	n.d.	[23]
**55**	1400	HT-29	18000	HFF	12.9	n.d.	[26]
**55**	4500	A549	18000	HFF	4.00	n.d.	[26]
**56**	n.d.	n.d.	n.d.	n.d.	n.d.	n.d.	-
**57**	4500	HT-29	18000	HFF	4.00	n.d.	[26]
**57**	5500	A549	18000	HFF	3.27	n.d.	[26]
**57**	1440	PNT1A	18000	HFF	12.5	n.d.	[18,26]
**57**	>10000	DU145	18000	HFF	<1.80	n.d.	[18,26]
**57**	>>10000	PC-3	18000	HFF	<<1.80	n.d.	[18,26]
**57**	3040	LNCaP	18000	HFF	5.92	n.d.	[18,26]
**57**	3690	22Rv1	18000	HFF	4.88	n.d.	[18,26]
**58**	n.d.	n.d.	n.d.	n.d.	n.d.	n.d.	-
**59**	n.d.	n.d.	n.d.	n.d.	n.d.	n.d.	-
**60**	n.d.	n.d.	n.d.	n.d.	n.d.	n.d.	-
**61**	n.d.	n.d.	n.d.	n.d.	n.d.	n.d.	-
**62**	n.d.	n.d.	n.d.	n.d.	n.d.	n.d.	-
**63**	n.d.	n.d.	n.d.	n.d.	n.d.	n.d.	-
**64**	130	PNT1A	n.d.	n.d.	n.d.	n.d.	[18]
**64**	550	DU145	n.d.	n.d.	n.d.	n.d.	[18]
**64**	340	PC-3	n.d.	n.d.	n.d.	n.d.	[18]
**64**	590	LNCaP	n.d.	n.d.	n.d.	n.d.	[18]
**64**	970	22Rv1	n.d.	n.d.	n.d.	n.d.	[18]
**65**	n.d.	n.d.	n.d.	n.d.	n.d.	n.d.	-
**66**	n.d.	n.d.	n.d.	n.d.	n.d.	n.d.	-
**67**	>100000	HT-29	n.d.	n.d.	n.d.	n.d.	[23]
**67**	>100000	A549	n.d.	n.d.	n.d.	n.d.	[23]
**67**	>100000	SKMel-5	n.d.	n.d.	n.d.	n.d.	[23]
**68**	590	K562	n.d.	n.d.	n.d.	n.d.	[28]
**68**	2030	A549	n.d.	n.d.	n.d.	n.d.	[28]
**68**	2150	HeLa	n.d.	n.d.	n.d.	n.d.	[28]
**69**	610	K562	n.d.	n.d.	n.d.	n.d.	[28]
**69**	2190	A549	n.d.	n.d.	n.d.	n.d.	[28]
**69**	2080	HeLa	n.d.	n.d.	n.d.	n.d.	[28]
**70**	3030	K562	n.d.	n.d.	n.d.	n.d.	[28]
**70**	2940	A549	n.d.	n.d.	n.d.	n.d.	[28]
**70**	5190	HeLa	n.d.	n.d.	n.d.	n.d.	[28]
**71**	670	K562	n.d.	n.d.	n.d.	n.d.	[28]
**71**	2950	A549	n.d.	n.d.	n.d.	n.d.	[28]
**71**	2610	HeLa	n.d.	n.d.	n.d.	n.d.	[28]
**72**	2520	K562	n.d.	n.d.	n.d.	n.d.	[28]
**72**	2910	A549	n.d.	n.d.	n.d.	n.d.	[28]
**72**	5000	HeLa	n.d.	n.d.	n.d.	n.d.	[28]
**73**	1240	K562	n.d.	n.d.	n.d.	n.d.	[28]
**73**	2930	A549	n.d.	n.d.	n.d.	n.d.	[28]
**73**	5950	HeLa	n.d.	n.d.	n.d.	n.d.	[28]
**74**	2780	K562	n.d.	n.d.	n.d.	n.d.	[28]
**74**	6870	A549	n.d.	n.d.	n.d.	n.d.	[28]
**74**	9800	HeLa	n.d.	n.d.	n.d.	n.d.	[28]
**75**	1480	K562	n.d.	n.d.	n.d.	n.d.	[28]
**75**	2830	A549	n.d.	n.d.	n.d.	n.d.	[28]
**75**	5930	HeLa	n.d.	n.d.	n.d.	n.d.	[28]

**76**	7100	K562	n.d.	n.d.	n.d.	n.d.	28
**76**	7120	A549	n.d.	n.d.	n.d.	n.d.	28
**76**	5170	HeLa	n.d.	n.d.	n.d.	n.d.	28
**77**	2940	K562	n.d.	n.d.	n.d.	n.d.	28
**77**	6870	A549	n.d.	n.d.	n.d.	n.d.	28
**77**	4060	HeLa	n.d.	n.d.	n.d.	n.d.	28
**78**	3630	K562	n.d.	n.d.	n.d.	n.d.	28
**78**	7420	A549	n.d.	n.d.	n.d.	n.d.	28
**78**	2880	HeLa	n.d.	n.d.	n.d.	n.d.	28
**79**	5790	K562	n.d.	n.d.	n.d.	n.d.	28
**79**	2530	A549	n.d.	n.d.	n.d.	n.d.	28
**79**	3800	HeLa	n.d.	n.d.	n.d.	n.d.	28
**80**	2920	HT-29	1150	MRC-5	0.390	n.d.	15,16
**80**	1190	MCF-7	1150	MRC-5	0.970	n.d.	15,16
**80**	1560	HeLa	1150	MRC-5	0.740	n.d.	15,16
**81**	2260	HT-29	510	MRC-5	0.230	n.d.	15,16
**81**	1060	MCF-7	510	MRC-5	0.480	n.d.	15,16
**81**	950	HeLa	510	MRC-5	0.540	n.d.	15,16
**82**	400	HT-29	910	MRC-5	2.28	n.d.	15,16
**82**	690	MCF-7	910	MRC-5	1.32	n.d.	15,16
**82**	350	HeLa	910	MRC-5	2.60	n.d.	15,16
**83**	1850	HT-29	2040	MRC-5	1.10	n.d.	15,16
**83**	1420	MCF-7	2040	MRC-5	1.44	n.d.	15,16
**83**	540	HeLa	2040	MRC-5	3.78	n.d.	15,16
**84**	420	HT-29	810	MRC-5	1.93	n.d.	15,16
**84**	150	MCF-7	810	MRC-5	5.40	n.d.	15,16
**84**	290	HeLa	810	MRC-5	2.79	n.d.	15,16
**85**	360	HT-29	710	MRC-5	1.97	n.d.	15,16
**85**	240	MCF-7	710	MRC-5	2.96	n.d.	15,16
**85**	240	HeLa	710	MRC-5	2.96	n.d.	15,16
**86**	11	MCF-7	64	WI38	5.82	n.d.	2
**86**	18	A549	64	WI38	3.56	n.d.	2
**87**	11	MCF-7	64	WI38	5.82	n.d.	1
**87**	18	A549	64	WI38	3.56	n.d.	1
**88**	129	MCF-7	439	WI38	3.40	n.d.	1,2
**88**	146	A549	439	WI38	3.01	n.d.	1,2
**89**	1610	MCF-7	8500	WI38	5.28	n.d.	1,2
**89**	2750	A549	8500	WI38	3.09	n.d.	1,2
**90**	10	MCF-7	63	WI38	6.30	n.d.	1,2
**90**	96	A549	63	WI38	0.660	n.d.	1,2
**91**	800	MCF-7	1220	WI38	1.53	n.d.	1,2
**91**	1320	A549	1220	WI38	0.920	n.d.	1,2
**92**	825	A549	5530	WI38	6.70	n.d.	29
**92**	3330	MCF-7	5530	WI38	1.66	n.d.	29
**92**	1085	MIA-Pa-Ca-2	5530	WI38	5.10	n.d.	29
**92**	909	PANC-1	5530	WI38	6.08	n.d.	29
**92**	2670	ALT	5530	WI38	2.07	n.d.	29
**93**	24	A549	1190	WI38	49.6	DNA damage response, up-regulation of Suppressor of Tumorigenicity gene, down-regulation of genes involved in pancreatic cancer	29,30
**93**	159	MCF-7	1190	WI38	7.48		29,30
**93**	7	MIA-Pa-Ca-2	1190	WI38	170		29,30
**93**	18	PANC-1	1190	WI38	66.1		29,30
**93**	11	GemMIA-R3	1190	WI38	108		29,30,32
**93**	26	Capan-1	1190	WI38	45.8		29,30,47
**93**	16	BxPC-3	1190	WI38	74.4		29,30,47
**93**	93	ALT	1190	WI38	12.8		29,30
**94**	146	A549	1880	WI38	12.9	n.d.	29
**94**	1030	MCF-7	1880	WI38	1.83	n.d.	29
**94**	139	MIA-Pa-Ca-2	1880	WI38	13.5	n.d.	29
**94**	808	PANC-1	1880	WI38	2.33	n.d.	29

**94**	710	ALT	1880	WI38	2.65	n.d.	[29]
**95**	198	A549	5330	WI38	26.9	n.d.	[29]
**95**	1110	MCF-7	5330	WI38	4.80	n.d.	[29]
**95**	108	MIA-Pa-Ca-2	5330	WI38	49.4	n.d.	[29]
**95**	84	PANC-1	5330	WI38	63.4	n.d.	[29]
**95**	535	ALT	5330	WI38	9.96	n.d.	[29]
**96**	86	A549	1490	WI38	17.3	n.d.	[29]
**96**	316	MCF-7	1490	WI38	4.72	n.d.	[29]
**96**	48	MIA-Pa-Ca-2	1490	WI38	31.0	n.d.	[29]
**96**	46	PANC-1	1490	WI38	32.4	n.d.	[29]
**96**	85	ALT	1490	WI38	17.5	n.d.	[29]
**97**	2180	A549	17650	WI38	8.10	n.d.	[29]
**97**	>25000	MCF-7	17650	WI38	<0.710	n.d.	[29]
**97**	206	MIA-Pa-Ca-2	17650	WI38	85.7	n.d.	[29]
**97**	220	PANC-1	17650	WI38	80.2	n.d.	[29]
**97**	10874	ALT	17650	WI38	1.62	n.d.	[29]
**98**	92	A549	1650	WI38	17.9	n.d.	[29]
**98**	1538	MCF-7	1650	WI38	1.07	n.d.	[29]
**98**	59	MIA-Pa-Ca-2	1650	WI38	28.0	n.d.	[29]
**98**	163	PANC-1	1650	WI38	10.1	n.d.	[29]
**98**	451	ALT	1650	WI38	3.66	n.d.	[29]
**99**	130	A549	2240	WI38	17.2	n.d.	[29]
**99**	1070	MCF-7	2240	WI38	2.09	n.d.	[29]
**99**	220	MIA-Pa-Ca-2	2240	WI38	10.2	n.d.	[29]
**99**	340	PANC-1	2240	WI38	6.59	n.d.	[29]
**99**	1290	ALT	2240	WI38	1.74	n.d.	[29]
**100**	67	A549	1830	WI38	27.3	n.d.	[29]
**100**	357	MCF-7	1830	WI38	5.13	n.d.	[29]
**100**	59	MIA-Pa-Ca-2	1830	WI38	31.0	n.d.	[29]
**100**	45	PANC-1	1830	WI38	40.7	n.d.	[29]
**100**	224	ALT	1830	WI38	8.17	n.d.	[29]
**101**	26	A549	1220	WI38	46.9	n.d.	[29]
**101**	222	MCF-7	1220	WI38	5.50	n.d.	[29]
**101**	36	MIA-Pa-Ca-2	1220	WI38	33.9	n.d.	[29]
**101**	33	PANC-1	1220	WI38	37.0	n.d.	[29]
**101**	89	ALT	1220	WI38	13.7	n.d.	[29]
**102**	9	MCF-7	40	WI38	4.44	n.d.	[1,2]
**102**	15	A549	40	WI38	2.67	n.d.	[1,2]
**103**	219	MCF-7	972	WI38	4.44	n.d.	[1,2]
**103**	274	A549	972	WI38	3.55	n.d.	[1,2]
**103**	81	HT-29D	972	WI38	12.0	n.d.	[1,2]
**103**	480	HGC-27	972	WI38	2.03	n.d.	[1,2]
**103**	530	GIST882	972	WI38	1.83	n.d.	[1,2]
**104**	n.d.	n.d.	n.d.	n.d.	n.d.	n.d.	**-**
**105**	n.d.	n.d.	n.d.	n.d.	n.d.	n.d.	**-**
**106**	105	MCF-7	292	WI38	2.78	Inhibition of telomerase activity, senescence	[1,2,19]
**106**	29	A549	292	WI38	10.1		[1,2,19]
**106**	42	HCT116	292	WI38	6.95		[1,2,19]
**106**	200	HT-29	292	WI38	1.46		[1,2,19]
**106**	36	U2OS	292	WI38	8.11		[1,2,19]
**106**	160	MDA-MB321	292	WI38	1.83		[1,2,19]
**106**	63	HT-29D	292	WI38	4.63		[1,2,19]
**106**	510	HGC-27	292	WI38	0.570		[1,2,19]
**106**	1300	GIST882	292	WI38	0.220		[1,2,19]
**107**	18	MCF-7	87	WI38	4.83	Inhibition of telomerase activity	[1,2]

**107**	11	A549	87	WI38	7.91		[1,2]
**107**	20	HT-29D	87	WI38	4.35		[1,2]
**107**	170	HGC-27	87	WI38	0.510		[1,2]
**108**	n.d.	n.d.	n.d.	n.d.	n.d.	n.d.	-
**109**	288	MCF-7	10000	WI38	34.7	n.d.	[1,2]
**109**	1700	A549	10000	WI38	5.88	n.d.	[1,2]
**110**	8300	MCF-7	>10000	WI38	>1.20	n.d.	[1,2]
**110**	>10000	A549	>10000	WI38	~1.00	n.d.	[1,2]
**111**	n.d.	n.d.	n.d.	n.d.	n.d.	n.d.	-
**112**	n.d.	n.d.	n.d.	n.d.	n.d.	n.d.	-
**113**	33	U251MG	820	AG01522	24.8	Inhibition of *hTERT* and *bcl-2* oncogenes expression	[22,23]
**113**	2500	HT-29	820	AG01522	0.330		[22,23]
**113**	7600	A549	820	AG01522	0.110		[22,23]
**113**	3900	SKMel-5	820	AG01522	0.210		[22,23]
**114**	n.d.	n.d.	n.d.	n.d.	n.d.	n.d.	-
**115**	n.d.	n.d.	n.d.	n.d.	n.d.	n.d.	-
**116**	n.d.	n.d.	n.d.	n.d.	n.d.	n.d.	-
**117**	n.d.	n.d.	n.d.	n.d.	n.d.	n.d.	-
**118**	n.d.	n.d.	n.d.	n.d.	n.d.	n.d.	-
**119**	n.d.	n.d.	n.d.	n.d.	n.d.	n.d.	-
**120**	4000	HT-29	n.d.	n.d.	n.d.	n.d.	[23]
**120**	3200	A549	n.d.	n.d.	n.d.	n.d.	[23]
**120**	5100	SKMel-5	n.d.	n.d.	n.d.	n.d.	[23]
**121**	1000	HT-29	n.d.	n.d.	n.d.	n.d.	[23]
**121**	1300	A549	n.d.	n.d.	n.d.	n.d.	[23]
**122**	4000	HT-29	n.d.	n.d.	n.d.	n.d.	[23]
**122**	9000	A549	n.d.	n.d.	n.d.	n.d.	[23]
**123**	9300	HT-29	40000	HFF	4.30	n.d.	[26]
**123**	27500	A549	40000	HFF	1.45	n.d.	[26]
**124**	n.d.	n.d.	n.d.	n.d.	n.d.	n.d.	-
**125**	4200	HT-29	40000	HFF	9.52	n.d.	[26]
**125**	20600	A549	40000	HFF	1.94	n.d.	[26]
**126**	n.d.	n.d.	n.d.	n.d.	n.d.	n.d.	-
**127**	n.d.	n.d.	n.d.	n.d.	n.d.	n.d.	-
**128**	>100000	HT-29	n.d.	n.d.	n.d.	n.d.	[23]
**128**	>100000	A549	n.d.	n.d.	n.d.	n.d.	[23]
**128**	>100000	SKMel-5	n.d.	n.d.	n.d.	n.d.	[23]
**129**	n.d.	n.d.	n.d.	n.d.	n.d.	n.d.	-
**130**	n.d.	n.d.	n.d.	n.d.	n.d.	n.d.	-
**131**	n.d.	n.d.	n.d.	n.d.	n.d.	n.d.	-
**132**	n.d.	n.d.	n.d.	n.d.	n.d.	n.d.	-
**133**	n.d.	n.d.	n.d.	n.d.	n.d.	n.d.	-
**134**	n.d.	n.d.	n.d.	n.d.	n.d.	n.d.	-
**135**	n.d.	n.d.	n.d.	n.d.	n.d.	n.d.	-
**136**	n.d.	n.d.	n.d.	n.d.	n.d.	n.d.	-
**137**	27	MCF-7	58	WI38	2.15	n.d.	[1,2]
**137**	57	A549	58	WI38	1.02	n.d.	[1,2]
**138**	7070	PNT1A	n.d.	n.d.	n.d.	n.d.	[18]
**138**	>>10000	DU145	n.d.	n.d.	n.d.	n.d.	[18]
**138**	>10000	PC-3	n.d.	n.d.	n.d.	n.d.	[18]
**138**	>>10000	LNCaP	n.d.	n.d.	n.d.	n.d.	[18]
**138**	7670	22Rv1	n.d.	n.d.	n.d.	n.d.	[18]
**139**	10510	RCC4	3320	WI38	0.320	n.d.	[37–39]
**139**	7170	786-O	3320	WI38	0.460	n.d.	[37–39]
**139**	5620	MCF-7	3320	WI38	0.590	n.d.	[37–39]
**139**	2790	MIA-Pa-Ca-2	3320	WI38	1.19	n.d.	[37–39]
**139**	2540	A549	3320	WI38	1.31	n.d.	[37–39]
**140**	7600	RCC4	>25000	WI38	>3.29	n.d.	[37]
**140**	16100	786-O	>25000	WI38	>1.55	n.d.	[37]
**140**	17500	MCF-7	>25000	WI38	>1.43	n.d.	[37]
**140**	7050	MIA-Pa-Ca-2	>25000	WI38	>3.55	n.d.	[37]

**140**	5750	A549	>25000	WI38	>4.35	n.d.	[37]
**140**	2500	PANC-1	>25000	WI38	>10.0	n.d.	[37]
**141**	10	MCF-7	63	WI38	6.30	n.d.	[2]
**141**	14	A549	63	WI38	4.50	n.d.	[2]
**141**	15	HT-29D	63	WI38	4.20	n.d.	[2]
**141**	41	HGC-27	63	WI38	1.54	n.d.	[2]
**141**	1600	GIST882	63	WI38	0.0400	n.d.	[2]
**142**	>10000	RCC4	>10000	WI38	~1.00	n.d.	[37–39]
**142**	>10000	786-O	>10000	WI38	~1.00	n.d.	[37–39]
**142**	>10000	MCF-7	>10000	WI38	~1.00	n.d.	[37–39]
**142**	5650	MIA-Pa-Ca-2	>10000	WI38	>1.77	n.d.	[37–39]
**142**	5570	A549	>10000	WI38	>1.80	n.d.	[37–39]
**143**	2410	A549	6840	WI38	2.84	n.d.	[38,39]
**143**	3110	RCC4	6840	WI38	2.20	n.d.	[38,39]
**143**	2830	MIA-Pa-Ca-2	6840	WI38	2.42	n.d.	[38,39]
**143**	1100	786-O	6840	WI38	6.22	n.d.	[38,39]
**143**	2610	MCF-7	6840	WI38	2.62	n.d.	[38,39]
**144**	8380	RCC4	12650	WI38	1.51	n.d.	[37–39]
**144**	1200	786-O	12650	WI38	10.5	n.d.	[37–39]
**144**	3120	MCF-7	12650	WI38	4.05	n.d.	[37–39]
**144**	2500	MIA-Pa-Ca-2	12650	WI38	5.06	n.d.	[37–39]
**144**	2920	A549	12650	WI38	4.33	n.d.	[37–39]
**145**	1600	GIST882	63	WI38	0.0400	Down-regulation of KIT and BCL-2 proteins expression, inhibition of telomerase activity	[1,41]
**145**	500	GIST48	63	WI38	0.130	Down-regulation of KIT and BCL-2 proteins expression, inhibition of telomerase activity	[1,41]
**145**	400	GIST62	63	WI38	0.160	Down-regulation of KIT and BCL-2 proteins expression, inhibition of telomerase activity	[1,41]
**145**	40	HT-29	63	WI38	1.58	Down-regulation of KIT and BCL-2 proteins expression, inhibition of telomerase activity	[1,41]
**145**	100	HGC-27	63	WI38	0.630	Down-regulation of KIT and BCL-2 proteins expression, inhibition of telomerase activity	[1,41]
**145**	10	MCF-7	63	WI38	6.30	Down-regulation of KIT and BCL-2 proteins expression, inhibition of telomerase activity	[1,41]
**145**	14	A549	63	WI38	4.50	Down-regulation of KIT and BCL-2 proteins expression, inhibition of telomerase activity	[1,41]
**146**	296	MCF-7	1460	WI38	4.93	n.d.	[1,2]
**146**	204	A549	1460	WI38	7.16	n.d.	[1,2]
**147**	1010	MCF-7	5590	WI38	5.53	n.d.	[42]
**147**	470	A549	5590	WI38	11.9	n.d.	[42]
**148**	6700	MCF-7	>10000	WI38	>1.49	Inhibition of telomerase activity, senescence	[1,2]
**148**	>10000	A549	>10000	WI38	~1.00	Inhibition of telomerase activity, senescence	[1,2]
**149**	4930	A549	1170	WI38	0.240	n.d.	[38,39]
**149**	5100	RCC4	1170	WI38	0.230	n.d.	[38,39]
**149**	1480	786-O	1170	WI38	0.790	n.d.	[38,39]
**149**	180	MCF-7	1170	WI38	6.50	n.d.	[38,39]
**150**	1750	RCC4	610	WI38	0.350	n.d.	[37–39]
**150**	630	786-O	610	WI38	0.970	n.d.	[37–39]
**150**	170	MCF-7	610	WI38	3.59	n.d.	[37–39]
**150**	40	MIA-Pa-Ca-2	610	WI38	15.2	n.d.	[37–39]
**150**	1550	A549	610	WI38	0.390	n.d.	[37–39]
**151**	300	RCC4	180	WI38	0.600	n.d.	[37]
**151**	160	786-O	180	WI38	1.13	n.d.	[37]
**151**	21	MCF-7	180	WI38	8.57	n.d.	[37]
**151**	5	MIA-Pa-Ca-2	180	WI38	36.0	n.d.	[37]
**151**	20	A549	180	WI38	9.00	n.d.	[37]
**151**	5	PANC-1	180	WI38	36.0	n.d.	[37]
**152**	81	MCF-7	167	WI38	2.06	Chromosomal instability, telomeric aggregation, inhibition of telomerase activity, senescence	[1,2]
**152**	115	A549	167	WI38	1.45	Chromosomal instability, telomeric aggregation, inhibition of telomerase activity, senescence	[1,2]
**153**	167	MCF-7	9042	WI38	54.1	n.d.	[43]
**153**	108	A549	9042	WI38	83.7	n.d.	[43]
**153**	111	MIA-Pa-Ca-2	9042	WI38	81.5	n.d.	[43]
**153**	148	PANC-1	9042	WI38	61.1	n.d.	[43]
**153**	186	HPAC	9042	WI38	48.6	n.d.	[43]

**153**	1520	BxPc-3	9042	WI38	5.95	n.d.	[43]
**154**	437	MCF-7	965	WI38	2.21	n.d.	[1,2]
**154**	1190	A549	965	WI38	0.810	n.d.	[1,2]
**155**	3000	RCC4	2300	WI38	0.770	n.d.	[37]
**155**	390	786-O	2300	WI38	5.90	n.d.	[37]
**155**	560	MCF-7	2300	WI38	4.11	n.d.	[37]
**155**	95	MIA-Pa-Ca-2	2300	WI38	24.2	n.d.	[37]
**155**	160	A549	2300	WI38	14.4	n.d.	[37]
**155**	1500	PANC-1	2300	WI38	1.53	n.d.	[37]
**156**	2200	RCC4	2800	WI38	1.27	n.d.	[37]
**156**	290	786-O	2800	WI38	9.66	n.d.	[37]
**156**	510	MCF-7	2800	WI38	5.49	n.d.	[37]
**156**	137	MIA-Pa-Ca-2	2800	WI38	20.4	n.d.	[37]
**156**	460	A549	2800	WI38	6.09	n.d.	[37]
**156**	1100	PANC-1	2800	WI38	2.55	n.d.	[37]
**157**	280	RCC4	2460	WI38	8.79	n.d.	[37–39]
**157**	30	MCF-7	2460	WI38	82.0	n.d.	[37–39]
**157**	10	MIA-Pa-Ca-2	2460	WI38	246	n.d.	[37–39]
**157**	<10	A549	2460	WI38	>246	n.d.	[37–39]
**158**	610	RCC4	300	WI38	0.490	n.d.	[37]
**158**	440	786-O	300	WI38	0.680	n.d.	[37]
**158**	14	MCF-7	300	WI38	21.4	n.d.	[37]
**158**	50	MIA-Pa-Ca-2	300	WI38	6.00	n.d.	[37]
**158**	7	A549	300	WI38	42.9	n.d.	[37]
**158**	2	PANC-1	300	WI38	150	n.d.	[37]
**159**	560	RCC4	230	WI38	0.410	Up-regulation of DNA damage responsive genes, down-regulation of telomere maintenance genes	[29,30,37–39]
**159**	320	786-O	230	WI38	0.720		[29,30,37–39]
**159**	70	MCF-7	230	WI38	3.29		[29,30,37–39]
**159**	10	MIA-Pa-Ca-2	230	WI38	23.0		[29,30,37–39]
**159**	19	A549	230	WI38	12.1		[29,30,37–39]
**159**	3	PANC-1	230	WI38	76.7		[29,30,37–39]
**159**	63	ALT	230	WI38	3.65		[29,30,37–39]
**160**	100	MCF-7	5500	WI38	55.0	n.d.	[43]
**160**	69	A549	5500	WI38	79.7	n.d.	[43]
**160**	73	MIA-Pa-Ca-2	5500	WI38	75.3	n.d.	[43]
**160**	106	PANC-1	5500	WI38	51.9	n.d.	[43]
**160**	117	HPAC	5500	WI38	47.0	n.d.	[43]
**160**	1020	BxPc-3	5500	WI38	5.39	n.d.	[43]
**161**	196	MCF-7	8430	WI38	43.0	DNA damage response, telomere uncapping, senescence	[43]
**161**	258	A549	8430	WI38	32.7		[43]
**161**	121	MIA-Pa-Ca-2	8430	WI38	69.7		[43]
**161**	193	PANC-1	8430	WI38	43.7		[43]
**161**	205	HPAC	8430	WI38	41.1		[43]
**161**	759	BxPc-3	8430	WI38	11.1		[43]
**162**	1.3	MIA-Pa-Ca-2	n.d.	n.d.	n.d.	Down-regulation of several cancer gene pathways	[47]
**162**	1.4	PANC-1	n.d.	n.d.	n.d.		[47]
**162**	5.9	Capan-1	n.d.	n.d.	n.d.		[47]
**162**	2.6	BxPc-3	n.d.	n.d.	n.d.		[47]
**163**	300	HT-29	n.d.	n.d.	n.d.	n.d.	[48]

**163**	1500	A549	n.d.	n.d.	n.d.	n.d.	[48]
**164**	1370	PNT1A	n.d.	n.d.	n.d.	n.d.	[18]
**164**	>>10000	DU145	n.d.	n.d.	n.d.	n.d.	[18]
**164**	>>10000	PC-3	n.d.	n.d.	n.d.	n.d.	[18]
**164**	10330	LNCaP	n.d.	n.d.	n.d.	n.d.	[18]
**164**	>>10000	22Rv1	n.d.	n.d.	n.d.	n.d.	[18]
**165**	280	PNT1A	n.d.	n.d.	n.d.	Down-regulation of AR, KIT and BCL-2 proteins expression	[18]
**165**	80	DU145	n.d.	n.d.	n.d.		[18]
**165**	180	PC-3	n.d.	n.d.	n.d.		[18]
**165**	140	LNCaP	n.d.	n.d.	n.d.		[18]
**165**	10	22Rv1	n.d.	n.d.	n.d.		[18]
**165**	8	A375	n.d.	n.d.	n.d.		[49]
**166**	50	A375	n.d.	n.d.	n.d.	n.d.	[49]
**167**	>500	A375	n.d.	n.d.	n.d.	n.d.	[49]
**168**	20	A375	n.d.	n.d.	n.d.	n.d.	[49]
**169**	74	A375	n.d.	n.d.	n.d.	n.d.	[49]
**170**	>500	A375	n.d.	n.d.	n.d.	n.d.	[49]
**171**	104	A375	n.d.	n.d.	n.d.	n.d.	[49]
**172**	54	A375	n.d.	n.d.	n.d.	n.d.	[49]
**173**	415	A375	n.d.	n.d.	n.d.	n.d.	[49]
**174**	195	A375	n.d.	n.d.	n.d.	n.d.	[49]
**175**	94	A375	n.d.	n.d.	n.d.	n.d.	[49]
**176**	101	A375	n.d.	n.d.	n.d.	n.d.	[49]
**177**	>500	A375	n.d.	n.d.	n.d.	n.d.	[49]
**178**	>500	A375	n.d.	n.d.	n.d.	n.d.	[49]
**179**	n.d.	n.d.	n.d.	n.d.	n.d.	n.d.	**-**
**180**	300	HeLa	n.d.	n.d.	n.d.	n.d.	[17]
**181**	5800	HeLa	n.d.	n.d.	n.d.	n.d.	[17]
**182**	1510	HeLa	n.d.	n.d.	n.d.	n.d.	[17]
**183**	n.d.	n.d.	n.d.	n.d.	n.d.	n.d.	**-**
**184**	n.d.	n.d.	n.d.	n.d.	n.d.	n.d.	**-**
**185**	720	HCT116	n.d.	n.d.	n.d.	n.d.	[19]
**185**	800	HT-29	n.d.	n.d.	n.d.	n.d.	[19]
**185**	450	U2OS	n.d.	n.d.	n.d.	n.d.	[19]
**185**	550	MDA-MB321	n.d.	n.d.	n.d.	n.d.	[19]
**186**	1890	PNT1A	n.d.	n.d.	n.d.	DNA damage response, down-regulation of *kRAS* and *c-myc* oncogenes expression	[18]
**186**	2190	DU145	n.d.	n.d.	n.d.		[18]
**186**	>>10000	PC-3	n.d.	n.d.	n.d.		[18]
**186**	6560	LNCaP	n.d.	n.d.	n.d.		[18]
**186**	3450	22Rv1	n.d.	n.d.	n.d.		[18]
**186**	0.15	HCT116	n.d.	n.d.	n.d.		[19]
**186**	0.85	HT-29	n.d.	n.d.	n.d.		[19]
**186**	>1	U2OS	n.d.	n.d.	n.d.		[19]
**186**	0.085	MDA-MB321	n.d.	n.d.	n.d.		[19]
**187**	140	HCT116	n.d.	n.d.	n.d.	n.d.	[19]
**187**	790	HT-29	n.d.	n.d.	n.d.	n.d.	[19]
**187**	420	U2OS	n.d.	n.d.	n.d.	n.d.	[19]
**187**	340	MDA-MB321	n.d.	n.d.	n.d.	n.d.	[19]
**188**	0.25	HCT116	n.d.	n.d.	n.d.	n.d.	[19]
**188**	0.29	HT-29	n.d.	n.d.	n.d.	n.d.	[19]
**188**	0.31	U2OS	n.d.	n.d.	n.d.	n.d.	[19]
**188**	0.017	MDA-MB321	n.d.	n.d.	n.d.	n.d.	[19]
**189**	250	HCT116	n.d.	n.d.	n.d.	n.d.	[19]
**189**	>800	HT-29	n.d.	n.d.	n.d.	n.d.	[19]
**189**	250	U2OS	n.d.	n.d.	n.d.	n.d.	[19]
**189**	100	MDA-MB321	n.d.	n.d.	n.d.	n.d.	[19]
**190**	5.7	HCT116	n.d.	n.d.	n.d.	n.d.	[19]
**190**	7.9	HT-29	n.d.	n.d.	n.d.	n.d.	[19]

**190**	8.6	U2OS	n.d.	n.d.	n.d.	n.d.	[19]
**190**	1.3	MDA-MB321	n.d.	n.d.	n.d.	n.d.	[19]
**191**	n.d.	n.d.	n.d.	n.d.	n.d.	n.d.	-
**192**	n.d.	n.d.	n.d.	n.d.	n.d.	n.d.	-
**193**	n.d.	n.d.	n.d.	n.d.	n.d.	n.d.	-
**194**	n.d.	n.d.	n.d.	n.d.	n.d.	n.d.	-
**195**	n.d.	n.d.	n.d.	n.d.	n.d.	n.d.	-
**196**	n.d.	n.d.	n.d.	n.d.	n.d.	n.d.	-
**197**	n.d.	n.d.	n.d.	n.d.	n.d.	n.d.	-
**198**	n.d.	n.d.	n.d.	n.d.	n.d.	n.d.	-
**199**	1000	A549	1300	WI38	1.30	n.d.	[11]
**199**	800	MCF-7	1300	WI38	1.63	n.d.	[11]
**199**	400	MIA-Pa-Ca-2	1300	WI38	3.25	n.d.	[11]
**199**	400	PANC-1	1300	WI38	3.25	n.d.	[11]
**199**	1000	ALT	1300	WI38	1.30	n.d.	[11]
**200**	n.d.	n.d.	n.d.	n.d.	n.d.	n.d.	-
**201**	n.d.	n.d.	n.d.	n.d.	n.d.	Inhibition of telomerase activity	[4]
**202**	n.d.	n.d.	n.d.	n.d.	n.d.	n.d.	-
**203**	n.d.	n.d.	n.d.	n.d.	n.d.	Inhibition of telomerase activity	[5]
**204**	n.d.	n.d.	n.d.	n.d.	n.d.	Inhibition of telomerase activity	[52]
**205**	2300	HeLa	3900	ASF-4-4L2	1.70	n.d.	[53]
**206**	3200	HeLa	2800	ASF-4-4L2	0.880	n.d.	[53]
**207**	2900	HeLa	1900	ASF-4-4L2	0.660	n.d.	[53]
**208**	5200	A549	6100	WI38	1.17	n.d.	[11]
**208**	5500	MCF-7	6100	WI38	1.11	n.d.	[11]
**208**	700	MIA-Pa-Ca-2	6100	WI38	8.71	n.d.	[11]
**208**	1000	PANC-1	6100	WI38	6.10	n.d.	[11]
**208**	3300	ALT	6100	WI38	1.85	n.d.	[11]
**209**	4600	A549	>25000	WI38	>5.43	n.d.	[11]
**209**	19100	MCF-7	>25000	WI38	>1.31	n.d.	[11]
**209**	8600	MIA-Pa-Ca-2	>25000	WI38	>2.91	n.d.	[11]
**209**	8200	PANC-1	>25000	WI38	>3.05	n.d.	[11]
**209**	>25000	ALT	>25000	WI38	~1.00	n.d.	[11]
**210**	10400	A549	>25000	WI38	>2.40	n.d.	[11]
**210**	>25000	MCF-7	>25000	WI38	~1.00	n.d.	[11]
**210**	19300	MIA-Pa-Ca-2	>25000	WI38	>1.30	n.d.	[11]
**210**	13600	PANC-1	>25000	WI38	>1.84	n.d.	[11]
**210**	14900	ALT	>25000	WI38	>1.68	n.d.	[11]
**211**	500	A549	2900	WI38	5.80	n.d.	[11]
**211**	1200	MCF-7	2900	WI38	2.42	n.d.	[11]
**211**	1000	MIA-Pa-Ca-2	2900	WI38	2.90	n.d.	[11]
**211**	600	PANC-1	2900	WI38	4.83	n.d.	[11]
**211**	1100	ALT	2900	WI38	2.64	n.d.	[11]
**212**	n.d.	n.d.	n.d.	n.d.	n.d.	n.d.	-
**213**	n.d.	n.d.	n.d.	n.d.	n.d.	n.d.	-
**214**	n.d.	n.d.	n.d.	n.d.	n.d.	Inhibition of telomerase activity	[52]
**215**	n.d.	n.d.	n.d.	n.d.	n.d.	n.d.	-
**216**	n.d.	n.d.	n.d.	n.d.	n.d.	n.d.	-

Table S6. Overview of the in vivo studies on NDIs. MTD = maximum tolerated dose; TVI = tumour volume inhibition; n.d. = not defined.

Compound	Xenograft model	MTD	Administration	Therapeutic regimen	TVI (days of treatment)	Adverse effects	Refs.
**43**	HT-29 (colon)	n.d.	Intraperitoneal, dissolved in PBS	11 mg/kg, 3 times a week	None	Body weight loss	16
**52**	MZ CRC-1 (thyroid)	n.d.	Intraperitoneal, dissolved in PBS	12 mg/kg, every 2 days	50% (55)	Body weight loss	25
	TT (thyroid)	n.d.	Intraperitoneal, dissolved in PBS	12 mg/kg, every 2 days	37% (58)	Body weight loss	25
**85**	HT-29 (colon)	n.d.	Intraperitoneal, dissolved in PBS	39 mg/kg, 3 times a week	35% (12)	Body weight loss	16
**93**	PDAC (pancreas)	50 mg/kg	Intravenous, dissolved in saline	10 mg/kg, 2 times a week	52% (62)	None	29,30
	PDAC (pancreas)	50 mg/kg	Intravenous, dissolved in saline	15 mg/kg, 2 times a week	85% (62)	None	29,30
	MIA-Pa-Ca-2 (pancreas)	n.d.	Intravenous, dissolved in PBS	10 mg/kg, 2 times a week	61% (53)	None	47
	MIA-Pa-Ca-2 (pancreas)	n.d.	Intravenous, dissolved in PBS	15 mg/kg, 2 times a week	78% (53)	None	47
**153**	MIA-Pa-Ca-2 (pancreas)	15 mg/kg	Intravenous, dissolved in PBS	3 mg/kg, 3 times a week	50% (26)	None	45
	HPAC (pancreas)	15 mg/kg	Intravenous, dissolved in PBS	3 mg/kg, 3 times a week	30% (34)	None	45
**159**	MIA-Pa-Ca-2 (pancreas)	30 mg/kg	Intravenous, dissolved in saline	10 mg/kg, 2 times a week	60% (40)	None	39,46
	MIA-Pa-Ca-2 (pancreas)	30 mg/kg	Intravenous, dissolved in saline	15 mg/kg, 2 times a week	80% (40)	None	39,46
	PDAC (pancreas)	30 mg/kg	Intravenous, dissolved in saline	15 mg/kg, 2 times a week	57% (62)	None	29,30
**162**	MIA-Pa-Ca-2 (pancreas)	n.d.	Intravenous, dissolved in PBS	1 mg/kg, once a week	89% (53)	None	47
	MIA-Pa-Ca-2 (pancreas)	n.d.	Intravenous, dissolved in PBS	1 mg/kg, 2 times a week	91% (53)	None	47

Table S7. Overview of the crystallographic and molecular modelling studies on NDIs. Figures of the structures of the DNA/NDI complexes were reprinted with permission from: ref. 1, Copyright (2008), Elsevier; ref. 4, Copyright (1996), Royal Society of Chemistry; ref. 10, Copyright (2017), Elsevier; ref. 11, Copyright (2015), Elsevier; ref. 13, Copyright (2015), American Chemical Society; ref. 20, Copyright (2021), Elsevier; ref. 27, Copyright (2016), John Wiley and Sons; ref. 30, Copyright (2018), American Chemical Society; ref. 31, Copyright (2020), American Chemical Society; ref. 35, Copyright (2008), Elsevier; ref. 37, Copyright (2013), Elsevier; ref. 38, Copyright (2013), American Chemical Society; ref. 40, Copyright (2009), American Chemical Society; ref. 42, Copyright (2012), American Chemical Society; ref. 53, Copyright (2019), John Wiley and Sons.

Compound	Sequence 5′→3′<br>(length in nucleotides per strand)	G-quadruplex/<br>duplex topology<br>(location in the DNA)	Structure of the DNA/NDI complex	Refs.
**Crystallographic studies**				
**109**	d[TAGGG(TTAGGG)$_3$]<br>(23-mer)	Parallel unimolecular<br>G-quadruplex<br>(telomere)	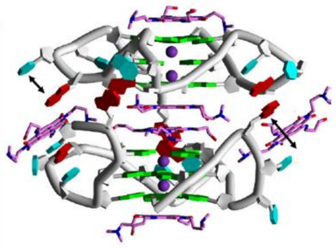	35
**153**	d[AGGG(TTAGGG)$_3$]<br>(22-mer)	Parallel unimolecular<br>G-quadruplex<br>(telomere)	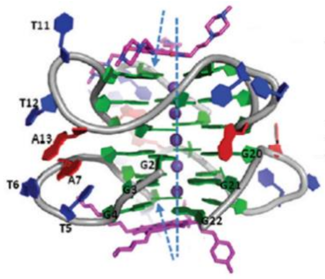	42
**159**	d[AGGG(TTAGGG)$_3$]<br>(22-mer)	Parallel unimolecular<br>G-quadruplex<br>(telomere)	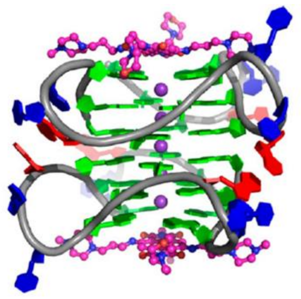	38
**160**	d[AGGG(TTAGGG)$_3$]<br>(22-mer)	Parallel unimolecular<br>G-quadruplex<br>(telomere)	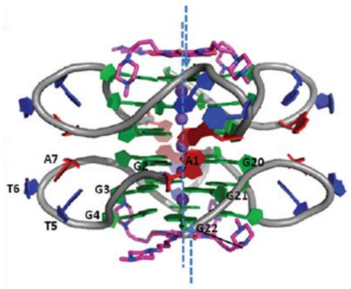	42
**Molecular modelling studies**				
**18**	d[TAGGG(TTAGGG)$_3$]<br>(23-mer)	Hybrid-1 unimolecular<br>G-quadruplex<br>(telomere)	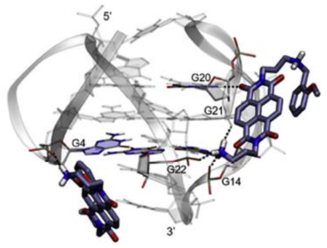	10

**18**	d[AGGG(TTAGGG)$_3$T]<br>(23-mer)	Hybrid-2 unimolecular<br>G-quadruplex<br>(telomere)	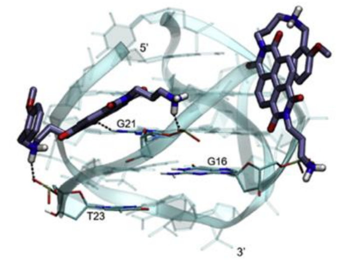	10
**18**	d(GGATGTGAGTGTGAG TGTGAGGG)<br>d(CCCTCACACTCACACTCACATCC)<br>(23-mer)	Bimolecular<br>B-duplex<br>(all over DNA)	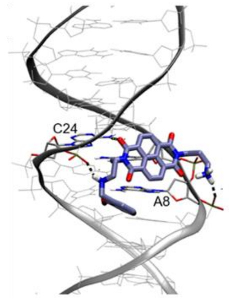	10
**20**	d[TAGGG(TTAGGG)$_3$]<br>(23-mer)	Hybrid-1 unimolecular<br>G-quadruplex<br>(telomere)	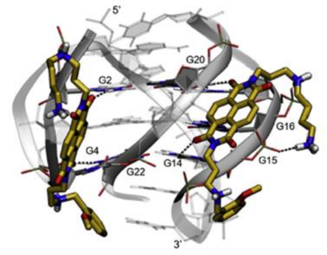	10
**20**	d[AGGG(TTAGGG)$_3$T]<br>(23-mer)	Hybrid-2 unimolecular<br>G-quadruplex<br>(telomere)	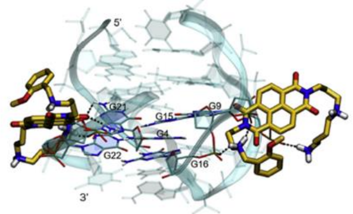	10
**20**	d(GGATGTGAGTGTGAG TGTGAGGG)<br>d(CCCTCACACTCACACTCACATCC)<br>(23-mer)	Bimolecular<br>B-duplex<br>(all over DNA)	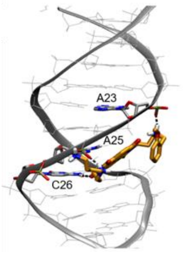	10
**23**	d[TAGGG(TTAGGG)$_3$]<br>(23-mer)	Hybrid-1 unimolecular<br>G-quadruplex<br>(telomere)	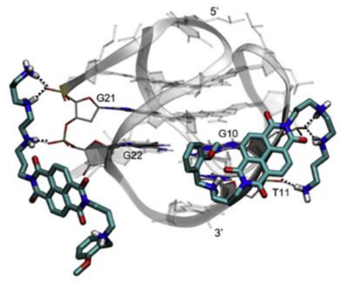	10

**23**	d[AGGG(TTAGGG)$_3$T]<br>(23-mer)	Hybrid-2 unimolecular<br>G-quadruplex<br>(telomere)	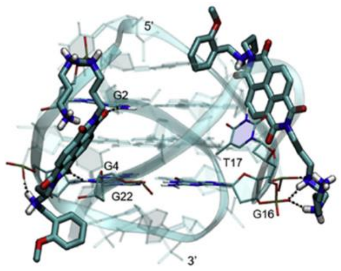	10
**23**	d(GGATGTGAGTGTGAG TGTGAGGG)<br>d(CCCTCACACTCACACTCACATCC)<br>(23-mer)	Bimolecular<br>B-duplex<br>(all over DNA)	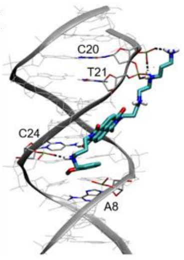	10
**33**	d[AGGG(TTAGGG)$_3$]<br>(22-mer)	Parallel unimolecular<br>G-quadruplex<br>(telomere)	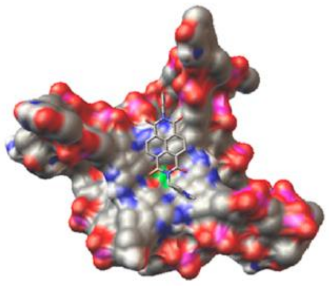	13
**34**	d[AGGG(TTAGGG)$_3$]<br>(22-mer)	Parallel unimolecular<br>G-quadruplex<br>(telomere)	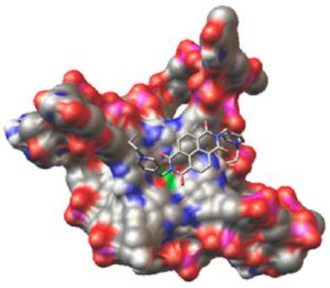	13
**39**	d[AGGG(TTAGGG)$_3$]<br>(22-mer)	Parallel unimolecular<br>G-quadruplex<br>(telomere)	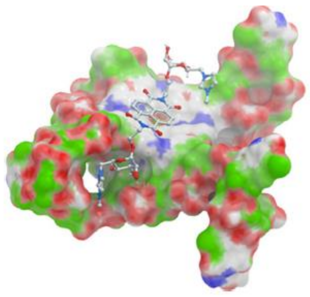	6
**46**	d[GGG(TTAGGG)$_7$]<br>(45-mer)	Hybrid unimolecular dimeric<br>G-quadruplex<br>(telomere)	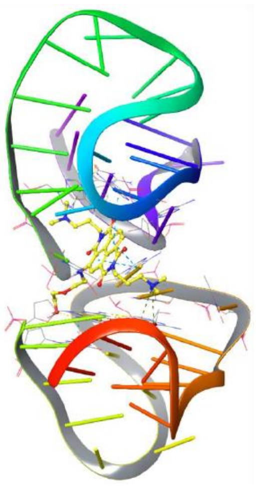	20

**93**	d[AGGG(TTAGGG)$_3$]<br>(22-mer)	Parallel unimolecular<br>G-quadruplex<br>(telomere)	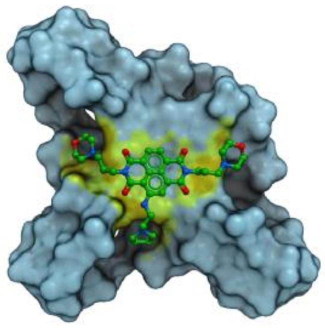	30
**93**	d(AIGGGAGGGICTGGGAGGGC)<br>(20-mer)	Parallel unimolecular<br>G-quadruplex<br>(*hTERT* promoter)	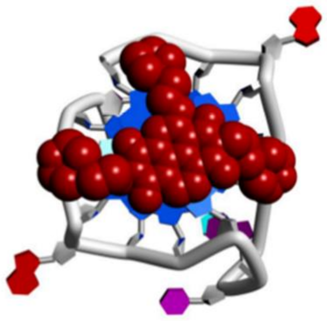	31
**116**	d[GGG(TTAGGG)$_3$]<br>(21-mer)	Parallel unimolecular<br>G-quadruplex<br>(telomere)	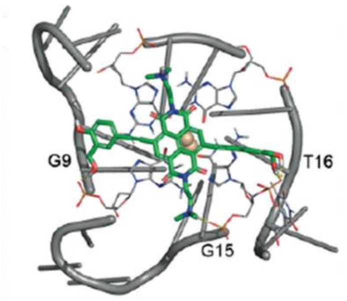	27
**117**	d[GGG(TTAGGG)$_3$]<br>(21-mer)	Parallel unimolecular<br>G-quadruplex<br>(telomere)	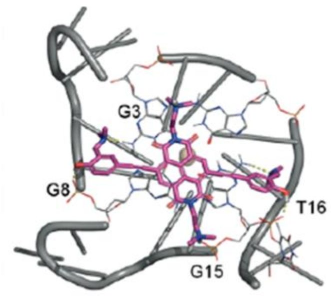	27
**118**	d[GGG(TTAGGG)$_3$]<br>(21-mer)	Parallel unimolecular<br>G-quadruplex<br>(telomere)	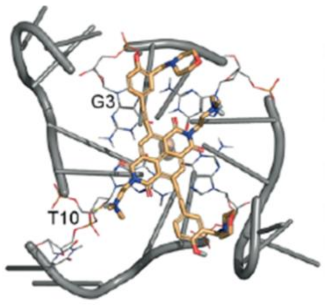	27
**145**	d[TAGGG(TTAGGG)$_3$]<br>(23-mer)	Parallel unimolecular<br>G-quadruplex<br>(telomere)	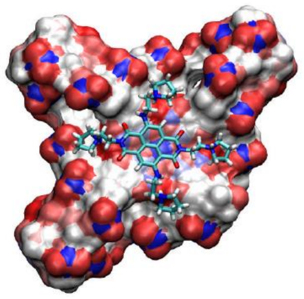	40

**152**	d[AGGG(TTAGGG)$_3$]<br>(22-mer)	Parallel unimolecular<br>G-quadruplex<br>(telomere)	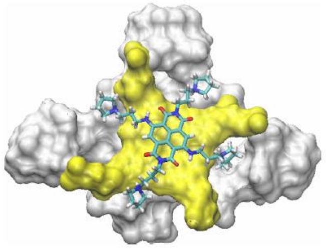	1
**153**	d[AGGG(TTAGGG)$_3$]<br>(22-mer)	Parallel unimolecular<br>G-quadruplex<br>(telomere)	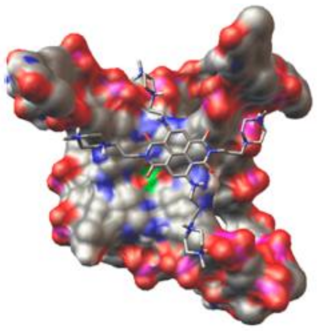	13,43,44
**158**	d[GGG(TTAGGG)$_3$]<br>(21-mer)	Parallel unimolecular<br>G-quadruplex<br>(telomere)	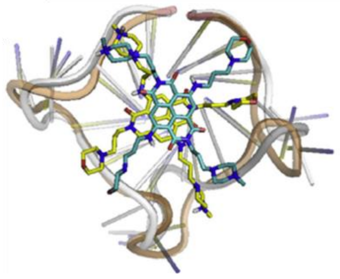	37
**159**	d[GGG(TTAGGG)$_3$]<br>(21-mer)	Parallel unimolecular<br>G-quadruplex<br>(telomere)	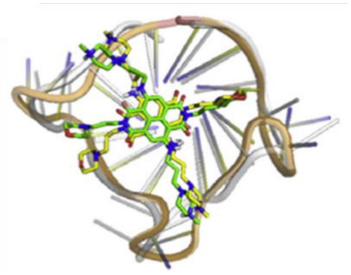	37
**159**	d[AGGG(TTAGGG)$_3$]<br>(22-mer)	Parallel unimolecular<br>G-quadruplex<br>(telomere)	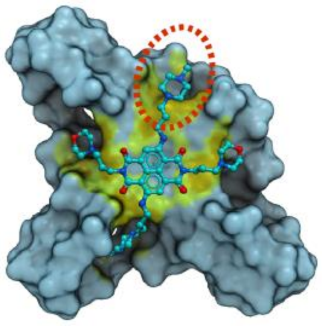	30
**159**	d(AIGGGAGGGICTGGGAGGGC)<br>(20-mer)	Parallel unimolecular<br>G-quadruplex<br>(*hTERT* promoter)	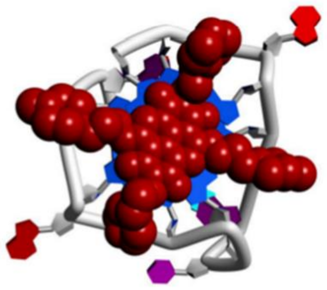	31

**159**	d(GGGCGCGGAGGAAGGGGGCGGG)<br>(22-mer)	Parallel unimolecular<br>G-quadruplex<br>(*bcl-2* promoter)	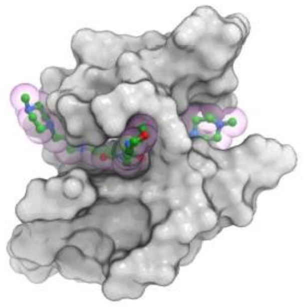	46
**160**	d[AGGG(TTAGGG)$_3$]<br>(22-mer)	Parallel unimolecular<br>G-quadruplex<br>(telomere)	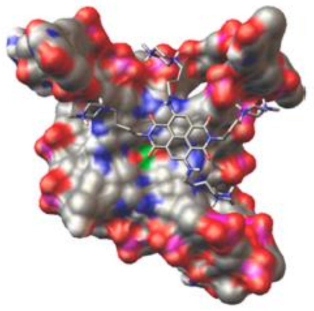	13,43,44
**162**	d[TTGGG(TTAGGG)$_3$A]<br>(24-mer)	Hybrid-1 unimolecular<br>G-quadruplex<br>(telomere)	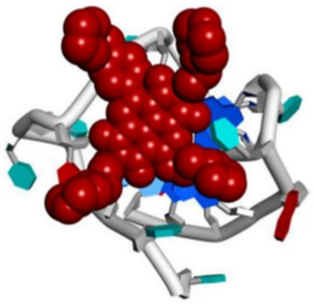	31
**162**	d(AIGGGAGGGICTGGGAGGGC)<br>(20-mer)	Parallel unimolecular<br>G-quadruplex<br>(*hTERT* promoter)	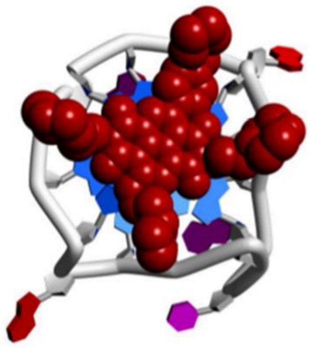	31
**182**	d[GGG(TTAGGG)$_7$]<br>(45-mer)	Hybrid unimolecular dimeric<br>G-quadruplex<br>(telomere)	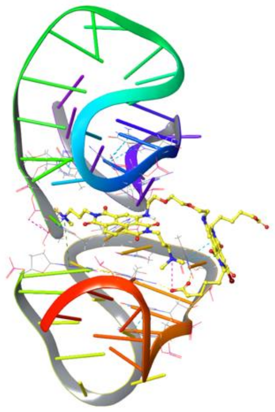	20

**199**	d(TAGGGTTAGGGT)<br>(12-mer)	Parallel bimolecular<br>G-quadruplex<br>(telomere)	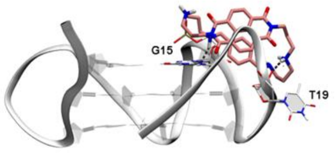	11
**199**	d(CGATCG)<br>(6-mer)	Bimolecular<br>B-duplex<br>(all over DNA)	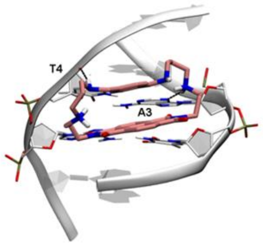	11
**201**	d[TTGGG(TTAGGG)$_3$A]<br>(24-mer)	Hybrid-1 unimolecular<br>G-quadruplex<br>(telomere)	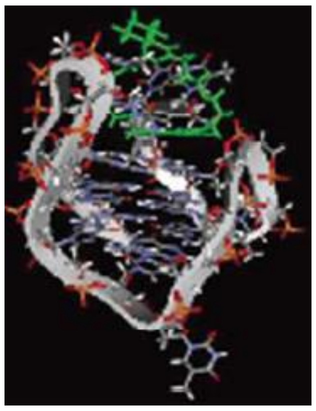	4
**203**	d[TTGGG(TTAGGG)$_3$A]<br>(24-mer)	Hybrid-1 unimolecular<br>G-quadruplex<br>(telomere)	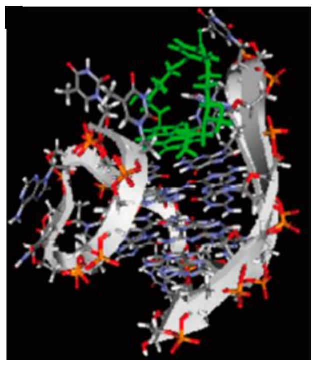	5
**205**	d[TTGGG(TTAGGG)$_3$A]<br>(24-mer)	Hybrid-1 unimolecular<br>G-quadruplex<br>(telomere)	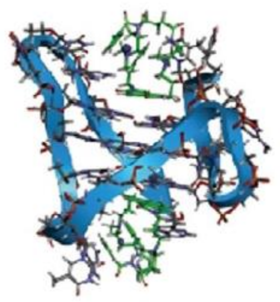	53

**208**	d(TAGGGTTAGGGT)<br>(12-mer)	Parallel bimolecular<br>G-quadruplex<br>(telomere)	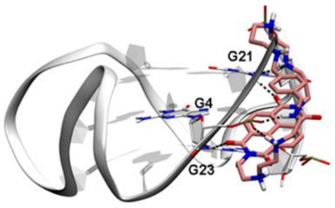	11
**208**	d(CGATCG)<br>(6-mer)	Bimolecular<br>B-duplex<br>(all over DNA)	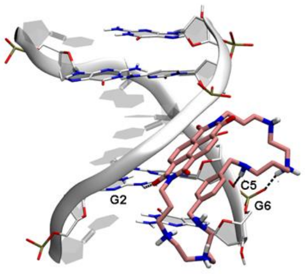	11
**209**	d(TAGGGTTAGGGT)<br>(12-mer)	Parallel bimolecular<br>G-quadruplex<br>(telomere)	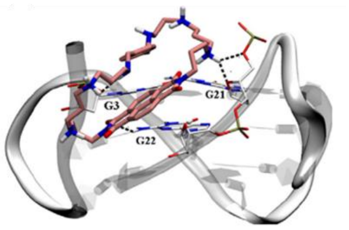	11
**209**	d(CGATCG)<br>(6-mer)	Bimolecular<br>B-duplex<br>(all over DNA)	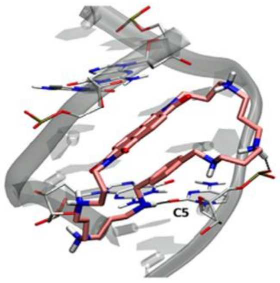	11
**210**	d(TAGGGTTAGGGT)<br>(12-mer)	Parallel bimolecular<br>G-quadruplex<br>(telomere)	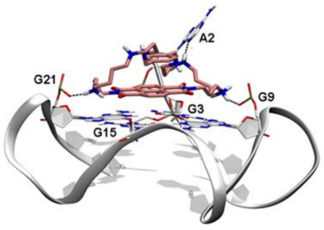	11
**210**	d(CGATCG)<br>(6-mer)	Bimolecular<br>B-duplex<br>(all over DNA)	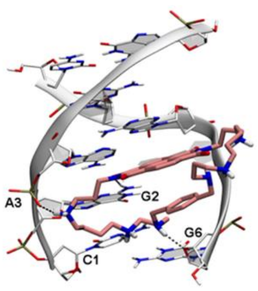	11
**211**	d(TAGGGTTAGGGT)<br>(12-mer)	Parallel bimolecular<br>G-quadruplex<br>(telomere)	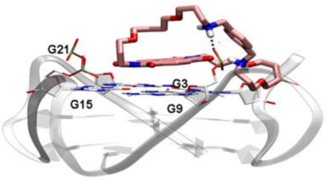	11

**211**	d(CGATCG)<br>(6-mer)	Bimolecular<br>B-duplex<br>(all over DNA)	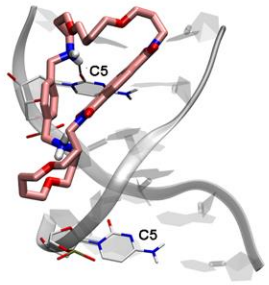	11
**212**	d[TTGGG(TTAGGG)$_3$A]<br>(24-mer)	Hybrid-1 unimolecular<br>G-quadruplex<br>(telomere)	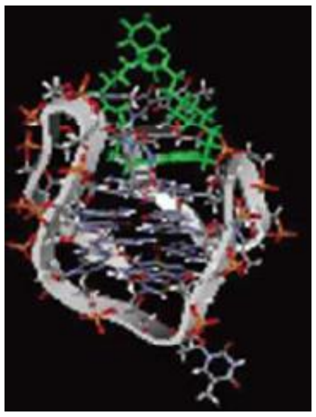	4

References

(1) Cuenca, F.; Greciano, O.; Gunaratnam, M.; Haider, S.; Munnur, D.; Nanjunda, R.; Wilson, W. D.; Neidle, S. Tri- and Tetra-Substituted Naphthalene Diimides as Potent G-Quadruplex Ligands. *Bioorg. Med. Chem. Lett.* **2008**, *18*, 1668–1673.

(2) Gunaratnam, M.; Cuenca, F.; Neidle, S. Naphthalene Diimide Compounds, US8,796,456B2, **2014**.

(3) Czerwinska, I.; Sato, S.; Juskowiak, B.; Takenaka, S. Interactions of Cyclic and Non-Cyclic Naphthalene Diimide Derivatives with Different Nucleic Acids. *Bioorganic Med. Chem.* **2014**, *22*, 2593–2601.

(4) Esaki, Y.; Islam, M. M.; Fujii, S.; Sato, S.; Takenaka, S. Design of Tetraplex Specific Ligands: Cyclic Naphthalene Diimide. *Chem. Commun.* **2014**, *50*, 5967–5969.

(5) Islam, M. M.; Fujii, S.; Sato, S.; Okauchi, T.; Takenaka, S. A Selective G-Quadruplex DNA-Stabilizing Ligand Based on a Cyclic Naphthalene Diimide Derivative. *Molecules* **2015**, *20*, 10963–10979.

(6) Street, S. T. G.; Chin, D. N.; Hollingworth, G. J.; Berry, M.; Morales, J. C.; Galan, M. C. Divalent Naphthalene Diimide Ligands Display High Selectivity for the Human Telomeric G-Quadruplex in K^+ Buffer. *Chem. Eur. J.* **2017**, *23*, 6953–6958.

(7) Pasini, A.; Marchetti, C.; Sissi, C.; Cortesi, M.; Giordano, E.; Minarini, A.; Milelli, A. Novel Polyamine-Naphthalene Diimide Conjugates Targeting Histone Deacetylases and DNA for Cancer Phenotype Reprogramming. *ACS Med. Chem. Lett.* **2017**, *8*, 1218–1223.

(8) Răsădean, D. M.; Sheng, B.; Dash, J.; Pantoş, G. D. Amino-Acid-Derived Naphthalenediimides as Versatile G-Quadruplex Binders. *Chem. Eur. J.* **2017**, *23*, 8491–8499.

(9) Răsădean, D. M.; Harrison, S. W. O.; Owens, I. R.; Bromley, F. M.; Pantos, G. D. Importance of Chiral Recognition in Designing Metal-Free Ligands for G-Quadruplex DNA. *Molecules* **2019**, *24*, 1473.

(10) Milelli, A.; Marchetti, C.; Greco, M. L.; Moraca, F.; Costa, G.; Turrini, E.; Catanzaro, E.; Betari, N.; Calcabrini, C.; Sissi, C.; Alcaro, S.; Fimognari, C.; Tumiatti, V.; Minarini, A. Naphthalene Diimide-Polyamine Hybrids as Antiproliferative Agents: Focus on the Architecture of the Polyamine Chains. *Eur. J. Med. Chem.* **2017**, *128*, 107–122.

(11) Marchetti, C.; Minarini, A.; Tumiatti, V.; Moraca, F.; Parrotta, L.; Alcaro, S.; Rigo, R.; Sissi, C.; Gunaratnam, M.; Ohnmacht, S. A.; Neidle, S.; Milelli, A. Macrocyclic Naphthalene Diimides as G-Quadruplex Binders. *Bioorganic Med. Chem.* **2015**, *23*, 3819–3830.

(12) Sur, S.; Tiwari, V.; Sinha, D.; Kamran, M. Z.; Dubey, K. D.; Suresh Kumar, G.; Tandon, V. Naphthalenediimide-Linked Bisbenzimidazole Derivatives as Telomeric G-Quadruplex-Stabilizing Ligands with Improved Anticancer Activity. *ACS Omega* **2017**, *2*, 966–980.

(13) Prato, G.; Silvent, S.; Saka, S.; Lamberto, M.; Kosenkov, D. Thermodynamics of Binding of Di- and Tetrasubstituted Naphthalene Diimide Ligands to DNA G-Quadruplex. *J. Phys. Chem. B* **2015**, *119*, 3335–3347.

(14) Nadai, M.; Doria, F.; Scalabrin, M.; Pirota, V.; Grande, V.; Bergamaschi, G.; Amendola, V.; Winnerdy, F. R.; Phan, A. T.; Richter, S. N.; Freccero, M. A Catalytic and Selective Scissoring Molecular Tool for Quadruplex Nucleic Acids. *J. Am. Chem. Soc.* **2018**, *140*, 14528–14532.

(15) Arévalo-Ruiz, M.; Doria, F.; Belmonte-Reche, E.; De Rache, A.; Campos-Salinas, J.; Lucas, R.; Falomir, E.; Carda, M.; Pérez-Victoria, J. M.; Mergny, J. L.; Freccero, M.; Morales, J. C. Synthesis, Binding Properties, and Differences in Cell Uptake of G-Quadruplex Ligands Based on Carbohydrate Naphthalene Diimide Conjugates. *Chem. Eur. J.* **2017**, *23*, 2157–2164.

(16) Morales Sanchez, J. C.; Perez-Victoria Moreno De Barreda, J. M.; Arevalo Ruiz, M.; Belmonte Reche, E.; Martinez Garcia, M.; Lucas Rodriguez, R.; Freccero, M.; Doria, F.; Carda Usò, P. M.; Falomir Ventura, E.; Lopez Rubio, J. J. Naphthalene Diimide Compound for Treatment of Diseases. WO2018/060423A1, **2018**.

(17) Platella, C.; Pirota, V.; Musumeci, D.; Rizzi, F.; Iachettini, S.; Zizza, P.; Biroccio, A.; Freccero, M.; Montesarchio, D.; Doria, F. Trifunctionalized Naphthalene Diimides and Dimeric Analogues as G-Quadruplex-Targeting Anticancer Agents Selected by Affinity Chromatography. *Int. J. Mol. Sci.* **2020**, *21*, 1964.

(18) Tassinari, M.; Cimino-Reale, G.; Nadai, M.; Doria, F.; Butovskaya, E.; Recagni, M.; Freccero, M.; Zaffaroni, N.; Richter, S. N.; Folini, M. Down-Regulation of the Androgen Receptor by G-Quadruplex Ligands Sensitizes Castration-Resistant Prostate Cancer Cells to Enzalutamide. *J. Med. Chem.* **2018**, *61*, 8625–8638.

(19) Doria, F.; Salvati, E.; Pompili, L.; Pirota, V.; D'Angelo, C.; Manoli, F.; Nadai, M.; Richter, S. N.; Biroccio, A.; Manet, I.; Freccero, M. Dyads of G-Quadruplex Ligands Triggering DNA Damage Response and Tumour Cell Growth Inhibition at Sub-nM Concentration. *Chem. Eur. J.* **2019**, *25*, 11085–11097.

(20) Pirota, V.; Platella, C.; Musumeci, D.; Benassi, A.; Amato, J.; Pagano, B.; Colombo, G.; Freccero, M.; Doria, F.; Montesarchio, D. On the Binding of Naphthalene Diimides to a Human Telomeric G-Quadruplex Multimer Model. *Int. J. Biol. Macromol.* **2021**, *166*, 1320-1334.

(21) Platella, C.; Trajkovski, M.; Doria, F.; Freccero, M.; Montesarchio, D.; Plavec, J. On the Interaction of an Anticancer Trisubstituted Naphthalene

Diimide with G-Quadruplexes of Different Topologies: A Structural Insight. *Nucleic Acids Res.* **2020**, *48*, 12380-12393.

(22) Muoio, D.; Berardinelli, F.; Leone, S.; Coluzzi, E.; di Masi, A.; Doria, F.; Freccero, M.; Sgura, A.; Folini, M.; Antoccia, A. Naphthalene Diimide-Derivatives G-Quadruplex Ligands Induce Cell Proliferation Inhibition, Mild Telomeric Dysfunction and Cell Cycle Perturbation in U251MG Glioma Cells. *FEBS J.* **2018**, *285*, 3769–3785.

(23) Doria, F.; Nadai, M.; Folini, M.; Di Antonio, M.; Germani, L.; Percivalle, C.; Sissi, C.; Zaffaroni, N.; Alcaro, S.; Artese, A.; Richter, S. N.; Freccero, M. Hybrid Ligand-Alkylating Agents Targeting Telomeric G-Quadruplex Structures. *Org. Biomol. Chem.* **2012**, *10*, 2798–2806.

(24) Nadai, M.; Cimino-Reale, G.; Sattin, G.; Doria, F.; Butovskaya, E.; Zaffaroni, N.; Freccero, M.; Palumbo, M.; Richter, S. N.; Folini, M. Assessment of Gene Promoter G-Quadruplex Binding and Modulation by a Naphthalene Diimide Derivative in Tumor Cells. *Int. J. Oncol.* **2015**, *46*, 369–380.

(25) Lopergolo, A.; Perrone, R.; Tortoreto, M.; Doria, F.; Beretta, G. L.; Zuco, V.; Freccero, M.; Borrello, M. G.; Lanzi, C.; Richter, S. N.; Zaffaroni, N.; Folini, M. Targeting of RET Oncogene by Naphthalene Diimide-Mediated Gene Promoter G-Quadruplex Stabilization Exerts Anti-Tumor Activity in Oncogene-Addicted Human Medullary Thyroid Cancer. *Oncotarget* **2016**, *7*, 49649–49663.

(26) Nadai, M.; Doria, F.; Di Antonio, M.; Sattin, G.; Germani, L.; Percivalle, C.; Palumbo, M.; Richter, S. N.; Freccero, M. Naphthalene Diimide Scaffolds with Dual Reversible and Covalent Interaction Properties towards G-Quadruplex. *Biochimie* **2011**, *93*, 1328–1340.

(27) Doria, F.; Nadai, M.; Costa, G.; Sattin, G.; Gallati, C.; Bergamaschi, G.; Moraca, F.; Alcaro, S.; Freccero, M.; Richter, S. N. Extended Naphthalene Diimides with Donor/Acceptor Hydrogen-Bonding Properties Targeting G-Quadruplex Nucleic Acids. *European J. Org. Chem.* **2016**, *2016*, 4824–4833.

(28) Wei, H.; Lv, M.; Duan, X.; Li, S.; Yao, Y.; Wang, K.; Zhang, P.; Li, X.; Chen, H. Cytotoxicity and DNA-Binding Property of Water-Soluble Naphthalene Diimide Derivatives Bearing 2-Oligoethoxy Ethanamine Side Chain End-Labeled with Tertiary Amino Groups. *Med. Chem. Res.* **2014**, *23*, 2277–2286.

(29) Neidle, S.; Marchetti, C.; Ohnmacht, S. A. Substituted Naphthalene Diimides and Their Use. WO2017/103587A1, **2017**.

(30) Marchetti, C.; Zyner, K. G.; Ohnmacht, S. A.; Robson, M.; Haider, S. M.; Morton, J. P.; Marsico, G.; Vo, T.; Laughlin-Toth, S.; Ahmed, A. A.; Di Vita, G.; Pazitna, I.; Gunaratnam, M.; Besser, R. J.; Andrade, A. C. G.; Diocou, S.; Pike, J. A.; Tannahill, D.; Pedley, R. B.; Evans, T. R. J.; Wilson, W. D.; Balasubramanian, S.; Neidle, S. Targeting Multiple Effector Pathways in Pancreatic Ductal Adenocarcinoma with a G-Quadruplex-Binding Small Molecule. *J. Med. Chem.* **2018**, *61*, 2500–2517.

(31) Vo, T.; Oxenford, S.; Angell, R.; Marchetti, C.; Ohnmacht, S. A.; Wilson, W. D.; Neidle, S. Substituted Naphthalenediimide Compounds Bind Selectively to Two Human Quadruplex Structures with Parallel Topology. *ACS Med. Chem. Lett.* **2020**, *11*, 991–999.

(32) Ahmed, A. A.; Marchetti, C.; Ohnmacht, S. A.; Neidle, S. A G-Quadruplex-Binding Compound Shows Potent Activity in Human Gemcitabine-Resistant Pancreatic Cancer Cells. *Sci. Rep.* **2020**, *10*, 12192.

(33) Salvati, E.; Doria, F.; Manoli, F.; D'Angelo, C.; Biroccio, A.; Freccero, M.; Manet, I. A Bimodal Fluorescent and Photocytotoxic Naphthalene Diimide for Theranostic Applications. *Org. Biomol. Chem.* **2016**, *14*, 7238–7249.

(34) Hou, J. Q.; Chen, S. Bin; Tan, J. H.; Luo, H. Bin; Li, D.; Gu, L. Q.; Huang, Z. S. New Insights from Molecular Dynamic Simulation Studies of the Multiple Binding Modes of a Ligand with G-Quadruplex DNA. *J. Comput. Aided. Mol. Des.* **2012**, *26*, 1355–1368.

(35) Parkinson, G. N.; Cuenca, F.; Neidle, S. Topology Conservation and Loop Flexibility in Quadruplex-Drug Recognition: Crystal Structures of Inter- and Intramolecular Telomeric DNA Quadruplex-Drug Complexes. *J. Mol. Biol.* **2008**, *381*, 1145–1156.

(36) Zuffo, M.; Ladame, S.; Doria, F.; Freccero, M. Tuneable Coumarin-NDI Dyads as G-Quadruplex Specific Light-up Probes. *Sensors Actuators, B Chem.* **2017**, *245*, 780–788.

(37) Mpima, S.; Ohnmacht, S. A.; Barletta, M.; Husby, J.; Pett, L. C.; Gunaratnam, M.; Hilton, S. T.; Neidle, S. The Influence of Positional Isomerism on G-Quadruplex Binding and Anti-Proliferative Activity of Tetra-Substituted Naphthalene Diimide Compounds. *Bioorganic Med. Chem.* **2013**, *21*, 6162–6170.

(38) Micco, M.; Collie, G. W.; Dale, A. G.; Ohnmacht, S. A.; Pazitna, I.; Gunaratnam, M.; Reszka, A. P.; Neidle, S. Structure-Based Design and Evaluation of Naphthalene Diimide G-Quadruplex Ligands as Telomere Targeting Agents in Pancreatic Cancer Cells. *J. Med. Chem.* **2013**, *56*, 2959–2974.

(39) Neidle, S.; Ohnmacht, S. A.; Gunaratnam, M.; Dale, A. G.; Micco, M.; Collie, G. W. Diimide Compounds. US9,493,460B2, **2016**.

(40) Gunaratnam, M.; Beltran, M.; Galesa, K.; Haider, S. M.; Anthony, P.; Cuenca, F.; Fletcher, J. A.; Neidle, S. Targeting Human Gastrointestinal Stromal Tumour Cells with a Quadruplex-Binding Small Molecule. *J. Med. Chem.* **2009**, *52*, 3774–3783.

(41) Gunaratnam, M.; Collie, G. W.; Reszka, A. P.; Todd, A. K.; Parkinson, G. N.; Neidle, S. A Naphthalene Diimide G-Quadruplex Ligand Inhibits Cell Growth and Down-Regulates BCL-2 Expression in an Imatinib-Resistant Gastrointestinal Cancer Cell Line. *Bioorganic Med. Chem.* **2018**,

26, 2958–2964.

(42) Collie, G. W.; Promontorio, R.; Hampel, S. M.; Micco, M.; Neidle, S.; Parkinson, G. N. Structural Basis for Telomeric G-Quadruplex Targeting by Naphthalene Diimide Ligands. *J. Am. Chem. Soc.* **2012**, *134*, 2723–2731.

(43) Hampel, S. M.; Sidibe, A.; Gunaratnam, M.; Riou, J. F.; Neidle, S. Tetrasubstituted Naphthalene Diimide Ligands with Selectivity for Telomeric G-Quadruplexes and Cancer Cells. *Bioorganic Med. Chem. Lett.* **2010**, *20*, 6459–6463.

(44) Spinello, A.; Barone, G.; Grunenberg, J. Molecular Recognition of Naphthalene Diimide Ligands by Telomeric Quadruplex-DNA: The Importance of the Protonation State and Mediated Hydrogen Bonds. *Phys. Chem. Chem. Phys.* **2015**, *18*, 2871–2877.

(45) Gunaratnam, M.; de la Fuente, M.; Hampel, S. M.; Todd, A. K.; Reszka, A. P.; Schätzlein, A.; Neidle, S. Targeting Pancreatic Cancer with a G-Quadruplex Ligand. *Bioorganic Med. Chem.* **2011**, *19*, 7151–7157.

(46) Ohnmacht, S. A.; Marchetti, C.; Gunaratnam, M.; Besser, R. J.; Haider, S. M.; Di Vita, G.; Lowe, H. L.; Mellinas-Gomez, M.; Diocou, S.; Robson, M.; Šponer, J.; Islam, B.; Barbara Pedley, R.; Hartley, J. A.; Neidle, S. A G-Quadruplex-Binding Compound Showing Anti-Tumour Activity in an in Vivo Model for Pancreatic Cancer. *Sci. Rep.* **2015**, *5*, 11385.

(47) Ahmed, A. A.; Angell, R.; Oxenford, S.; Worthington, J.; Williams, N.; Barton, N.; Fowler, T. G.; O'Flynn, D. E.; Sunose, M.; McConville, M.; Vo, T.; Wilson, W. D.; Karim, S. A.; Morton, J. P.; Neidle, S. Asymmetrically Substituted Quadruplex-Binding Naphthalene Diimide Showing Potent Activity in Pancreatic Cancer Models. *ACS Med. Chem. Lett.* **2020**, *11*, 1634–1644.

(48) Doria, F.; Nadai, M.; Sattin, G.; Pasotti, L.; Richter, S. N.; Freccero, M. Water Soluble Extended Naphthalene Diimides as pH Fluorescent Sensors and G-Quadruplex Ligands. *Org. Biomol. Chem.* **2012**, *10*, 3830–3840.

(49) Recagni, M.; Tassinari, M.; Doria, F.; Cimino-Reale, G.; Zaffaroni, N.; Freccero, M.; Folini, M.; Richter, S.N. The Oncogenic Signaling Pathways in BRAF-Mutant Melanoma Cells Are Modulated by Naphthalene Diimide-like G-Quadruplex Ligands. *Cells* **2019**, *8*, 1274.

(50) Zuffo, M.; Guédin, A.; Leriche, E. D.; Doria, F.; Pirota, V.; Gabelica, V.; Mergny, J. L.; Freccero, M. More Is Not Always Better: Finding the Right Trade-off between Affinity and Selectivity of a G-Quadruplex Ligand. *Nucleic Acids Res.* **2018**, *46*, e115.

(51) Zou, T.; Sato, S.; Yasukawa, R.; Takeuchi, R.; Ozaki, S.; Fujii, S.; Takenaka, S. The Interaction of Cyclic Naphthalene Diimide with G-Quadruplex under Molecular Crowding Condition. *Molecules* **2020**, *25*, 668.

(52) Takeuchi, R.; Zou, T.; Wakahara, D.; Nakano, Y.; Sato, S.; Takenaka, S. Cyclic Naphthalene Diimide Dimer with a Strengthened Ability to Stabilize Dimeric G-Quadruplex. *Chem. Eur. J.* **2019**, *25*, 8691–8695.

(53) Kaneyoshi, S.; Zou, T.; Ozaki, S.; Takeuchi, R.; Udou, A.; Nakahara, T.; Fujimoto, K.; Fujii, S.; Sato, S.; Takenaka, S. Cyclic Naphthalene Diimide with a Ferrocene Moiety as a Redox-Active Tetraplex-DNA Ligand. *Chem. Eur. J.* **2020**, *26*, 139–142.

(54) Hampel, S. M.; Pepe, A.; Greulich-Bode, K. M.; Malhotra, S. V.; Reszka, A. P.; Veith, S.; Boukamp, P.; Neidle, S. Mechanism of the Antiproliferative Activity of Some Naphthalene Diimide G-Quadruplex Ligands. *Mol. Pharmacol.* **2013**, *83*, 470–480.
